# Pd(II)/PIDA‐Enabled Migratory Triple Functionalization of Terminal Alkenes via a 1,2‐C/Pd(IV) Dyotropic Rearrangement

**DOI:** 10.1002/anie.202518735

**Published:** 2025-10-23

**Authors:** Chen‐Xu Liu, Qian Wang, Jieping Zhu

**Affiliations:** ^1^ Laboratory of Synthesis and Natural Products (LSPN) Institute of Chemical Sciences and Engineering, Ecole Polytechnique Fédérale de Lausanne, EPFL‐SB‐ISIC‐LSPN BCH 5304 Lausanne 1015 Switzerland

**Keywords:** Dyotropic rearrangement, Heterocycle, High valent palladium, Oxypalladation, PIDA

## Abstract

We report a Pd(II)‐catalyzed migratory triple functionalization of terminal alkenes. The reaction of homoallylic amides with phenyliodine(III) diacetate (PIDA) under Pd(II) catalysis delivers 6‐acetoxylated 5,6‐dihydro‐4*H*‐1,3‐oxazines through the formation of one C─C and two C─O bonds. Mechanistic studies suggest a sequence involving oxypalladation, oxidation of Pd(II) to Pd(IV), a 1,2‐alkyl(aryl)/Pd(IV) dyotropic rearrangement (DR), and subsequent acetoxylation. While Pd(II)/Pd(IV) catalysis with PIDA as the oxidant has enabled numerous powerful transformations, the DR reported here is unprecedented.

## Graphical abstract

Pd‐catalyzed reaction of homoallylic amides with phenyliodine(III) diacetate (PIDA) under Pd(II) catalysis delivers 6‐acetoxylated 5,6‐dihydro‐4H‐1,3‐oxazines through the formation of one C─C and two C─O bonds.

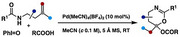

Pd‐catalyzed bond‐forming processes involving Pd(0)/Pd(II) intermediates have become indispensable tools in organic synthesis, with widespread applications in the construction of complex natural products, commodity chemicals, and pharmaceuticals. While Pd(0) and Pd(II) are the most commonly exploited oxidation states, high‐valent palladium chemistry involving Pd(III)^[^ ^1^, ^2^ ^]^ and Pd(IV)^[^ ^3^, ^4^, ^5^, ^6^, ^7^ ^]^ intermediates has advanced significantly over the past decades. Notably, owing to their distinct electronic structures and higher oxidation states, Pd(IV) complexes exhibit unique reactivities that complement those of Pd(II) species, thereby enabling new chemical transformations and expanding the scope of Pd catalysis. For example, it readily undergoes the C─X bond forming reductive elimination (RE),^[^ ^8^, ^9^, ^10^, ^11^, ^12^, ^13^, ^14^, ^15^, ^16^ ^]^ and Pd(IV) is an excellent leaving group in S_N_2‐type displacement reactions (Scheme 1a).^[^ ^17^, ^18^, ^19^, ^20^, ^21^, ^22^ ^]^

**Scheme 1:**
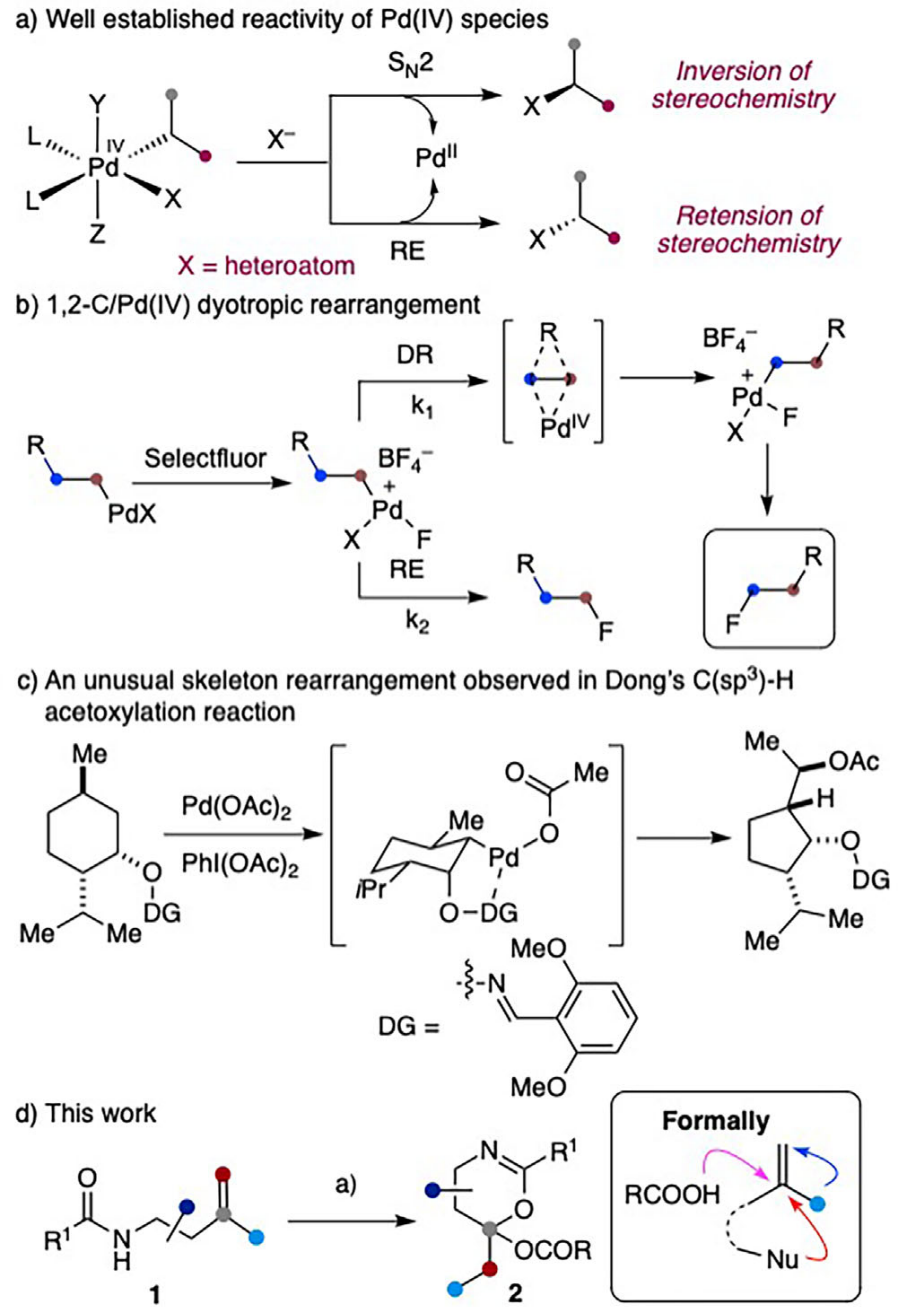
Established reactivities of Pd(IV) complexes and DR.^a)^ Pd(MeCN)_4_(BF_4_)_2_ (10 mol%), PIDA (2.0 equiv.), 5 Å MS, MeCN, RT; or PhIO (2.2 equiv.), RCO_2_H, then **1**, Pd(MeCN)_4_(BF_4_)_2_ (10 mol%), 5 Å MS, MeCN, RT. *Abbrev*. RE = reductive elimination, DR = dyotropic rearrangement, PIDA = phenyliodine(III) diacetate. MS = molecular sieves.

The Pd(IV)‐based dyotropic rearrangement (DR), recently uncovered in our laboratory,^[^ ^23^, ^24^, ^25^, ^26^, ^27^ ^]^ represents a novel reactivity mode of Pd(IV) species (Scheme 1b). To date, F^+^‐based oxidants, particularly Selectfluor {1‐Chloromethyl‐4‐fluoro‐1,4‐diazoniabicyclo[2.2.2]octane bis(tetrafluoroborate)}, remain the only class of oxidants capable of promoting both the Pd(II) to Pd(IV) oxidation and the subsequent DR.^[^ ^28^, ^29^, ^30^, ^31^, ^32^, ^33^ ^]^ The reluctance of the Pd(IV) intermediate to undergo C─F bond‐forming RE,^[^ ^34^ ^]^ combined with the low nucleophilicity of fluoride and the relatively facile DR (k_1_ > k_2_, Scheme 1b)^[^ ^35^ ^]^ renders the DR competitive with other established reaction pathways.

In addition to Selectfluor, other oxidants are known to oxidize Pd(II) to Pd(IV) species. Among these, phenyliodine(III) diacetate (PIDA) has been closely associated with the development of Pd(IV) chemistry,^[^ ^36^, ^37^, ^38^, ^39^ ^]^ particularly in the dioxygenation and amidoacetoxylation of alkenes.^[^ ^8^, ^9^, ^10^, ^11^, ^12^, ^13^, ^14^, ^15^, ^16^, ^17^, ^18^, ^19^, ^20^, ^21^, ^22^ ^]^ In these transformations, a C(sp^3^)─Pd(IV) intermediate is proposed (Scheme 1a, X = OAc), which typically undergoes either inner‐sphere RE or an S_N_2‐type displacement by acetate to furnish the observed products. DRs of such C(sp^3^)─Pd(IV) complexes have not been reported. However, in at least two instances, rearrangement products have been observed either as a minor product^[^ ^40^ ^]^ or as unexpected outcomes in a specific molecular setting.^[^ ^41^ ^]^ For example, in their studies on Pd‐catalyzed C(sp^3^)─H acetoxylation, Dong and coworkers reported an unexpected ring contraction of a menthol‐derived oxime under their reaction conditions [Pd(OAc)_2_ (10 mol%), PIDA (1.3 equiv.)] (Scheme 1c).

As part of our ongoing research on Pd‐based DR, we sought to explore alternative oxidants capable of triggering the domino sequence involving Pd‐oxidation followed by DR. Herein, we report that *N*‐acyl homoallylic amines **1** undergo a domino diacyloxylation of alkenes in the presence of a catalytic amount of Pd(MeCN)_4_(BF_4_)_2_ (10 mol%) and PIDA (2.0 equiv.), delivering 5,6‐dihydro‐4*H*‐1,3‐oxazines **2** in good yields (Scheme 1d).^[^ ^42^, ^43^ ^]^ Alternatively, using (diacyloxyiodo)benzene, generated in situ from a carboxylic acid and iodosobenzene, allowed the incorporation of diverse acyloxy groups at the C_6_ position of the 1,3‐oxazines **2**. Enabled by a 1,2‐alkyl(aryl)/Pd(IV) DR, this transformation represents a formal migratory triple functionalization of 1,1‐disubstituted alkenes, forming one C─C and two C─O bonds across a double bond.^[^ ^44^, ^45^ ^]^ While the PdX_2_/PIDA combination has been extensively used to access C(sp^2^)─Pd(IV) and C(sp^3^)─Pd(IV) species, their involvement in DRs has, to the best of our knowledge, not been previously reported.

Homoallylic amide **1a** was selected as a benchmark substrate for the optimization of the reaction conditions. As summarized in Table 1, acetonitrile emerged as the solvent of choice (entries 1–4), and Pd(MeCN)_4_(BF_4_)_2_ (10 mol%) proved to be the optimal Pd(II) source among those tested (entries 1, 5, 6). Using 2 equiv. of PIDA gave a higher yield of product **2a** compared to 1.5 equiv. (entries 7 versus 1), whereas further increasing the amount has no beneficial effect on the reaction efficiency (entries 7, 8). The addition of 5 Å molecular sieves (MS) improved the yield to 74% (entry 9), while the inclusion of potassium carbonate was detrimental (entry 10).

**Table 1:** Survey of reaction conditions^a)^.

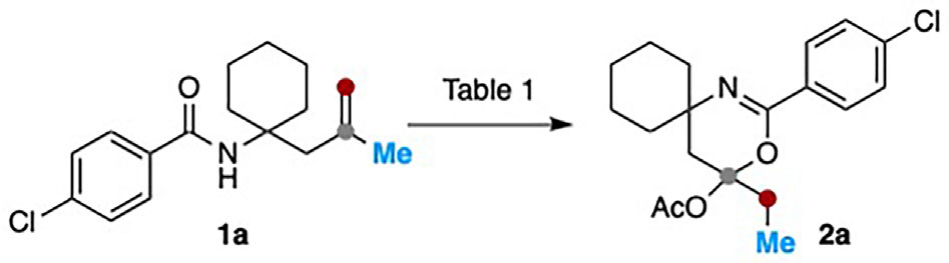				
Entryy	PdX_2_	Solventt	Additive	Yield%^b)^
1	Pd(MeCN)_4_(BF_4_)_2_	MeCN	–	57^c)^
2	Pd(MeCN)_4_(BF_4_)_2_	DMF	–	20^c)^
3	Pd(MeCN)_4_(BF_4_)_2_	DCE	–	31^c)^
4	Pd(MeCN)_4_(BF_4_)_2_	DCM	–	29^c)^
5	PdCl_2_(MeCN)_2_	MeCN	–	30^c)^
6	Pd(OAc)_2_	MeCN	–	<5^c)^
7	Pd(MeCN)_4_(BF_4_)_2_	MeCN	–	68^d)^
8	Pd(MeCN)_4_(BF_4_)_2_	MeCN	–	66^e)^
9	Pd(MeCN)_4_(BF_4_)_2_	MeCN	5 Å MS (50 mg)	74^d)^,70^f)^
10	Pd(MeCN)_4_(BF_4_)_2_	MeCN	K_2_CO_3_ (1.0 equiv.) mg)	10

^a)^ General conditions: **1a** (0.1 mmol), Pd(MeCN)_4_(BF_4_)_2_ (10 mol%), PIDA, solvent (1.0 mL, *c* 0.1 M), additive, RT, 1 h.

^b)^ NMR yield.

^c)^ 1.5 equiv. of PIDA.

^d)^ 2.0 equiv. of PIDA.

^e)^ 2.5 equiv. of PIDA.

^f)^ Isolated yield.

With the optimum conditions in hand [Pd(MeCN)_4_(BF_4_)_2_ (10 mol%), PIDA (2.0 equiv.), MeCN (*c* 0.1 M), 5 Å MS, RT], we next explored the scope of this reaction (Scheme 2a). A variety of acyl groups were tolerated, including benzoyl and its derivatives bearing either electron‐withdrawing (Cl, F, NO_2_) or electron‐donating substituents (OMe, Me) at different positions (**2a**‐**2f**). Polysubstituted 5,6‐dihydro‐1,3‐oxazines, including spirocyclic compounds, were also readily accessed. Notably, the reaction of 2,4‐disubstituted‐4‐amidobutene‐1 with PIDA furnished tetrasubstituted 5,6‐dihydro‐4*H*‐1,3‐oxazines **2o** and **2p** with high diastereoselectivities. These results stand in sharp contrast to the fluorocyclization of the same amides, which proceeds with poor selectivity.^[^ ^42^ ^]^ A range of aliphatic chains (**2q**‐**2s**), including functionalized ones (**2t**‐**2v**) as well as a phenyl group (**2w**), participated in the migration process to deliver the corresponding 1,3‐oxazines. Notably, products arising from a 1,3‐Pd(IV) shift were not observed in the conversion of **1q**‐**1v** to **2q**‐**2v**.^[^ ^42^, ^46^ ^]^

**Scheme 2:**
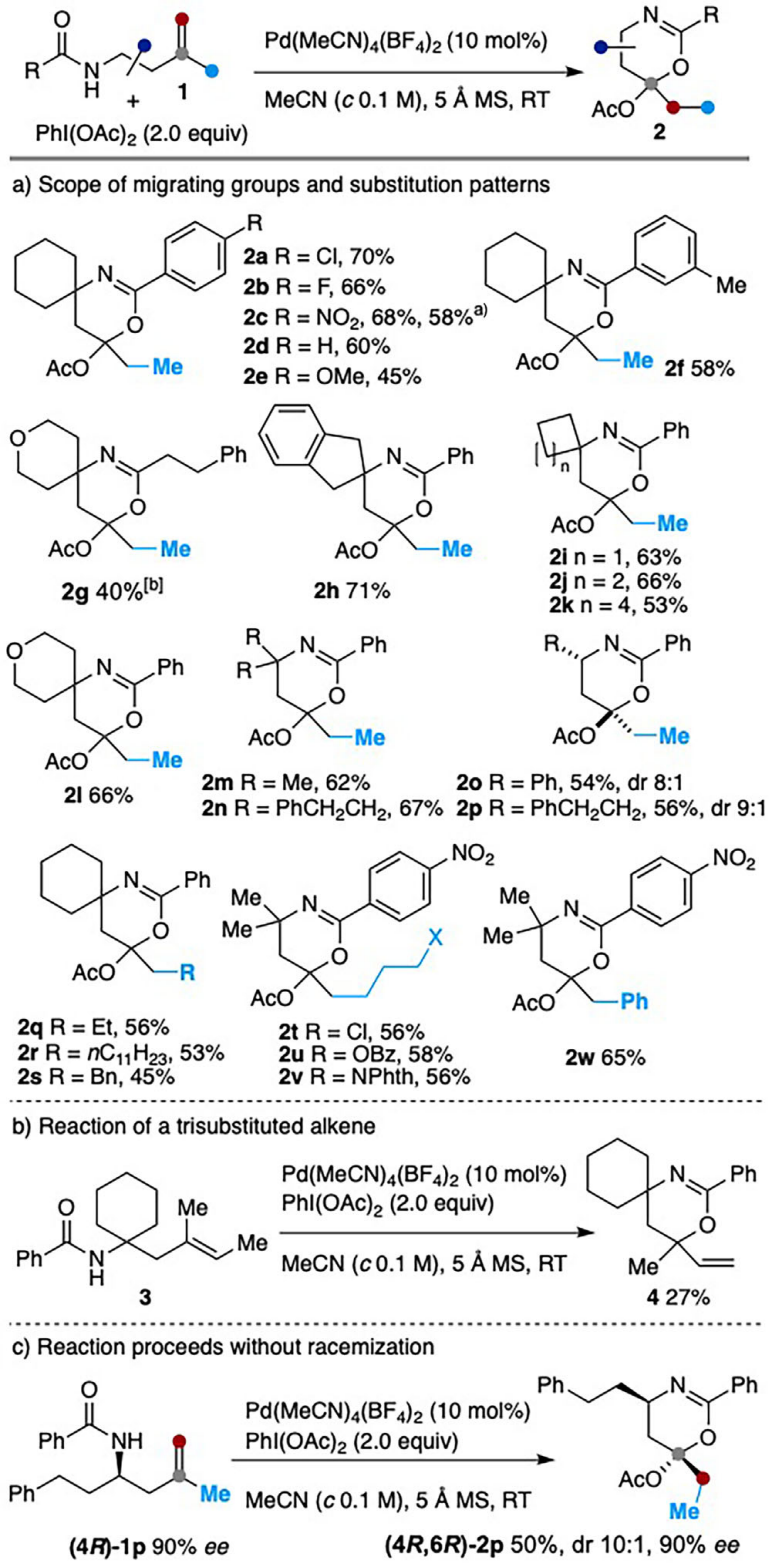
Substrate scope. The reaction was performed on a 0.1 mmol scale unless otherwise specified. ^a)^ Reaction conducted on a 1.0 mmol scale using 2 mol% of Pd catalyst and 1.5 equiv. of PIDA. ^b)^ Ketone resulting from the hydrolysis of **2g** was isolated in 21% yield.

The reaction is, however, not without limitations. When a trisubstituted alkene **3** was subjected to the standard conditions, a sequence of oxypalladation followed by β‐hydride elimination furnished product **4** in 27% (Scheme 2b). In contrast, a substrate bearing a tetrasubstituted alkene and homoallylic amides lacking substitution at the methylene linker gave rise to a complex reaction mixture.

It is also noteworthy that bis[(trifluoroacetoxy)iodo]benzene (PIFA) proved ineffective for this transformation. Specifically, the reaction of **1h** or **1j** with PIFA, instead of PIDA, under otherwise standard conditions resulted in a complex mixture.

Finally, starting from enantioenriched homoallylic amide (4*R*)‐**1p**, 1,3‐oxazine (4*R*,6*R*)‐**2p** was obtained without racemization (Scheme 2c).

To introduce diverse acyloxy groups at the C_6_ position of 5,6‐dihydro‐4*H*‐1,3‐oxazines, various (diacyloxyiodo)benzenes were prepared from carboxylic acid and iodosobenzene following literature procedures.^[^ ^47^, ^48^ ^]^ Without purification, the crude product PhI(OCOR)_2_ was directly used as both the oxidant and the donor of the acyloxy group. As shown in Scheme 3, a broad range of carboxylic acids could be incorporated at the C_6_ position of the heterocycle **2**. Carboxylic acids bearing functional groups such as alkyl halide (**2z**), ether (**2aa**), and alkyne (**2ad**) were tolerated. Cyclopropanecarboxylic acid and 2‐cyclobutylacetic acid also proved to be competent reaction partners, furnishing **2ae** and **2af** in 60% and 56% yield, respectively. Notably, heterocycle‐containing carboxylic acids were compatible as well, leading to products **2aj** and **2ag**. Finally, excellent diastereoselectivity (dr 12:1) was observed in the formation of compound **2ak**, whose relative stereochemistry was confirmed by X‐ray crystallographic analysis.^[^ ^49^ ^]^

**Scheme 3:**
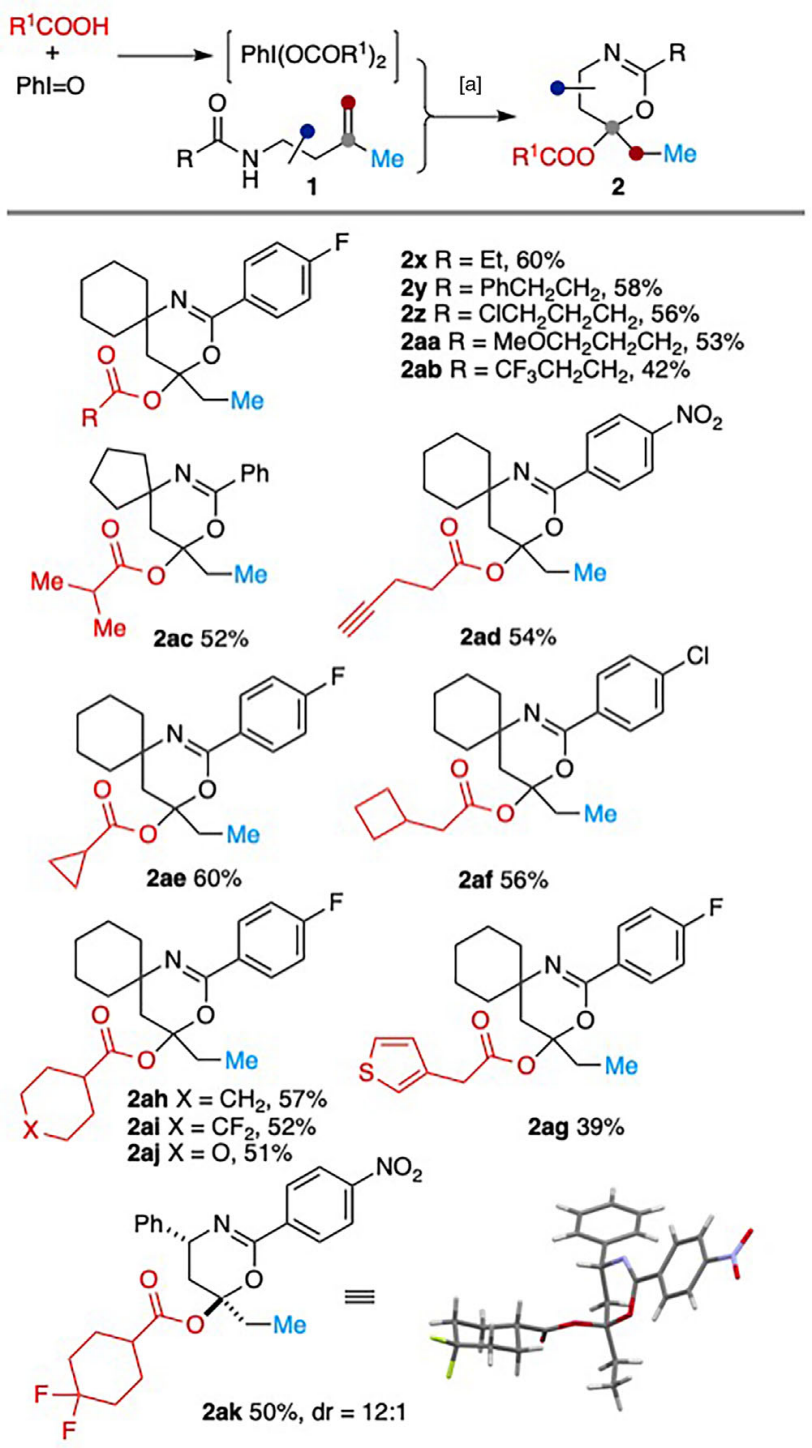
^a)^ Reaction conditions: PhIO (2.2 equiv.), RCOOH (2.0 equiv.), DCM, MgSO_4_ (100.0 mg), RT, 1.0 h, filtration and evaporation of solvent, then **1** (0.1 mmol), Pd(MeCN)_4_(BF_4_)_2_ (10 mol%), 5 Å MS, MeCN (1.0 mL, *c* 0.1 M), RT, 2 h.

Control experiments were conducted to probe the reaction mechanism. Omitting either the Pd salt or PIDA^[^ ^50^, ^51^, ^52^ ^]^ under otherwise standard conditions resulted in partial decomposition and recovery of the starting materials, indicating that both are essential for the reaction to proceed. Treatment of [^13^C]‐labeled **1j** with PIDA under the standard conditions afforded [^13^C]‐**2j**, clearly indicating migration of the methyl group to the terminal sp^2^ carbon (Scheme 4a). When **1j** was reacted with a stoichiometric amount of Pd(MeCN)_4_(BF_4_)_2_ in CD_3_CN, a Pd(II) intermediate **5** was obtained and fully characterized by both ^1^H and ^13^C NMR spectroscopy. High‐resolution mass spectrometry gave an exact mass of 389.0847 (calcd for C_18_H_23_N_2_OPd^+^ = 389.0840), consistent with Pd complex **5**, ligated by a single molecule of acetonitrile (L = MeCN, *n* = 1). Upon addition of PIDA to this solution, product **2j** was obtained in 52% yield, supporting the involvement of Pd(II) complex **5** as a plausible intermediate en route to **2j**. Aiming at preparing crystalline Pd(II) complex, 4,4‐di‐*tert*‐butyl‐2,2′‐bipyridine (**6**) was added to the acetonitrile solution of **5**. Unexpectedly, instead of forming a more stable bipyridine‐ligated Pd‐complex, a retro‐oxypalladation occurred, regenerating the starting material **1j**. This observation suggests a *syn*‐orientation between C_a_─O and C_b_─Pd bonds in complex **5**.^[^ ^53^ ^]^

**Scheme 4:**
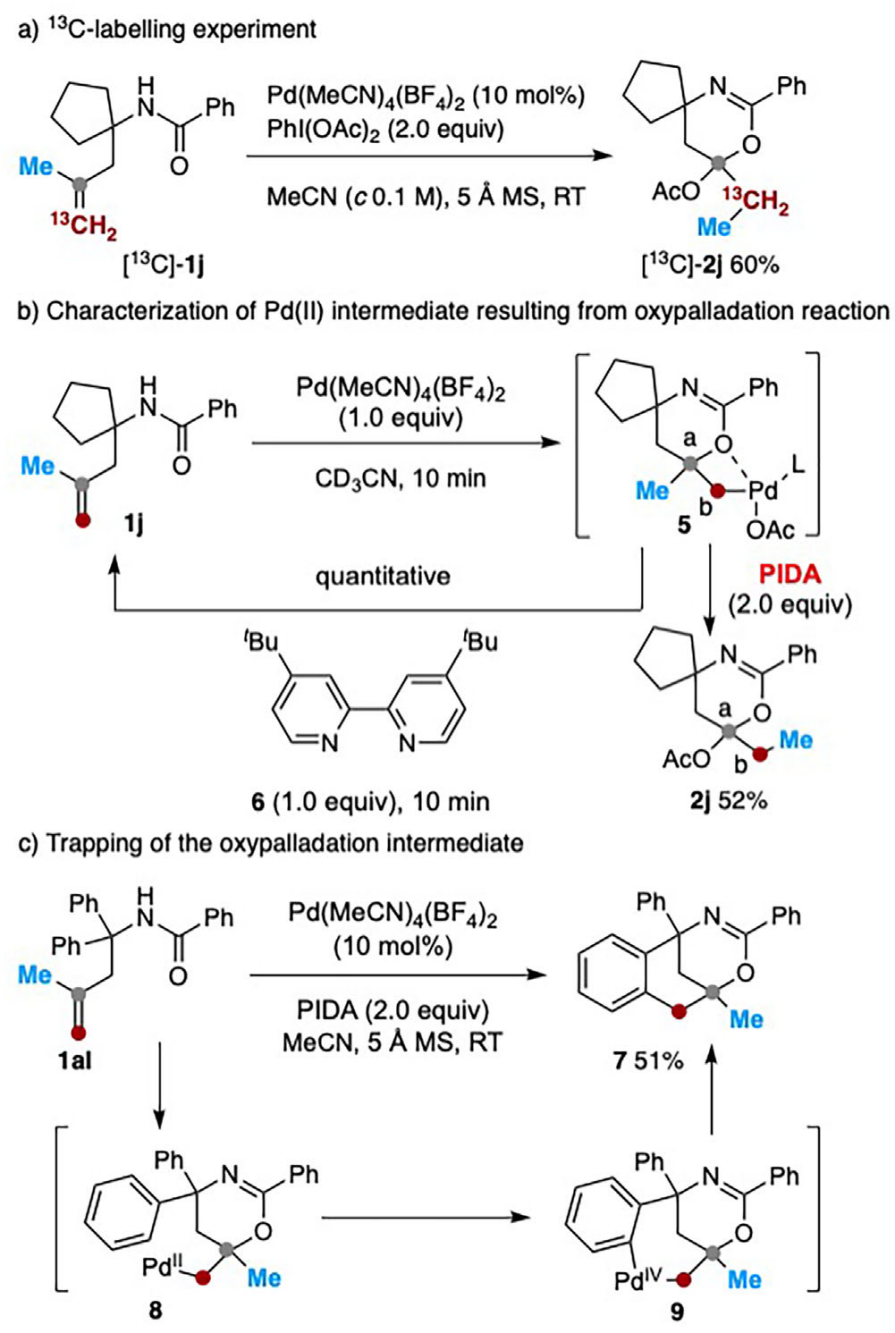
Mechanistic studies.

Further support for the intermediacy of **5** came from a trapping experiment. Reaction of **1ai** with PIDA under the standard conditions afforded the bridged compound **7** in 51% yield, along with 1,3‐oxazine **2al** (10%). Mechanistically, this transformation likely proceeds via a 6‐*exo*‐trig oxypalladation of **1ai**, followed by oxidation of the resulting Pd(II) species **8** and subsequent C─H activation to form palladacycle **9**.^[^ ^54^, ^55^, ^56^ ^]^ RE from **9** would then deliver product **7**. To incorporate the stereochemical outcome into the mechanistic discussion, α‐secondary amides **1** were used as model substrates. Based on the aforementioned control experiments, the reaction is proposed to begin with a 6‐*exo*‐trig oxypalladation of **1**, generating the Pd(II) intermediate **10**. Oxidation of **10** by PIDA would furnish the corresponding Pd(IV) species **11**. A 1,2‐R/Pd(IV) DR of **11** would lead to intermediate **12**, which, upon C─O bond‐forming RE, delivers product **2** with concurrent regeneration of the Pd(II) catalyst (Scheme 5). Alternatively, the Pd(IV) species may undergo elimination assisted by the oxygen lone pair to generate the oxonium species **13**. Subsequent nucleophilic attack by acetate on the face opposite to the C_3_‐substituent would then furnish product **2** with high stereoselectivity. Monitoring the reaction progress revealed that the diastereomeric ratio remained constant, suggesting that oxonium **13** may serve as an intermediate en route to **2**. Moreover, the 1,2‐C/Pd(IV) DR (**11** to **12**) is likely kinetically faster than either the C─O bond‐forming RE or the S_N_2‐type reaction from **11**, as compound **14** was not detected from the reaction mixture.

**Scheme 5:**
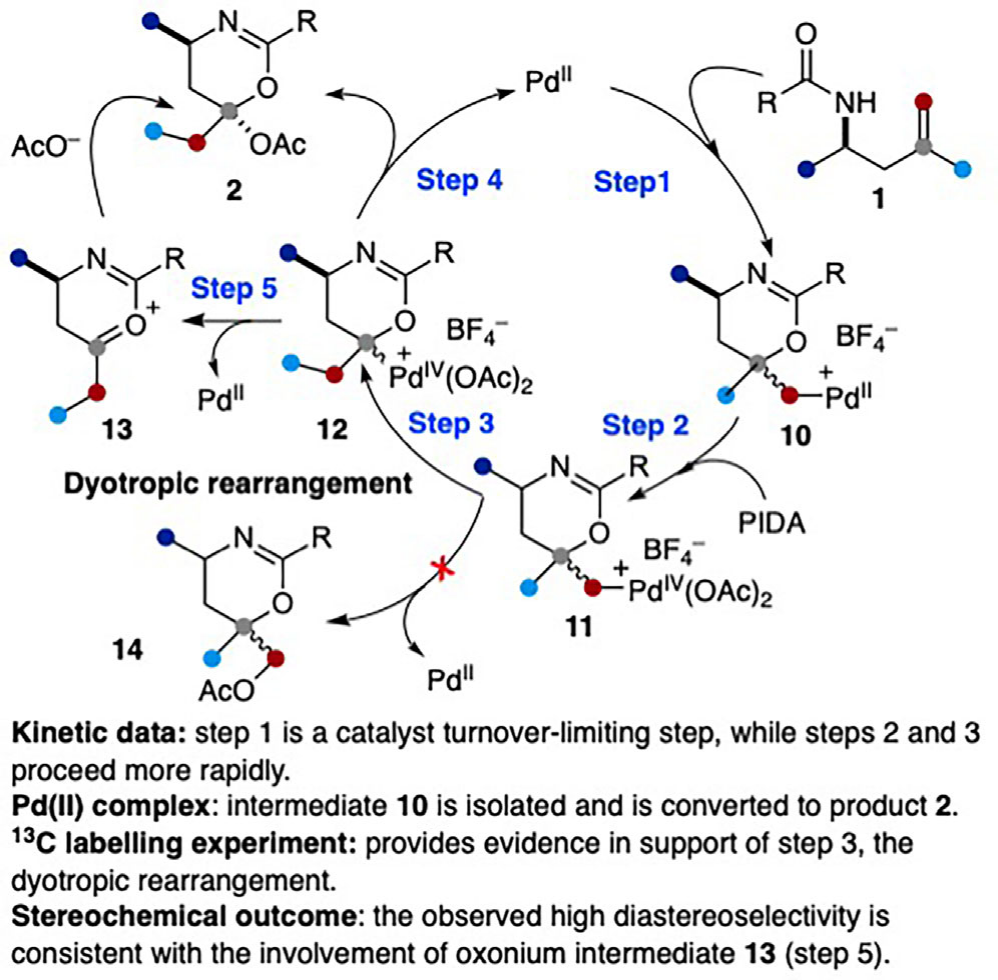
Proposed reaction mechanism.

Kinetic analysis of the reaction of **1h** with PIDA by the initial rates method revealed a first‐order dependence on both **1h** and the palladium catalyst and a zero‐order dependence on PIDA (Figure 1). These results implicate either Pd–alkene coordination or the oxypalladation step as the turnover‐limiting event in the catalytic cycle).

**Figure 1:**
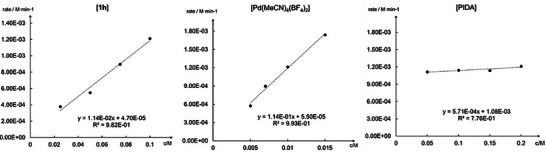
Kinetic study. The reaction displays first‐order dependence on both substrate **1h** and the Pd(II) catalyst but zero order on PIDA.

In summary, we have developed a novel Pd(II)‐catalyzed migratory triple functionalization of terminal alkenes. Operating via a Pd(II)/Pd(IV) catalytic cycle with PIDA as the oxidant, this transformation converts homoallylic amides into 6‐acetoxylated 5,6‐dihydro‐4*H*‐1,3‐oxazines through the formation of one C─C and two C─O bonds. Mechanistic studies suggest a sequence involving oxypalladation, Pd(II)‐to‐Pd(IV) oxidation, a 1,2‐alkyl(aryl)/Pd(IV) DR, and a final acetoxylation step. Although PIDA is commonly employed in Pd(II)/Pd(IV) catalysis, the DR disclosed here is unprecedented.

## Supporting Information

The authors have cited additional references within the Supporting Information.^[^ ^57^, ^58^, ^59^ ^]^

## Conflict of Interests

The authors declare no conflict of interest.

## Supporting information



Supporting Information

Supporting Information

## Data Availability

The data that support the findings of this study are available in the Supporting Information of this article.
