# Supplementary material for: Pd(II)/PIDA‐Enabled Migratory Triple Functionalization of Terminal Alkenes via a 1,2‐C/Pd(IV) Dyotropic Rearrangement
Source: Angew Chem Int Ed Engl. 2025 Oct 23;64(50):e202518735. doi: 10.1002/anie.202518735 (PMC12684323; doi:10.1002/anie.202518735)

Supplementary Information for

Pd(II)/PIDA-Enabled Migratory Triple Functionalization of Terminal Alkenes via a 1,2-C/Pd(IV) Dyotropic Rearrangement

Chen-Xu Liu, Qian Wang, Jieping Zhu*

Laboratory of Synthesis and Natural Products (LSPN), Institute of Chemical Sciences and Engineering, Ecole Polytechnique Fédérale de Lausanne, EPFL-SB-ISIC-LSPN, CH-1015 Lausanne, Switzerland.

* Email: jieping.zhu@epfl.ch

Table of Contents

General methods S3

Optimization of chemoselective palladium-catalyzed dyotropic rearrangement reactions S4

General procedure for the synthesis of substrates[1] S5

General procedure for the palladium catalyzed dyotropic rearrangement reaction S11

General procedure for the evaluation of trisubstituted substrate S33

General procedure for the evaluation of chiral (*R*)-**1p** in palladium-catalyzed dyotropic rearrangement S35

Mechanistic study S39

X-Ray crystallographic data S50

References...S53

Copies of the NMR spectra...S54

General methods

Reagents and solvents were purchased from commercial sources (Aldrich, Acros, Merck, Fluka and VWR international) and used without further purification. Flash column chromatography was performed using Silicycle P60 silica: 230-400 mesh (40-63 μm) silica. Reactions were monitored using Merck Kieselgel 60F254 aluminium plates. TLC was visualized by UV fluorescence (254 nm) then by $KMnO_4$ or phosphomolybdic acid. NMR spectra were recorded on a Brüker Avance III-400, Brüker Avance-400 or Brüker DPX-400 spectrometer at room temperature. Chemical shifts (δ) were reported in parts per million (ppm) relative to residual solvent peaks rounded to the nearest 0.01 for proton and 0.1 for carbon (ref: $CDCl_3$ [^1H: 7.26, ^{13}C: 77.0]). Coupling constants (J) were reported in Hz to the nearest 0.1 Hz. Peak multiplicity was indicated as follows s (singlet), d (doublet), t (triplet), q (quartet), p (pentet), sext (sextet), h (septet), m (multiplet) and br (broad). Attribution of peaks was done using the multiplicities and integrals of the peaks. HRMS measurements were performed by the mass spectrometry service of the EPFL by ESI-TOF using a QTOF Ultima from Waters, or APPIFT-ICR using a linear ion trap Fourier transform ion cyclotron resonance mass spectrometer from Thermo Scientific. High resolution mass values are given in m/z. Melting points were measured using a Stuart SMP30. Infrared (IR) data were recorded on an Alpha-P Bruker FT-IR Spectrometer. Absorbance frequencies are reported in reciprocal centimeters (cm^{-1}). Enantiomeric excesses were measured on an Waters HPLC Investigator system using chiral stationary phase columns. Optical rotations α_D were obtained with a Jasco P-2000 polarimeter (589 nm).

Optimization of chemoselective palladium-catalyzed dyotropic rearrangement reactions

Table S1. Survey of Reaction Conditions

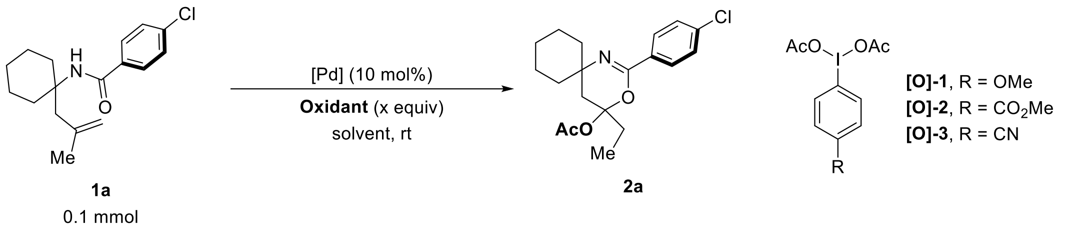

Entry[a]	[Pd]	Oxidant	Solvent	x equiv	Addi-tive	Conver-sion	Yield[b]
1	$Pd(MeCN)_4(BF_4)_2$	PIDA	MeCN	1.5	--	85	57
2	$Pd(MeCN)_2Cl_2$	PIDA	MeCN	1.5	--	80	30
3	$Pd(OAc)_2$	PIDA	MeCN	1.5	--	20	<5
4	$Pd(MeCN)_4(BF_4)_2$	**[O]-1**	MeCN	1.5	--	83	48
5	$Pd(MeCN)_4(BF_4)_2$	**[O]-2**	MeCN	1.5	--	89	51
6	$Pd(MeCN)_4(BF_4)_2$	**[O]-3**	MeCN	1.5	--	87	52
7	$Pd(MeCN)_4(BF_4)_2$	PIDA	DMF	1.5	--	75	20
8	$Pd(MeCN)_4(BF_4)_2$	PIDA	DCE	1.5	--	70	31
9	$Pd(MeCN)_4(BF_4)_2$	PIDA	DCM	1.5	--	73	29
10	$Pd(MeCN)_4(BF_4)_2$	PIDA	MeCN	2.0	--	>95	68
11	$Pd(MeCN)_4(BF_4)_2$	PIDA	MeCN	2.5	--	>95	66
12	$Pd(MeCN)_4(BF_4)_2$	PIDA	MeCN	2.0	3Å MS[c]	>95	63
13	$Pd(MeCN)_4(BF_4)_2$	PIDA	MeCN	2.0	4Å MS[d]	>95	65
14	$Pd(MeCN)_4(BF_4)_2$	PIDA	MeCN	2.0	5Å MS[e]	>95	74 (70)[g]
15	$Pd(MeCN)_4(BF_4)_2$	PIDA	MeCN	2.0	K_2CO_3[f]	>95	10
16	--	PIDA	MeCN	1.5	--	<5%	--

[a]Conditions: **1a** (0.1 mmol), [Pd] (10 mol%) and oxidant in solvent (1 mL) at room temperature. [b]NMR yields using mesitylene as an internal standard. [c]3Å MS (50 mg). [d]4Å MS (50 mg). [e]5Å MS (50 mg). [f]K_2CO_3 (1.0 equiv). [g]Isolated yield.

General procedure for the synthesis of substrates[1]

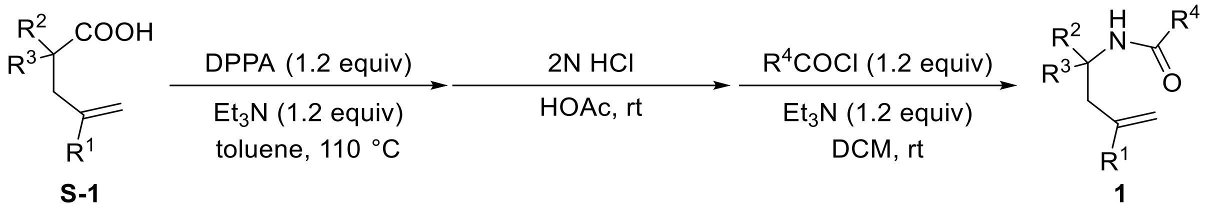

Step 1: To a solution of acid **S-1**[2-4] and Et_3N (1.2 equiv) in toluene (1 mmol/mL) at 0 ºC was added diphenyl phosphoryl azide (1.2 equiv) slowly. The mixture was stirred at 0 ºC for 1 h, at room temperature for 1 h and at 110 ºC for 5 h. After being cooled to room temperature, the mixture was washed with H_2O and brine, dried over Na_2SO_4, filtered and evaporated. The product was directly used for the next step.

Step 2: To the resulting crude product was added HOAc (4 mmol/mL) at 0 ºC, followed by aqueous 2N HCl. After being stirred at room temperature for 12 h, the mixture was washed with pentane, made basic with aqueous 2N NaOH and extracted with pentane. The extracts were combined, dried over Na_2SO_4 and filtered. Evaporation of pentane provided the amine as a liquid. The product was directly used for the next step.

Step 3: To a solution of the amine and Et_3N (1.2 equiv) in DCM (1 mmol/mL) at 0 ºC was added the acyl chloride (1.2 equiv) slowly. The mixture was stirred at 0 ºC for 15 min and at room temperature for 4 h. After that, the mixture was washed with H_2O and brine, dried over Na_2SO_4, filtered and evaporated. The residue was purified by silica gel flash column chromatography (eluent: hexanes / ethyl acetate = 20:1 → 10:1) to give the desired product **1**.

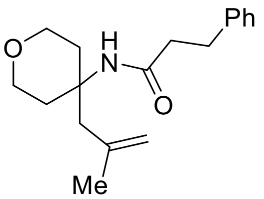

1g. Colorless oil (270.0 mg, 47% yield, 2 mmol scale).

Analytical data for **1g**:

^{1}H NMR (500 MHz, $CDCl_3$) δ 7.29 (dd, J = 8.3, 6.9 Hz, 2H), 7.23 – 7.18 (m, 3H), 4.96 (s, 1H), 4.86 (p, J = 1.6 Hz, 1H), 4.61 (d, J = 2.2 Hz, 1H), 3.67 (dt, J = 12.0, 3.9 Hz, 2H), 3.35 (td, J = 11.6, 2.3 Hz, 2H), 2.96 (t, J = 7.5 Hz, 2H), 2.51 (s, 2H), 2.49 (t, J = 7.6 Hz, 2H), 2.01 (dq, J = 14.1, 2.6 Hz, 2H),

1.70 (t, J = 1.1 Hz, 3H), 1.66 – 1.60 (m, 2H).

^{13}C NMR (126 MHz, $CDCl_3$) δ 171.8, 141.2, 140.6, 128.6, 128.4, 126.4, 115.4, 63.4, 53.7, 45.1, 39.2, 35.4, 31.5, 24.9.

IR (neat) ν_{max} (cm^{-1}) = 2994, 2989, 2890, 2854, 1670, 1163, 1144, 1129, 1112, 1075, 1066, 695.

HRMS (nanochip-ESI/LTQ-Orbitrap) m/z: $[M + H]^+$ Calcd for $C_{18}H_{26}NO_2^+$ 288.1958; Found 288.1962.

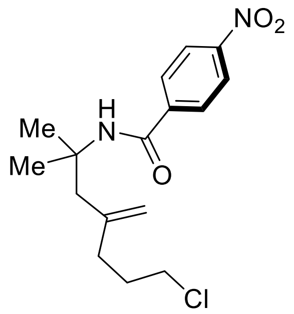

1t. Colorless oil (1.82 g, 56% yield, 10 mmol scale).

Analytical data for **1t**:

^{1}H NMR (400 MHz, $CDCl_3$) δ 8.26 – 8.22 (m, 2H), 7.86 – 7.83 (m, 2H), 6.09 (s, 1H), 4.97 (q, J = 1.5 Hz, 1H), 4.87 – 4.84 (m, 1H), 3.51 (t, J = 6.4 Hz, 2H), 2.58 (s, 2H), 2.25 – 2.21 (m, 2H), 1.94 – 1.87 (m, 2H), 1.49 (s, 6H).

^{13}C NMR (101 MHz, $CDCl_3$) δ 164.9, 149.3, 144.9, 141.4, 127.8, 123.8, 115.2, 54.4, 45.1, 44.4, 34.4, 30.7, 27.7.

IR (neat) ν_{max} (cm^{-1}) = 2978, 2947, 2852, 1768, 1684, 1647, 1588, 1477, 1329, 1248, 1173, 951, 933, 848, 821, 766, 729, 643.

HRMS (ESI/QTOF) m/z: $[M + H]^+$ Calcd for $C_{16}H_{22}ClN_2O_3^+$ 325.1313; Found 325.1311.

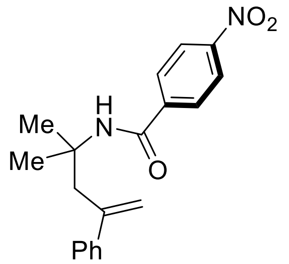

1w. Colorless oil (311.1 mg, 48% yield, 2 mmol scale).

Analytical data for **1w**:

^{1}H NMR (500 MHz, $CDCl_3$) δ 8.07 – 8.03 (m, 2H), 7.42 – 7.39 (m, 2H), 7.32 – 7.27 (m, 2H), 7.26 – 7.22 (m, 1H), 7.21 – 7.18 (m, 2H), 5.75 (s, 1H), 5.34 (d, J = 1.7 Hz, 1H), 5.17 (d, J = 1.7 Hz, 1H), 3.00 (s, 2H), 1.47 (s, 6H).

^{13}C NMR (126 MHz, $CDCl_3$) δ 164.5, 149.1, 145.4, 142.7, 141.0, 128.9, 127.6, 127.5, 126.5, 123.3, 118.7, 54.7, 45.9, 27.1.

IR (neat) ν_{max} (cm^{-1}) = 2994, 2989, 2869, 2863, 2855, 1648, 1637, 1629, 1541, 1535, 1488, 1312, 1286, 1173, 1129, 1039, 782, 728, 711, 692, 670, 665.

HRMS (ESI/QTOF) m/z: $[M + H]^+$ Calcd for $C_{19}H_{21}N_2O_3^+$ 325.1547; Found 325.1548.

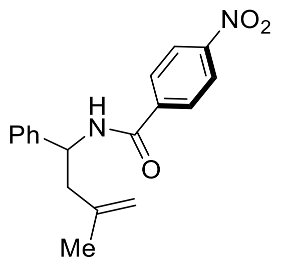

1ak. White solid (759.8 mg, 49% yield, 5 mmol scale).
Analytical data for **1ak**:
m.p. = 123 ~ 125 °C.

^{1}H NMR (400 MHz, $CDCl_3$) δ 8.26 – 8.23 (m, 2H), 7.91 – 7.87 (m, 2H), 7.38 – 7.33 (m, 4H), 7.32 – 7.26 (m, 1H), 6.52 (d, J = 7.5 Hz, 1H), 5.32 (ddd, J = 8.9, 7.4, 6.3 Hz, 1H), 4.88 (p, J = 1.7 Hz, 1H), 4.84 – 4.82 (m, 1H), 2.69 – 2.56 (m, 2H), 1.78 (s, 3H).

^{13}C NMR (101 MHz, $CDCl_3$) δ 164.8, 149.6, 142.1, 141.6, 140.1, 128.8, 128.1, 127.6, 126.3, 123.8, 114.1, 51.9, 45.1, 22.0.

IR (neat) ν_{max} (cm^{-1}) = 2976, 2833, 1720, 1708, 1688, 1638, 1619, 1557, 1542, 1512, 1492, 1467, 1422, 1355, 1312, 1269, 1244, 1173, 1087, 1043, 1022, 988, 922, 877, 842, 837, 774, 755, 666, 642, 610.

HRMS (ESI/QTOF) m/z: $[M + H]^+$ Calcd for $C_{18}H_{19}N_2O_3^+$ 311.1390; Found 311.1389.

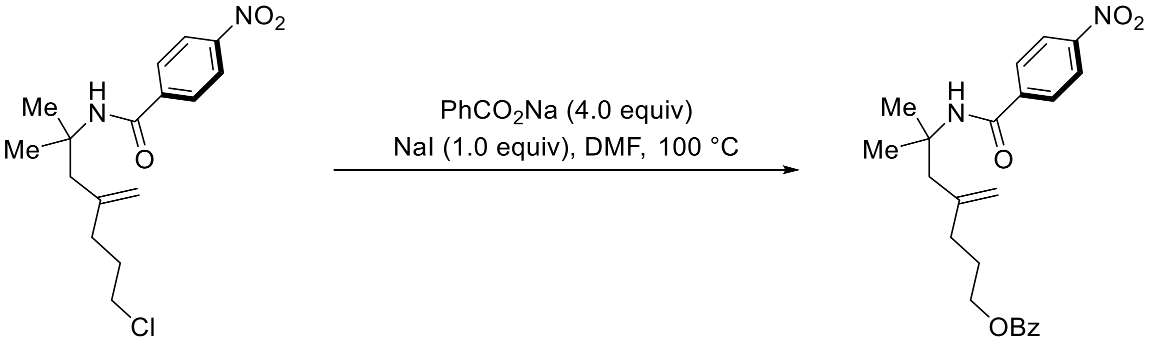

1t **1u**

To a solution of **1t** (324.1 mg, 1.0 mmol) and NaI (148.9 mg, 1.0 mmol, 1.0 equiv) in DMF (10 mL) was added sodium benzoate (576.4 mg, 4.0 mmol, 4.0 equiv) slowly. The mixture was stirred at 100 ºC for 12 h. After being cooled to room temperature, diethyl ether (20 mL) was added and the mixture was washed with H_2O and brine, dried over Na_2SO_4, filtered and evaporated. The residue was purified by silica gel flash column chromatography (eluent: hexanes / ethyl acetate = 10:1 → 5:1) to give the desired product **1u**.

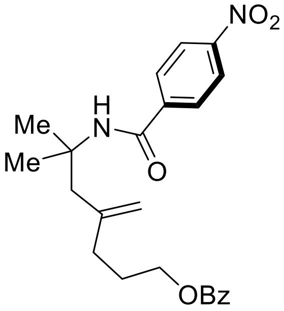

1u. Colorless oil (348.8 mg, 85% yield).

Analytical data for **1u**:

^{1}H NMR (400 MHz, $CDCl_3$) δ 8.23 – 8.19 (m, 2H), 8.01 – 7.97 (m, 2H), 7.84 – 7.81 (m, 2H), 7.57 – 7.52 (m, 1H), 7.44 – 7.39 (m, 2H), 6.07 (s, 1H), 5.02 (q, *J* = 1.5 Hz, 1H), 4.89 – 4.87 (m, 1H), 4.30 (t, *J* = 6.4 Hz, 2H), 2.61 (s, 2H), 2.27 – 2.21 (m, 2H), 1.98-1.90 (m, 2H), 1.50 (s, 6H).

^{13}C NMR (101 MHz, $CDCl_3$) δ 166.5, 164.9, 149.3, 145.3, 141.4, 132.9, 130.2, 129.5, 128.3, 127.8, 123.8, 115.0, 64.3, 54.4, 45.5, 33.9, 27.7, 27.1.

IR (neat) ν_{max} (cm^{-1}) = 2967, 2833, 1743, 1655, 1567, 1533, 1431, 1317, 1266, 1232, 1177, 1094, 994, 911, 877, 827, 771, 723, 632.

HRMS (ESI/QTOF) m/z: $[M + H]^+$ Calcd for $C_{23}H_{27}N_2O_5^+$ 411.1914; Found 411.1935.

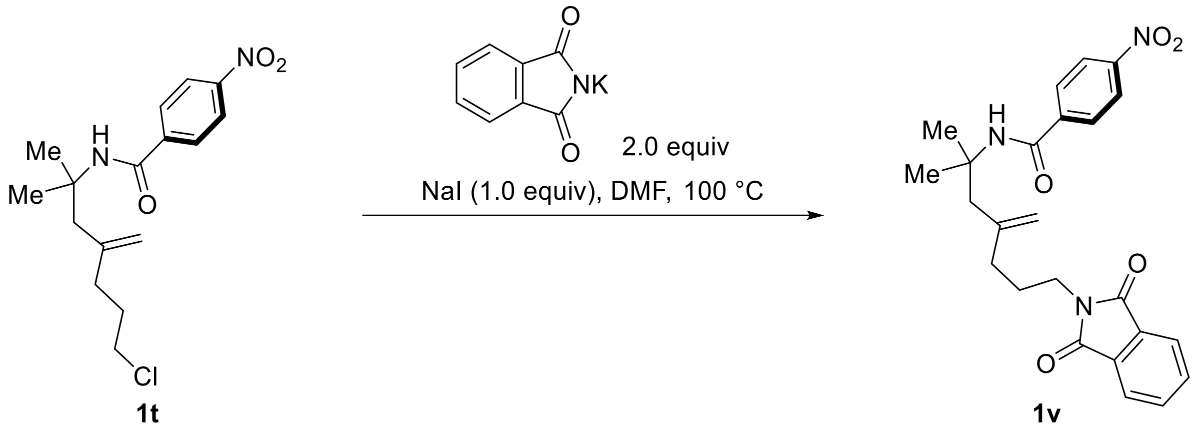

To a solution of **1t** (324.1 mg, 1.0 mmol) and NaI (148.9 mg, 1.0 mmol, 1.0 equiv) in DMF (10 mL) was added potassium phthalimide (370.4 mg, 2.0 mmol, 2.0 equiv) slowly. The mixture was stirred at 100 ºC for 12 h. After being cooled to room temperature, diethyl ether (20 mL) was added and the mixture was washed with H_2O and brine, dried over Na_2SO_4, filtered and evaporated. The residue was purified by silica gel flash column chromatography (eluent: hexanes / ethyl acetate = 10:1 → 5:1) to give the desired product **1v**.

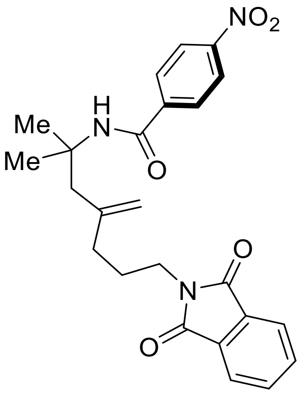

1v. White solid (343.8 mg, 79% yield).

Analytical data for **1v**:

m.p. = 132 ~ 134 °C.

^{1}H NMR (400 MHz, $CDCl_3$) δ 8.25 – 8.21 (m, 2H), 7.87 – 7.82 (m, 2H), 7.80 (dd, *J* = 5.4, 3.1 Hz, 2H), 7.70 (dd, *J* = 5.5, 3.0 Hz, 2H), 6.10 (s, 1H), 5.00 (q, *J* = 1.5 Hz, 1H), 4.85 (d, *J* = 1.7 Hz, 1H), 3.65 (t, *J* = 7.2 Hz, 2H), 2.57 (s, 2H), 2.14 (t, *J* = 7.7 Hz, 2H), 1.89 – 1.81 (m, 2H), 1.48 (s, 6H).

^{13}C NMR (101 MHz, $CDCl_3$) δ 168.4, 164.9, 149.3, 145.1, 141.4, 133.9, 132.0, 127.8, 123.8, 123.2, 114.9, 54.3, 45.7, 37.6, 34.6, 27.7, 26.8.

IR (neat) ν_{max} (cm^{-1}) = 2964, 2862, 2813, 1744, 1698, 1658, 1572, 1456, 1432, 1377, 1296, 1269, 1174, 1061, 972, 921, 871, 842, 781, 772, 718, 667, 638.

HRMS (ESI/QTOF) m/z: $[M + H]^+$ Calcd for $C_{24}H_{26}N_3O_5^+$ 436.1867; Found 436.1881.

General procedure for the palladium catalyzed dyotropic rearrangement reaction

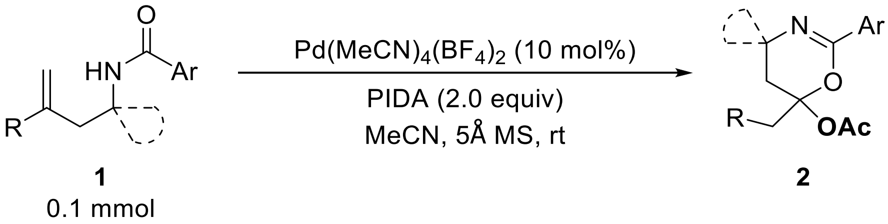

At room temperature, to a 10-mL test tube charged with a magnetic stirring bar were added substrate **1** (0.1 mmol, 1.0 equiv), PIDA (64.4 mg, 0.2 mmol, 2.0 equiv), [Pd(MeCN)$_4$(BF$_4$)$_2$] (4.5 mg, 0.01 mmol, 10 mol%), 5Å molecular sieve (50 mg) and anhydrous MeCN (1.0 mL). The reaction mixture was allowed to stir at room temperature. After the reaction was complete (monitored by TLC), diethyl ether (2 mL) was added and the reaction mixture was filtered through a short pad of silica gel with diethyl ether. The filtrate was concentrated. The residue was purified by silica gel flash column chromatography to give the desired product **2**.

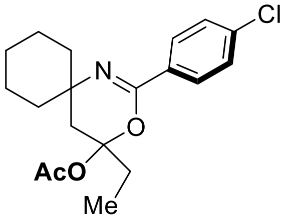

2a. Colorless oil (24.3 mg, 70% yield).

Analytical data for **2a**:

^{1}H NMR (600 MHz, CDCl$_3$) δ 7.94 (d, J = 8.2 Hz, 2H), 7.34 (d, J = 8.2 Hz, 2H), 2.81 (d, J = 14.7 Hz, 1H), 2.32 (dq, J = 14.9, 7.5 Hz, 1H), 2.18 – 2.11 (m, 1H), 2.01 (s, 3H), 1.94 – 1.86 (m, 1H), 1.82 – 1.70 (m, 2H), 1.58 – 1.49 (m, 4H), 1.49 – 1.39 (m, 4H), 1.02 (t, J = 7.5 Hz, 3H).

^{13}C NMR (151 MHz, CDCl$_3$) δ 169.1, 148.3, 136.5, 132.3, 128.9, 128.2, 103.9, 51.6, 41.9, 37.4, 31.1, 25.9, 22.1, 21.82, 21.75, 7.0.

IR (neat) ν_{max} (cm^{-1}) = 2984, 2945, 2822, 1789, 1623, 1559, 1455, 1377, 1201, 1142, 11082, 981, 921, 915, 877, 851, 793, 719, 677.

HRMS (ESI/QTOF) m/z: [M + H]$^+$ Calcd for $C_{19}H_{25}ClNO_3^+$ 350.1517; Found 350.1528.

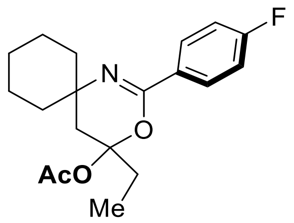

2b. Colorless oil (21.9 mg, 66% yield).

Analytical data for **2b**:

^{1}H NMR (600 MHz, $CDCl_3$) δ 8.03 – 7.98 (m, 2H), 7.05 (t, *J* = 8.7 Hz, 2H), 2.81 (d, *J* = 14.8 Hz, 1H), 2.32 (dq, *J* = 14.9, 7.5 Hz, 1H), 2.15 (dq, *J* = 14.7, 7.4 Hz, 1H), 2.01 (s, 3H), 1.94 – 1.86 (m, 1H), 1.81 – 1.69 (m, 2H), 1.59 – 1.49 (m, 4H), 1.48 – 1.40 (m, 4H), 1.02 (t, *J* = 7.5 Hz, 3H).

^{13}C NMR (151 MHz, $CDCl_3$) δ 169.2, 164.3 (d, *J* = 249.3 Hz), 148.3, 130.0, 129.6 (d, *J* = 8.3 Hz), 115.0 (d, *J* = 21.5 Hz), 103.9, 51.5, 41.9, 37.4, 31.1, 25.9, 22.1, 21.84, 21.77, 7.0.

^{19}F NMR (377 MHz, $CDCl_3$) δ -110.6.

IR (neat) ν_{max} (cm^{-1}) = 2989, 2941, 1721, 1622, 1612, 1551, 1432, 1398, 1327, 1286, 1236, 1213, 1143, 1128, 1072, 943, 921, 855, 825, 761, 692.

HRMS (ESI/QTOF) m/z: $[M + H]^+$ Calcd for $C_{19}H_{25}FNO_3^+$ 334.1813; Found 334.1817.

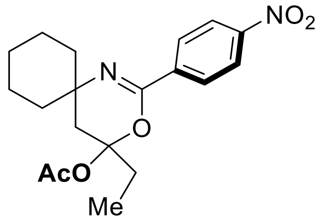

2c. Colorless oil (24.5 mg, 68% yield).

Analytical data for **2c**:

^{1}H NMR (500 MHz, $CDCl_3$) δ 8.24 – 8.20 (m, 2H), 8.19 – 8.15 (m, 2H), 2.81 (d, *J* = 14.9 Hz, 1H), 2.33 (dq, *J* = 14.9, 7.5 Hz, 1H), 2.18 (dq, *J* = 14.8, 7.5 Hz, 1H), 2.01 (s, 3H), 1.96 – 1.87 (m, 1H), 1.84 – 1.78 (m, 1H), 1.78 – 1.71 (m, 1H), 1.60 – 1.52 (m, 4H), 1.50 – 1.42 (m, 4H), 1.03 (t, *J* = 7.5 Hz, 3H).

^{13}C NMR (126 MHz, $CDCl_3$) δ 169.0, 149.0, 147.6, 139.6, 128.4, 123.2, 103.9, 52.1, 41.7, 37.5, 37.4, 31.0, 25.8, 22.0, 21.73, 21.69, 7.0.

IR (neat) ν_{max} (cm^{-1}) = 2973, 2833, 1742, 1659, 1632, 1597, 1421, 1378, 1289, 1184, 992, 843, 827, 811, 764, 682.

HRMS (Nanochip-based ESI/LTQ-Orbitrap) m/z: $[M + H]^+$ Calcd for $C_{19}H_{25}N_2O_5^+$ 361.1758; Found 361.1758.

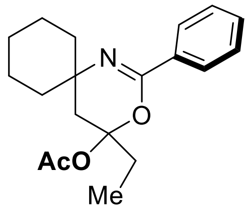

2d. Colorless oil (18.9 mg, 60% yield).

Analytical data for **2d**:

^{1}H NMR (600 MHz, $CDCl_3$) δ 8.01 (d, *J* = 7.5 Hz, 2H), 7.44 – 7.40 (m, 1H), 7.40 – 7.36 (m, 2H), 2.84 (d, *J* = 14.7 Hz, 1H), 2.34 (dq, *J* = 14.8, 7.5 Hz, 1H), 2.15 (dq, *J* = 14.7, 7.4 Hz, 1H), 2.01 (s, 3H), 1.95 – 1.87 (m, 1H), 1.83 – 1.72 (m, 2H), 1.62 – 1.49 (m, 4H), 1.49 – 1.40 (m, 4H), 1.03 (t, *J* = 7.5 Hz, 3H).

^{13}C NMR (151 MHz, $CDCl_3$) δ 169.2, 149.1, 133.8, 130.4, 128.0, 127.5, 103.8, 51.5, 42.0, 37.4, 37.3, 31.2, 25.9, 22.1, 21.9, 21.8, 6.9.

IR (neat) ν_{max} (cm^{-1}) = 2987, 2971, 1764, 1735, 1622, 1656, 1538, 1468, 1453, 1395, 1284, 1165, 1045, 1011, 954, 866, 828, 743, 722, 681.

HRMS (ESI/QTOF) m/z: $[M + H]^+$ Calcd for $C_{19}H_{26}NO_3^+$ 316.1907; Found 316.1916.

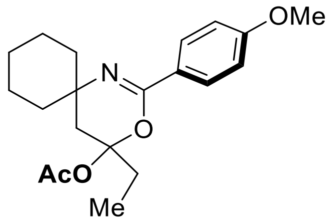

2e. Colorless oil (15.5 mg, 45% yield).

Analytical data for **2e**:

^{1}H NMR (500 MHz, $CDCl_3$) δ 7.95 (d, *J* = 8.4 Hz, 2H), 6.90 – 6.87 (m, 2H), 3.83 (s, 3H), 2.82 (d, *J* = 14.8 Hz, 1H), 2.33 (dq, *J* = 14.8, 7.5 Hz, 1H), 2.14 (dq, *J* = 14.7, 7.4 Hz, 1H), 2.00 (s, 3H), 1.95 – 1.85 (m, 1H), 1.82 – 1.69 (m, 2H), 1.59 – 1.38 (m, 8H), 1.02 (t, *J* = 7.4 Hz, 3H).

^{13}C NMR (126 MHz, $CDCl_3$) δ 169.2, 161.4, 148.8, 129.0, 126.4, 113.3, 103.8, 55.3, 51.3, 42.0, 37.4, 37.2, 31.2, 25.9, 22.1, 21.9, 21.8, 7.0.

IR (neat) ν_{max} (cm^{-1}) = 2989, 2943, 1751, 1683, 1552, 1458, 1371, 1277, 1243, 1213, 1191, 1093, 1071, 1036, 982, 944, 923, 861, 811, 778, 752, 687, 634.

HRMS (ESI/QTOF) m/z: $[M + H]^+$ Calcd for $C_{20}H_{28}NO_4^+$ 346.2013; Found 346.2017.

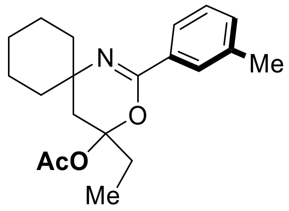

2f. Colorless oil (19.9 mg, 58% yield).

Analytical data for **2f**:

^{1}H NMR (500 MHz, $CDCl_3$) δ 7.82 (s, 1H), 7.80 (d, *J* = 7.7 Hz, 1H), 7.27 (t, *J* = 7.5 Hz, 1H), 7.23 (d, *J* = 7.6 Hz, 1H), 2.86 (d, *J* = 14.8 Hz, 1H), 2.39 (s, 3H), 2.34 (dq, *J* = 14.9, 7.8 Hz, 1H), 2.14 (dq, *J* = 14.7, 7.5 Hz, 1H), 2.01 (s, 3H), 1.94 – 1.86 (m, 1H), 1.82 – 1.70 (m, 2H), 1.54 (d, *J* = 14.8 Hz, 1H), 1.63-1.39 (m, 7H), 1.03 (t, *J* = 7.4 Hz, 3H).

^{13}C NMR (126 MHz, $CDCl_3$) δ 169.2, 149.3, 137.7, 133.7, 131.2, 127.97, 127.95, 124.6, 103.8, 51.5, 42.0, 37.2, 37.0, 31.2, 25.9, 22.1, 21.9, 21.8, 21.4, 7.0.

IR (neat) ν_{max} (cm^{-1}) = 2968, 2944, 2861, 1725, 1671, 1551, 1466, 1399, 1179, 1055, 1023, 993, 937, 883, 859, 791, 757, 691.

HRMS (ESI/QTOF) m/z: $[M + H]^+$ Calcd for $C_{20}H_{28}NO_3^+$ 330.2064; Found 330.2066.

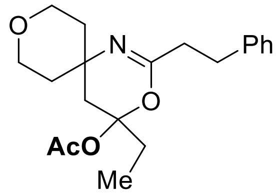

2g. Colorless oil (13.8 mg, 40% yield).

Analytical data for **2g**:

^{1}H NMR (600 MHz, $CDCl_3$) δ 7.27 – 7.22 (m, 4H), 7.19 – 7.15 (m, 1H), 4.03 (ddd, *J* = 12.4, 10.4, 2.7 Hz, 1H), 3.76 (ddd, *J* = 11.4, 10.0, 3.0 Hz, 1H), 3.64 (dt, *J* = 11.4, 4.2 Hz, 1H), 3.58 (dt, *J* = 11.5, 4.2 Hz, 1H), 2.94 (t, *J* = 7.6 Hz, 2H), 2.62 – 2.50 (m, 3H), 2.16 (dq, *J* = 14.9, 7.5 Hz, 1H), 2.02 (dq, *J* = 13.1, 6.6, 5.8 Hz, 1H), 1.98 (s, 3H), 1.64 – 1.54 (m, 4H), 1.40 (d, *J* = 14.7 Hz, 1H), 0.90 (t, *J* = 7.5 Hz,

3H).

^{13}C NMR (151 MHz, $CDCl_3$) δ 168.9, 153.2, 141.0, 128.5, 128.2, 125.9, 103.4, 63.4, 63.3, 48.2, 40.4, 38.5, 38.4, 36.0, 31.9, 30.9, 22.1, 6.8.

IR (neat) ν_{max} (cm^{-1}) = 2963, 2953, 2895, 1679, 1674, 1664, 1466, 1457, 1362, 1275, 1248, 1186, 1175, 1136, 1083, 1069, 1060, 1026, 778, 695.

HRMS (nanochip-ESI/LTQ-Orbitrap) m/z: $[M + H]^+$ Calcd for $C_{20}H_{28}NO_4^+$ 346.2013; Found 346.2019.

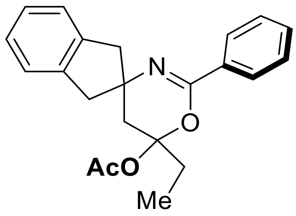

2h. Colorless oil (24.8 mg, 71% yield).

Analytical data for **2h**:

^{1}H NMR (500 MHz, $CDCl_3$) δ 7.97 – 7.94 (m, 2H), 7.43 – 7.38 (m, 1H), 7.36 – 7.32 (m, 2H), 7.27 – 7.24 (m, 1H), 7.23 – 7.16 (m, 3H), 3.16 – 3.04 (m, 4H), 2.88 (d, *J* = 14.4 Hz, 1H), 2.38 (dq, *J* = 15.0, 7.5 Hz, 1H), 2.28 (dq, *J* = 14.7, 7.5 Hz, 1H), 2.12 (s, 3H), 2.02 (d, *J* = 14.5 Hz, 1H), 1.07 (t, *J* = 7.5 Hz, 3H).

^{13}C NMR (126 MHz, $CDCl_3$) δ 169.2, 150.2, 142.2, 140.9, 133.3, 130.5, 128.0, 127.6, 126.41, 126.37, 124.5, 103.6, 60.2, 49.2, 47.1, 37.5, 30.4, 22.2, 7.1.

IR (neat) ν_{max} (cm^{-1}) = 2991, 2843, 1756, 1688, 1551, 1372, 1261, 1146, 1077, 1082, 931, 855, 834, 782, 744, 696.

HRMS (Sicrit plasma/LTQ-Orbitrap) m/z: $[M + H]^+$ Calcd for $C_{22}H_{24}NO_3^+$ 350.1751; Found 350.1748.

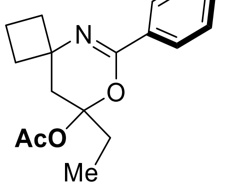

2i. Colorless oil (18.1 mg, 63% yield).

Analytical data for **2i**:

^{1}H NMR (800 MHz, $CDCl_3$) δ 8.01 (d, J = 7.5 Hz, 2H), 7.45 – 7.41 (m, 1H), 7.40 – 7.37 (m, 2H), 2.88 (d, J = 14.2 Hz, 1H), 2.32 (dq, J = 15.0, 7.5 Hz, 1H), 2.29 – 2.24 (m, 2H), 2.21 – 2.15 (m, 3H), 2.13 – 2.08 (m, 1H), 1.98 (s, 3H), 1.91 – 1.84 (m, 1H), 1.76 (d, J = 14.3 Hz, 1H), 1.03 (t, J = 7.5 Hz, 3H).

^{13}C NMR (201 MHz, $CDCl_3$) δ 169.3, 150.1, 133.4, 130.5, 128.1, 127.5, 104.0, 54.9, 37.1, 36.0, 35.6, 30.1, 21.9, 15.6, 6.9.

IR (neat) ν_{max} (cm^{-1}) = 2984, 2943, 2834, 1763, 1665, 1524, 1347, 1288, 1243, 1165, 1123, 1066, 988, 943, 827, 834, 766.

HRMS (ESI/QTOF) m/z: $[M + H]^+$ Calcd for $C_{17}H_{22}NO_3^+$ 288.1594; Found 288.1602.

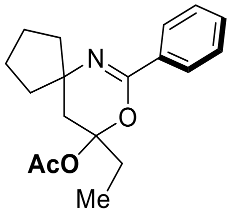

2j. Colorless oil (19.9 mg, 66% yield).

Analytical data for **2j**:

^{1}H NMR (600 MHz, $CDCl_3$) δ 7.98 (d, J = 7.5 Hz, 2H), 7.41 (t, J = 7.2 Hz, 1H), 7.37 (t, J = 7.7 Hz, 2H), 2.65 (d, J = 14.4 Hz, 1H), 2.35 (dq, J = 14.8, 7.5 Hz, 1H), 2.17 (dq, J = 14.8, 7.5 Hz, 1H), 2.07 – 1.95 (m, 2H), 2.02 (s, 3H), 1.91 (d, J = 14.4 Hz, 1H), 1.87 – 1.81 (m, 1H), 1.77 – 1.64 (m, 3H), 1.62 – 1.56 (m, 2H), 1.05 – 1.03 (m, 3H).

^{13}C NMR (151 MHz, $CDCl_3$) δ 169.4, 148.9, 133.8, 130.3, 128.0, 127.4, 103.8, 59.9, 42.9, 40.0, 37.3, 30.6, 24.7, 22.9, 22.1, 7.0.

IR (neat) ν_{max} (cm^{-1}) = 2977, 2854, 1766, 1692, 1638, 1592, 1455, 1387, 1248, 1213, 1177, 1082, 948, 837, 821, 755.

HRMS (ESI/QTOF) m/z: $[M + H]^+$ Calcd for $C_{18}H_{24}NO_3^+$ 302.1751; Found 302.1746.

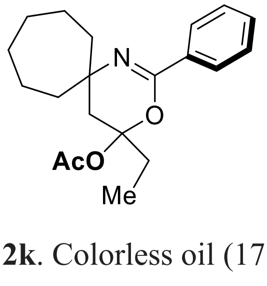

2k. Colorless oil (17.5 mg, 53% yield).

Analytical data for **2k**:

^{1}H NMR (400 MHz, $CDCl_3$) δ 8.01 – 7.97 (m, 2H), 7.44-7.34 (m, 3H), 2.80 (d, *J* = 14.8 Hz, 1H), 2.33 (dq, *J* = 14.9, 7.5 Hz, 1H), 2.16 (dq, *J* = 14.7, 7.4 Hz, 1H), 2.01 (s, 3H), 1.90 – 1.37 (m, 13H), 1.03 (t, *J* = 7.5 Hz, 3H).

^{13}C NMR (151 MHz, $CDCl_3$) δ 169.2, 148.7, 133.9, 130.3, 128.0, 127.5, 103.7, 54.8, 45.0, 39.9, 38.0, 31.2, 29.6, 29.5, 22.5, 22.14, 22.10, 7.0.

IR (neat) ν_{max} (cm^{-1}) = 2983, 2954, 2848, 1732, 1671, 1533, 1470, 1352, 1269, 1159, 1131, 1062, 989, 944, 829, 760, 684.

HRMS (ESI/QTOF) m/z: $[M + H]^+$ Calcd for $C_{20}H_{28}NO_3^+$ 330.2064; Found 330.2072.

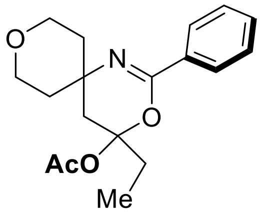

2l. Colorless oil (20.9 mg, 66% yield).

Analytical data for **2l**:

^{1}H NMR (500 MHz, $CDCl_3$) δ 8.07 – 8.03 (m, 2H), 7.47 – 7.42 (m, 1H), 7.41 – 7.38 (m, 2H), 4.25 (td, *J* = 11.1, 2.6 Hz, 1H), 4.00 (td, *J* = 11.2, 2.7 Hz, 1H), 3.77 (dt, *J* = 11.4, 3.9 Hz, 1H), 3.72 (dt, *J* = 11.4, 3.9 Hz, 1H), 2.71 (d, *J* = 14.7 Hz, 1H), 2.33 (dq, *J* = 15.0, 7.5 Hz, 1H), 2.20 (dq, *J* = 14.8, 7.5 Hz, 1H), 2.00 (s, 3H), 1.87 (dq, *J* = 13.9, 2.8 Hz, 1H), 1.81 – 1.71 (m, 2H), 1.61 (d, *J* = 14.7 Hz, 1H), 1.43 – 1.36 (m, 1H), 1.04 (t, *J* = 7.5 Hz, 3H).

^{13}C NMR (126 MHz, $CDCl_3$) δ 169.0, 149.4, 133.4, 130.7, 128.1, 127.5, 103.7, 63.6, 63.5, 48.8, 40.4, 39.5, 39.0, 30.9, 22.1, 7.0.

IR (neat) ν_{max} (cm^{-1}) = 2983, 2916, 2833, 2819, 1734, 1652, 1552, 1493, 1371, 1268, 1141, 1099, 1056, 1026, 952, 847, 838, 794, 776, 696, 622.

HRMS (Sicrit plasma/LTQ-Orbitrap) m/z: $[M + H]^+$ Calcd for $C_{18}H_{24}NO_4^+$ 318.1700; Found 318.1699.

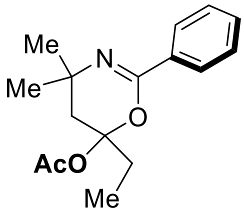

2m. Colorless oil (17.1 mg, 62% yield).

Analytical data for **2m**:

^{1}H NMR (600 MHz, $CDCl_3$) δ 7.98 (d, *J* = 7.6 Hz, 2H), 7.45 – 7.41 (m, 1H), 7.40 – 7.35 (m, 2H), 2.69 (d, *J* = 14.6 Hz, 1H), 2.36 – 2.29 (m, 1H), 2.18 (dq, *J* = 14.7, 7.4 Hz, 1H), 2.03 (s, 3H), 1.69 (d, *J* = 14.6 Hz, 1H), 1.34 (s, 3H), 1.30 (s, 3H), 1.03 (t, *J* = 7.5 Hz, 3H).

^{13}C NMR (151 MHz, $CDCl_3$) δ 169.2, 150.1, 133.7, 130.5, 128.1, 127.5, 103.7, 49.4, 38.9, 33.3, 31.0, 29.1, 22.1, 6.9.

IR (neat) ν_{max} (cm^{-1}) = 2983, 2953, 1722, 1644, 1567, 1433, 1371, 1255, 1044, 1026, 955, 887, 832, 782, 733, 692.

HRMS (ESI/QTOF) m/z: $[M + H]^+$ Calcd for $C_{16}H_{22}NO_3^+$ 276.1594; Found 276.1594.

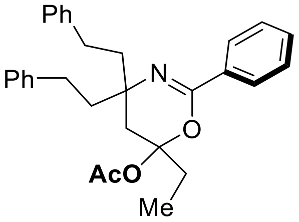

2n. Colorless oil (30.5 mg, 67% yield).

Analytical data for **2n**:

^{1}H NMR (500 MHz, $CDCl_3$) δ 8.06 – 8.04 (m, 2H), 7.48 – 7.44 (m, 1H), 7.43 – 7.39 (m, 2H), 7.31 (t, *J* = 7.5 Hz, 2H), 7.29 – 7.23 (m, 4H), 7.22 – 7.15 (m, 4H), 2.86 – 2.79 (m, 3H), 2.73 – 2.65 (m, 2H), 2.38 (dq, *J* = 14.9, 7.5 Hz, 1H), 2.23 (dq, *J* = 14.8, 7.4 Hz, 1H), 2.14 (td, *J* = 13.3, 4.9 Hz, 1H), 2.01 (td, *J* = 12.8, 12.4, 5.0 Hz, 1H), 1.95 – 1.86 (m, 2H), 1.93 (s, 3H), 1.80 (d, *J* = 14.9 Hz, 1H), 1.06 (t, *J* = 7.5 Hz, 3H).

^{13}C NMR (126 MHz, $CDCl_3$) δ 169.2, 150.1, 142.5, 142.4, 133.6, 130.6, 128.42, 128.35, 128.2, 128.1, 127.5, 125.8, 125.7, 103.5, 54.1, 43.2, 41.1, 34.4, 31.1, 29.90, 29.88, 22.2, 7.0.

IR (neat) ν_{max} (cm^{-1}) = 2981, 2864, 1721, 1659, 1537, 1466, 1321, 1278, 1163, 1142, 1116, 1086, 952, 948, 933, 891, 872, 833, 749, 689.

HRMS (Sicrit plasma/LTQ-Orbitrap) m/z: $[M + H]^+$ Calcd for $C_{30}H_{34}NO_3^+$ 456.2533; Found 456.2532.

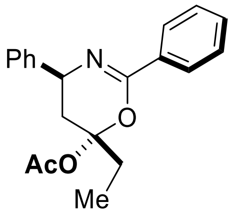

2o. Colorless oil (17.5 mg, 54% yield, 8:1 dr).

Analytical data for **2o**:

^{1}H NMR (600 MHz, $CDCl_3$) δ 8.10 (d, J = 7.5 Hz, 2H), 7.50 – 7.45 (m, 1H), 7.44 – 7.40 (m, 4H), 7.38 (t, J = 7.5 Hz, 2H), 7.31 – 7.27 (m, 1H), 4.74 (dd, J = 12.5, 5.0 Hz, 1H), 2.89 (dd, J = 14.0, 4.9 Hz, 1H), 2.34 (d, J = 7.5 Hz, 1H), 2.32 (d, J = 7.5 Hz, 1H), 2.12 (s, 3H), 1.66 (t, J = 13.2 Hz, 1H), 1.05 (t, J = 7.5 Hz, 3H).

^{13}C NMR (151 MHz, $CDCl_3$) δ 168.9, 153.1, 143.5, 133.1, 130.9, 128.5, 128.1, 127.5, 126.9, 126.6, 103.7, 52.6, 36.6, 29.9, 22.1, 7.2.

IR (neat) ν_{max} (cm^{-1}) = 2953, 2877, 1680, 1499, 1385, 1276, 1173, 1036, 1012, 954, 864, 772, 686, 644, 629.

HRMS (ESI/QTOF) m/z: $[M + H]^+$ Calcd for $C_{20}H_{22}NO_3^+$ 324.1594; Found 324.1596.

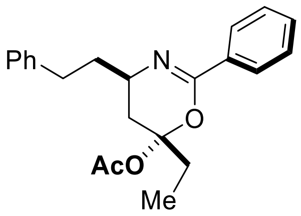

2p. Colorless oil (19.7 mg, 56% yield, 9:1 dr).

Analytical data for **2p**:

^{1}H NMR (600 MHz, $CDCl_3$) δ 8.01 (d, J = 7.6 Hz, 2H), 7.44 (t, J = 7.3 Hz, 1H), 7.39 (t, J = 7.5 Hz, 2H), 7.30 (t, J = 7.4 Hz, 2H), 7.26 (d, J = 7.7 Hz, 2H), 7.21 – 7.17 (m, 1H), 3.53 (dq, J = 12.3, 6.1 Hz, 1H), 2.98 (ddd, J = 15.3, 9.7, 6.3 Hz, 1H), 2.85 (ddd, J = 14.4, 9.7, 6.1 Hz, 1H), 2.68 (dd, J = 13.8, 4.7 Hz, 1H), 2.34 (dq, J = 14.9, 7.5 Hz, 1H), 2.26 (dq, J = 14.4, 7.4 Hz, 1H), 2.00 (s, 3H), 1.97 – 1.85 (m, 2H), 1.48 (t, J = 13.0 Hz, 1H), 1.05 (t, J = 7.5 Hz, 3H).

^{13}C NMR (151 MHz, $CDCl_3$) δ 168.9, 151.8, 142.3, 133.2, 130.6, 128.6, 128.3, 128.1, 127.3, 125.7,

104.0, 48.4, 38.4, 33.5, 32.1, 30.0, 21.9, 7.1.

IR (neat) ν_{max} (cm^{-1}) = 2972, 2855, 1669, 1480, 1464, 1379, 1348, 1281, 1155, 1031, 794, 692.

HRMS (ESI/QTOF) m/z: $[M + Na]^+$ Calcd for $C_{22}H_{25}NNaO_3^+$ 374.1727; Found 374.1734.

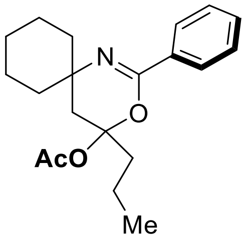

2q. Colorless oil (18.4 mg, 56% yield).

Analytical data for **2q**:

^{1}H NMR (500 MHz, $CDCl_3$) δ 8.02 – 7.99 (m, 2H), 7.44 – 7.40 (m, 1H), 7.40 – 7.36 (m, 2H), 2.85 (d, *J* = 14.8 Hz, 1H), 2.29 (ddd, *J* = 14.1, 11.7, 4.9 Hz, 1H), 2.09 (ddd, *J* = 14.1, 11.7, 4.8 Hz, 1H), 2.00 (s, 3H), 1.95 – 1.86 (m, 1H), 1.83 – 1.70 (m, 2H), 1.66 – 1.39 (m, 10H), 0.99 (t, *J* = 7.4 Hz, 3H).

^{13}C NMR (126 MHz, $CDCl_3$) δ 169.3, 149.1, 133.8, 130.4, 128.0, 127.5, 103.6, 51.5, 42.0, 40.3, 37.6, 37.4, 25.9, 22.2, 21.84, 21.76, 16.0, 14.0.

IR (neat) ν_{max} (cm^{-1}) = 2982, 2911, 2902, 2861, 1713, 1692, 1577, 1427, 1345, 1211, 1191, 1077, 1034, 992, 951, 897, 863, 781, 752, 695, 631.

HRMS (ESI/QTOF) m/z: $[M + H]^+$ Calcd for $C_{20}H_{28}NO_3^+$ 330.2064; Found 330.2063.

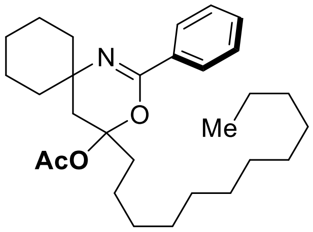

2r. Colorless oil (24.1 mg, 53% yield).

Analytical data for **2r**:

^{1}H NMR (500 MHz, $CDCl_3$) δ 8.02 – 8.00 (m, 2H), 7.45 – 7.40 (m, 1H), 7.40 – 7.36 (m, 2H), 2.84 (d, *J* = 14.7 Hz, 1H), 2.29 (ddd, *J* = 14.2, 11.7, 3.9 Hz, 1H), 2.14 – 2.08 (m, 1H), 2.00 (s, 3H), 1.95 – 1.86 (m, 1H), 1.83 – 1.70 (m, 2H), 1.60 – 1.39 (m, 10H), 1.38 – 1.23 (m, 18H), 0.88 (t, *J* = 6.9 Hz, 3H).

^{13}C NMR (126 MHz, $CDCl_3$) δ 169.2, 149.1, 133.8, 130.4, 128.0, 127.5, 103.7, 51.5, 42.0, 38.1, 37.6, 37.4, 31.9, 29.7, 29.64, 29.62, 29.56, 29.50, 29.47, 29.3, 25.9, 22.7, 22.6, 22.2, 21.9, 21.8, 14.1.

IR (neat) ν_{max} (cm^{-1}) = 2965, 2941, 2877, 1734, 1635, 1569, 1451, 1322, 1251, 1177, 1154, 1099, 955, 833, 762, 716, 697, 634.

HRMS (ESI/QTOF) m/z: $[M + H]^+$ Calcd for $C_{29}H_{46}NO_3^+$ 456.3472; Found 456.3481.

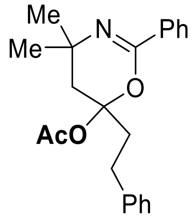

2s. Colorless oil (15.8 mg, 45% yield).

Analytical data for **2s**:

^{1}H NMR (600 MHz, $CDCl_3$) δ 7.96 (d, *J* = 7.6 Hz, 2H), 7.44 (t, *J* = 7.3 Hz, 1H), 7.39 (t, *J* = 7.5 Hz, 2H), 7.30 (t, *J* = 7.5 Hz, 2H), 7.23 (d, *J* = 7.5 Hz, 2H), 7.21 (t, *J* = 7.6 Hz, 1H), 2.91 (ddd, *J* = 13.5, 11.0, 5.5 Hz, 1H), 2.81 – 2.74 (m, 2H), 2.70 (ddd, *J* = 14.2, 11.0, 5.4 Hz, 1H), 2.42 (ddd, *J* = 15.2, 10.9, 5.4 Hz, 1H), 1.98 (s, 3H), 1.74 (d, *J* = 14.6 Hz, 1H), 1.34 (s, 3H), 1.30 (s, 3H).

^{13}C NMR (151 MHz, $CDCl_3$) δ 169.3, 149.9, 141.0, 133.6, 130.6, 130.2, 128.5, 128.1, 127.5, 126.0, 103.0, 49.4, 39.5, 39.3, 33.3, 29.1, 29.0, 22.0.

IR (neat) ν_{max} (cm^{-1}) = 2939, 2882, 1726, 1672, 1570, 1439, 1331, 1262, 1173, 1083, 944, 917, 881, 699, 644.

HRMS (Nanochip-based ESI/LTQ-Orbitrap) m/z: $[M + H]^+$ Calcd for $C_{22}H_{26}NO_3^+$ 352.1907; Found 352.1907.

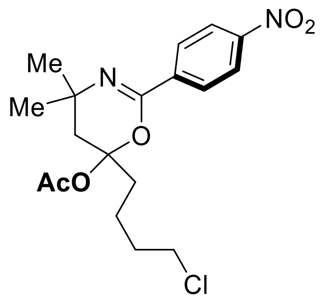

2t. Colorless oil (21.4 mg, 56% yield).

Analytical data for **2t**:

^{1}H NMR (600 MHz, $CDCl_3$) δ 8.24 – 8.20 (m, 2H), 8.16 – 8.12 (m, 2H), 3.59 (t, *J* = 6.4 Hz, 2H), 2.70 (d, *J* = 14.7 Hz, 1H), 2.32 (ddd, *J* = 14.1, 11.6, 5.0 Hz, 1H), 2.20 (ddd, *J* = 14.1, 11.6, 5.0 Hz, 1H),

2.04 (s, 3H), 1.89 – 1.82 (m, 2H), 1.76 – 1.67 (m, 2H), 1.63 – 1.57 (m, 1H), 1.34 (s, 3H), 1.31 (s, 3H).

^{13}C NMR (151 MHz, $CDCl_3$) δ 169.1, 149.1, 148.4, 139.3, 128.5, 123.3, 103.2, 50.0, 44.6, 39.2, 36.9, 33.1, 32.0, 29.0, 22.0, 19.9.

IR (neat) ν_{max} (cm^{-1}) = 2956, 2837, 2814, 1744, 1666, 1632, 1553, 1525, 1499, 1428, 1335, 1302, 1299, 1156, 1098, 1036, 994, 926, 877, 724, 719, 692, 672.

HRMS (ESI/QTOF) m/z: $[M + H]^+$ Calcd for $C_{18}H_{24}ClN_2O_5^+$ 383.1368; Found 383.1359.

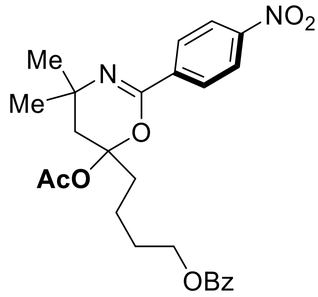

2u. Colorless oil (27.2 mg, 58% yield).

Analytical data for **2u**:

^{1}H NMR (600 MHz, $CDCl_3$) δ 8.14 (d, *J* = 8.7 Hz, 2H), 8.10 (d, *J* = 8.6 Hz, 2H), 8.04 (d, *J* = 8.1 Hz, 2H), 7.59 – 7.56 (m, 1H), 7.44 (t, *J* = 7.6 Hz, 2H), 4.38 (t, *J* = 6.4 Hz, 2H), 2.72 (d, *J* = 14.7 Hz, 1H), 2.40 (ddd, *J* = 13.8, 11.7, 4.9 Hz, 1H), 2.23 (ddd, *J* = 14.0, 11.8, 4.8 Hz, 1H), 2.01 (s, 3H), 1.86 (p, *J* = 7.0 Hz, 2H), 1.77 – 1.69 (m, 2H), 1.61 – 1.54 (m, 1H), 1.33 (s, 3H), 1.30 (s, 3H).

^{13}C NMR (151 MHz, $CDCl_3$) δ 169.0, 166.5, 149.1, 148.4, 139.3, 133.0, 130.3, 129.5, 128.41, 128.39, 123.2, 103.3, 64.3, 49.9, 39.1, 37.3, 33.0, 29.0, 28.4, 22.0, 19.1.

IR (neat) ν_{max} (cm^{-1}) = 2965, 2910, 2849, 1733, 1678, 1662, 1644, 1520, 1501, 1416, 1326, 1223, 1212, 1155, 1077, 998, 922, 872, 757, 669, 633.

HRMS (ESI/QTOF) m/z: $[M + H]^+$ Calcd for $C_{25}H_{29}N_2O_7^+$ 469.1969; Found 469.1968.

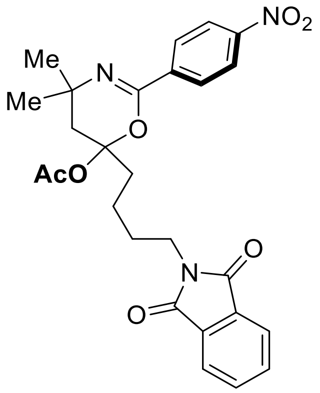

2v. Colorless oil (27.6 mg, 56% yield).

Analytical data for **2v**:

¹H NMR (600 MHz, $CDCl_3$) δ 8.16 (d, *J* = 8.5 Hz, 2H), 8.09 (d, *J* = 8.6 Hz, 2H), 7.85 – 7.82 (m, 2H), 7.75 – 7.70 (m, 2H), 3.72 (t, *J* = 7.2 Hz, 2H), 2.69 (d, *J* = 14.6 Hz, 1H), 2.35 (ddd, *J* = 14.0, 11.6, 4.9 Hz, 1H), 2.20 (ddd, *J* = 14.0, 11.6, 4.8 Hz, 1H), 2.01 (s, 3H), 1.76 (p, *J* = 7.5 Hz, 2H), 1.70 (d, *J* = 14.6 Hz, 1H), 1.63 – 1.56 (m, 1H), 1.52 – 1.43 (m, 1H), 1.32 (s, 3H), 1.29 (s, 3H).

¹³C NMR (151 MHz, $CDCl_3$) δ 169.0, 168.4, 149.1, 148.5, 139.3, 134.0, 132.0, 128.5, 123.21, 123.19, 103.2, 49.9, 39.1, 37.6, 37.3, 33.0, 29.0, 28.3, 22.0, 19.9.

IR (neat) ν_{max} (cm^{-1}) = 2972, 2888, 2843, 1711, 1655, 1545, 1442, 1427, 1311, 1259, 1195, 1164, 1062, 933, 912, 888, 834, 716, 690, 642.

HRMS (ESI/QTOF) m/z: $[M + H]^+$ Calcd for $C_{26}H_{28}N_3O_7^+$ 494.1922; Found 494.1919.

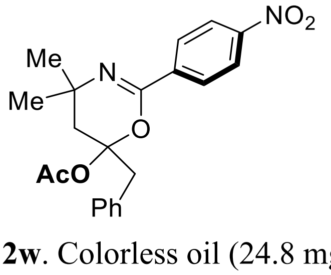

2w. Colorless oil (24.8 mg, 65% yield).

Analytical data for **2w**:

¹H NMR (500 MHz, $CDCl_3$) δ 8.24 – 8.21 (m, 2H), 8.16 – 8.12 (m, 2H), 7.36 – 7.32 (m, 2H), 7.33 – 7.27 (m, 3H), 3.60 (d, *J* = 14.1 Hz, 1H), 3.50 (d, *J* = 14.1 Hz, 1H), 2.75 (d, *J* = 14.7 Hz, 1H), 1.98 (s, 3H), 1.75 (d, *J* = 14.7 Hz, 1H), 1.32 (s, 3H), 1.28 (s, 3H).

¹³C NMR (126 MHz, $CDCl_3$) δ 169.5, 149.1, 148.4, 139.3, 134.2, 130.6, 128.5, 128.4, 127.3, 123.3, 102.7, 50.0, 43.6, 38.8, 33.0, 28.9, 22.2.

IR (neat) ν_{max} (cm^{-1}) = 2915, 2886, 1781, 1640, 1633, 1538, 1527, 1522, 1490, 1452, 1290. 1173, 1145, 1019, 1007, 997, 986, 889, 716, 711, 683, 666.

HRMS (ESI/QTOF) m/z: $[M + H]^+$ Calcd for $C_{21}H_{23}N_2O_5^+$ 383.1601; Found 383.1611.

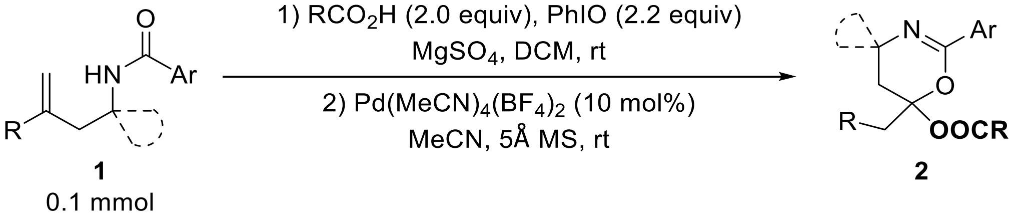

At room temperature, to a 10-mL test tube charged with a magnetic stirring bar were added the carboxylic acid (0.2 mmol, 2.0 equiv), iodosylbenzene (48.4 mg, 0.22 mmol, 2.2 equiv), magnesium sulfate (100 mg) and anhydrous DCM (2.0 mL). The reaction mixture was allowed to stir at room temperature. After the reaction was complete, DCM (2 mL) was added and the reaction mixture was filtered through a short pad of silica gel with diethyl ether. The filtrate was concentrated. The product was directly used for the next step.[5]

At room temperature, to a 10-mL test tube charged with a magnetic stirring bar were added substrate **1** (0.1 mmol, 1.0 equiv), the above hypervalent iodine compound, $[Pd(MeCN)_4(BF_4)_2]$ (4.5 mg, 0.01 mmol, 10 mol%), 5Å molecular sieves (50 mg) and anhydrous MeCN (1.0 mL). The reaction mixture was allowed to stir at room temperature. After the reaction was complete (monitored by TLC), diethyl ether (2 mL) was added and the reaction mixture was filtered through a short pad of silica gel with diethyl ether. The filtrate was concentrated. The residue was purified by silica gel flash column chromatography to give the desired product **2**.

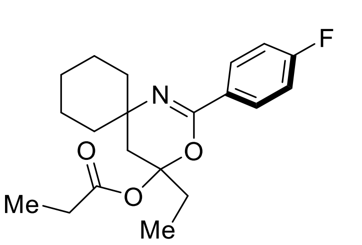

2x. Colorless oil (20.8 mg, 60% yield).

Analytical data for **2x**:

^{1}H NMR (500 MHz, $CDCl_3$) δ 8.00 (dd, *J* = 8.7, 5.7 Hz, 2H), 7.07 – 7.02 (m, 2H), 2.83 (d, *J* = 14.8

Hz, 1H), 2.37 – 2.22 (m, 3H), 2.16 (dq, J = 14.7, 7.4 Hz, 1H), 1.93 – 1.85 (m, 1H), 1.80 – 1.68 (m, 2H), 1.61 – 1.38 (m, 8H), 1.09 (t, J = 7.5 Hz, 3H), 1.01 (t, J = 7.5 Hz, 3H).

^{13}C NMR (126 MHz, $CDCl_3$) δ 172.6, 164.3 (d, J = 249.6 Hz), 148.4, 130.0, 129.6 (d, J = 8.4 Hz), 114.9 (d, J = 21.7 Hz), 103.7, 51.5, 41.9, 37.3, 31.2, 28.5, 25.9, 21.8, 21.8, 8.9, 7.0.

^{19}F NMR (377 MHz, $CDCl_3$) δ -110.6.

IR (neat) ν_{max} (cm^{-1}) = 2975, 2851, 1726, 1643, 1618, 1569, 1442, 1377, 1275, 1035, 977, 892, 867, 774, 693, 655.

HRMS (APCI/QTOF) m/z: $[M + H]^+$ Calcd for $C_{20}H_{27}FNO_3^+$ 348.1969; Found 348.1957.

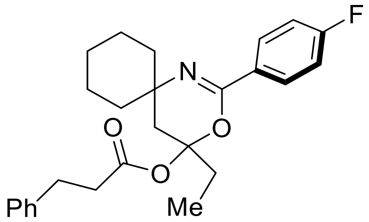

2y. Colorless oil (24.5 mg, 58% yield).

Analytical data for **2y**:

^{1}H NMR (600 MHz, $CDCl_3$) δ 8.02 – 7.96 (m, 2H), 7.23 (t, J = 7.4 Hz, 2H), 7.19 – 7.14 (m, 3H), 7.05 (t, J = 8.6 Hz, 2H), 2.89 (t, J = 7.7 Hz, 2H), 2.76 (d, J = 14.7 Hz, 1H), 2.66 – 2.53 (m, 2H), 2.28 (dq, J = 14.9, 7.5 Hz, 1H), 2.15 (dq, J = 14.7, 7.5 Hz, 1H), 1.92 – 1.86 (m, 1H), 1.73 – 1.63 (m, 2H), 1.56 – 1.48 (m, 3H), 1.48 – 1.39 (m, 4H), 1.39 – 1.34 (m, 1H), 0.94 (t, J = 7.5 Hz, 3H).

^{13}C NMR (151 MHz, $CDCl_3$) δ 170.9, 164.3 (d, J = 248.8 Hz), 148.3, 140.2, 130.0, 129.6 (d, J = 8.7 Hz), 128.4, 128.3, 126.3, 114.9 (d, J = 21.4 Hz), 104.0, 51.5, 41.9, 37.5, 37.4, 36.6, 31.1, 30.6, 25.9, 21.8, 21.7, 6.9.

^{19}F NMR (377 MHz, $CDCl_3$) δ -110.6.

IR (neat) ν_{max} (cm^{-1}) = 2967, 2933, 2827, 1754, 1661, 1475, 1176, 1081, 991, 943, 875, 864, 748, 751, 688.

HRMS (APCI/QTOF) m/z: $[M + H]^+$ Calcd for $C_{26}H_{31}FNO_3^+$ 424.2282; Found 424.2302.

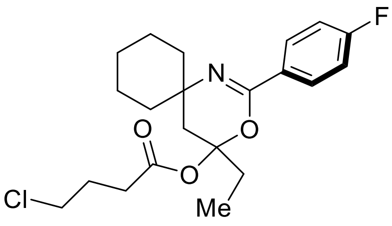

2z. Colorless oil (22.1 mg, 56% yield).

Analytical data for **2z**:

^{1}H NMR (500 MHz, $CDCl_3$) δ 8.00 (dd, J = 8.5, 5.6 Hz, 2H), 7.05 (t, J = 8.7 Hz, 2H), 3.56 (t, J = 6.3 Hz, 2H), 2.81 (d, J = 14.8 Hz, 1H), 2.46 (t, J = 7.2 Hz, 2H), 2.31 (dq, J = 14.8, 7.4 Hz, 1H), 2.16 (dq, J = 14.8, 7.4 Hz, 1H), 2.09 – 1.98 (m, 2H), 1.93 – 1.86 (m, 1H), 1.80 – 1.69 (m, 2H), 1.59 – 1.38 (m, 7H), 1.02 (t, J = 7.4 Hz, 3H), 0.91 – 0.82 (m, 1H).

^{13}C NMR (126 MHz, $CDCl_3$) δ 170.8, 164.3 (d, J = 249.7 Hz), 148.2, 129.9, 129.6 (d, J = 9.0 Hz), 115.0 (d, J = 21.7 Hz), 104.1, 51.5, 43.8, 41.9, 37.5, 31.9, 31.2, 27.3, 25.8, 21.8, 21.7, 7.0.

^{19}F NMR (377 MHz, $CDCl_3$) δ -110.5.

IR (neat) ν_{max} (cm^{-1}) = 2951, 1719, 1655, 1628, 1546, 1467, 1383, 1215, 1137, 964, 861, 705, 622.

HRMS (APCI/QTOF) m/z: $[M + H]^+$ Calcd for $C_{21}H_{28}ClFNO_3^+$ 396.1736; Found 396.1730.

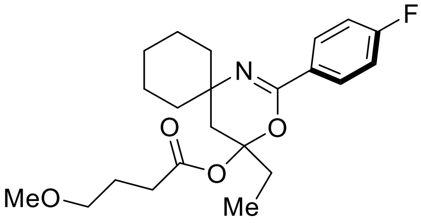

2aa. Colorless oil (20.7 mg, 53% yield).

Analytical data for **2aa**:

^{1}H NMR (600 MHz, $CDCl_3$) δ 8.01 – 7.98 (m, 2H), 7.05 (t, J = 8.7 Hz, 2H), 3.35 (t, J = 6.2 Hz, 2H), 3.27 (s, 3H), 2.80 (d, J = 14.7 Hz, 1H), 2.39 – 2.27 (m, 3H), 2.17 (dq, J = 14.7, 7.4 Hz, 1H), 1.93 – 1.87 (m, 1H), 1.86 – 1.80 (m, 2H), 1.80 – 1.69 (m, 2H), 1.58 – 1.39 (m, 8H), 1.01 (t, J = 7.5 Hz, 3H).

^{13}C NMR (151 MHz, $CDCl_3$) δ 171.6, 164.3 (d, J = 250.2 Hz), 148.3, 130.0, 129.6 (d, J = 8.7 Hz), 114.9 (d, J = 21.5 Hz), 103.8, 71.4, 58.5, 51.5, 41.9, 37.5, 37.4, 31.7, 31.2, 25.9, 24.6, 21.84, 21.78, 7.0.

^{19}F NMR (377 MHz, $CDCl_3$) δ -110.6.

IR (neat) ν_{max} (cm^{-1}) = 2963, 2847, 1732, 1699, 1530, 1463, 1388, 1395, 1173, 1122, 1073, 937, 884, 821, 747, 693, 651.

HRMS (APCI/QTOF) m/z: $[M + H]^+$ Calcd for $C_{22}H_{31}FNO_4^+$ 392.2232; Found 392.2244.

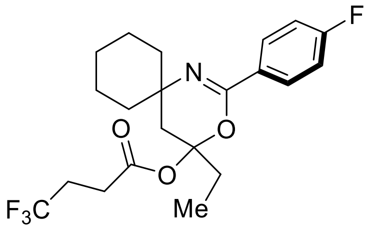

2ab. Colorless oil (17.4 mg, 42% yield).

Analytical data for **2ab**:

^{1}H NMR (600 MHz, $CDCl_3$) δ 8.00 (t, *J* = 7.2 Hz, 2H), 7.05 (t, *J* = 8.6 Hz, 2H), 2.76 (d, *J* = 14.8 Hz, 1H), 2.59 – 2.47 (m, 2H), 2.45 – 2.36 (m, 2H), 2.30 (dq, *J* = 14.7, 7.4 Hz, 1H), 2.19 (dq, *J* = 14.7, 7.5 Hz, 1H), 1.94 – 1.87 (m, 1H), 1.80 – 1.71 (m, 2H), 1.60 – 1.39 (m, 8H), 1.02 (t, *J* = 7.5 Hz, 3H).

^{13}C NMR (151 MHz, $CDCl_3$) δ 169.0, 164.4 (d, *J* = 250.7 Hz), 148.1, 129.7, 129.6 (d, *J* = 8.0 Hz), 126.4 (q, *J* = 276.1 Hz), 115.0 (d, *J* = 21.5 Hz), 104.6, 51.5, 41.8, 37.6, 37.5, 31.2, 29.0 (q, *J* = 30.2 Hz), 27.9 (q, *J* = 3.3 Hz), 25.8, 21.8, 21.7, 7.0.

^{19}F NMR (377 MHz, $CDCl_3$) δ -66.9, -110.4.

IR (neat) ν_{max} (cm^{-1}) = 2953, 2873, 1689, 1544, 1471, 1399, 1216, 1153, 1071, 973, 926, 836, 812, 759, 752, 686.

HRMS (APCI/QTOF) m/z: $[M + H]^+$ Calcd for $C_{21}H_{26}F_4NO_3^+$ 416.1843; Found 416.1851.

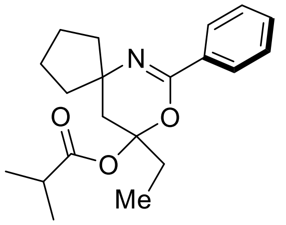

2ac. Colorless oil (17.1 mg, 52% yield).

Analytical data for **2ac**:

^{1}H NMR (600 MHz, $CDCl_3$) δ 7.97 (d, *J* = 7.1 Hz, 2H), 7.44 – 7.39 (m, 1H), 7.39 – 7.35 (m, 2H), 2.67 (d, *J* = 14.4 Hz, 1H), 2.50 (hept, *J* = 7.0 Hz, 1H), 2.32 (dq, *J* = 14.9, 7.5 Hz, 1H), 2.18 (dq, *J* = 14.6, 7.5 Hz, 1H), 2.08 – 1.93 (m, 2H), 1.91 (d, *J* = 14.4 Hz, 1H), 1.86 – 1.82 (m, 1H), 1.77 – 1.65 (m, 3H),

1.63 – 1.55 (m, 2H), 1.16 – 1.13 (m, 6H), 1.02 (t, J = 7.5 Hz, 3H).

^{13}C NMR (151 MHz, $CDCl_3$) δ 175.5, 149.1, 133.9, 130.3, 128.0, 127.4, 103.6, 59.9, 43.0, 40.2, 37.8, 34.8, 30.6, 24.7, 23.0, 19.2, 18.7, 6.8.

IR (neat) ν_{max} (cm^{-1}) = 2963, 2865, 1739, 1655, 1548, 1472, 1439, 1265, 1169, 1071, 971, 958, 939, 881, 851, 779, 755, 718, 681.

HRMS (ESI/QTOF) m/z: $[M + H]^+$ Calcd for $C_{20}H_{28}NO_3^+$ 330.2064; Found 330.2067.

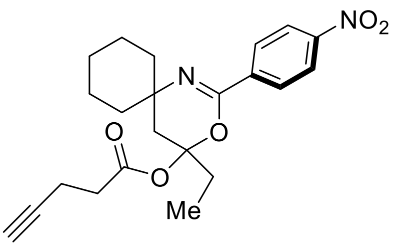

2ad. Colorless oil (21.59 mg, 54% yield).

Analytical data for **2ad**:

^{1}H NMR (600 MHz, $CDCl_3$) δ 8.23 – 8.20 (m, 2H), 8.18 – 8.15 (m, 2H), 2.80 (d, J = 14.8 Hz, 1H), 2.56 – 2.48 (m, 2H), 2.47 – 2.43 (m, 2H), 2.32 (dq, J = 14.9, 7.5 Hz, 1H), 2.21 (dq, J = 14.7, 7.4 Hz, 1H), 1.96 – 1.87 (m, 1H), 1.93 (t, J = 2.5 Hz, 1H), 1.80 (ddd, J = 12.4, 7.3, 3.5 Hz, 1H), 1.77 – 1.71 (m, 1H), 1.58 (d, J = 14.8 Hz, 1H), 1.57 – 1.52 (m, 3H), 1.50 – 1.42 (m, 4H), 1.04 (t, J = 7.5 Hz, 3H).

^{13}C NMR (151 MHz, $CDCl_3$) δ 169.7, 149.1, 147.5, 139.5, 128.4, 123.2, 104.3, 82.1, 69.2, 52.2, 41.6, 37.7, 37.5, 34.1, 31.1, 25.8, 21.73, 21.70, 14.1, 7.0.

IR (neat) ν_{max} (cm^{-1}) = 2955, 2933, 2842, 2245, 1718, 1673, 1562, 1433, 1321, 1277, 1184, 1041, 954, 923, 881, 862, 827, 783, 769, 651.

HRMS (ESI/QTOF) m/z: $[M + H]^+$ Calcd for $C_{22}H_{27}N_2O_5^+$ 399.1914; Found 399.1911.

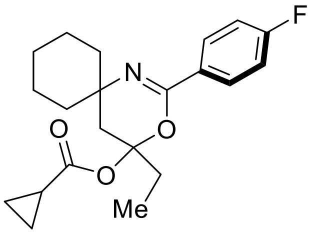

2ae. Colorless oil (21.6 mg, 60% yield).

Analytical data for **2ae**:

^{1}H NMR (500 MHz, $CDCl_3$) δ 8.02 – 7.99 (m, 2H), 7.05 (t, J = 8.7 Hz, 2H), 2.87 (d, J = 14.8 Hz, 1H),

2.32 (dq, J = 14.9, 7.5 Hz, 1H), 2.12 (dq, J = 14.7, 7.4 Hz, 1H), 1.91 – 1.83 (m, 1H), 1.82 – 1.68 (m, 2H), 1.63 – 1.40 (m, 9H), 1.03 (t, J = 7.5 Hz, 3H), 0.98 – 0.94 (m, 1H), 0.94 – 0.88 (m, 1H), 0.85 – 0.77 (m, 2H).

^{13}C NMR (126 MHz, $CDCl_3$) δ 173.0, 164.3 (d, J = 249.5 Hz), 148.4, 130.0, 129.6 (d, J = 8.6 Hz), 114.9 (d, J = 21.6 Hz), 103.8, 51.6, 42.1, 37.1, 31.3, 25.9, 21.9, 21.8, 13.5, 8.6, 8.2, 6.9.

^{19}F NMR (377 MHz, $CDCl_3$) δ -110.7.

IR (neat) ν_{max} (cm^{-1}) = 2982, 2864, 1731, 1689, 1661, 1570, 1557, 1487, 1372, 1365, 1275, 1153, 1077, 957, 877, 792, 748, 696.

HRMS (ESI/QTOF) m/z: $[M + H]^+$ Calcd for $C_{21}H_{27}FNO_3^+$ 360.1969; Found 360.1974.

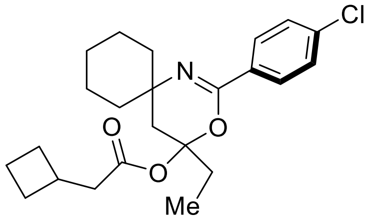

2af. Colorless oil (22.6 mg, 56% yield).

Analytical data for **2af**:

^{1}H NMR (600 MHz, $CDCl_3$) δ 7.93 (d, J = 8.3 Hz, 2H), 7.35 – 7.33 (m, 2H), 2.81 (d, J = 14.7 Hz, 1H), 2.63 (hept, J = 7.9 Hz, 1H), 2.40 – 2.32 (m, 2H), 2.31 – 2.26 (m, 1H), 2.16 – 2.05 (m, 3H), 1.91 – 1.83 (m, 2H), 1.82 – 1.70 (m, 3H), 1.69 – 1.62 (m, 2H), 1.56 – 1.39 (m, 8H), 0.99 (t, J = 7.5 Hz, 3H).

^{13}C NMR (151 MHz, $CDCl_3$) δ 170.9, 148.4, 136.4, 132.4, 128.8, 128.2, 103.7, 51.7, 42.1, 41.9, 37.4, 31.9, 31.1, 28.2, 28.1, 25.9, 21.8, 21.7, 18.5, 6.9.

IR (neat) ν_{max} (cm^{-1}) = 2984, 2933, 2877, 1744, 1672, 1552, 1461, 1432, 1381, 1285, 1177, 1088, 1051, 989, 961, 973, 871, 832, 811, 774, 732, 711, 691.

HRMS (Nanochip-based ESI/LTQ-Orbitrap) m/z: $[M + H]^+$ Calcd for $C_{23}H_{31}ClNO_3^+$ 404.1987; Found 404.1988.

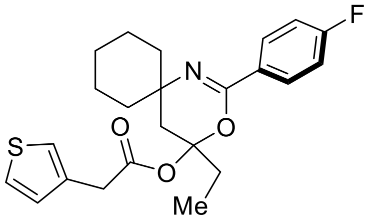

2ag. Colorless oil (16.2 mg, 39% yield).

Analytical data for **2ag**:

^{1}H NMR (600 MHz, $CDCl_3$) δ 8.03 – 7.95 (m, 2H), 7.24 (dd, *J* = 4.9, 3.0 Hz, 1H), 7.09 (d, *J* = 2.9 Hz, 1H), 7.06 (t, *J* = 8.5 Hz, 2H), 6.96 (dd, *J* = 5.0, 1.3 Hz, 1H), 3.61 (d, *J* = 15.8 Hz, 1H), 3.57 (d, *J* = 15.8 Hz, 1H), 2.78 (d, *J* = 14.8 Hz, 1H), 2.28 (dq, *J* = 14.9, 7.4 Hz, 1H), 2.17 (dq, *J* = 14.7, 7.4 Hz, 1H), 1.91 – 1.84 (m, 1H), 1.70 – 1.63 (m, 2H), 1.59 – 1.38 (m, 7H), 1.38 – 1.32 (m, 1H), 0.95 (t, *J* = 7.5 Hz, 3H).

^{13}C NMR (151 MHz, $CDCl_3$) δ 169.1, 164.3 (d, *J* = 250.4 Hz), 148.2, 133.1, 129.9, 129.6 (d, *J* = 8.6 Hz), 128.4, 125.7, 123.1, 115.0 (d, *J* = 21.6 Hz), 104.3, 51.5, 41.9, 37.4, 37.3, 36.8, 31.1, 25.8, 21.8, 21.7, 6.9.

^{19}F NMR (377 MHz, $CDCl_3$) δ -110.5.

IR (neat) ν_{max} (cm^{-1}) = 2982, 2971, 2844, 1712, 1655, 1619, 1575, 1443, 1367, 1275, 1177, 1148, 1069, 1038, 925, 891, 884, 752, 696.

HRMS (APCI/QTOF) m/z: $[M + H]^+$ Calcd for $C_{23}H_{27}FNO_3S^+$ 416.1690; Found 416.1684.

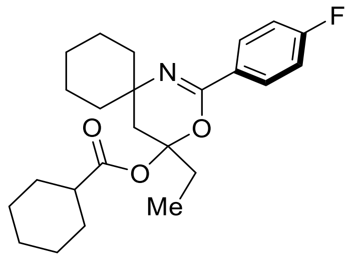

2ah. Colorless oil (22.9 mg, 57% yield).

Analytical data for **2ah**:

^{1}H NMR (500 MHz, $CDCl_3$) δ 8.00 (dd, *J* = 8.6, 5.6 Hz, 2H), 7.05 (t, *J* = 8.7 Hz, 2H), 2.87 (d, *J* = 14.8 Hz, 1H), 2.32 (dq, *J* = 14.9, 7.5 Hz, 1H), 2.22 (tt, *J* = 11.5, 3.6 Hz, 1H), 2.12 (dq, *J* = 14.7, 7.4 Hz, 1H), 1.92 – 1.82 (m, 3H), 1.78 – 1.67 (m, 4H), 1.64 – 1.55 (m, 3H), 1.52 – 1.41 (m, 6H), 1.40 – 1.31 (m, 2H), 1.27 – 1.15 (m, 3H), 1.00 (t, *J* = 7.5 Hz, 3H).

^{13}C NMR (126 MHz, $CDCl_3$) δ 174.3, 164.3 (d, *J* = 249.4 Hz), 148.5, 130.1, 129.6 (d, *J* = 8.7 Hz), 114.9 (d, *J* = 21.6 Hz), 103.6, 51.6, 44.0, 42.0, 37.4, 37.2, 31.2, 29.3, 28.6, 25.8, 25.6, 25.5, 25.3, 21.9, 21.8, 6.8.

^{19}F NMR (377 MHz, $CDCl_3$) δ -110.7.

IR (neat) ν_{max} (cm^{-1}) = 2953, 2848, 2825, 1734, 1688, 1548, 1477, 1392, 1352, 1283, 1144, 1058, 1016, 949, 883, 779, 692.

HRMS (APCI/QTOF) m/z: $[M + H]^+$ Calcd for $C_{24}H_{33}FNO_3^+$ 402.2439; Found 402.2430.

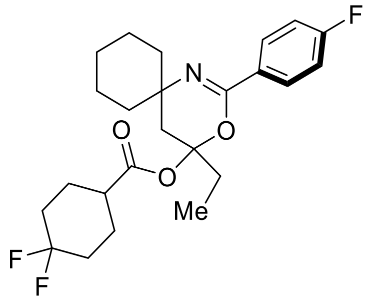

2ai. Colorless oil (22.7 mg, 52% yield).

Analytical data for **2ai**:

^{1}H NMR (500 MHz, $CDCl_3$) δ 7.99 (dd, J = 8.5, 5.5 Hz, 2H), 7.06 (t, J = 8.7 Hz, 2H), 2.83 (d, J = 14.8 Hz, 1H), 2.35 – 2.27 (m, 2H), 2.20 – 2.03 (m, 3H), 2.01 – 1.85 (m, 3H), 1.83 – 1.65 (m, 6H), 1.60 – 1.38 (m, 8H), 1.01 (t, J = 7.5 Hz, 3H).

^{13}C NMR (126 MHz, $CDCl_3$) δ 172.4, 164.4 (d, J = 250.3 Hz), 148.3, 129.8, 129.6 (d, J = 8.6 Hz), 122.5 (t, J = 241.1 Hz), 115.0 (d, J = 21.7 Hz), 104.2, 51.6, 41.9, 41.5, 37.4, 32.6 (t, J = 24.4 Hz), 32.5 (t, J = 23.5 Hz), 31.2, 25.8, 25.3 (dd, J = 8.2, 1.9 Hz), 24.8 (dd, J = 8.2, 1.9 Hz), 21.8, 21.7, 6.9.

^{19}F NMR (377 MHz, $CDCl_3$) δ -93.9 (d, J = 237.1 Hz), -100.5 (d, J = 237.5 Hz), -110.4.

IR (neat) ν_{max} (cm^{-1}) = 2973, 2823, 1716, 1655, 1629, 1557, 1442, 1366, 1280, 1136, 1098, 1044, 996, 835, 778, 663.

HRMS (ESI/QTOF) m/z: $[M + H]^+$ Calcd for $C_{24}H_{31}F_3NO_3^+$ 438.2251; Found 438.2260.

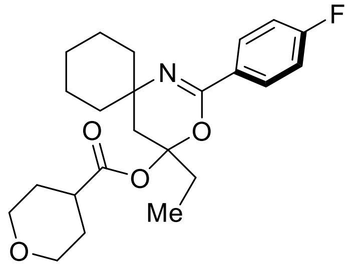

2aj. Colorless oil (20.6 mg, 51% yield).

Analytical data for **2aj**:

^{1}H NMR (500 MHz, $CDCl_3$) δ 7.99 (dd, J = 8.6, 5.5 Hz, 2H), 7.05 (t, J = 8.6 Hz, 2H), 3.97 – 3.90 (m,

2H), 3.40 – 3.34 (m, 2H), 2.85 (d, J = 14.8 Hz, 1H), 2.50 – 2.43 (m, 1H), 2.31 (dq, J = 13.9, 6.9, 6.4 Hz, 1H), 2.15 (dq, J = 14.7, 7.4 Hz, 1H), 1.91 – 1.83 (m, 1H), 1.82 – 1.68 (m, 6H), 1.59 – 1.38 (m, 8H), 1.01 (t, J = 7.5 Hz, 3H).

^{13}C NMR (126 MHz, $CDCl_3$) δ 172.6, 164.3 (d, J = 250.4 Hz), 148.4, 129.9, 129.6 (d, J = 8.8 Hz), 115.0 (d, J = 21.9 Hz), 104.1, 67.04, 66.95, 51.6, 41.9, 41.0, 37.5, 37.4, 31.2, 28.8, 28.3, 25.8, 21.8, 21.7, 6.9.

^{19}F NMR (377 MHz, $CDCl_3$) δ -110.5.

IR (neat) ν_{max} (cm^{-1}) = 2963, 2837, 1722, 1677, 1648, 1553, 1482, 1471, 1333, 1289, 1140, 1076, 923, 847, 745, 733, 692, 618.

HRMS (ESI/QTOF) m/z: $[M + H]^+$ Calcd for $C_{23}H_{31}FNO_4^+$ 404.2232; Found 404.2236.

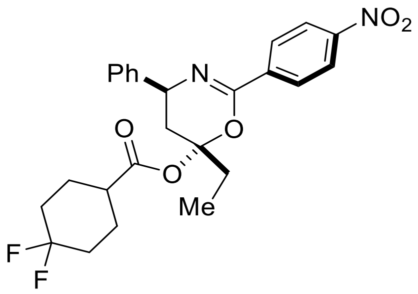

2ak. White solid (23.6 mg, 50% yield, 12:1 dr).

Analytical data for **2ak**:

m.p. = 104 ~ 106 °C.

^{1}H NMR (400 MHz, $CDCl_3$) δ 8.28 – 8.23 (m, 4H), 7.43 – 7.36 (m, 4H), 7.35 – 7.29 (m, 1H), 4.74 (dd, J = 12.4, 5.0 Hz, 1H), 2.90 (dd, J = 14.2, 5.0 Hz, 1H), 2.47 (tt, J = 10.3, 3.2 Hz, 1H), 2.42 – 2.29 (m, 2H), 2.18 – 1.96 (m, 4H), 1.92 – 1.67 (m, 5H), 1.06 (t, J = 7.5 Hz, 3H).

^{13}C NMR (151 MHz, $CDCl_3$) δ 172.0, 151.6, 149.4, 142.4, 138.6, 128.7, 128.5, 127.3, 126.4, 123.4, 122.4 (t, J = 241.4 Hz), 104.1, 53.0, 41.4, 36.4, 32.52 (t, J = 24.9 Hz), 32.50 (t, J = 24.8 Hz), 29.8, 25.3 – 25.1 (m, 2C), 7.2.

^{19}F NMR (377 MHz, $CDCl_3$) δ -94.0 (d, J = 238.0 Hz), -100.4 (d, J = 238.0 Hz).

IR (neat) ν_{max} (cm^{-1}) = 2988, 2843, 1704, 1644, 1617, 1540, 1433, 1412, 1385, 1217, 1109, 1053, 1021, 986, 844, 766, 682.

HRMS (ESI/QTOF) m/z: $[M + H]^+$ Calcd for $C_{25}H_{27}F_2N_2O_5^+$ 473.1883; Found 473.1878.

General procedure for the evaluation of trisubstituted substrate

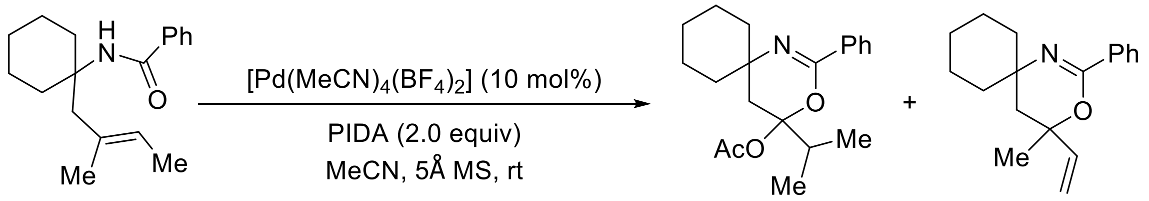

At room temperature, to a 10-mL test tube charged with a magnetic stirring bar were added substrate **3** (21.1 mg, 0.1 mmol, 1.0 equiv), PIDA (64.4 mg, 0.2 mmol, 2.0 equiv), [Pd(MeCN)$_4$(BF$_4$)$_2$] (4.5 mg, 0.01 mmol, 10 mol%), 5Å molecular sieve (50 mg) and anhydrous MeCN (1.0 mL). The reaction mixture was allowed to stir at room temperature. After the reaction was complete (monitored by TLC), diethyl ether (2 mL) was added and the reaction mixture was filtered through a short pad of silica gel with diethyl ether. The filtrate was concentrated. The residue was purified by silica gel flash column chromatography to give the desired product **4**.

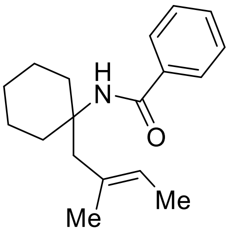

3. White solid.

Analytical data for **3**:

m.p. = 105 ~ 107 ºC.

^{1}H NMR (500 MHz, CDCl$_3$) δ 7.73 – 7.70 (m, 2H), 7.49 – 7.46 (m, 1H), 7.44 – 7.40 (m, 2H), 5.68 (s, 1H), 5.24 (qd, *J* = 6.8, 1.6 Hz, 1H), 2.57 (s, 2H), 2.29 – 2.22 (m, 2H), 1.65 (t, *J* = 1.3 Hz, 3H), 1.63 – 1.57 (m, 3H), 1.55 (d, *J* = 6.7 Hz, 3H), 1.49 – 1.37 (m, 4H), 1.33 – 1.24 (m, 1H).

^{13}C NMR (126 MHz, CDCl$_3$) δ 167.1, 136.5, 132.1, 131.0, 128.5, 126.6, 123.7, 56.8, 47.2, 35.2, 25.7, 22.0, 18.2, 13.6.

IR (neat) ν_{max} (cm^{-1}) = 2921, 2907, 2853, 1703, 1667, 1638, 1524, 1509, 1488, 1460, 1445, 1316, 1282, 719, 712, 692, 670, 661.

HRMS (ESI/QTOF) m/z: [M + H]$^+$ Calcd for $C_{18}H_{26}NO^+$ 272.2009; Found 272.2009.

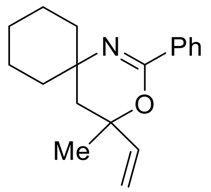

4. White solid (7.3 mg, 27% yield).

Analytical data for **4**:

m.p. = 79 ~ 81 °C.

^{1}H NMR (500 MHz, $CDCl_3$) δ 8.16 – 7.96 (m, 2H), 7.44 – 7.32 (m, 3H), 5.88 (dd, *J* = 17.3, 10.8 Hz, 1H), 5.21 (d, *J* = 17.2 Hz, 1H), 5.00 (d, *J* = 10.8 Hz, 1H), 2.14 (d, *J* = 14.0 Hz, 1H), 1.93 – 1.84 (m, 1H), 1.78 – 1.69 (m, 2H), 1.64 – 1.55 (m, 3H), 1.51 – 1.36 (m, 5H), 1.44 (s, 3H).

^{13}C NMR (126 MHz, $CDCl_3$) δ 151.1, 142.9, 134.9, 130.0, 127.9, 127.3, 112.0, 76.0, 52.2, 42.9, 42.0, 37.5, 29.9, 26.0, 22.1, 21.9.

IR (neat) ν_{max} (cm^{-1}) = 2954, 2930, 2855, 1702, 1638, 1579, 1538, 1523, 1509, 1487, 1373, 1362, 1349, 1336, 1328, 1309, 1273, 1260, 1074, 713, 692, 673, 663.

HRMS (ESI/QTOF) m/z: $[M + H]^+$ Calcd for $C_{18}H_{24}NO^+$ 270.1852; Found 270.1853.

General procedure for the evaluation of chiral (*R*)-1p in palladium-catalyzed dyotropic rearrangement

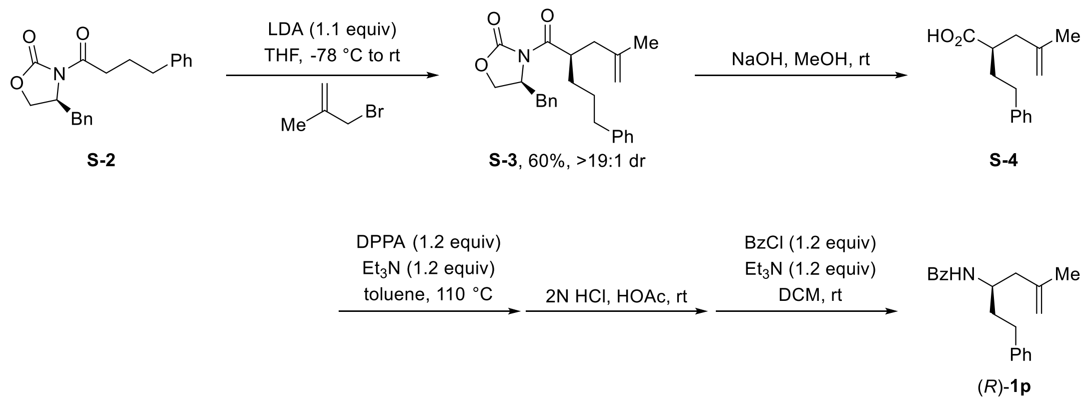

Step 1: To a solution of **S-2**[6] in THF (1 M) at -78 ºC was added LDA (1.0 M solution in THF, 1.1 equiv) dropwise. The mixture was stirred at -78 ºC for 1 h and 3-bromo-2-methylpropene (1.2 equiv) was added slowly. The reaction mixture was allowed to warm to room temperature and stirred overnight. Then, the mixture was quenched with dilute HCl (2 M) and extracted with ethyl acetate (twice). The extracts were combined, dried over Na_2SO_4, filtered and concentrated under reduced pressure. The residue was purified by silica gel flash column chromatography (eluent: hexanes / ethyl acetate = 20:1 → 10:1) to give the desired product **S-3**. The diastereoselectivity was determined based on the ^1H NMR spectrum of the crude product before it was purified by silica gel column chromatography.

Step 2: To a solution of **S-3** in MeOH (1 mL) was added NaOH (5.0 equiv) and the reaction mixture was stirred at room temperature. After the reaction was complete (monitored by TLC), the residue was diluted with water, acidified until pH = 1 with HCl (3 M), and extracted twice with ethyl acetate. The combined organic extracts were dried over Na_2SO_4 and concentrated under reduced pressure to give the product **S-4** which was used for the next step without further purification. Compound **S-4** was used for the next step following the *General procedure for the synthesis of substrates* to afford amide (*R*)-**1p**.

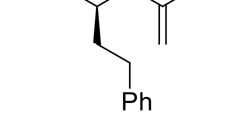

(*R*)-**1p**. White solid (252.2 mg, 43% yield, 90% *ee*, 2.0 mmol scale).

Analytical data for (*R*)-**1p**:

m.p. = 121 ~ 123 ºC.

[α]D28 = +0.6 (*c* 0.4, $CHCl_3$).

HPLC: The enantiomeric excess was determined by Daicel Chiralpack IB (0.46 cm x 25 cm), Hexanes / IPA = 95 / 05, 1.0 mL/min, λ = 254 nm, t (minor) = 16.45 min, t (major) = 17.89 min.

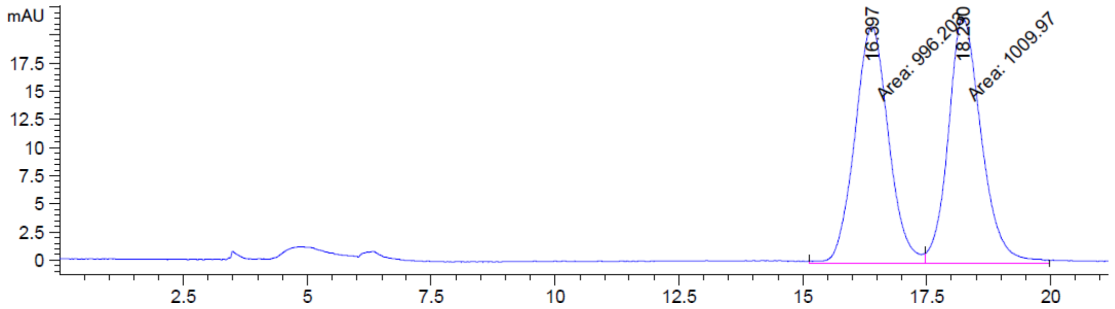

Peak #	RetTime [min]	Type	Width [min]	Area [mAU*s]	Height [mAU]	Area %
1	16.397	MF	0.7908	996.20251	20.99629	49.6569
2	18.230	FM	0.7722	1009.97009	21.79729	50.3431

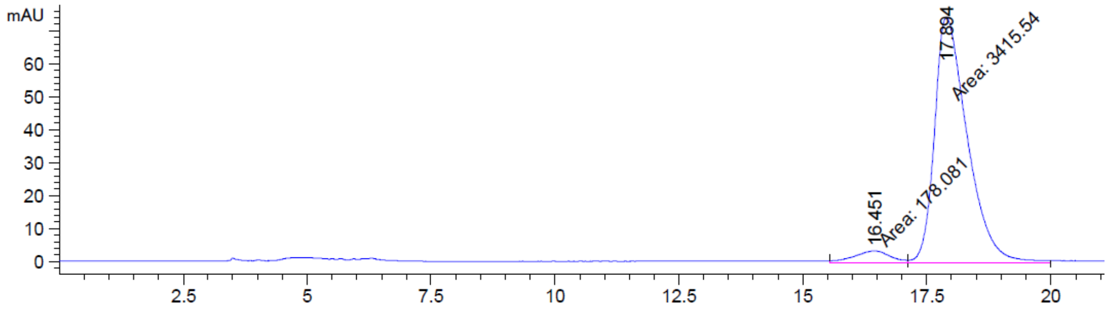

Peak #	RetTime [min]	Type	Width [min]	Area [mAU*s]	Height [mAU]	Area %
1	16.451	MF	0.8411	178.08084	3.52872	4.9555
2	17.894	FM	0.7659	3415.54224	74.32400	95.0445

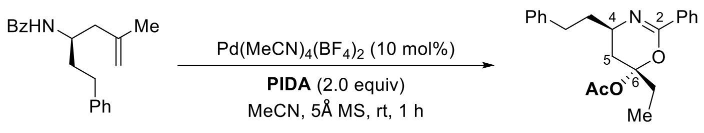

(*R*)-**1p**, 90% ee

(4*R*, 6*R*)-**2p**
50%, 10:1 dr, 90% ee

At room temperature, to a 10-mL test tube charged with a magnetic stirring bar were added substrate (*R*)-**1p** (29.3 mg, 0.1 mmol, 1.0 equiv), PIDA (64.4 mg, 0.2 mmol, 2.0 equiv), [Pd(MeCN)$_4$(BF$_4$)$_2$] (4.5 mg, 0.01 mmol, 10 mol%), 5Å molecular sieves (50 mg) and anhydrous MeCN (1.0 mL). The reaction mixture was allowed to stir at room temperature. After the reaction was complete (monitored by TLC), diethyl ether (2 mL) was added and the reaction mixture was filtered through a short pad of silica gel with diethyl ether. The filtrate was concentrated. The residue was purified by silica gel flash column chromatography to give the desired product (4*R*,6*R*)-**2p**. The diastereoselectivity was determined by the ^{1}H NMR spectrum of the crude product before the residue was purified by silica gel column chromatography.

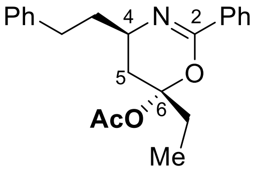

(4*R*,6*R*)-**2p**. Colorless oil (17.6 mg, 50% yield, 10:1 dr, 90% *ee*).

Analytical data for (4*R*,6*R*)-**2p**:

[α]$_D$^{28} = +6.2 (*c* 0.4, $CHCl_3$).

HPLC: The enantiomeric excess was determined by Daicel Chiralpack IC (0.46 cm x 25 cm), Hexanes / IPA = 99 / 01, 1.0 mL/min, λ = 254 nm, t (major) = 6.37 min, t (minor) = 6.80 min.

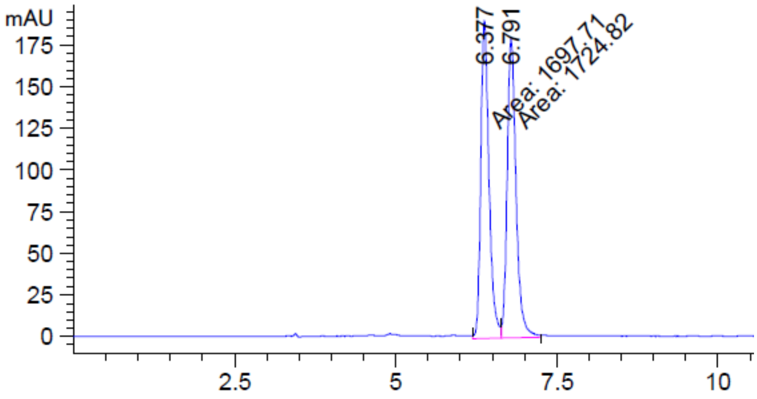

Peak #	RetTime [min]	Type	Width [min]	Area [mAU*s]	Height [mAU]	Area %
1	6.377	MF	0.1481	1697.71033	190.99828	49.6039
2	6.791	FM	0.1591	1724.82410	180.71880	50.3961

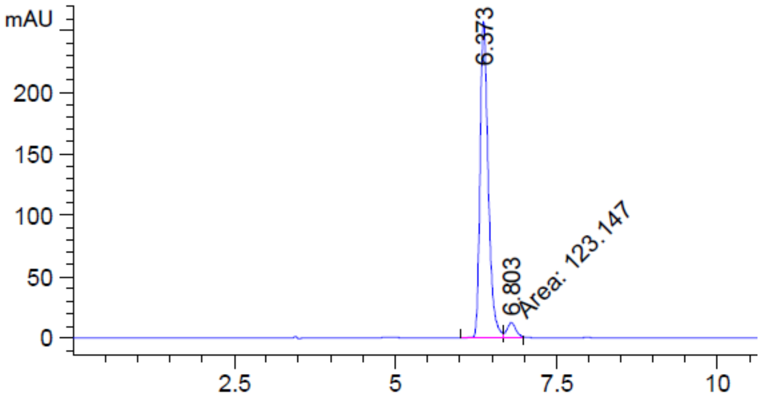

Peak #	RetTime [min]	Type	Width [min]	Area [mAU*s]	Height [mAU]	Area %
1	6.373	BV	0.1351	2276.72314	257.52094	94.8686
2	6.803	MF	0.1634	123.14674	12.56302	5.1314

Mechanistic study

^{13}C labeling experiment

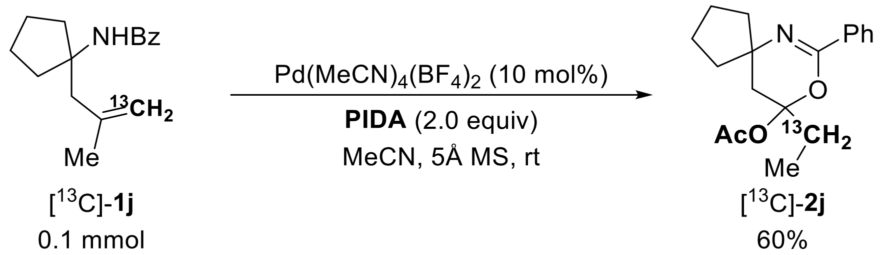

At room temperature, to a 10-mL test tube charged with a magnetic stirring bar were added substrate [^{13}C]-**1j** (24.4 mg, 0.1 mmol, 1.0 equiv), PIDA (64.4 mg, 0.2 mmol, 2.0 equiv), [Pd(MeCN)$_4$(BF$_4$)$_2$] (4.5 mg, 0.01 mmol, 10 mol%), 5Å molecular sieves (50 mg) and anhydrous MeCN (1.0 mL). The reaction mixture was allowed to stir at room temperature. After the reaction was complete (monitored by TLC), diethyl ether (2 mL) was added and the reaction mixture was filtered through a short pad of silica gel with diethyl ether. The filtrate was concentrated. The residue was purified by silica gel flash column chromatography to give the desired product [^{13}C]-**2j**.

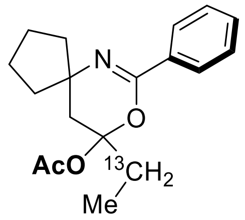

[^{13}C]-**2j**. Colorless oil (18.1 mg, 60% yield).

Analytical data for [^{13}C]-**2j**:

^{1}H NMR (600 MHz, CDCl$_3$) δ 7.98 (d, J = 7.5 Hz, 2H), 7.41 (t, J = 7.2 Hz, 1H), 7.37 (t, J = 7.4 Hz, 2H), 2.65 (d, J = 14.4 Hz, 1H), 2.34 (ddq, J = 129.1, 14.9, 7.5 Hz, 1H), 2.16 (ddq, J = 128.7, 14.9, 7.5 Hz, 1H), 2.09 – 1.96 (m, 2H), 2.02 (s, 3H), 1.91 (d, J = 14.4 Hz, 1H), 1.87 – 1.80 (m, 1H), 1.77 – 1.64 (m, 3H), 1.62 – 1.57 (m, 2H), 1.04 (td, J = 7.4, 4.3 Hz, 3H).

^{13}C NMR (151 MHz, CDCl$_3$) δ 169.4, 148.9, 133.8, 130.3, 128.0, 127.4, 103.8 (d, J = 45.2 Hz), 59.9, 42.9, 40.0, 37.3, 30.6, 24.7, 22.9, 22.1, 7.0 (d, J = 34.8 Hz).

IR (neat) ν_{max} (cm^{-1}) = 2953, 2861, 1716, 1654, 1587, 1553, 1422, 1495, 1375, 1162, 1077, 1061, 977, 944, 861, 752, 746, 716, 684.

HRMS (ESI/QTOF) m/z: $[M + H]^+$ Calcd for $C_{17}[^{13}C]H_{24}NO_3^+$ 303.1784; Found 303.1783.

General procedure for the synthesis of palladium intermediate 3

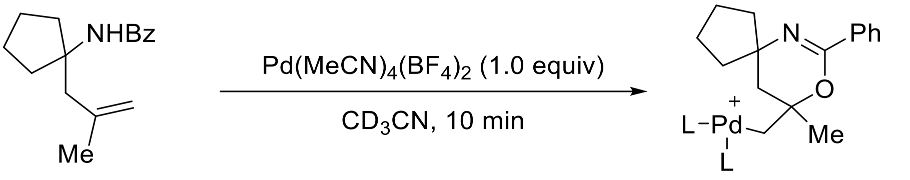

1j 0.025 mmol — L = MeCN or CD_3CN **5**

At room temperature, to a 10-mL test tube charged with a magnetic stirring bar were added substrate **1j** (6.1 mg, 0.025 mmol, 1.0 equiv), $[Pd(MeCN)_4(BF_4)_2]$ (11.1 mg, 0.025 mmol, 1.0 equiv) and anhydrous CD_3CN (1.0 mL). The reaction mixture was allowed to stir at room temperature and the ^1H NMR of the crude reaction mixture showed a full conversion to palladium intermediate **5**.

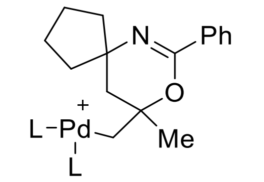

L = MeCN or CD_3CN

Analytical data for palladium intermediate **5**:

^{1}**H NMR** (400 MHz, Acetonitrile-d_3) δ 7.99 – 7.93 (m, 2H), 7.84 – 7.78 (m, 1H), 7.70 – 7.62 (m, 2H), 2.58 (d, J = 14.7 Hz, 1H), 2.47 (s, 2H), 2.46 (d, J = 15.0 Hz, 1H), 2.21 (ddd, J = 13.5, 8.1, 6.2 Hz, 1H), 2.16 – 2.09 (m, 2H), 2.08 – 2.00 (m, 1H), 1.94 – 1.87 (m, 2H), 1.84 – 1.73 (m, 2H), 1.80 (s, 3H).

^{13}**C NMR** (151 MHz, Acetonitrile-d_3) δ 168.2, 136.5, 130.3, 129.8, 127.3, 92.0, 64.0, 43.2, 42.0, 40.1, 27.7, 25.3, 24.5, 22.1.

General procedure for the synthesis of 2j from palladium intermediate 3

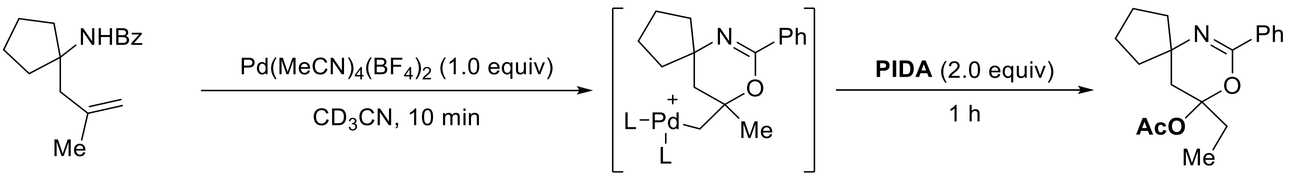

1j 0.025 mmol — L = MeCN or CD_3CN **5** — **2j** 52%

At room temperature, to a 10-mL test tube charged with a magnetic stirring bar were added substrate **1j** (6.1 mg, 0.025 mmol, 1.0 equiv), [Pd(MeCN)$_4$(BF$_4$)$_2$] (11.1 mg, 0.025 mmol, 1.0 equiv) and anhydrous CD_3CN (1.0 mL). The reaction mixture was allowed to stir for 10 min at room temperature. Then PIDA (16.1 mg, 0.05 mmol, 2.0 equiv) was added. After the reaction was complete (monitored by TLC), diethyl ether (2 mL) was added and the reaction mixture was filtered through a short pad of silica gel with diethyl ether. The filtrate was concentrated. The residue was purified by silica gel flash column chromatography to give the desired product **2j** (3.9 mg, 52% yield).

HRMS data of palladium intermediate 3: Experimental isotopic peaks versus predicted isotopic peaks

5-(MeCN): **HRMS** (Nanochip-based ESI/LTQ-Orbitrap) m/z: $[M]^+$ Calcd for $C_{28}H_{23}N_2OPd^+$ 389.0840; Found 389.0847. (*Note: For the sake of a clear HRMS,* ***5-(MeCN)*** *was prepared from* ***MeCN*** *instead of CD_3CN).*

Experimental mass result of **5-(MeCN)**:

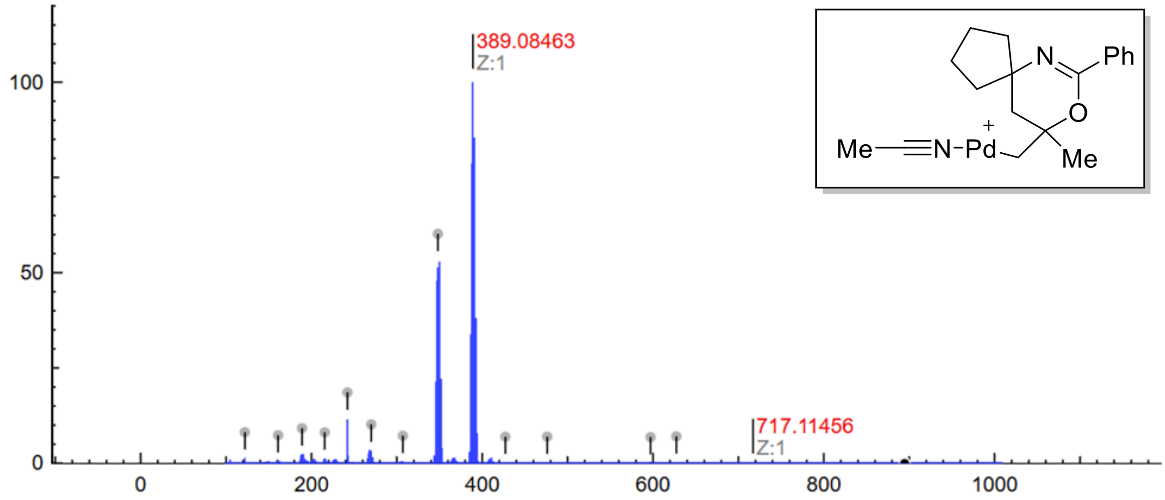

Experimental mass result of **3-(MeCN)**

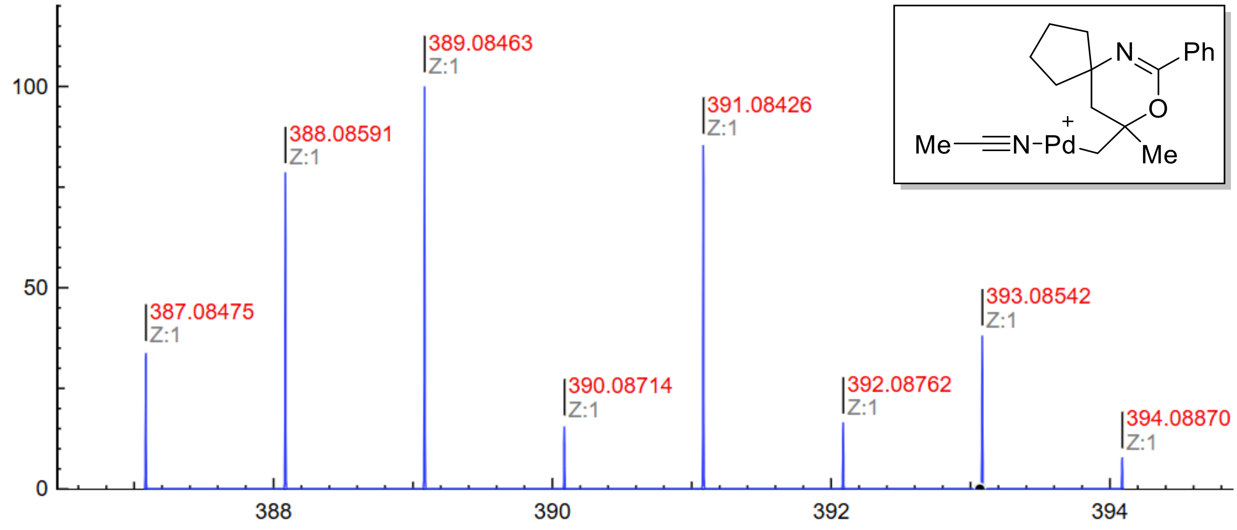

Experimental isotopic peaks of **($C_{16}H_{20}NO$)Pd**·MeCN

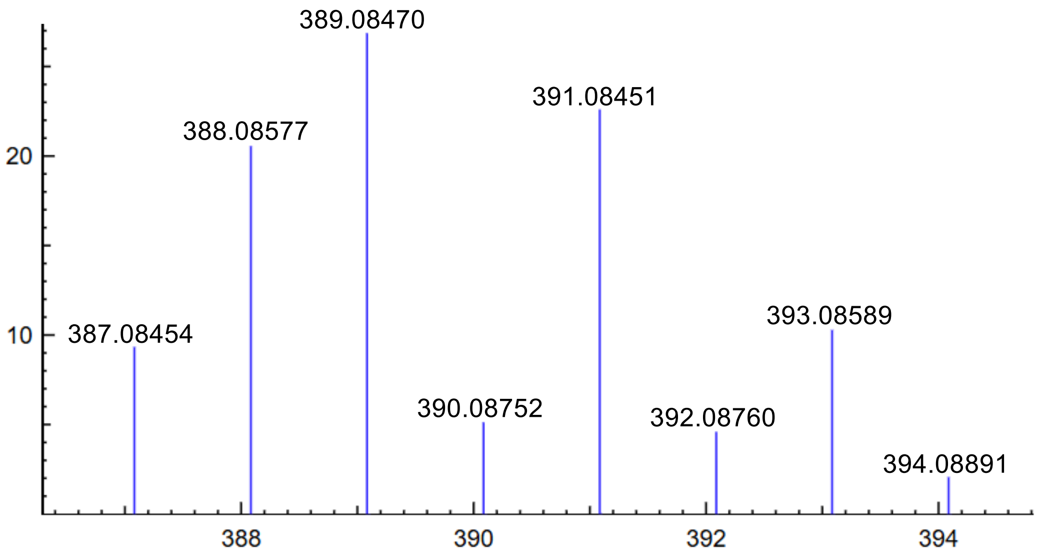

Predicted isotopic peaks of **($C_{16}H_{20}NO$)Pd**·MeCN

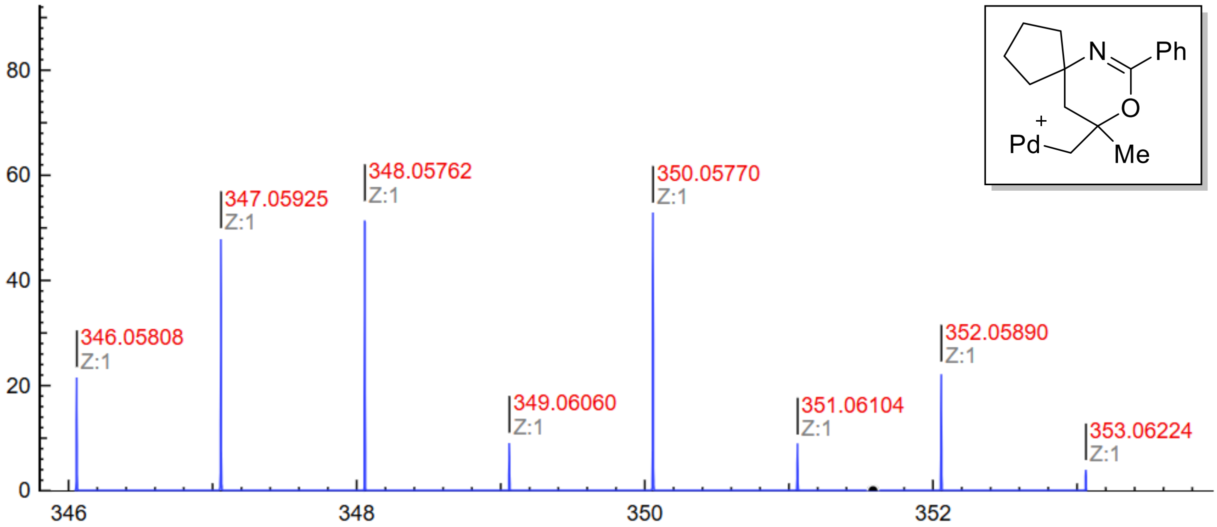

Experimental isotopic peaks of **($C_{16}H_{20}NO$)Pd**

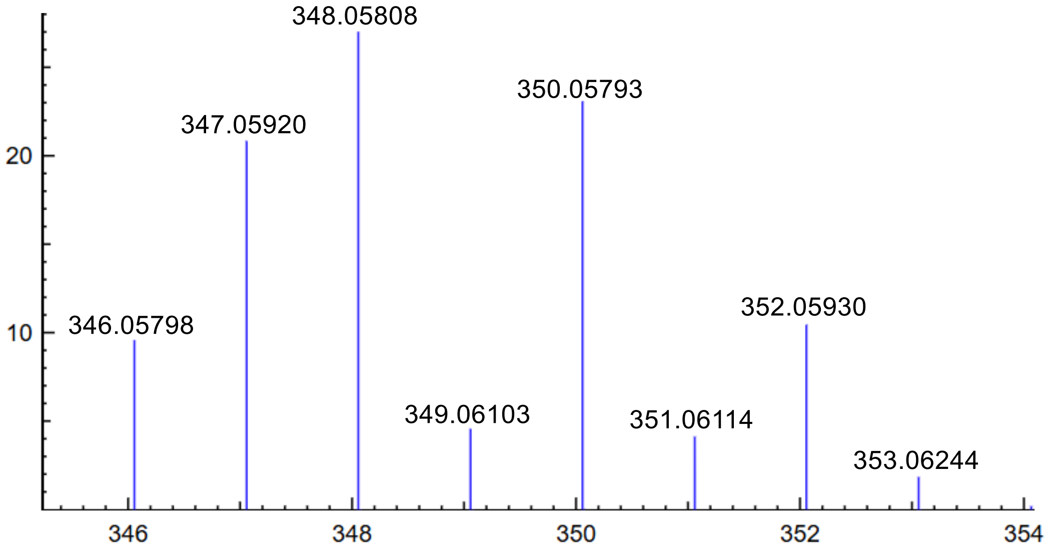

Predicted isotopic peaks of **($C_{16}H_{20}NO$)Pd**

Trapping of the oxypalladation intermediate

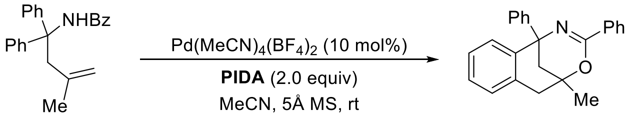

1al 0.1 mmol

7 51%

At room temperature, to a 10-mL test tube charged with a magnetic stirring bar were added substrate **1al** (34.1 mg, 0.1 mmol, 1.0 equiv), PIDA (64.4 mg, 0.2 mmol, 2.0 equiv), [Pd(MeCN)$_4$(BF$_4$)$_2$] (4.5 mg, 0.01 mmol, 10 mol%), 5Å molecular sieves (50 mg) and anhydrous MeCN (1.0 mL). The reaction mixture was allowed to stir at room temperature. After the reaction was complete (monitored by TLC), diethyl ether (2 mL) was added and the reaction mixture was filtered through a short pad of silica gel with diethyl ether. The filtrate was concentrated. The residue was purified by silica gel flash column chromatography to give the desired product **7**.

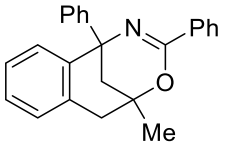

7. Colorless oil (17.3 mg, 51% yield).

Analytical data for **7**:

^{1}H NMR (600 MHz, CDCl$_3$) δ 8.31 (br, 2H), 8.06 (d, *J* = 7.5 Hz, 2H), 7.41 (br, 2H), 7.39 (t, *J* = 7.0 Hz, 1H), 7.35 (t, *J* = 7.4 Hz, 2H), 7.31 (t, *J* = 7.3 Hz, 1H), 7.13 (d, *J* = 7.5 Hz, 1H), 7.10 (t, *J* = 7.3 Hz, 1H), 7.01 (t, *J* = 7.5 Hz, 1H), 6.86 (d, *J* = 8.0 Hz, 1H), 3.34 (d, *J* = 17.7 Hz, 1H), 3.27 (d, *J* = 17.7 Hz, 1H), 2.59 (d, *J* = 13.1 Hz, 1H), 1.91 (d, *J* = 13.0 Hz, 1H), 1.64 (s, 3H).

^{13}C NMR (151 MHz, CDCl$_3$) δ 156.0, 147.0, 142.2, 134.3, 133.0, 130.2, 129.1, 128.9, 128.0, 127.8, 127.4, 126.6, 126.5, 126.2, 75.3, 59.1, 44.1, 43.0, 28.2.

IR (neat) ν_{max} (cm^{-1}) = 2973, 2911, 2858, 1731, 1655, 1532, 1529, 1442, 1344, 1165, 1072, 1011, 993, 955, 866, 841, 801, 761, 655.

HRMS (ESI/QTOF) m/z: [M + H]$^+$ Calcd for $C_{24}H_{22}NO^+$ 340.1696; Found 340.1704.

General procedure for the measurement of reaction orders

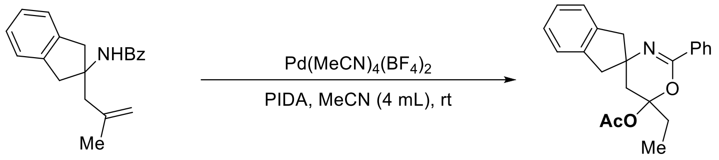

1h **2h**

At room temperature, ~~to~~ a 10-mL test tube equipped with a magnetic stirring bar was charged with substrate **1h**, PIDA and anhydrous MeCN (4.0 mL). Subsequently, $[Pd(MeCN)_4(BF_4)_2]$ was added. The mixture was stirred at room temperature (at a rate of 500 rpm), and aliquots of the reaction mixture (100 μL) were withdrawn via syringe at specified time intervals. Each aliquot was evaporated under reduced pressure, dissovled in $CDCl_3$ (600 μL), passed through a small plug of cotton into an NMR tube, and analyzed by quantitative ^{1}H NMR using mesitylene as an internal standard.

Reaction order of $[Pd(MeCN)_4(BF_4)_2]$

The concentrations of **1h** used: 0.1 M

The concentrations of PIDA used: 0.2 M

The concentrations of $[Pd(MeCN)_4(BF_4)_2]$ used: 0.005 M, 0.007 M, 0.010 M, 0.015 M.

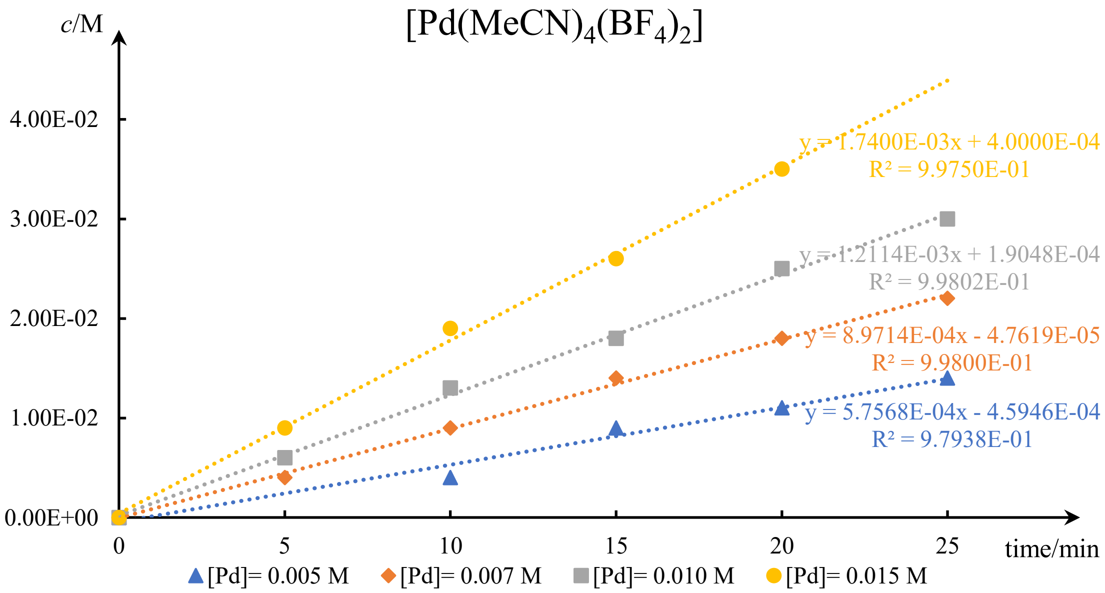

Figure S1. **1h** concentration vs. time for reactions with varying $[Pd(MeCN)_4(BF_4)_2]$ concentrations.

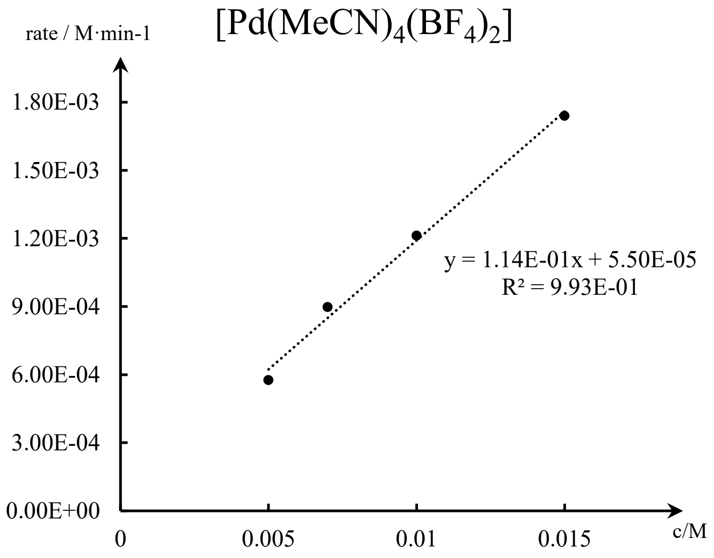

Figure S2. First-order dependence of the reaction rate on [$Pd(MeCN)_4(BF_4)_2$] concentration.

Reaction order of 1h

The concentrations of [$Pd(MeCN)_4(BF_4)_2$] used: 0.010 M

The concentrations of PIDA used: 0.2 M

The concentrations of **1h** used: 0.025 M, 0.050 M, 0.075 M, 0.100 M.

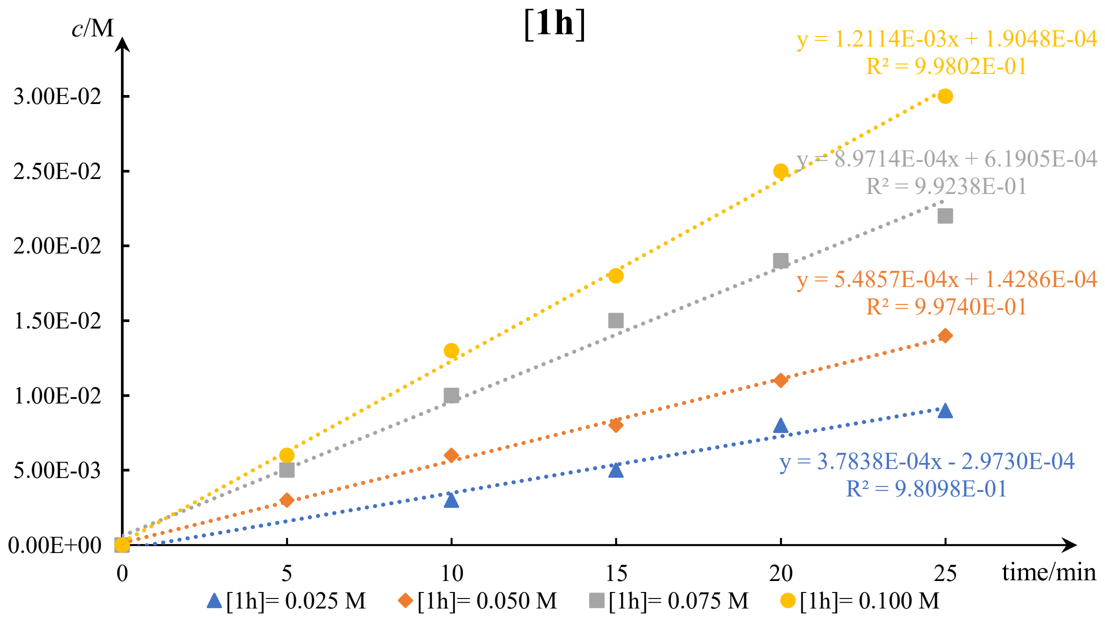

Figure S3. 1h concentration vs. time for reactions with varying initial **1h** concentrations.

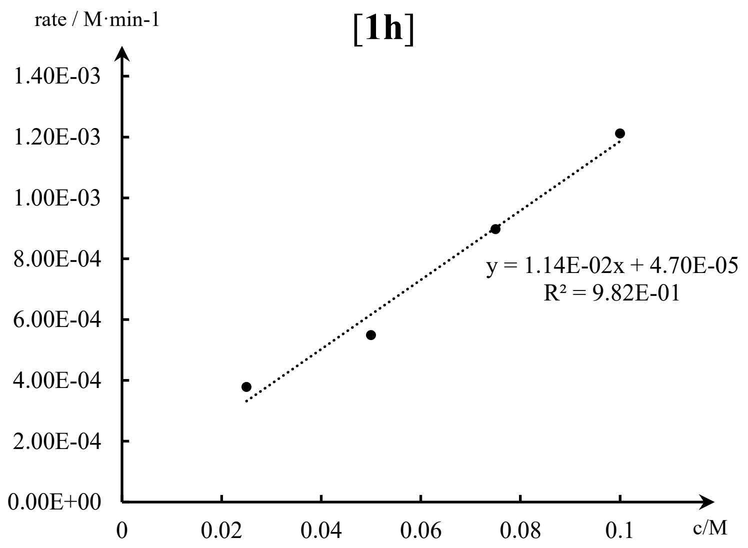

Figure S4. First-order dependence of the reaction rate on **1h** concentration.

Reaction order of PIDA

The concentrations of [Pd(MeCN)$_4$(BF$_4$)$_2$] used: 0.010 M

The concentrations of **1h** used: 0.1 M

The concentrations of PIDA used: 0.050 M, 0.100 M, 0.150 M, 0.200 M.

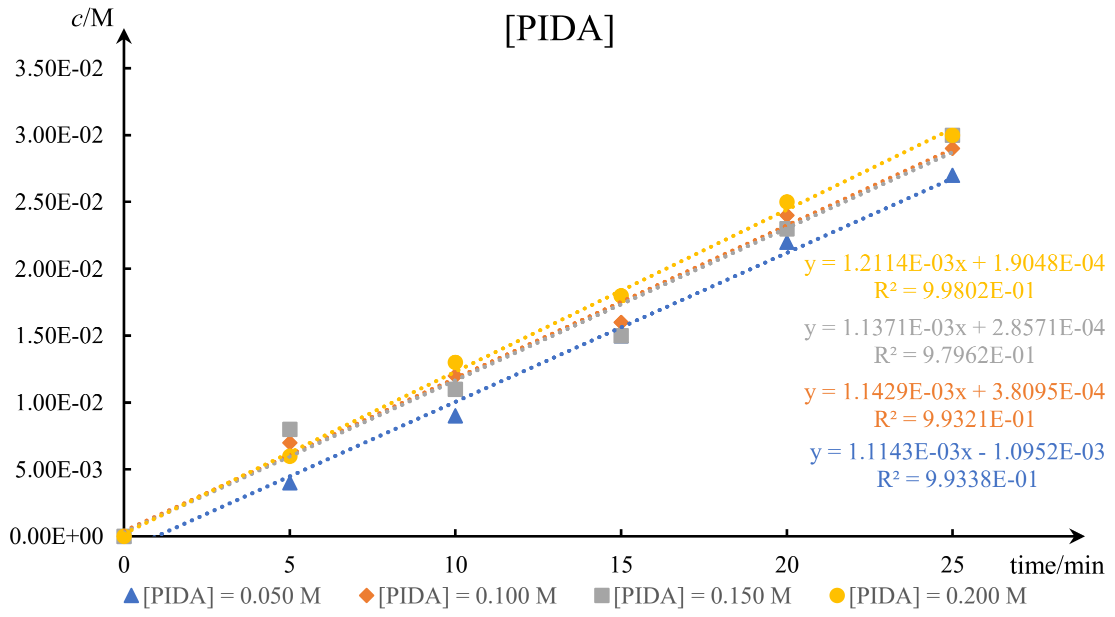

Figure S5. 1h concentration vs. time for reactions with varying PIDA concentrations.

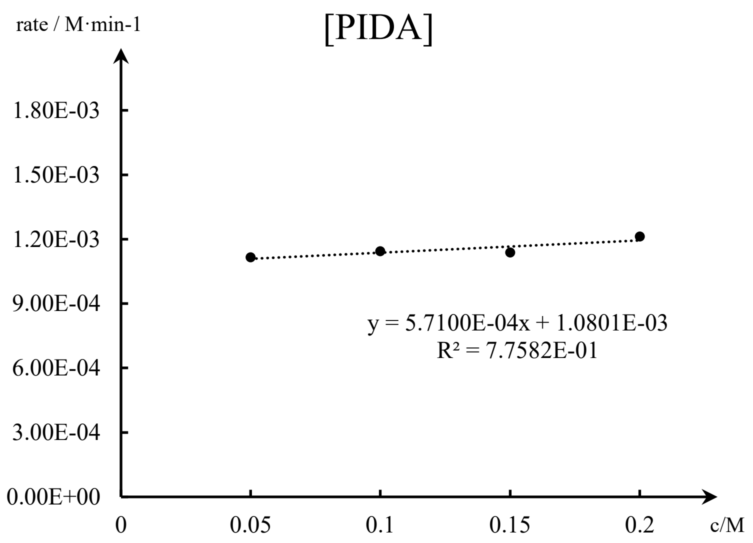

Figure S6. Zero-order dependence of the reaction rate on PIDA concentration.

X-Ray crystallographic data

X-Ray crystallographic data of **2ak** (CCDC 2443462):

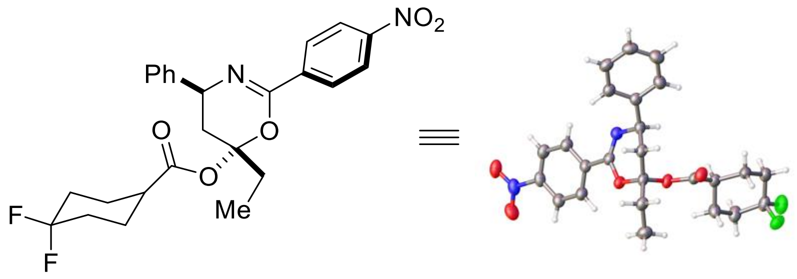

Experimental. Single clear pale colourless prism-shaped crystals of **2ak** were used as supplied. A suitable crystal with dimensions 0.16 × 0.15 × 0.09 mm^3 was selected and mounted on a XtaLAB Synergy R, DW system, HyPix-Arc 150 diffractometer. The crystal was kept at a steady T = 140.00(10) K during data collection. The structure was solved with the ShelXT (Sheldrick, 2015) solution program using dual methods and by using Olex2 1.5 (Dolomanov et al., 2009) as the graphical interface. The model was refined with ShelXL 2019/3 (Sheldrick, 2015) using full matrix least squares minimisation on $\boldsymbol{F}^2$.

Crystal Data. $C_{25}H_{26}F_2N_2O_5$, M_r = 472.48, triclinic, P-1 (No. 2), a = 9.6159(4) Å, b = 11.4601(6) Å, c = 11.9314(6) Å, α = 75.493(4)°, β = 69.621(5)°, γ = 70.138(4)°, V = 1146.11(11) Å^3, T = 140.00(10) K, Z = 2, Z' = 1, μ(Cu K$_\alpha$) = 0.895, 15172 reflections measured, 4421 unique (R_{int} = 0.0270) which were used in all calculations. The final wR_2 was 0.1459 (all data) and R_1 was 0.0513 (I≥2 σ(I)).

Table S2. Crystal data and structure refinement for **2ak**.

**Compound**	**2ak**
Formula	$C_{25}H_{26}F_2N_2O_5$
$D_{calc.}$/ g cm^{-3}	1.369
μ/mm^{-1}	0.895
Formula Weight	472.48
Colour	clear pale colourless
Shape	prism
Size/mm^3	0.16×0.15×0.09
T/K	140.00(10)
Crystal System	triclinic
Space Group	P-1
a/Å	9.6159(4)
b/Å	11.4601(6)
c/Å	11.9314(6)
α/°	75.493(4)
β/°	69.621(5)
γ/°	70.138(4)
V/Å^3	1146.11(11)
Z	2
Z'	1
Wavelength/Å	1.54184
Radiation type	Cu K_{α}
Θ_{min}/°	3.998
Θ_{max}/°	74.202
Measured Refl's.	15172
Indep't Refl's	4421
Refl's I≥2 σ(I)	3438
R_{int}	0.0270
Parameters	412
Restraints	0
Largest Peak	0.281
Deepest Hole	-0.268
GooF	1.042
wR_2 (all data)	0.1459
wR_2	0.1366
R_1 (all data)	0.0648
R_1	0.0513

Structure Quality Indicators

**Reflections:**	d min (CuKα) 2Θ=148.4° 0.80	I/σ(I) 38.4	Rint m=3.43 2.70%	Full 135.4° 95% to 148.4° 99.3
**Refinement:**	Shift 0.000	Max Peak 0.3	Min Peak -0.3	GooF 1.042

A clear pale colourless prism-shaped crystal with dimensions 0.16 × 0.15 × 0.09 mm^3 was mounted. Data were collected using a XtaLAB Synergy R, DW system, HyPix-Arc 150 diffractometer operating at T = 140.00(10) K.

Data were measured using ω scans with Cu K_α radiation. The diffraction pattern was indexed and the total number of runs and images was based on the strategy calculation from the program CrysAlisPro system (CCD 44.103a 64-bit (release 31-03-2025)). The maximum resolution that was achieved was Θ = 74.202° (0.80 Å).

The unit cell was refined using CrysAlisPro 1.171.44.104a (Rigaku OD, 2025) on 6962 reflections, 46% of the observed reflections.

Data reduction, scaling and absorption corrections were performed using CrysAlisPro 1.171.44.104a (Rigaku OD, 2025). The final completeness is 99.30 % out to 74.202° in Θ. A gaussian absorption correction was performed using CrysAlisPro 1.171.44.104a (Rigaku Oxford Diffraction, 2025). The numerical absorption correction was based on gaussian integration over a multifaceted crystal model. The empirical absorption correction was done using spherical harmonics, implemented in SCALE3 ABSPACK scaling algorithm. The absorption coefficient μ of this crystal is 0.895 mm^{-1} at this wavelength (λ = 1.54184Å) and the minimum and maximum transmissions are 0.675 and 1.000.

The structure was solved and the space group P-1 (# 2) determined by the ShelXT (Sheldrick, 2015) structure solution program using dual methods and refined by full matrix least squares minimisation on $\boldsymbol{F}^2$ using version 2019/3 of ShelXL (Sheldrick, 2015). All non-hydrogen atoms were refined anisotropically. Hydrogen atom positions were calculated geometrically and refined using the riding model.

There is a single formula unit in the asymmetric unit, which is represented by the reported sum formula. In other words: Z is 2 and Z' is 1. The moiety formula is C25 H26 F2 N2 O5.

References

1. Lin, H.; Pan, X.; Barsamian, A. L.; Kamenecka, T. M.; Bannister. T. D. *ACS Catal.* **2019**, *9*, 4887−4891.
2. Feng, Q.; Wang, Q.; Zhu, J. *Science* **2023**, *379*, 1363–1368.
3. Feng, Q.; Liu, C.-X.; Wang, Q.; Zhu, J. *Angew. Chem. Int. Ed*. **2024,** *63*, e20231639.
4. Liu, C.-X.; Wang, Q.; Zhu, J. *J. Am. Chem. Soc.* **2024**, *146*, 30014–30019.
5. Sakai, H. A.; MacMillan, D. W. C. *J. Am. Chem. Soc.* **2022**, *144*, 6185–6192.
6. Cherney, R. J.; Decicco, C. P.; Nelson, C. R.; Wan, L.; Meyer, D. T.; Hardman, K. D.; Copeland, R. A.; Arner, E. C. *Bioorg. Med. Chem. Lett.* **1997**, *7*, 1757–1762.

Copies of the NMR spectra

^{1}H NMR spectrum of **1g** (500 MHz, $CDCl_3$)

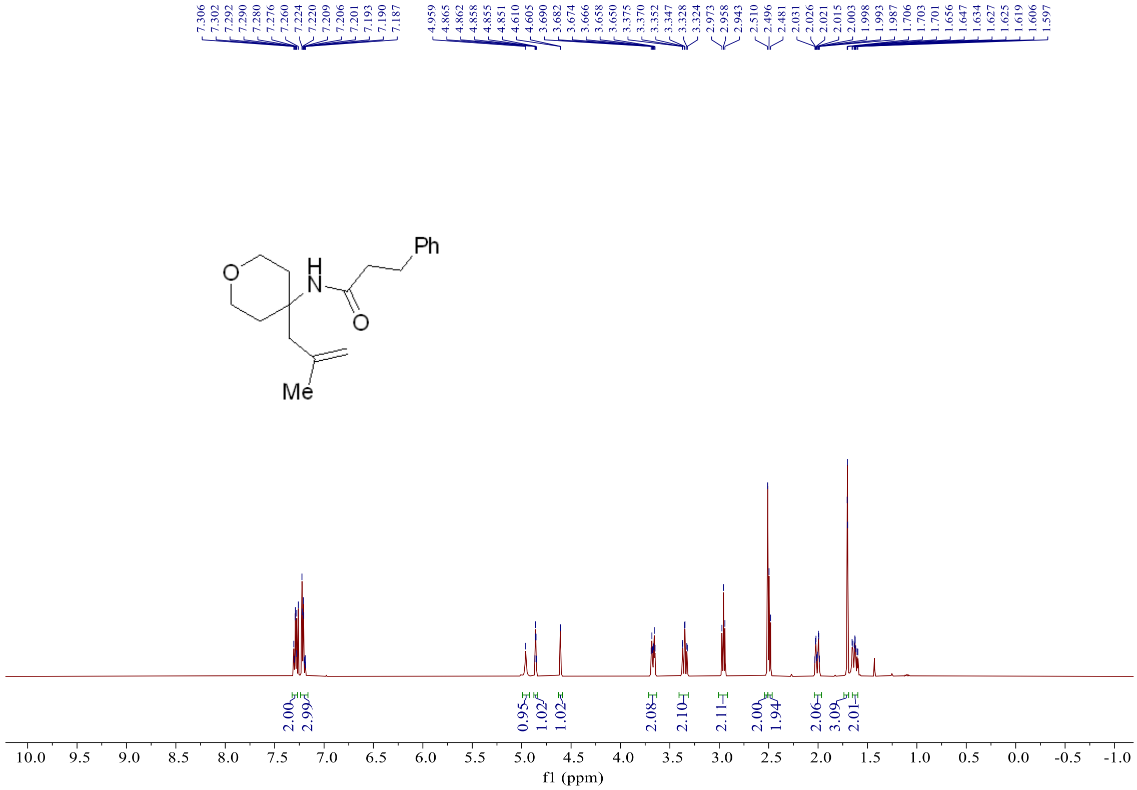

^{13}C NMR spectrum of **1g** (126 MHz, $CDCl_3$)

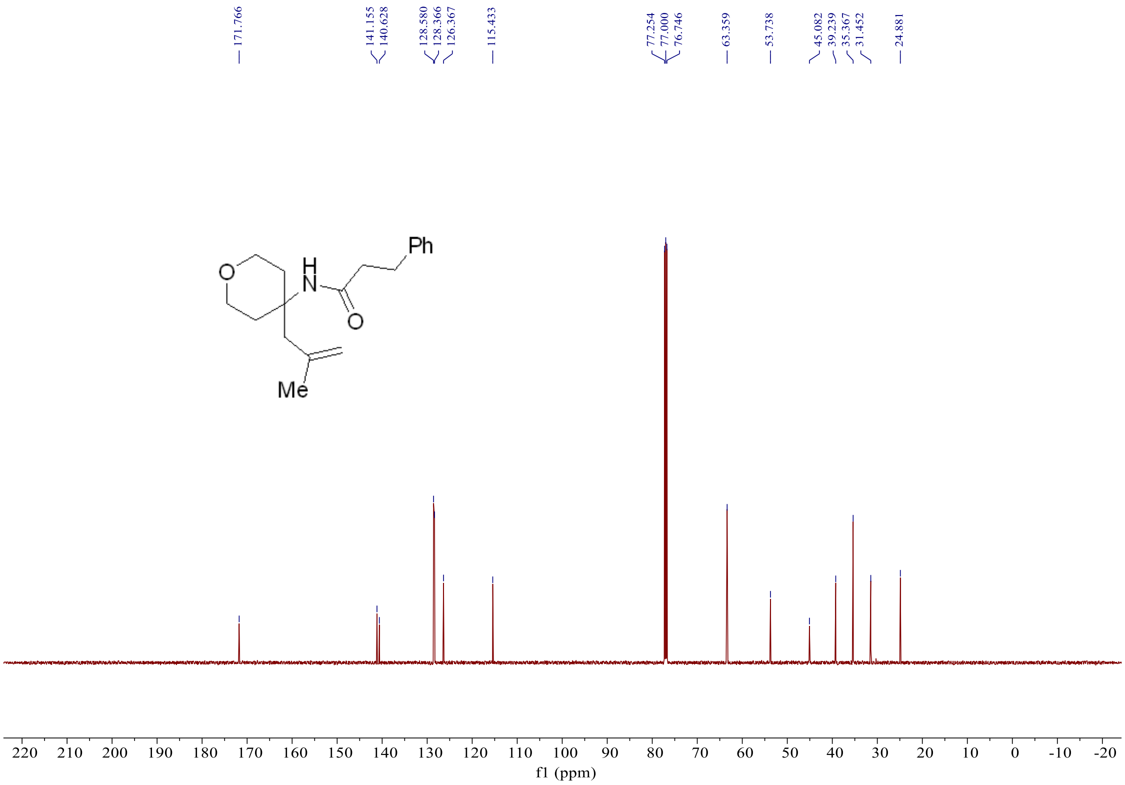

^{1}H NMR spectrum of **1t** (400 MHz, $CDCl_3$)

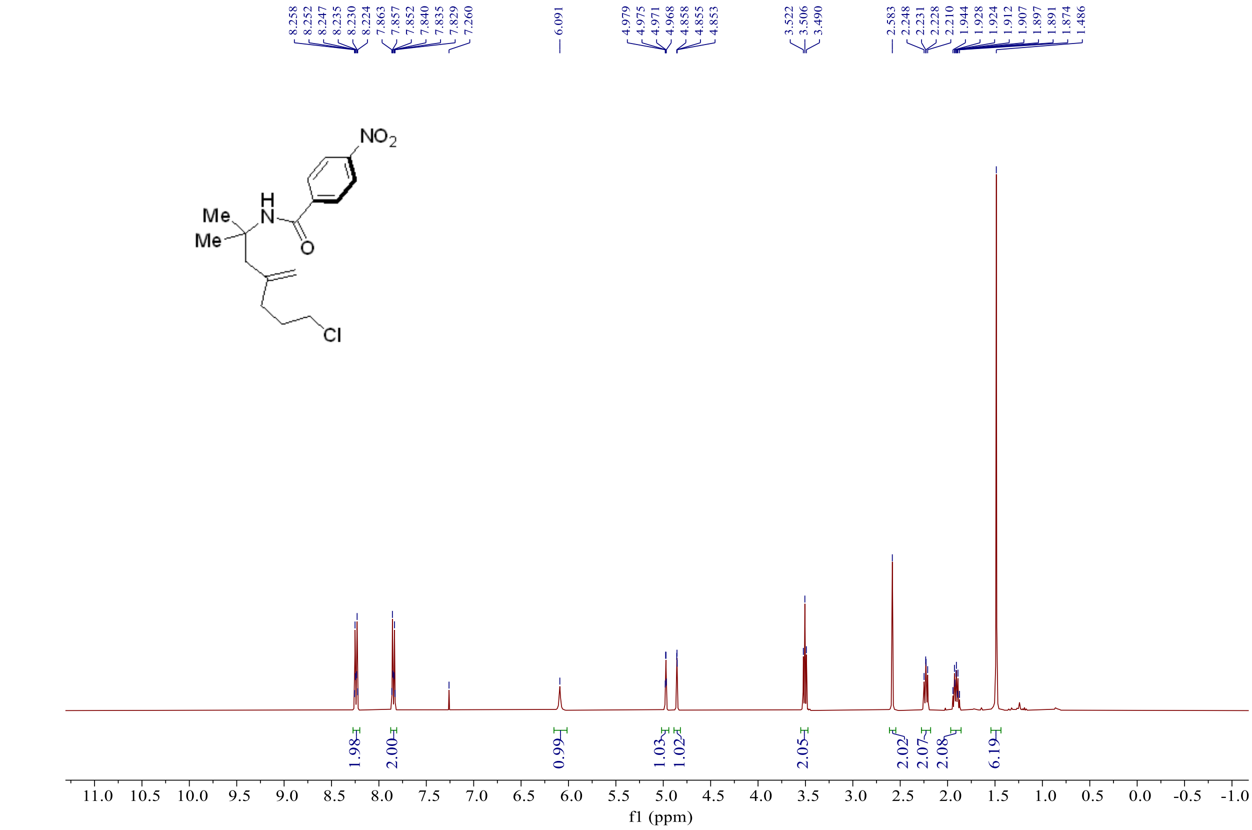

^{13}C NMR spectrum of **1t** (101 MHz, $CDCl_3$)

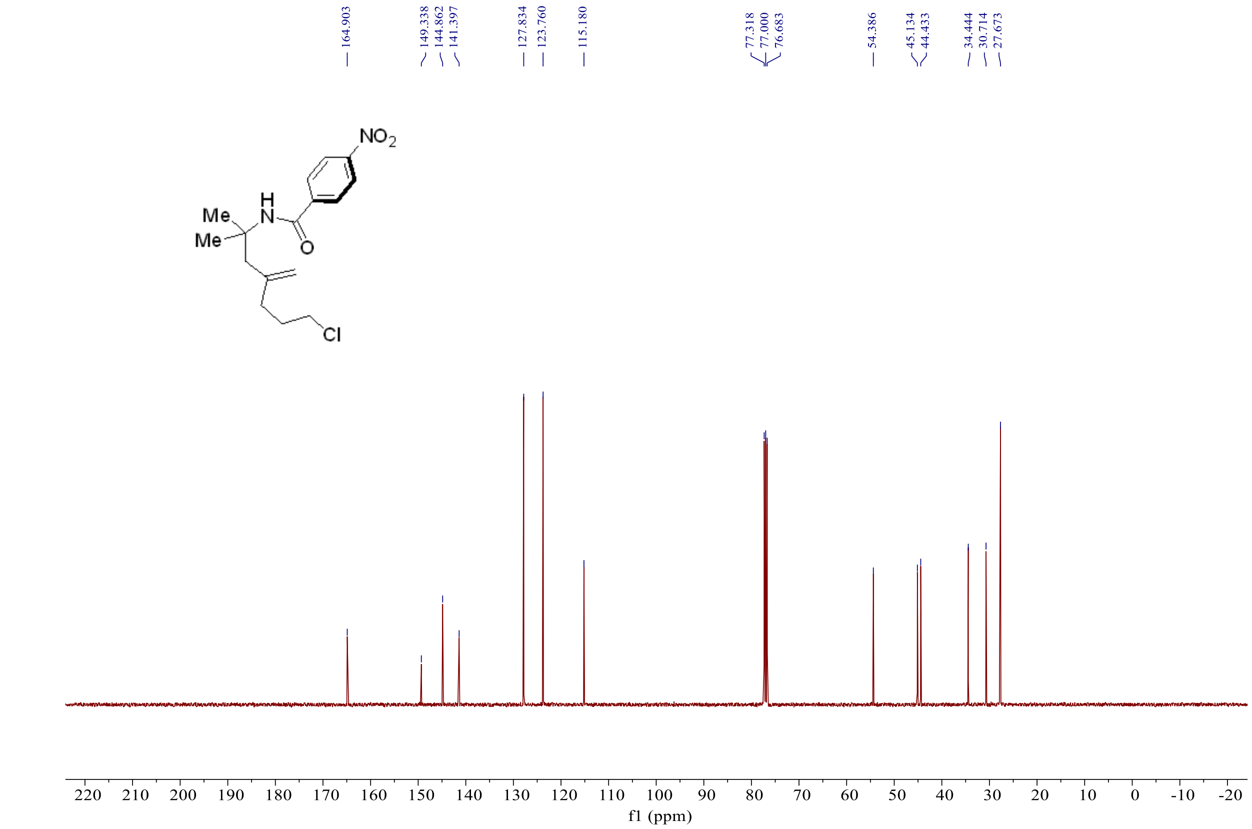

^{1}H NMR spectrum of **1u** (400 MHz, $CDCl_3$)

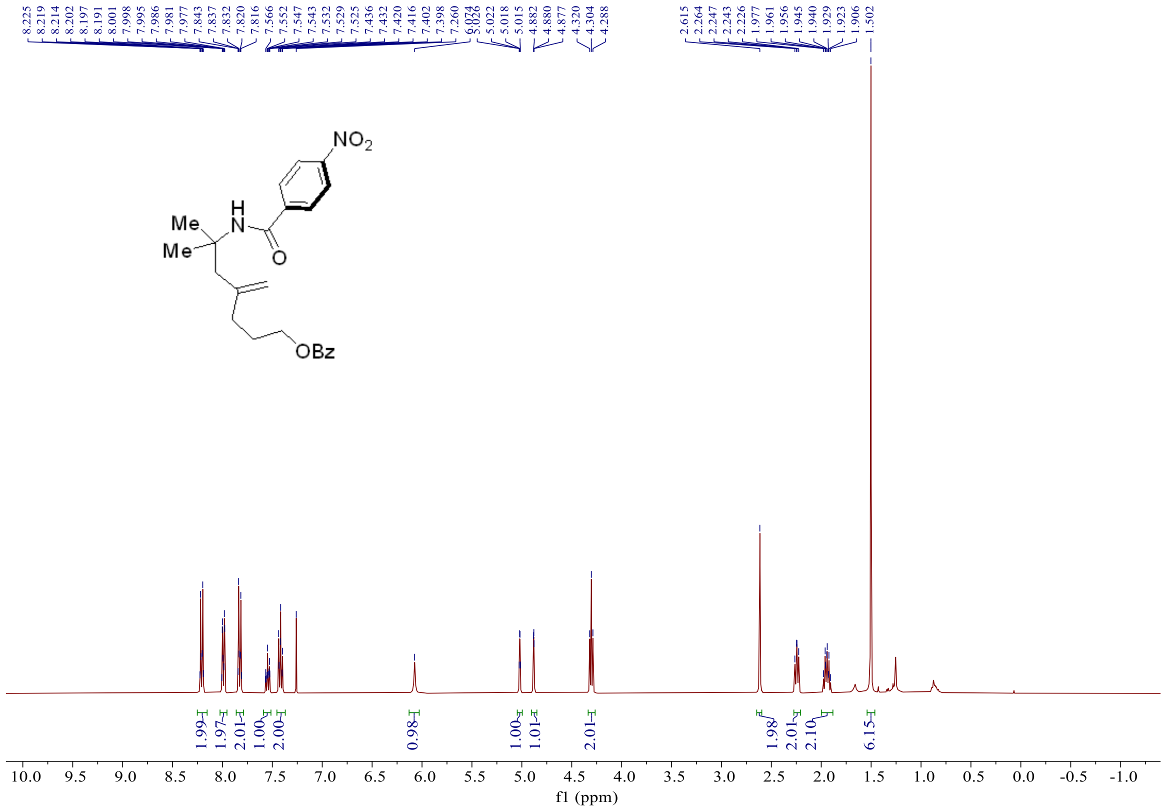

^{13}C NMR spectrum of **1u** (101 MHz, $CDCl_3$)

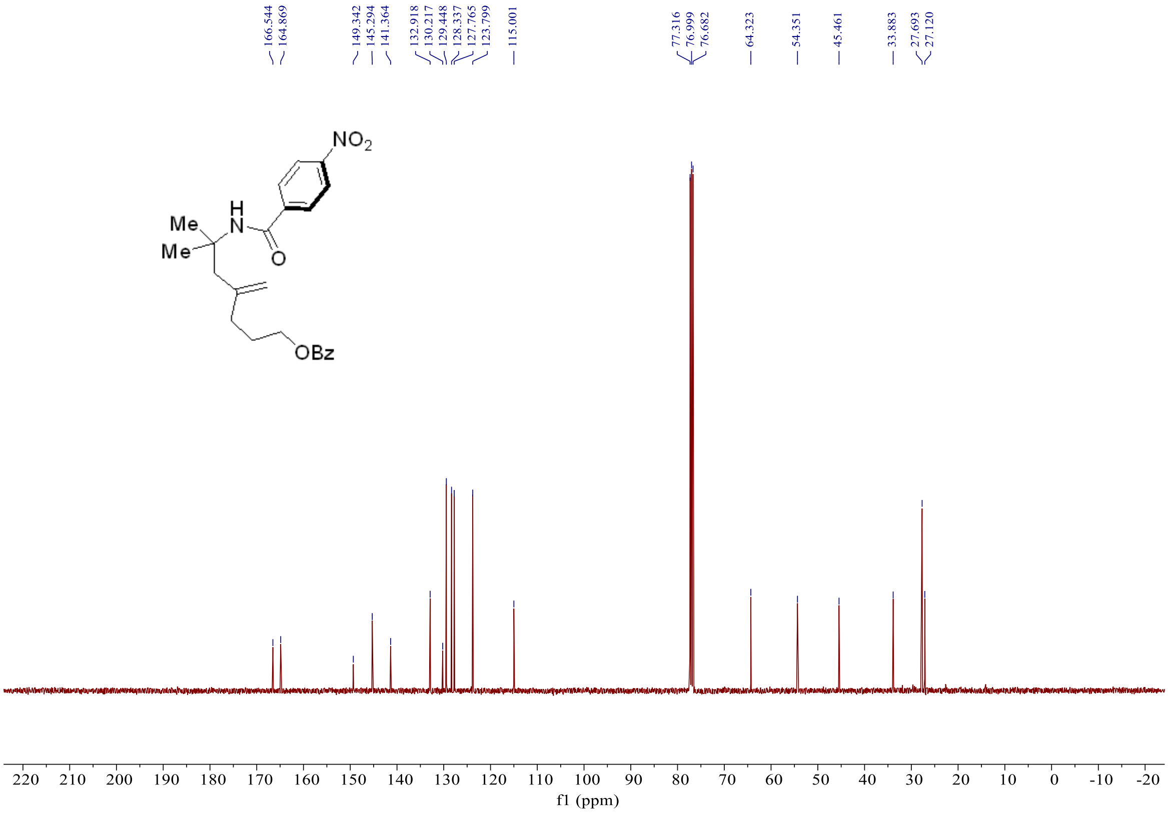

^{1}H NMR spectrum of **1v** (400 MHz, $CDCl_3$)

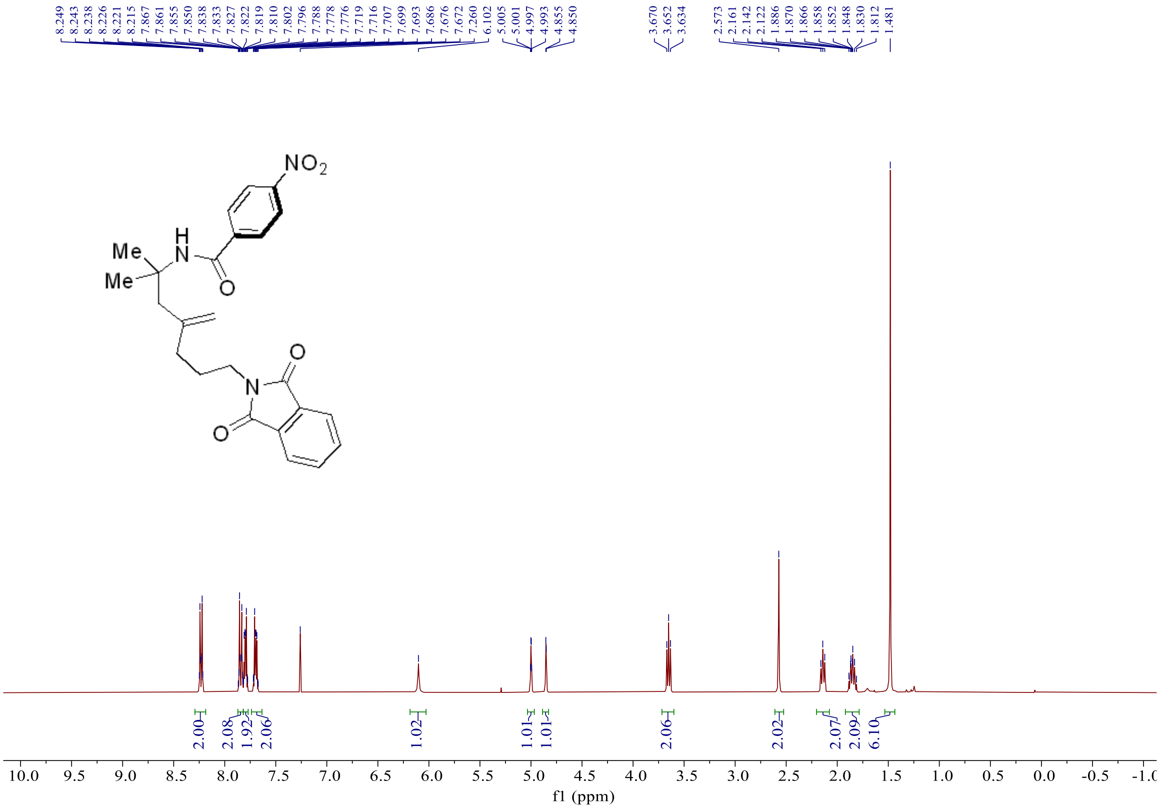

^{13}C NMR spectrum of **1v** (101 MHz, $CDCl_3$)

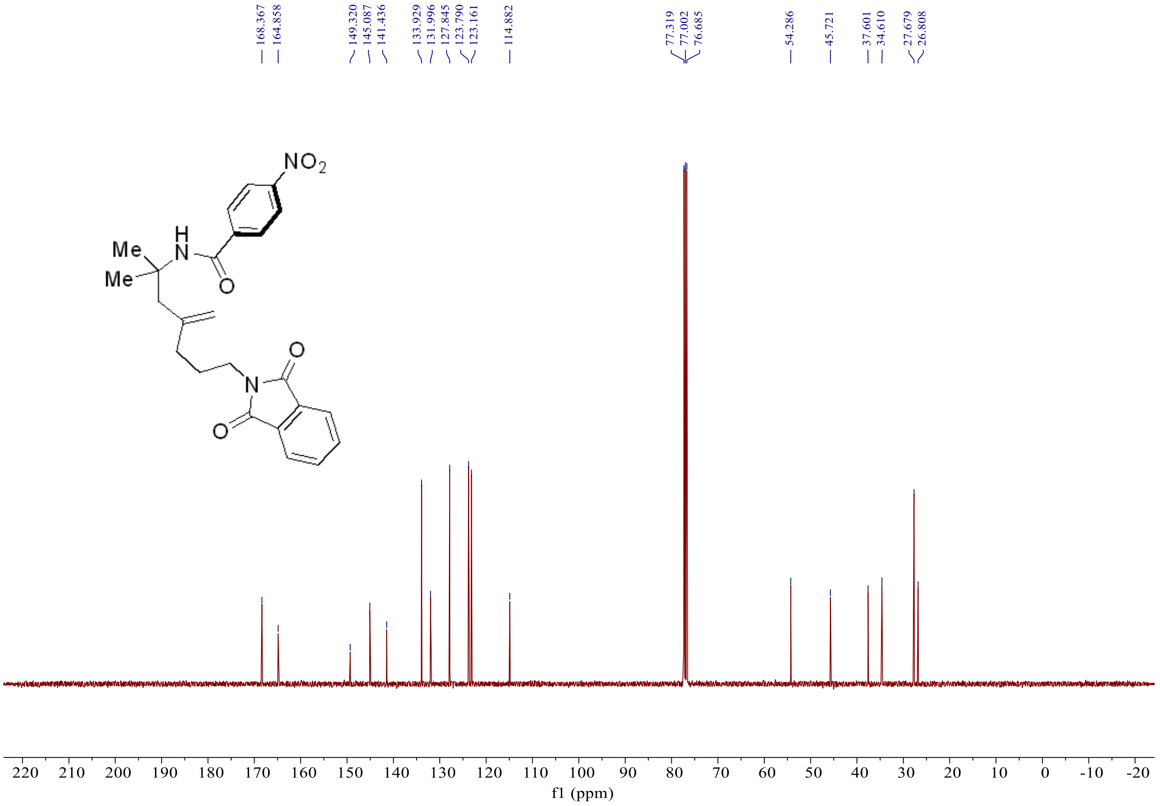

^{1}H NMR spectrum of **1w** (500 MHz, $CDCl_3$)

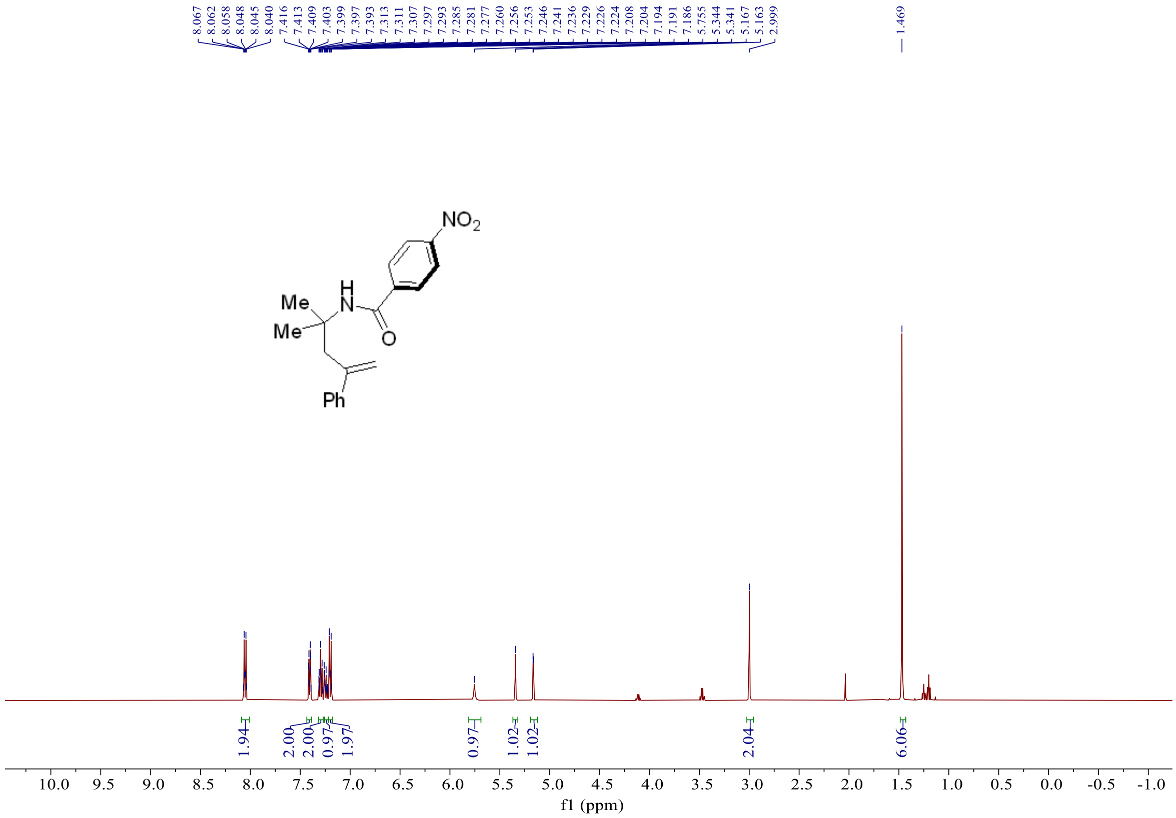

^{13}C NMR spectrum of **1w** (126 MHz, $CDCl_3$)

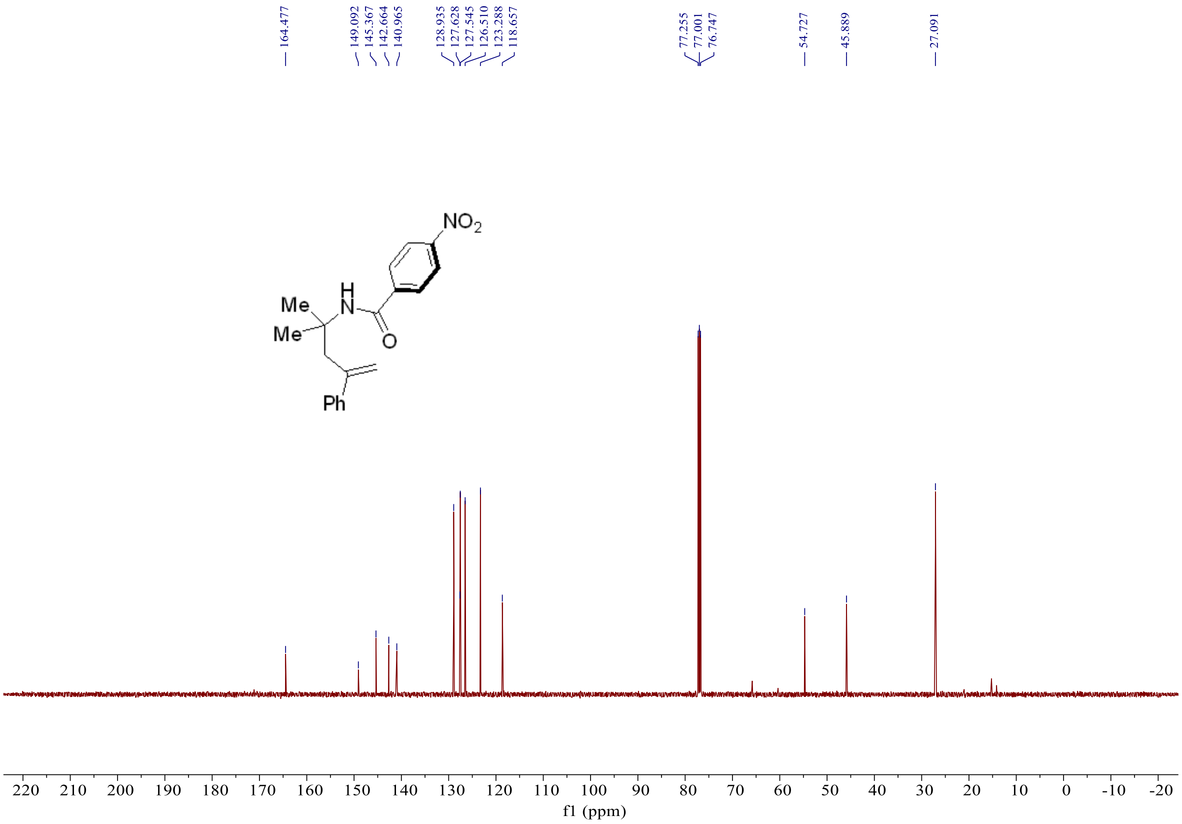

^{1}H NMR spectrum of **1ak** (400 MHz, $CDCl_3$)

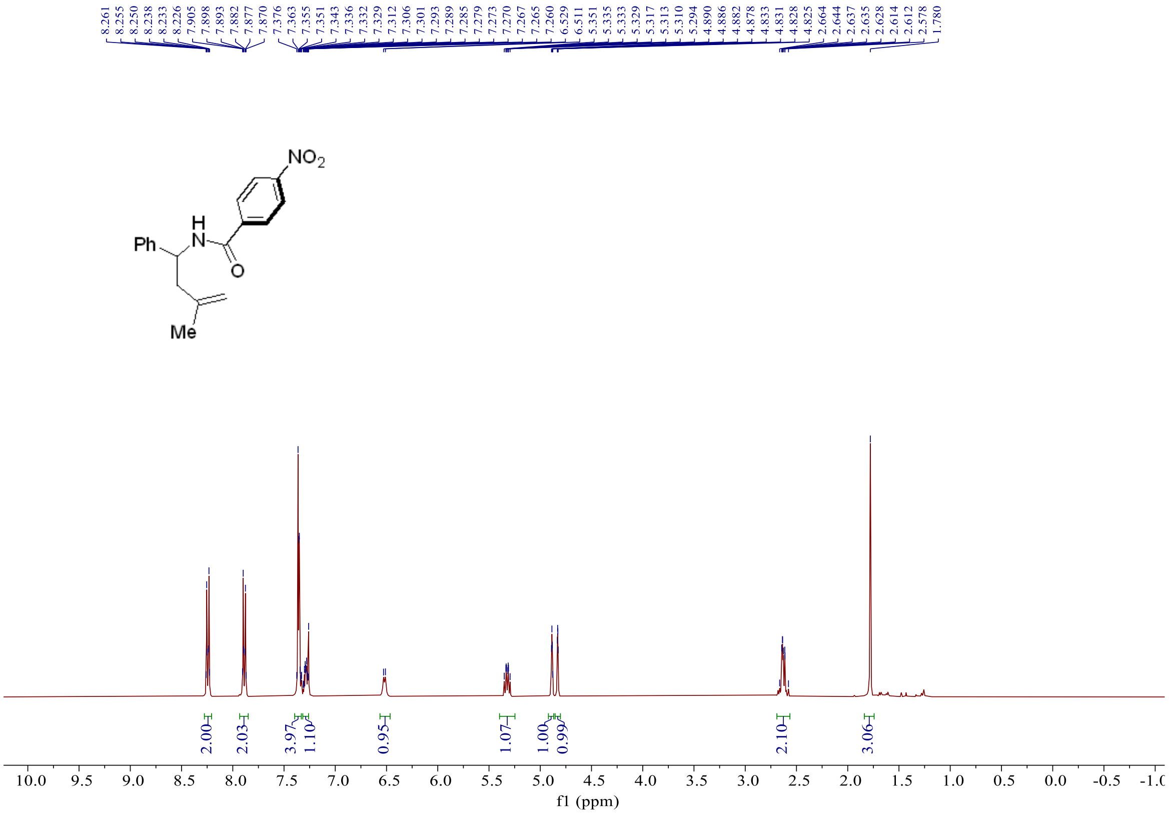

^{13}C NMR spectrum of **1ak** (101 MHz, $CDCl_3$)

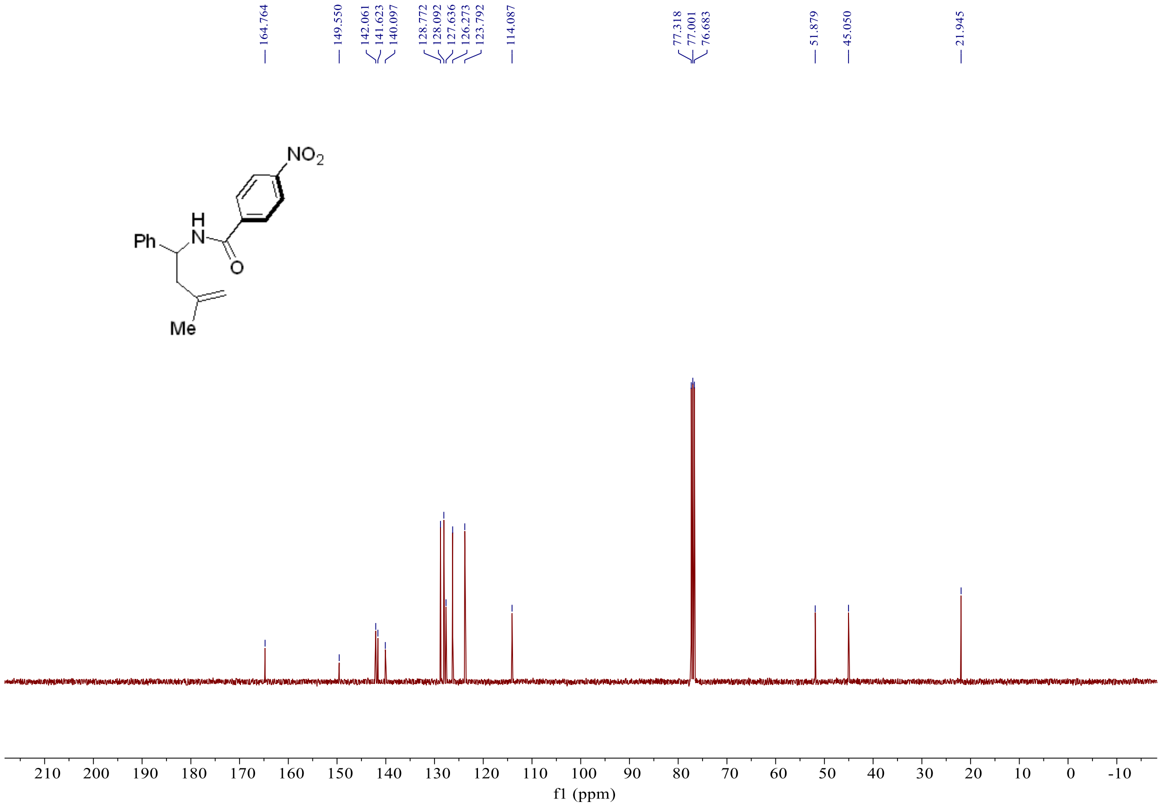

^{1}H NMR spectrum of **2a** (600 MHz, $CDCl_3$)

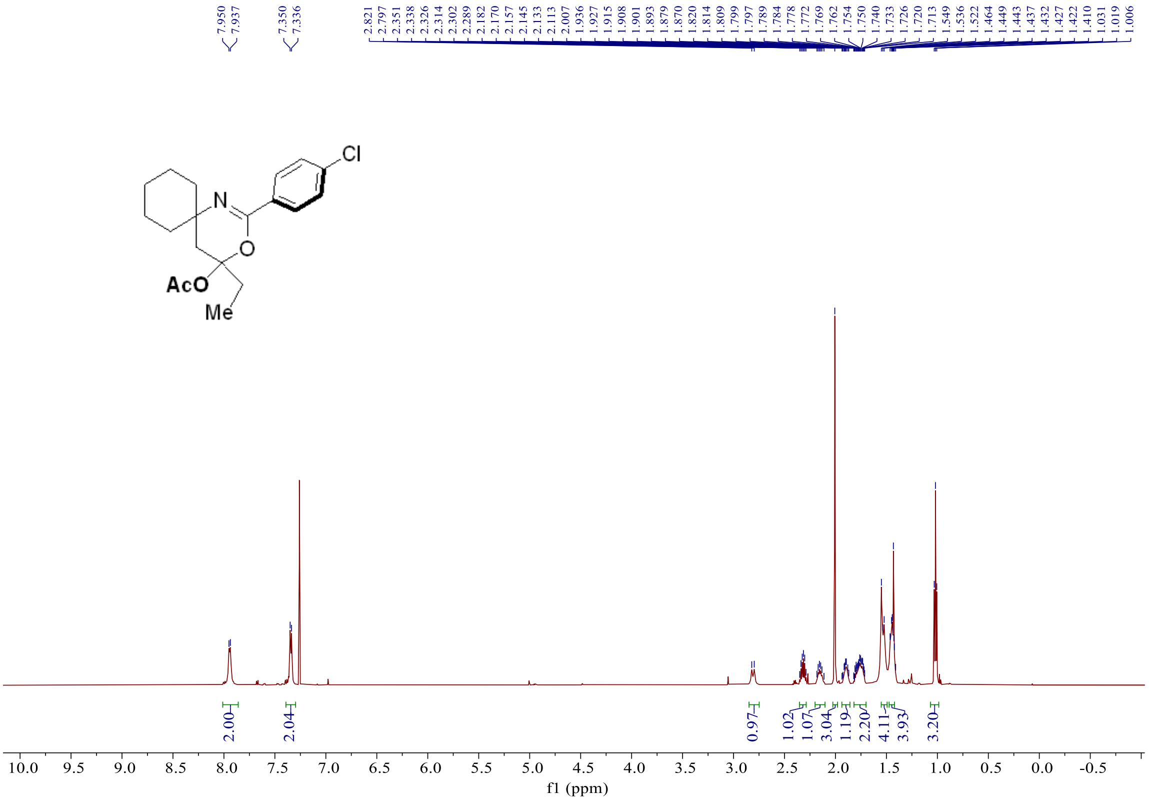

^{13}C NMR spectrum of **2a** (151 MHz, $CDCl_3$)

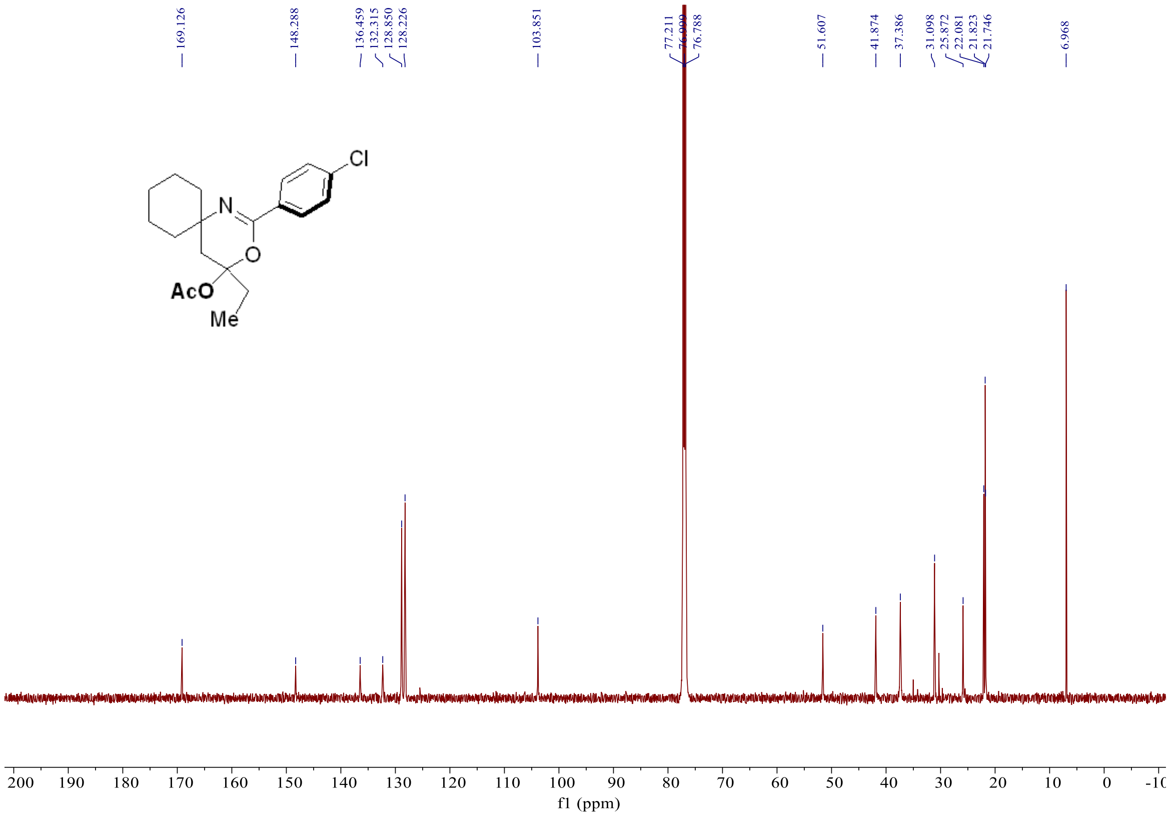

^{1}H NMR spectrum of **2b** (600 MHz, $CDCl_3$)

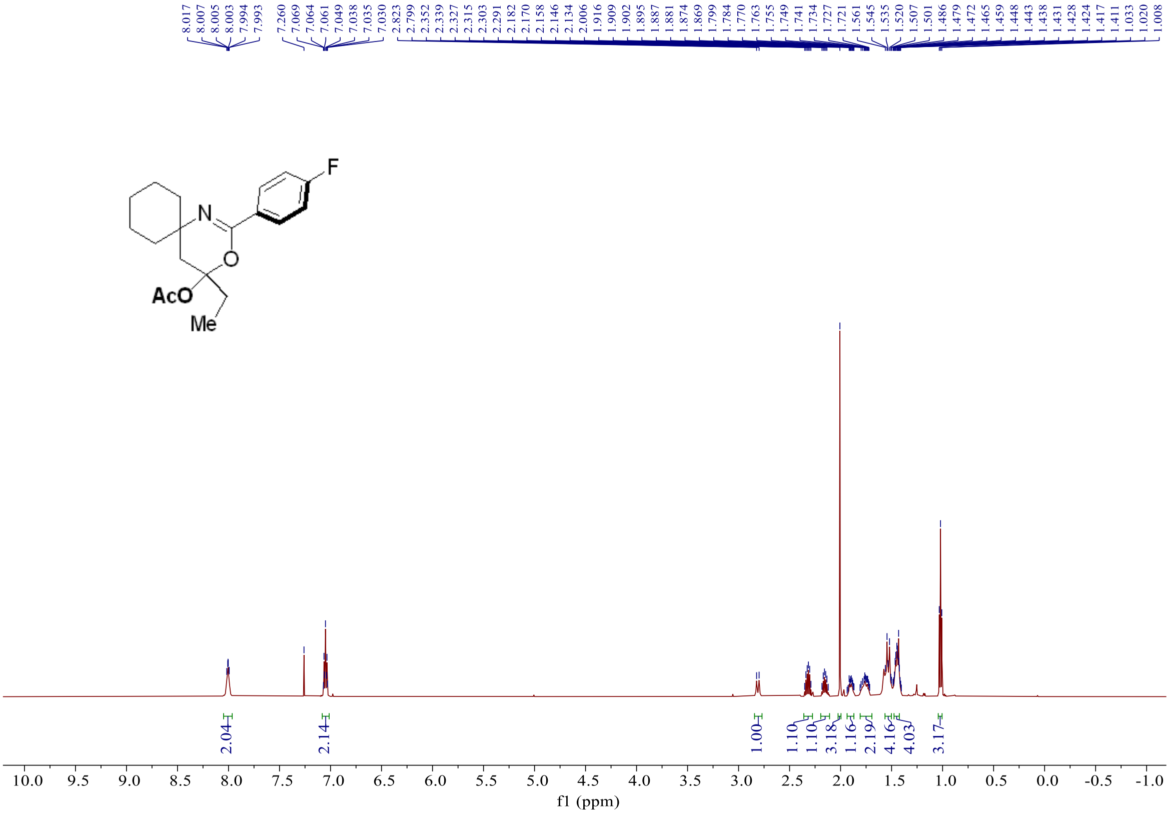

^{13}C NMR spectrum of **2b** (151 MHz, $CDCl_3$)

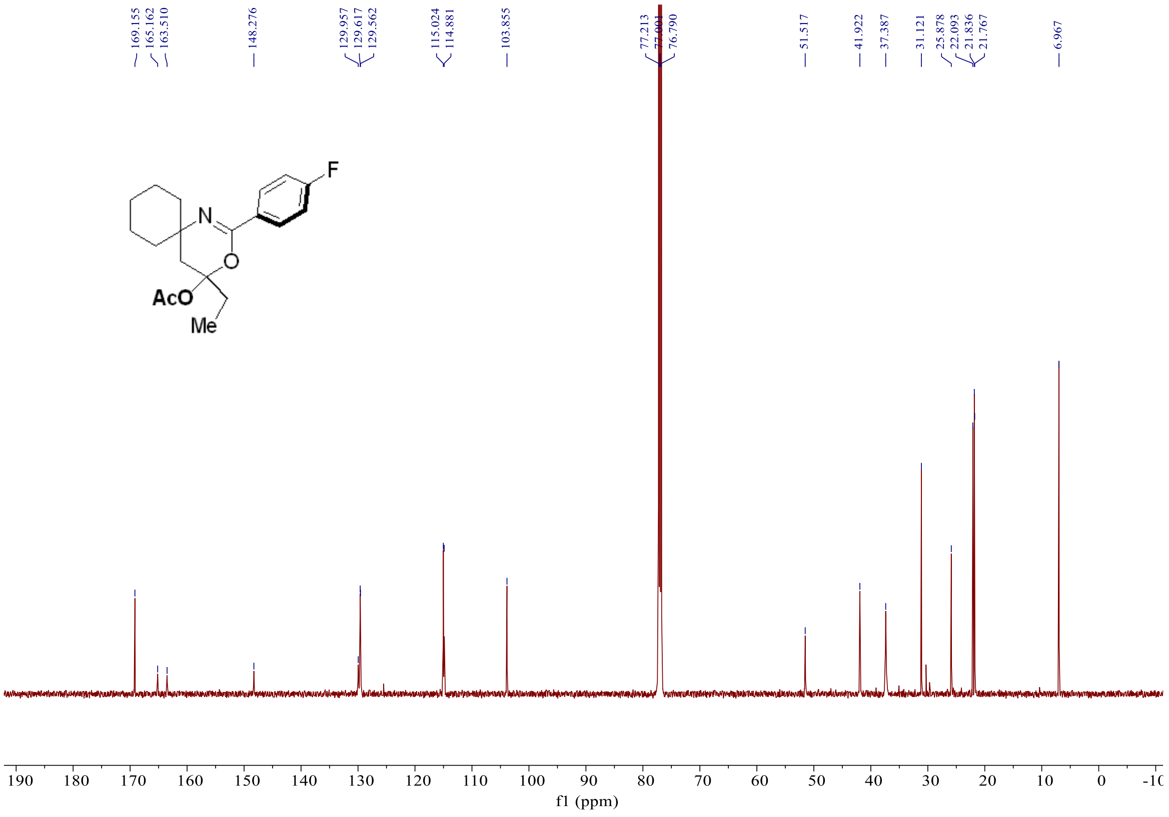

^{19}F NMR spectrum of **2b** (377 MHz, $CDCl_3$)

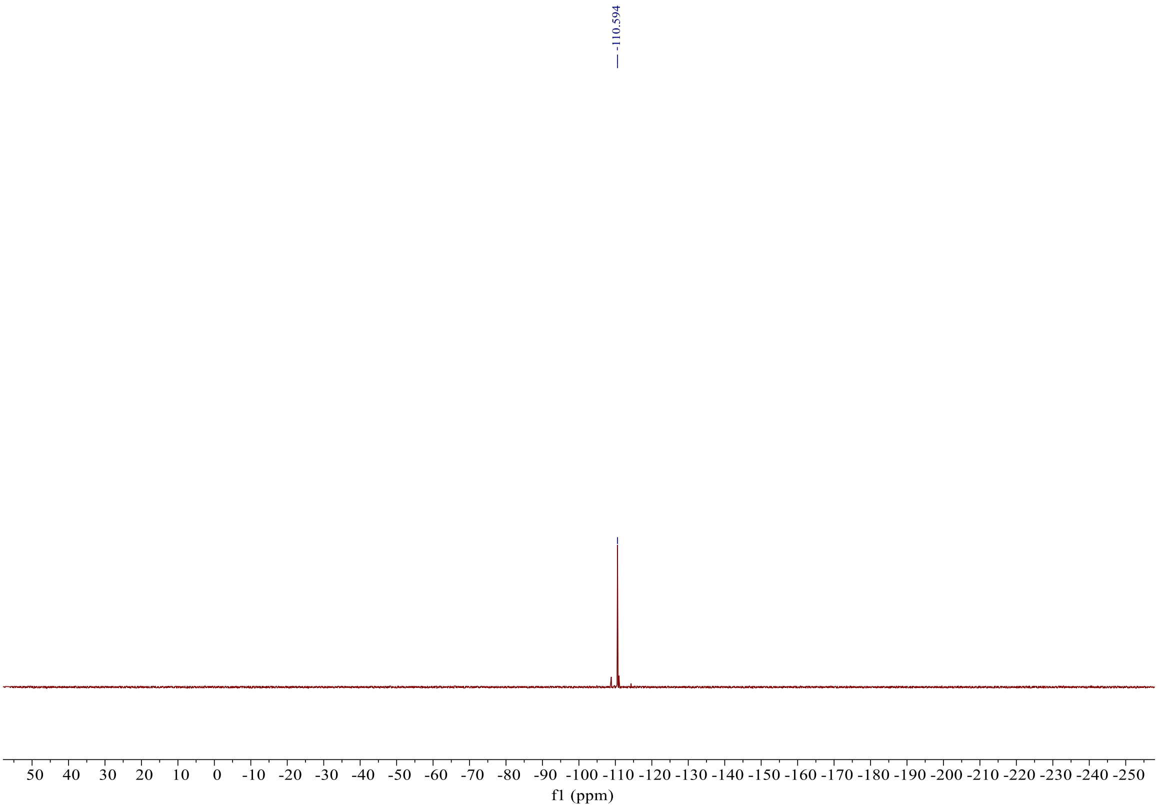

^{1}H NMR spectrum of **2c** (500 MHz, $CDCl_3$)

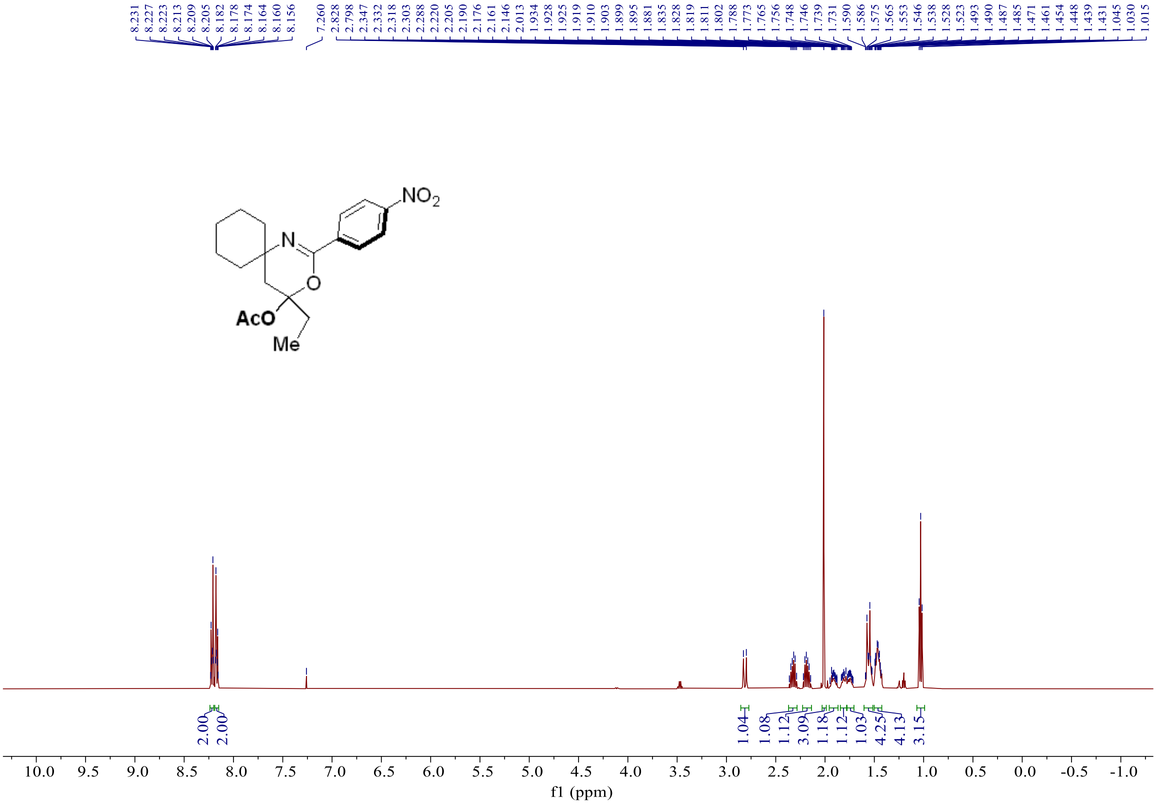

^{13}C NMR spectrum of **2c** (126 MHz, $CDCl_3$)

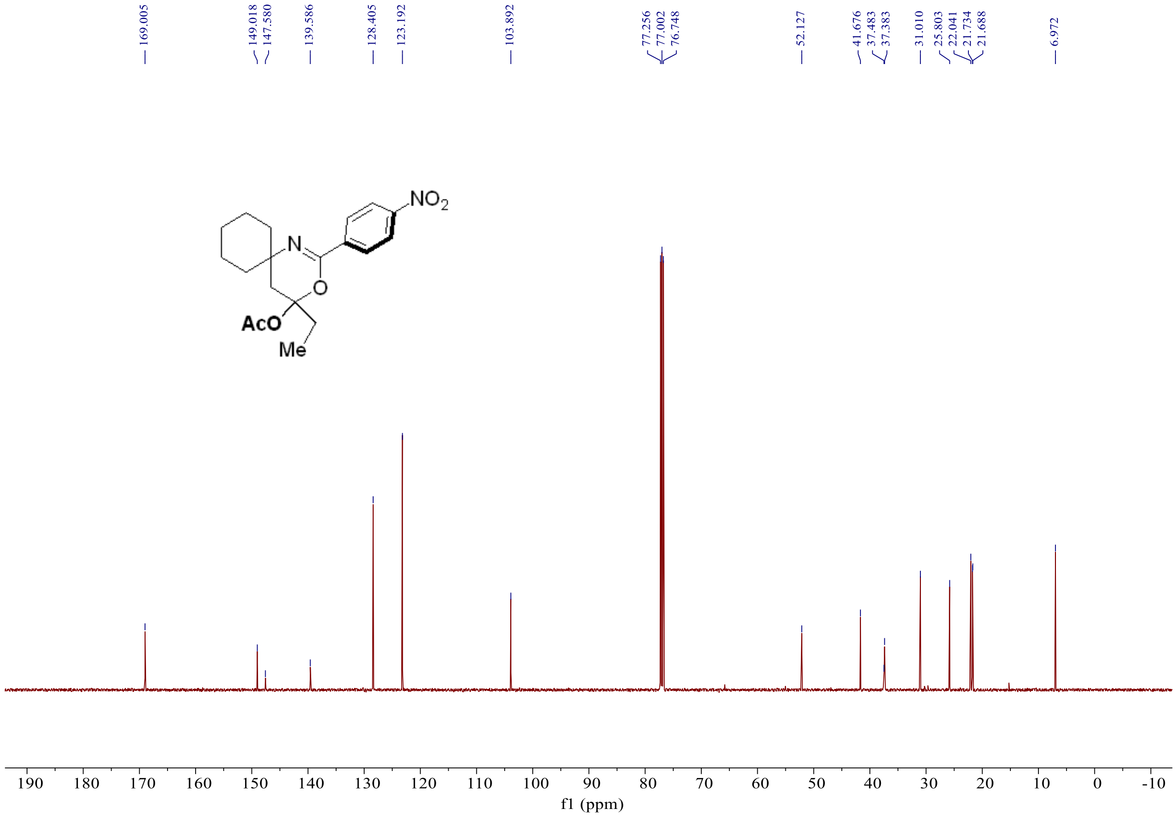

^{1}H NMR spectrum of **2d** (600 MHz, $CDCl_3$)

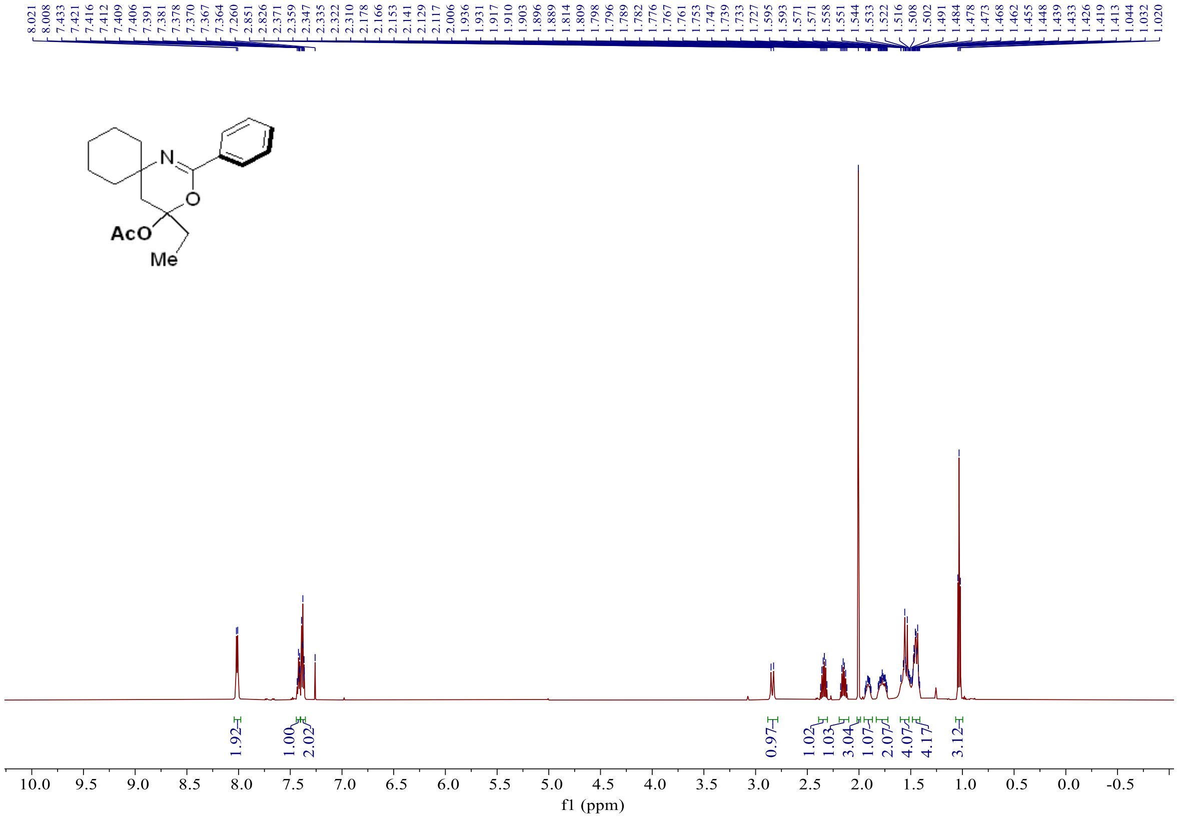

^{13}C NMR spectrum of **2d** (151 MHz, $CDCl_3$)

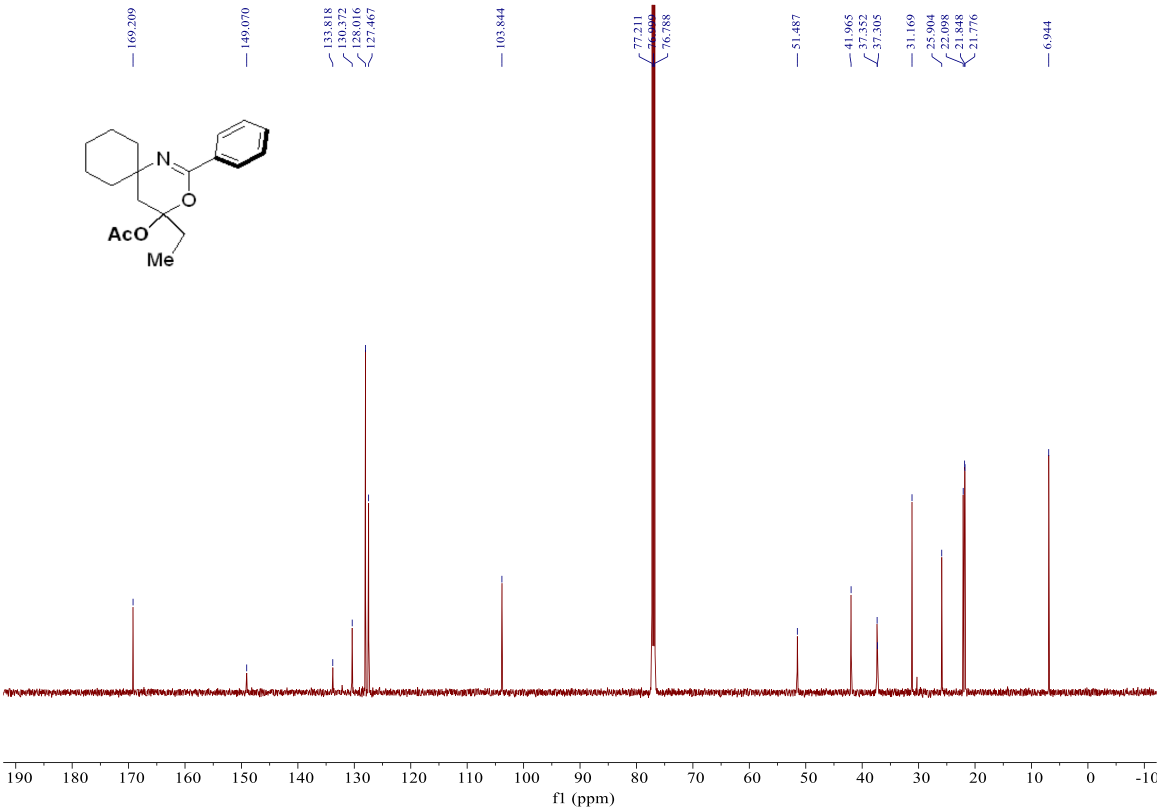

^{1}H NMR spectrum of **2e** (500 MHz, $CDCl_3$)

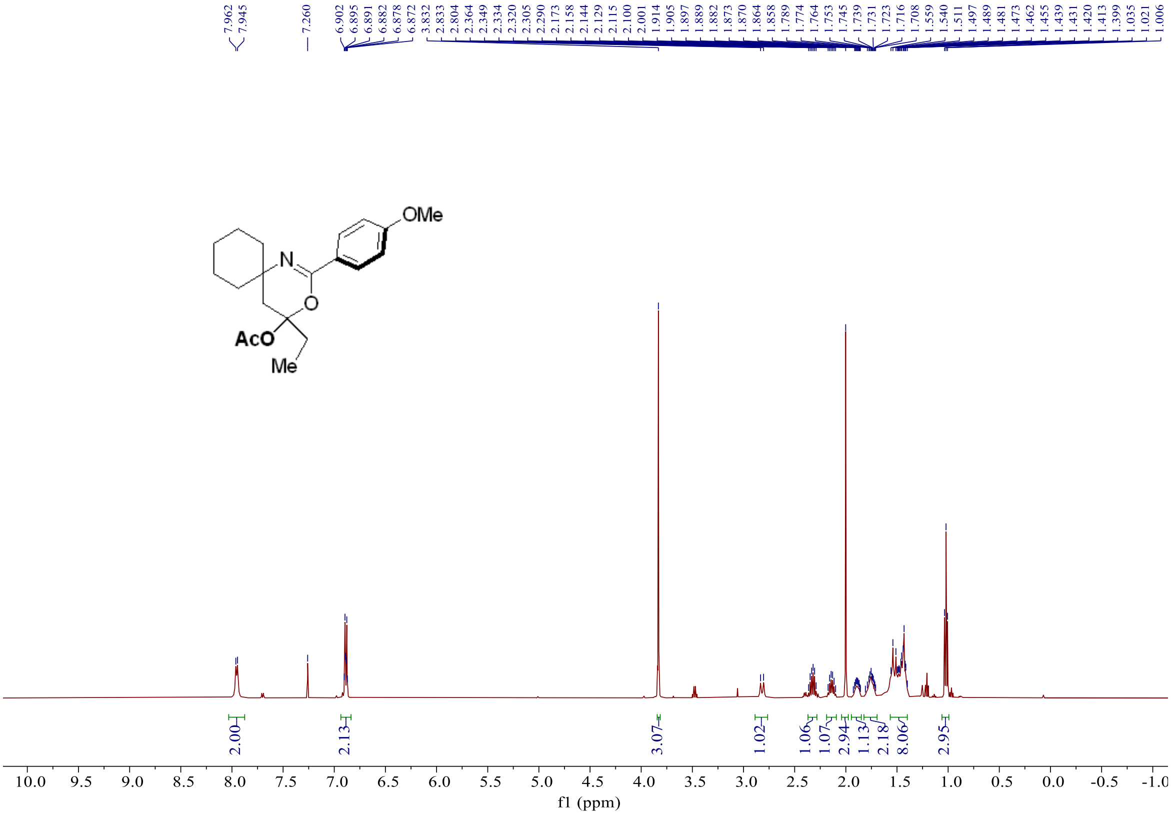

^{13}C NMR spectrum of **2e** (126 MHz, $CDCl_3$)

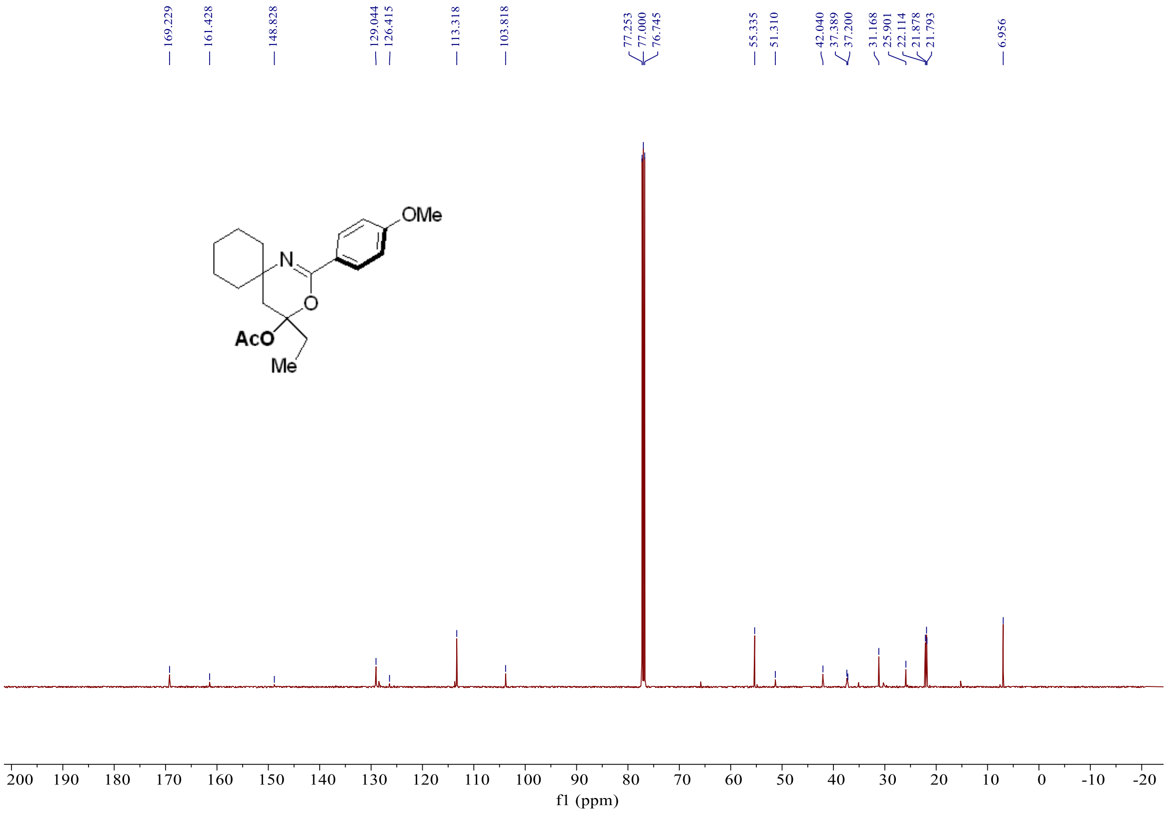

^{1}H NMR spectrum of **2f** (500 MHz, $CDCl_3$)

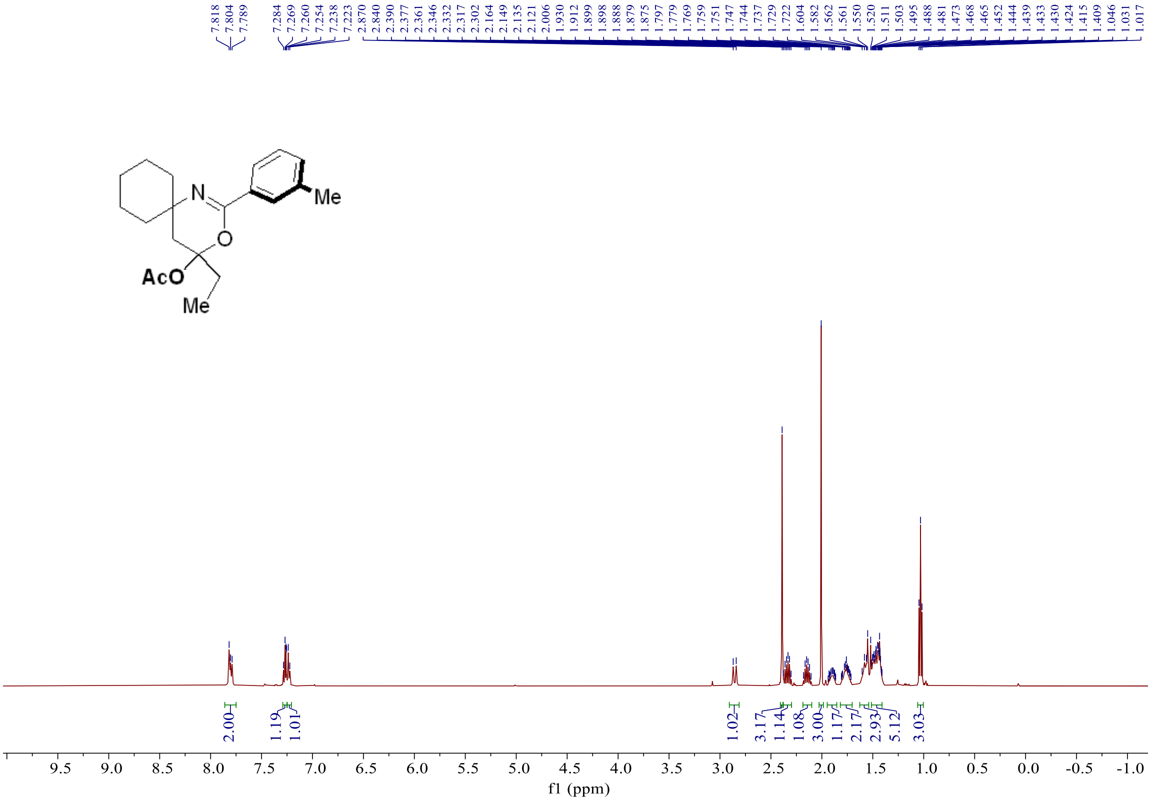

^{13}C NMR spectrum of **2f** (126 MHz, $CDCl_3$)

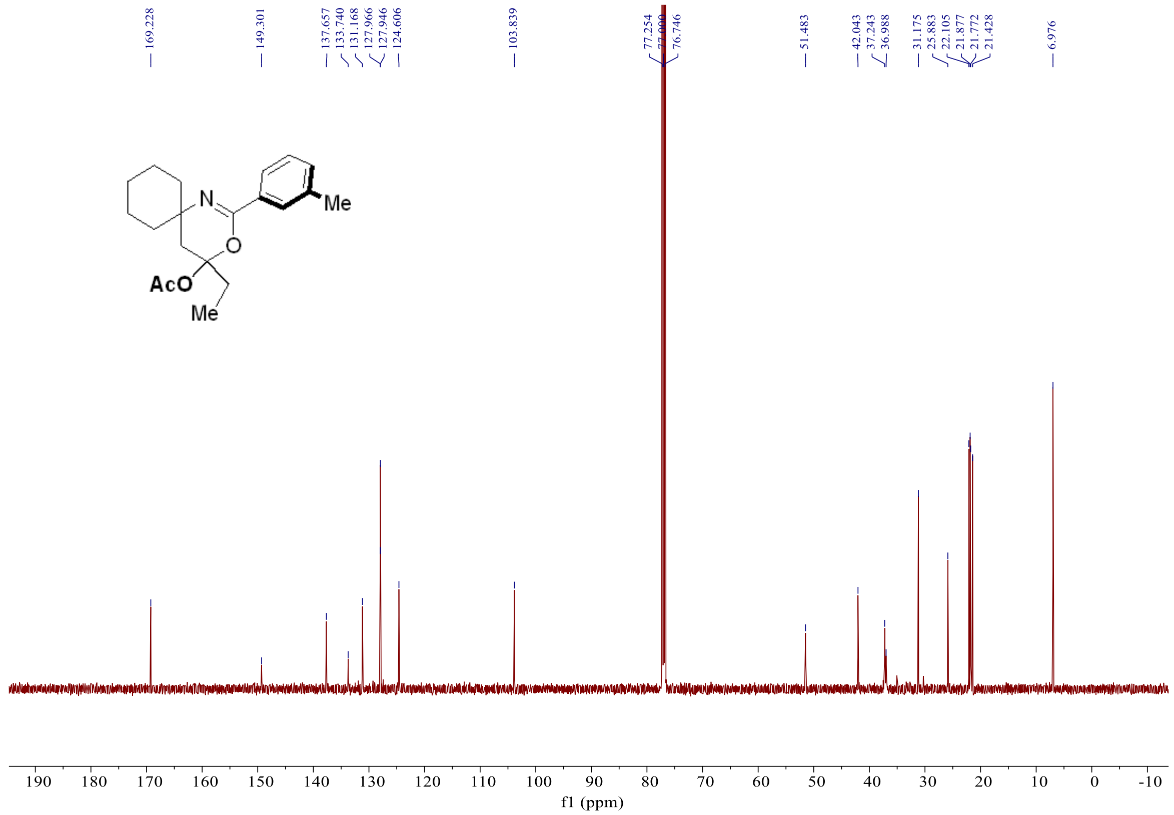

^{1}H NMR spectrum of **2g** (600 MHz, $CDCl_3$)

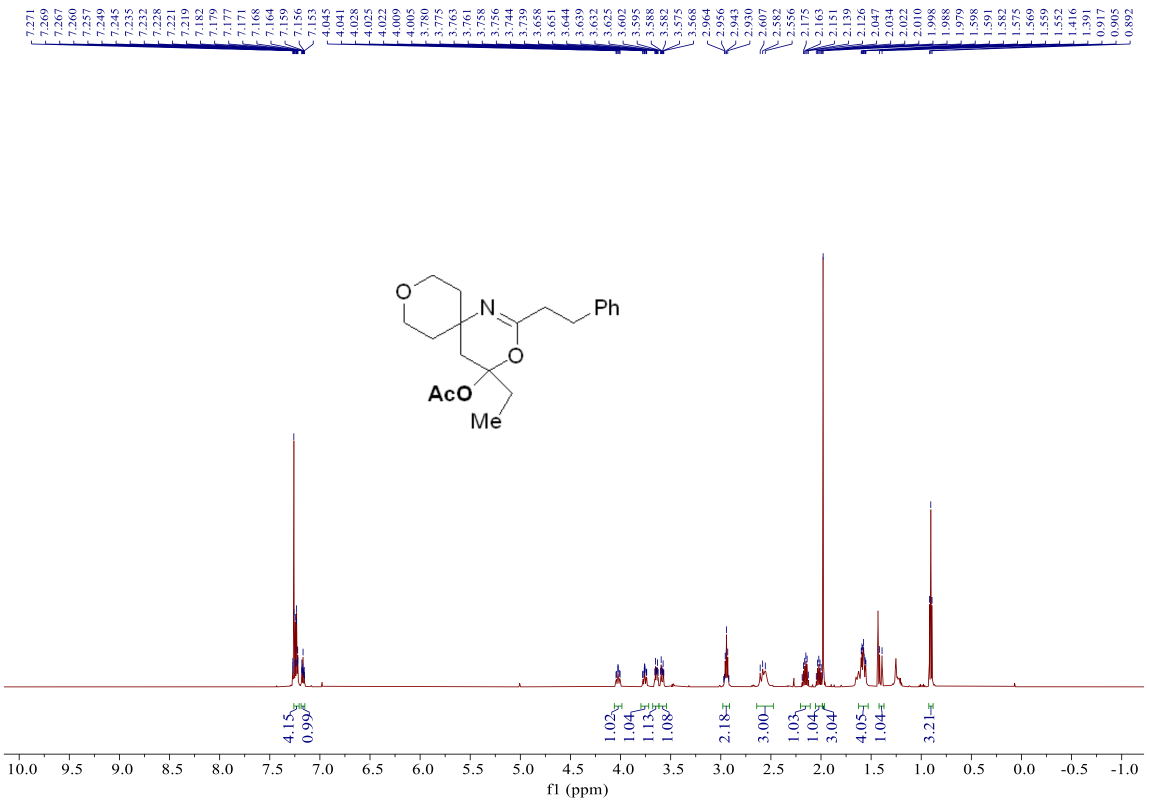

^{13}C NMR spectrum of **2g** (151 MHz, $CDCl_3$)

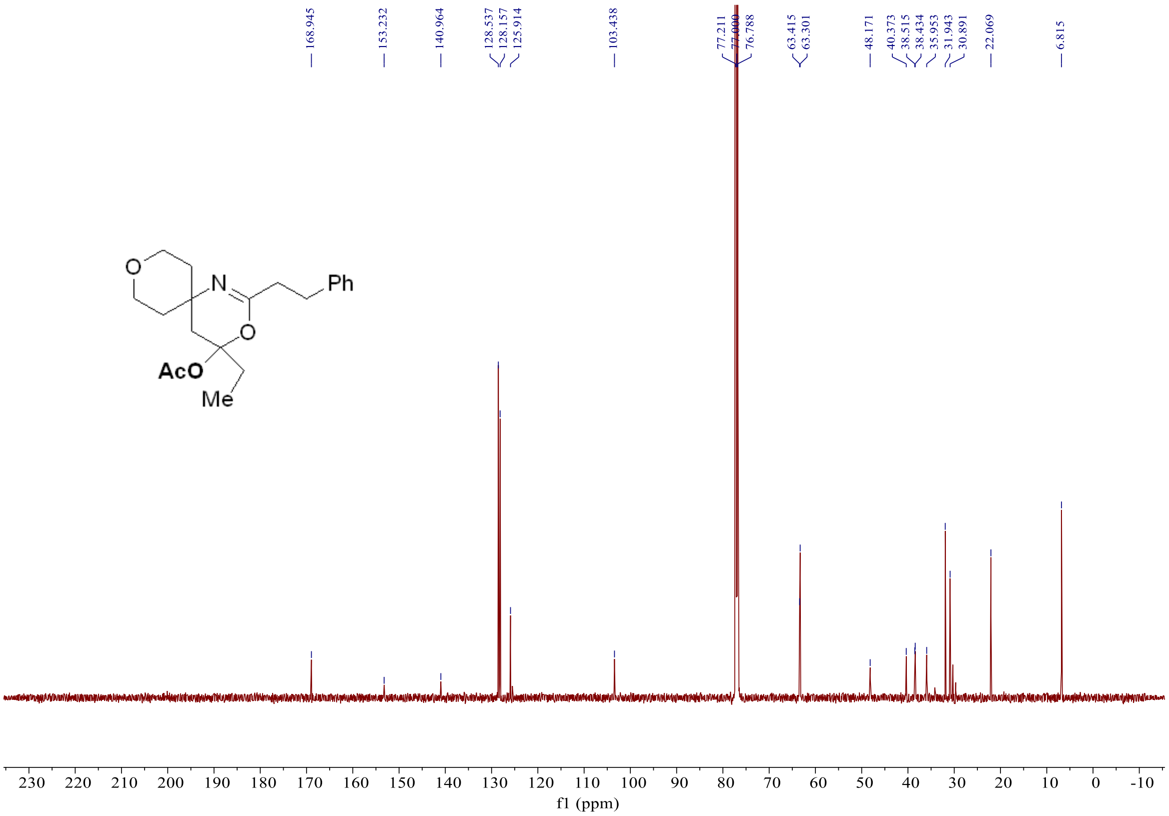

^{1}H NMR spectrum of **2h** (500 MHz, $CDCl_3$)

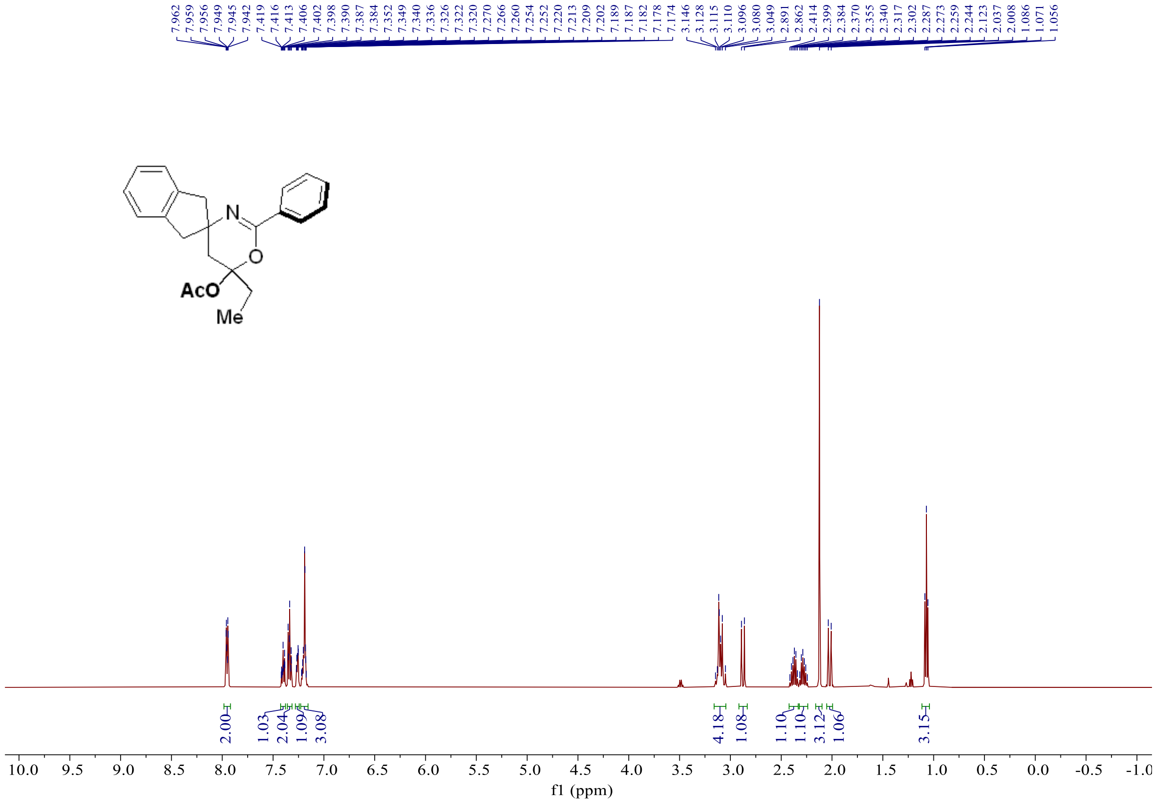

^{13}C NMR spectrum of **2h** (126 MHz, $CDCl_3$)

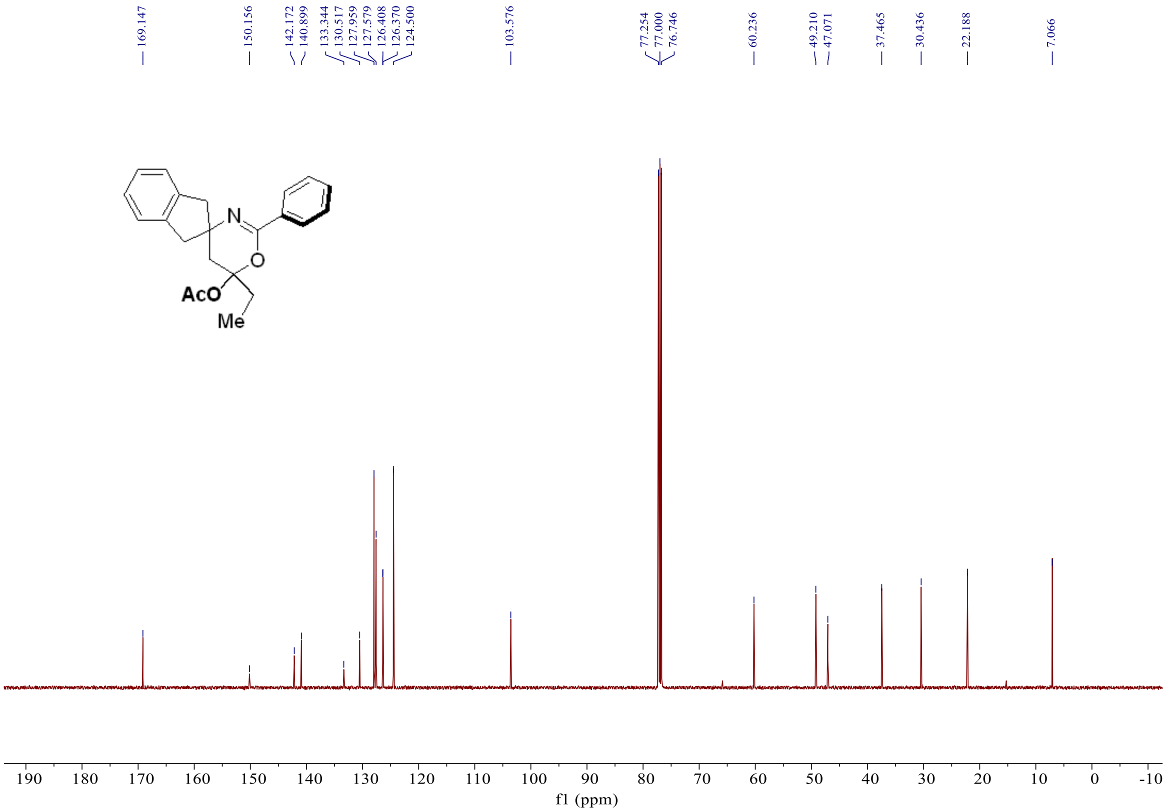

^{1}H NMR spectrum of **2i** (800 MHz, $CDCl_3$)

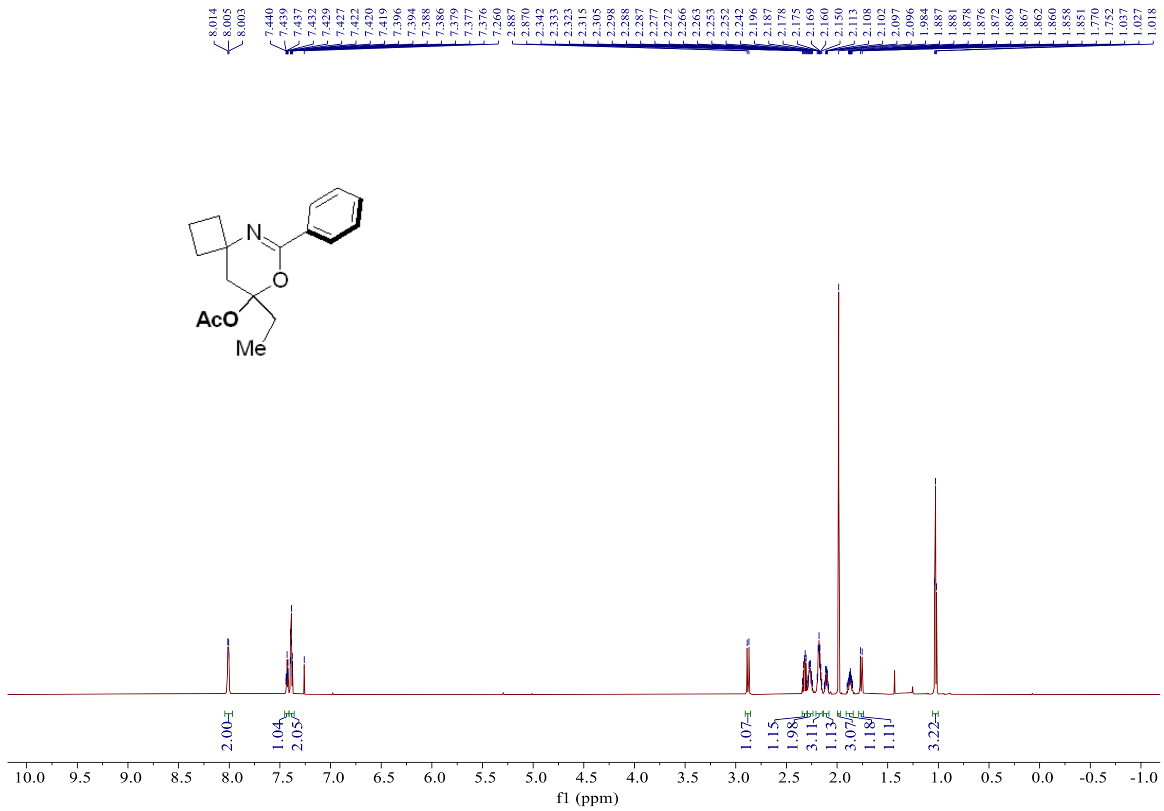

^{13}C NMR spectrum of **2i** (201 MHz, $CDCl_3$)

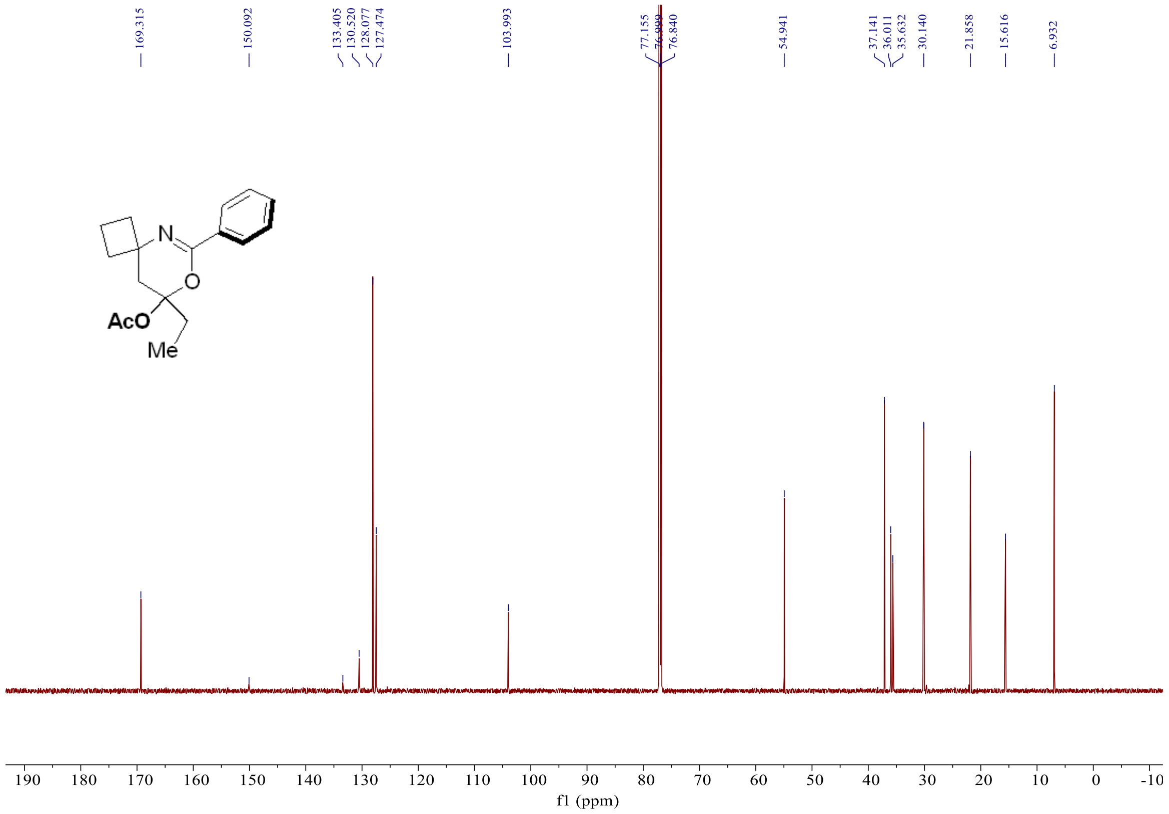

^{1}H NMR spectrum of **2j** (600 MHz, $CDCl_3$)

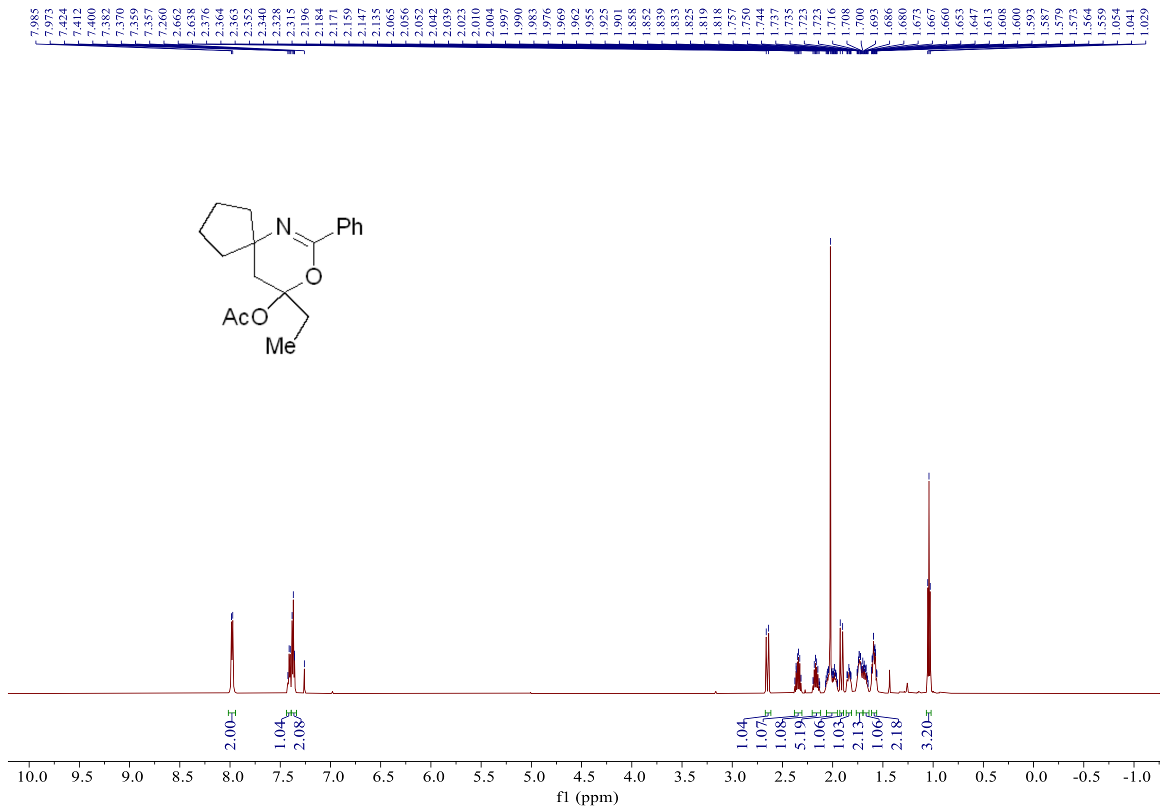

^{13}C NMR spectrum of **2j** (151 MHz, CDCl$_3$)

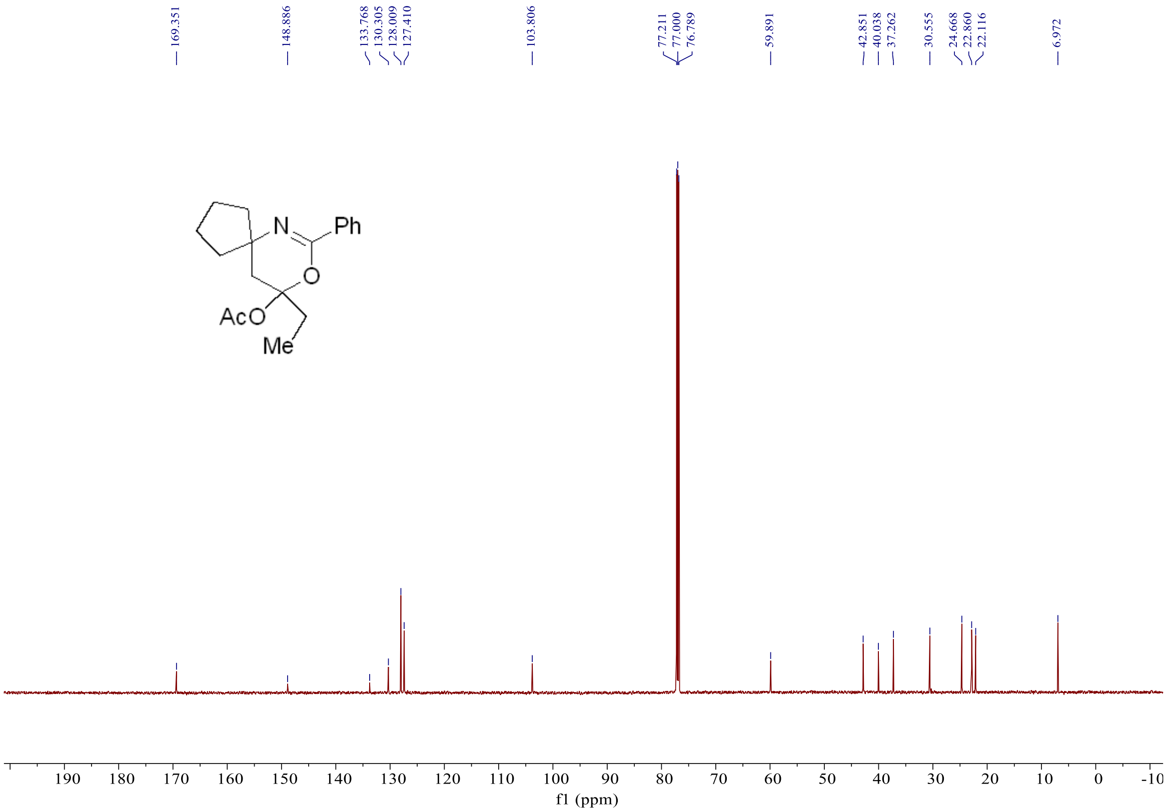

^{1}H NMR spectrum of **2k** (400 MHz, CDCl$_3$)

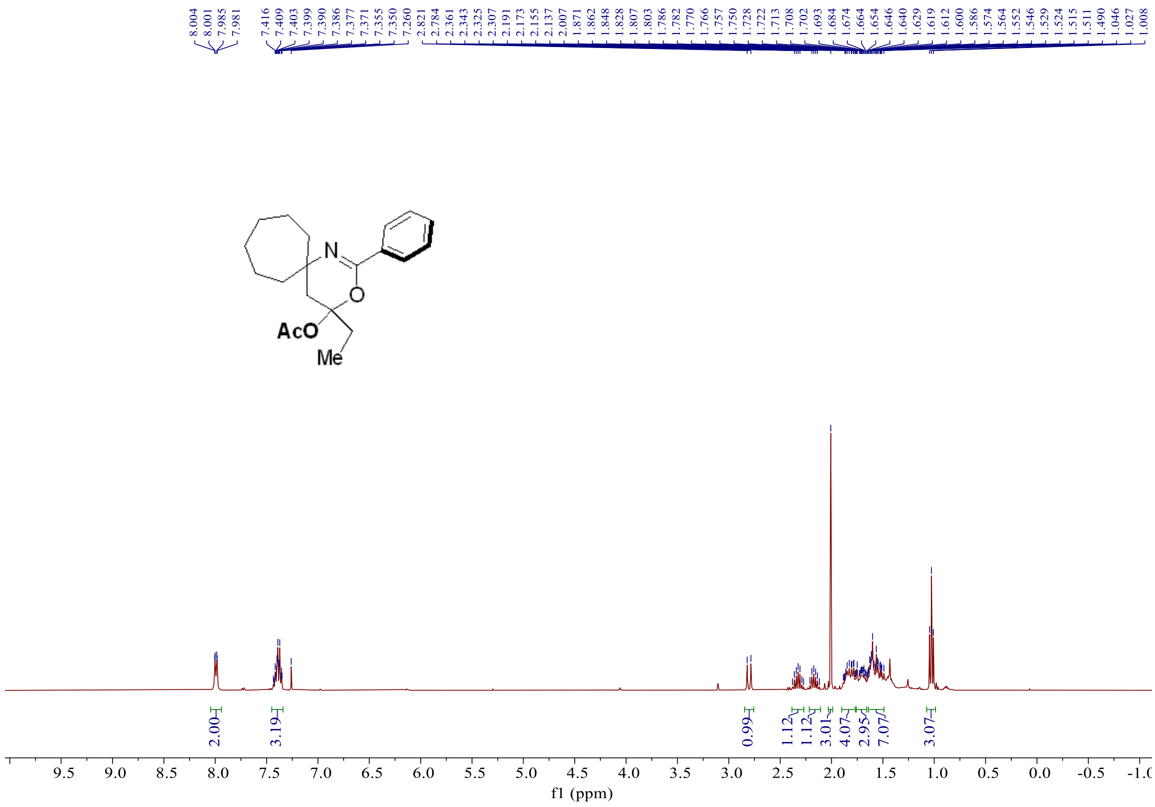

^{13}C NMR spectrum of **2k** (151 MHz, $CDCl_3$)

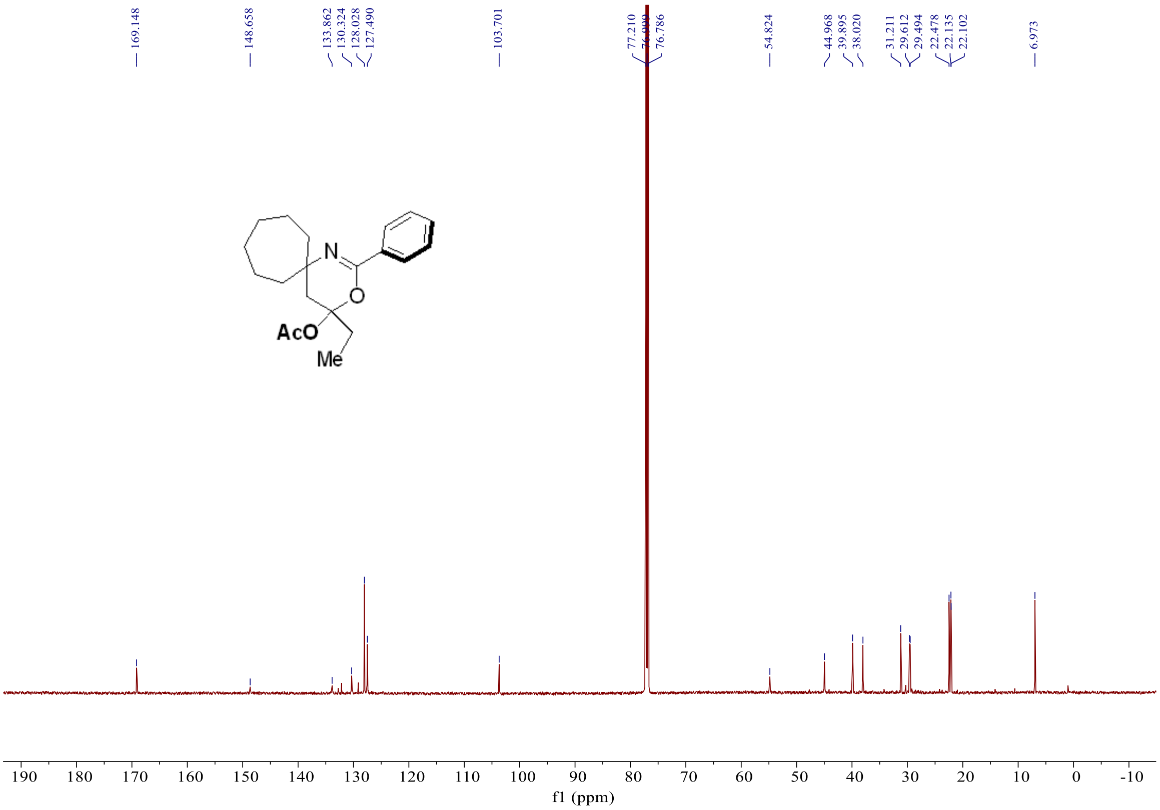

^{1}H NMR spectrum of **2l** (500 MHz, $CDCl_3$)

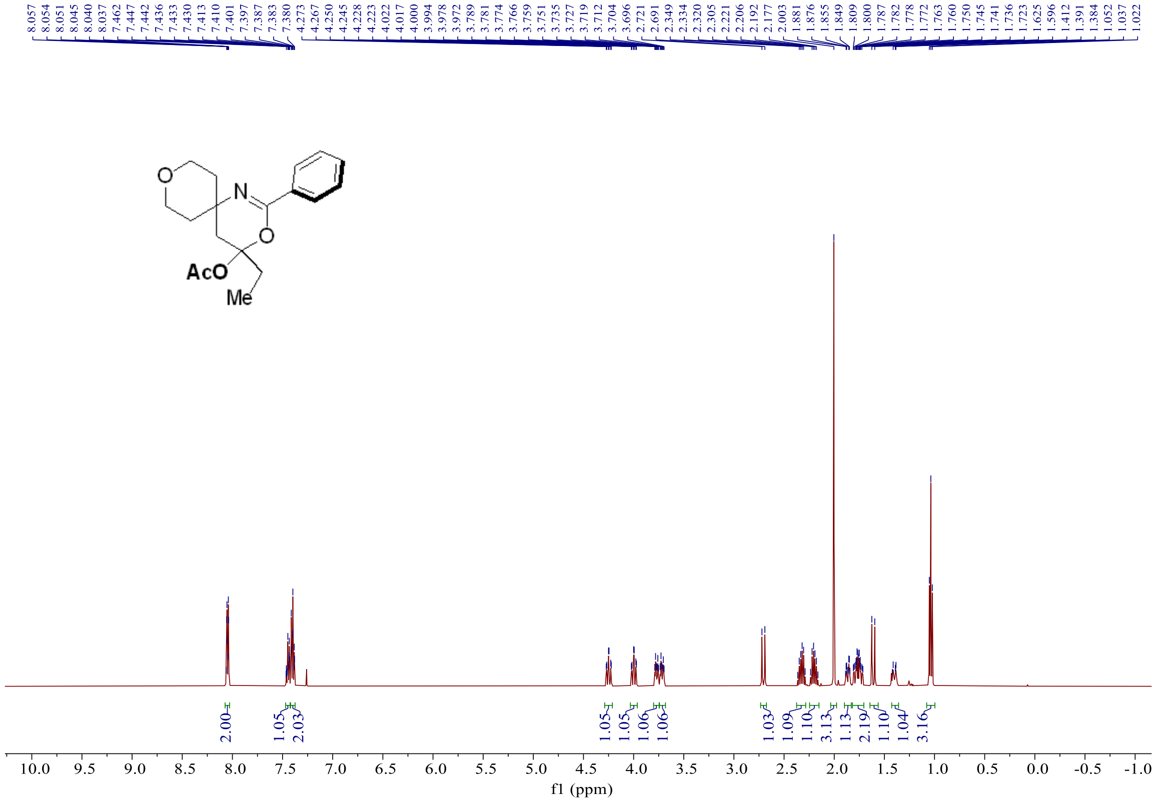

^{13}C NMR spectrum of **2l** (126 MHz, $CDCl_3$)

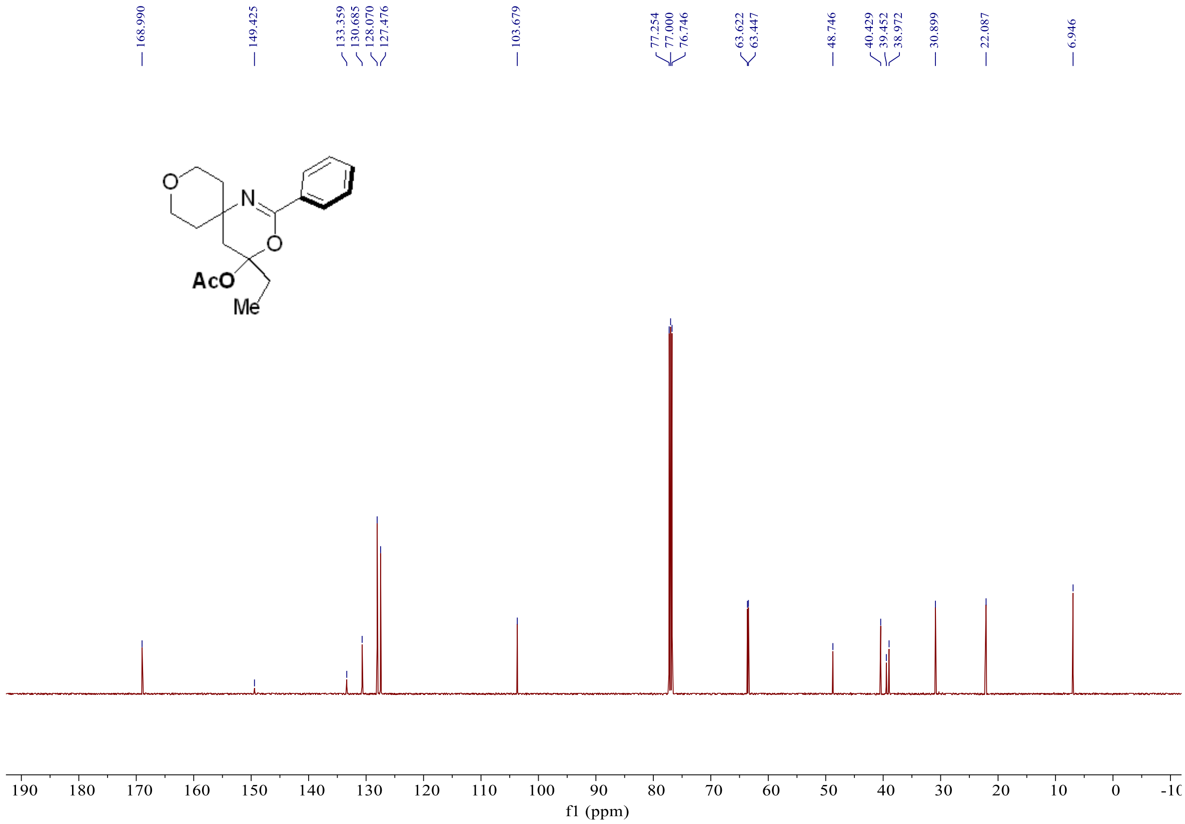

^{1}H NMR spectrum of **2m** (600 MHz, $CDCl_3$)

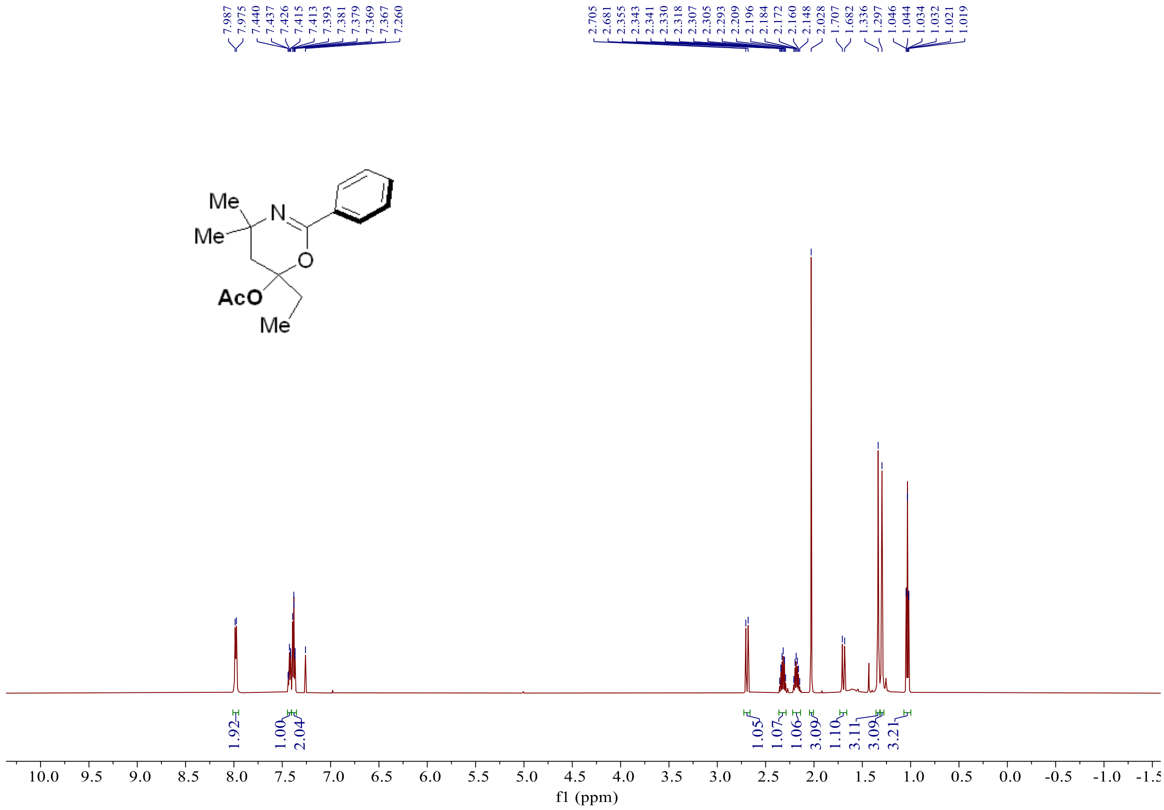

^{13}C NMR spectrum of **2m** (151 MHz, $CDCl_3$)

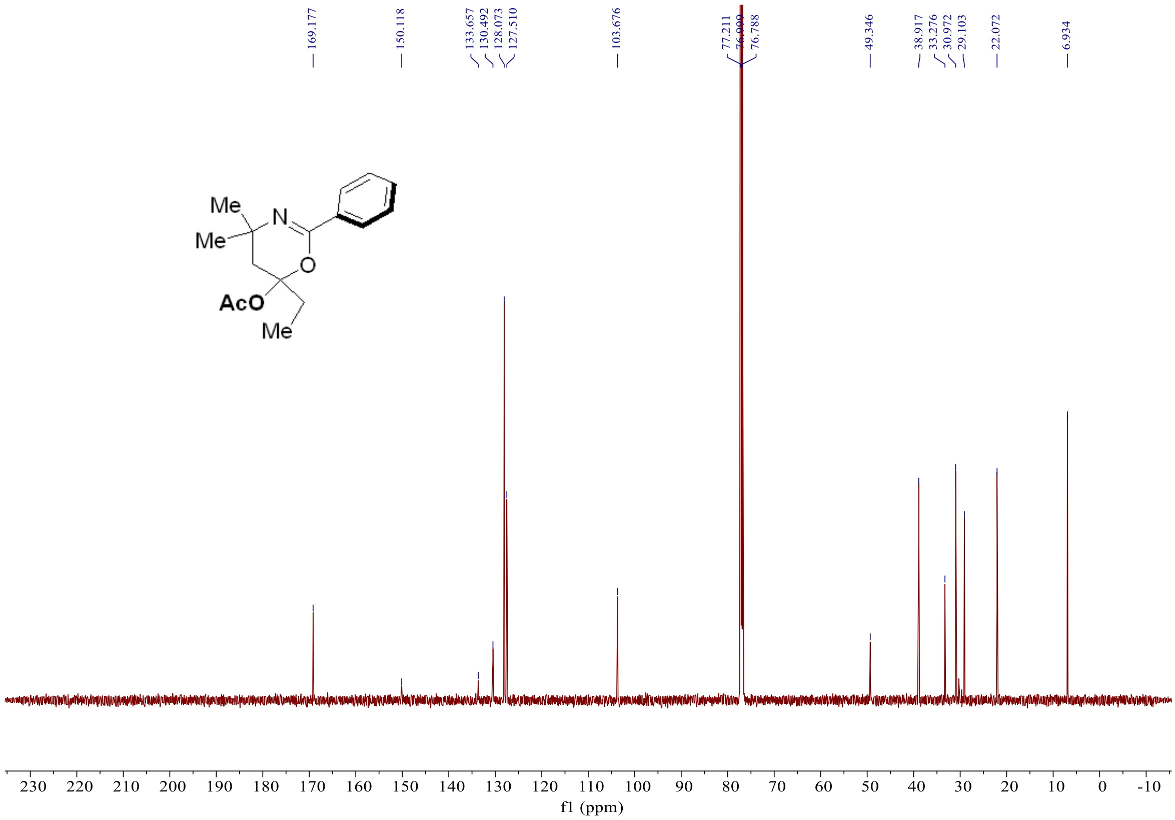

^{1}H NMR spectrum of **2n** (500 MHz, $CDCl_3$)

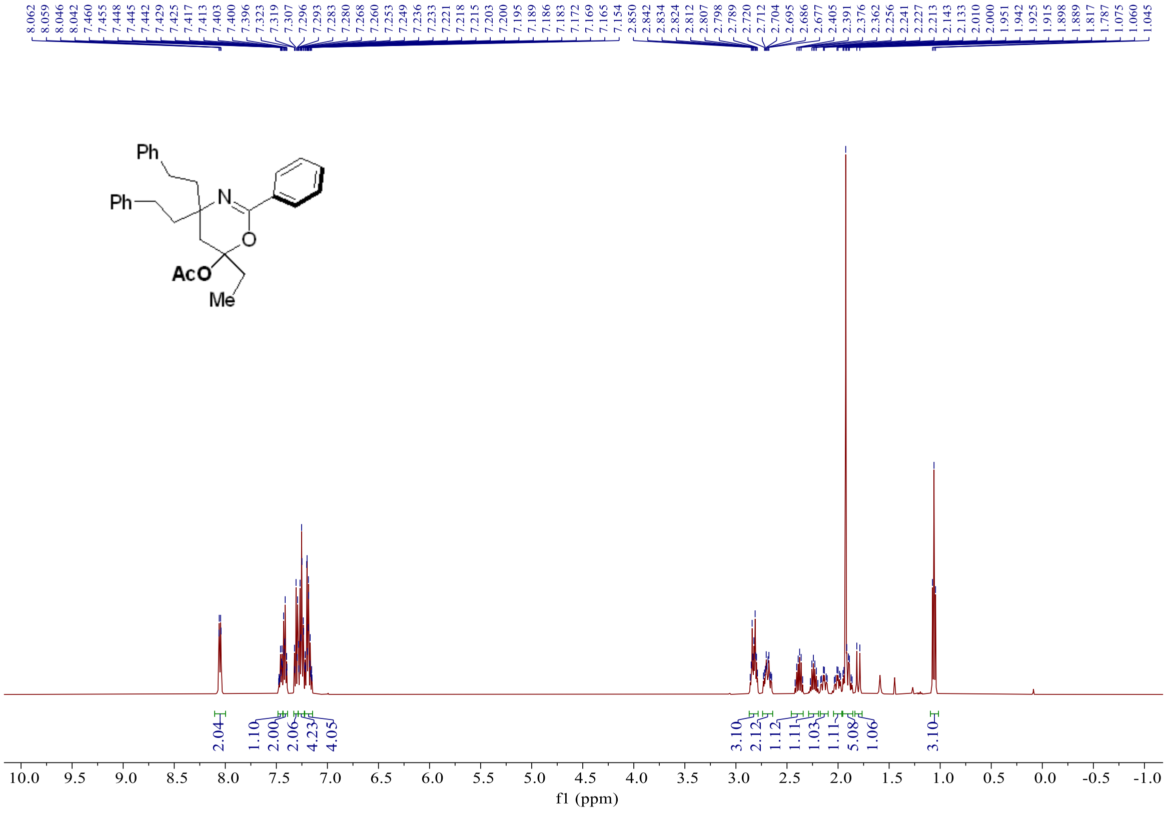

^{13}C NMR spectrum of **2n** (126 MHz, $CDCl_3$)

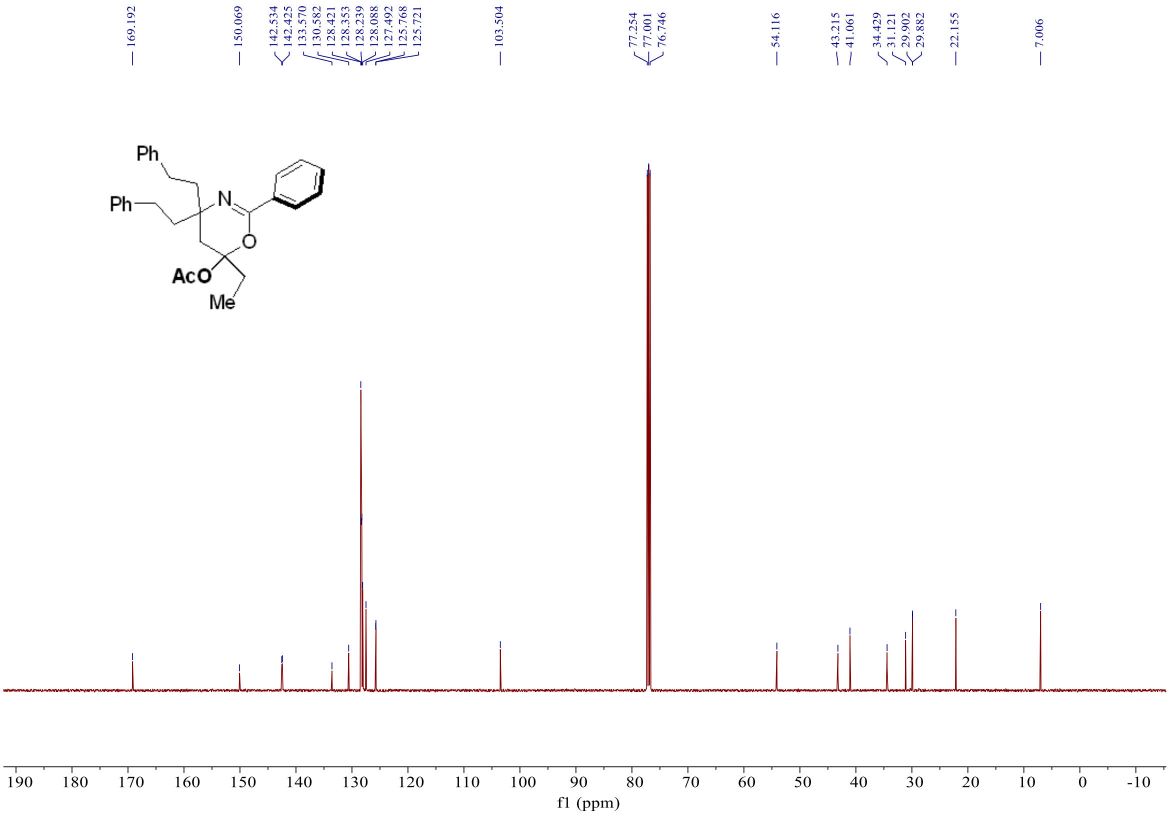

^{1}H NMR spectrum of **2o** (600 MHz, $CDCl_3$)

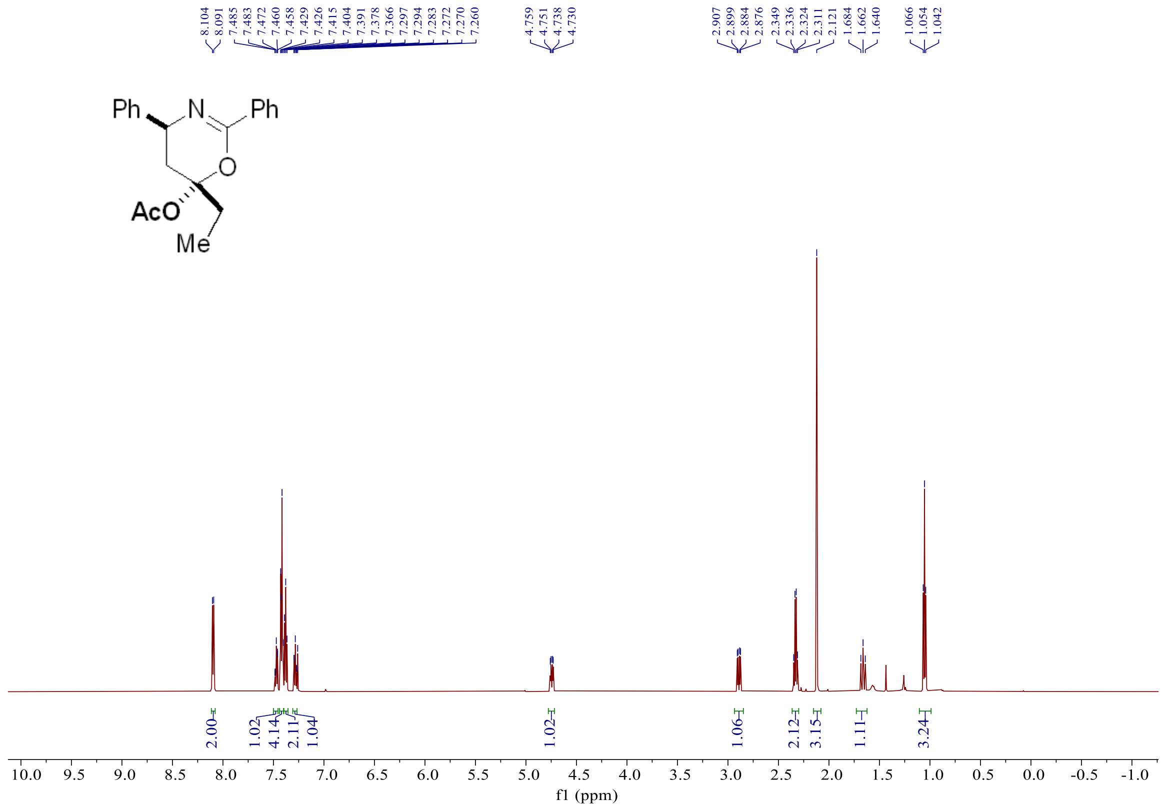

^{13}C NMR spectrum of **2o** (151 MHz, $CDCl_3$)

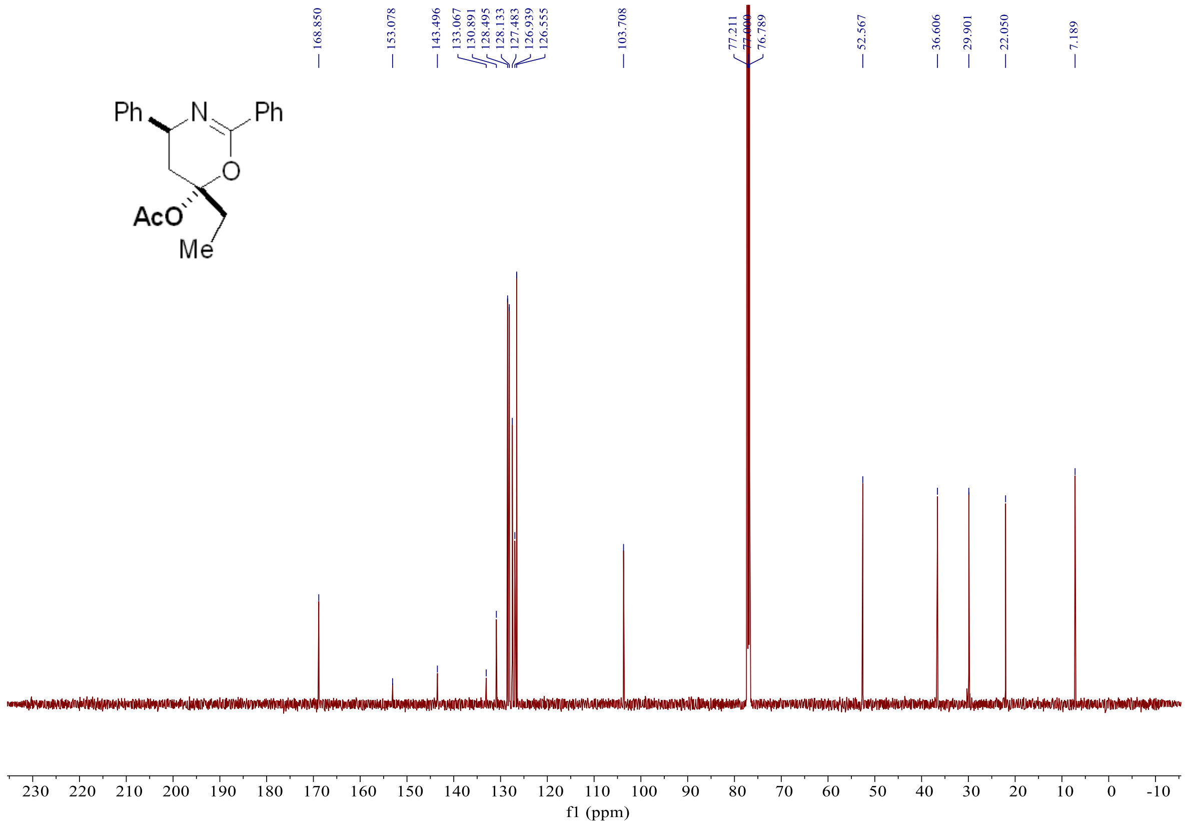

^{1}H NMR spectrum of **2p** (600 MHz, $CDCl_3$)

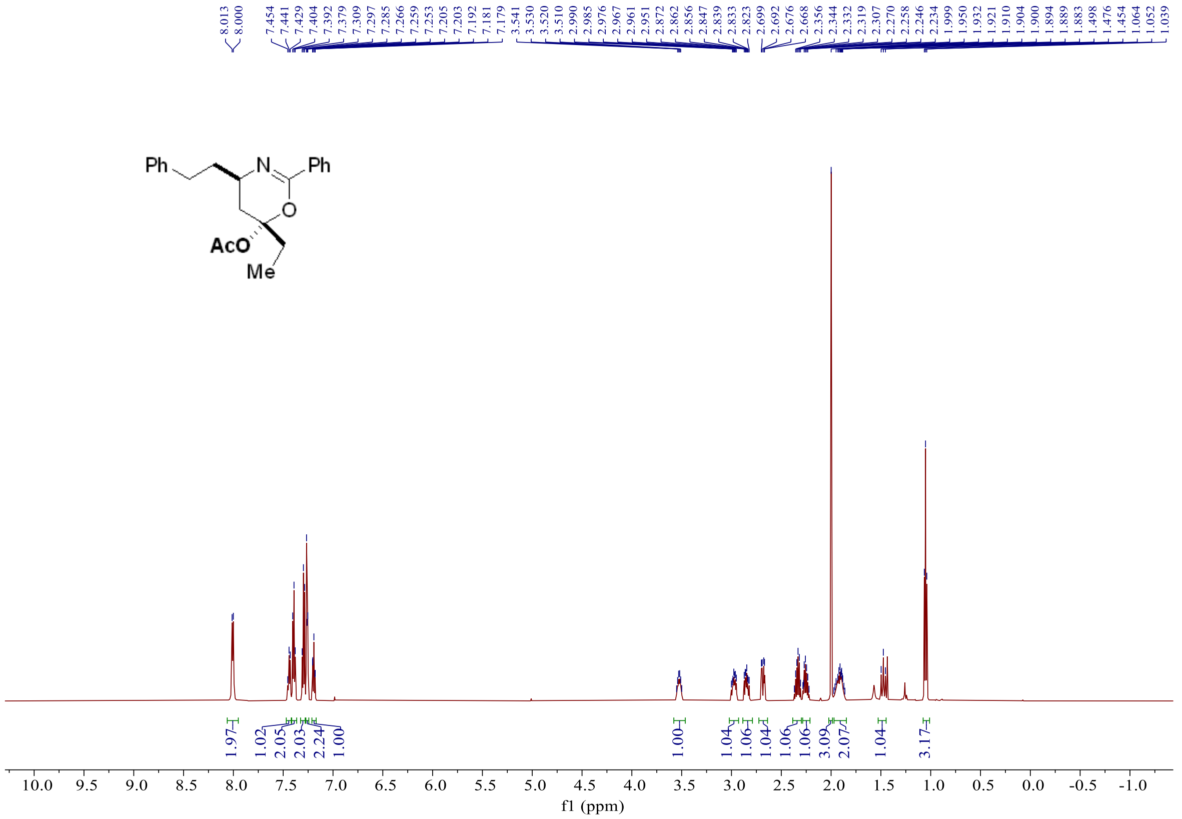

^{13}C NMR spectrum of **2p** (151 MHz, $CDCl_3$)

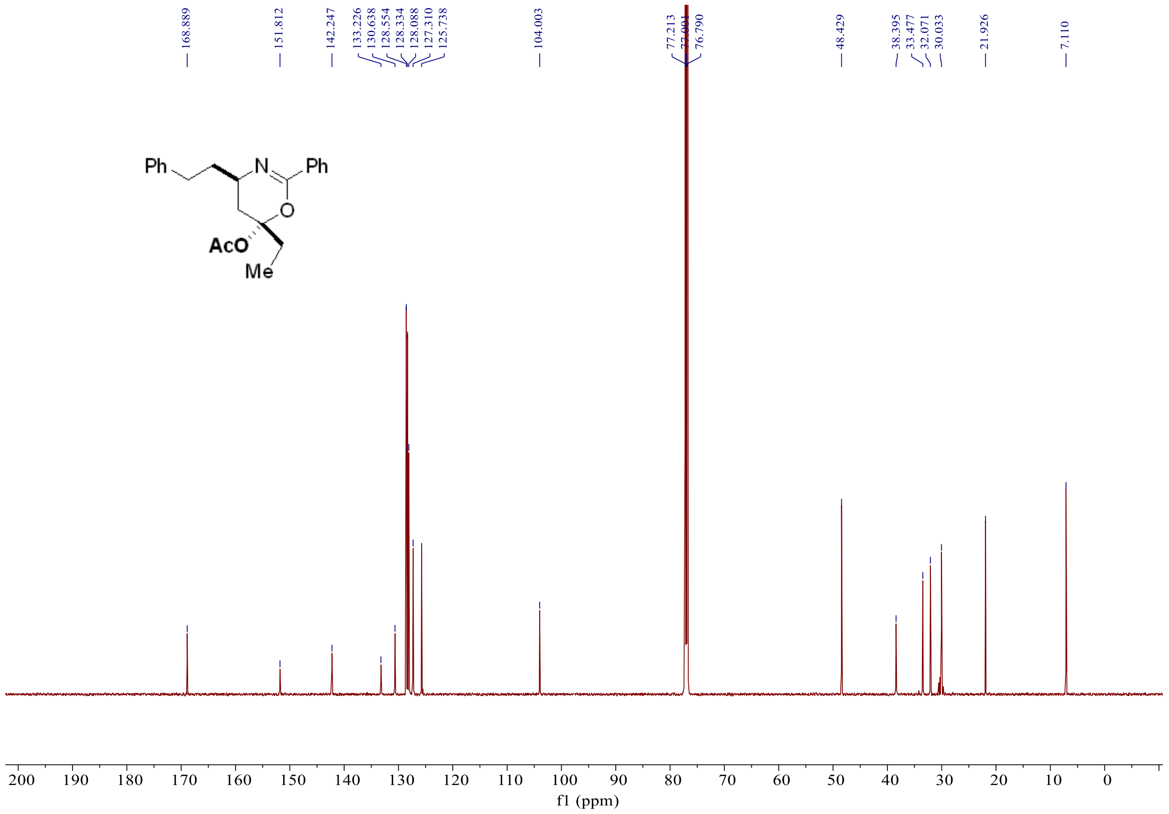

^{1}H NMR spectrum of **2q** (500 MHz, $CDCl_3$)

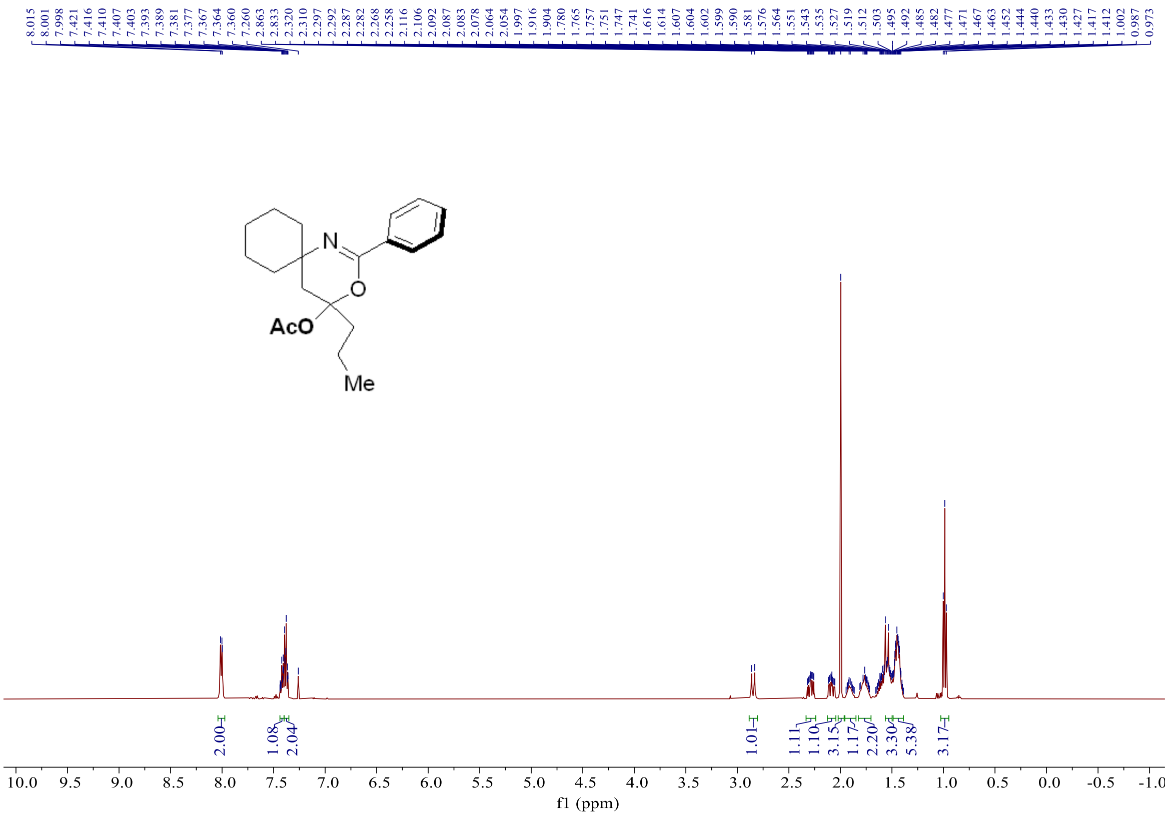

^{13}C NMR spectrum of **2q** (126 MHz, $CDCl_3$)

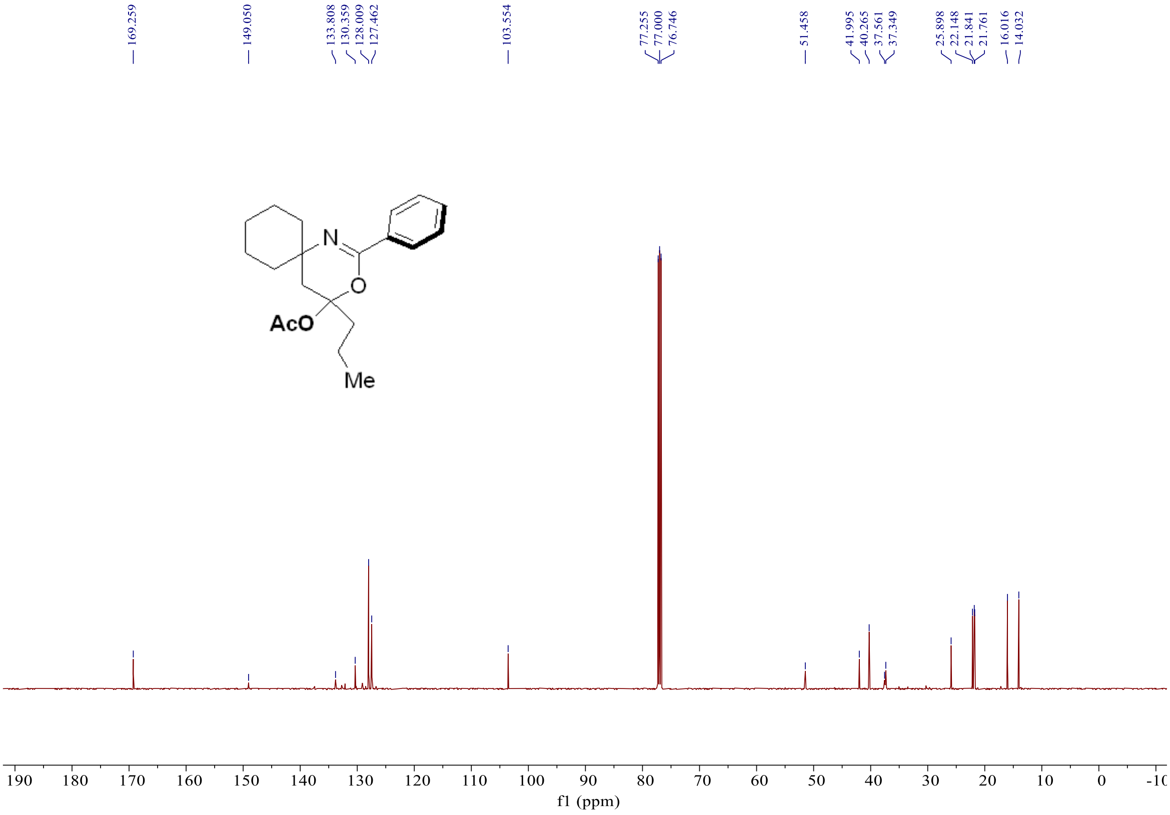

^{1}H NMR spectrum of **2r** (500 MHz, $CDCl_3$)

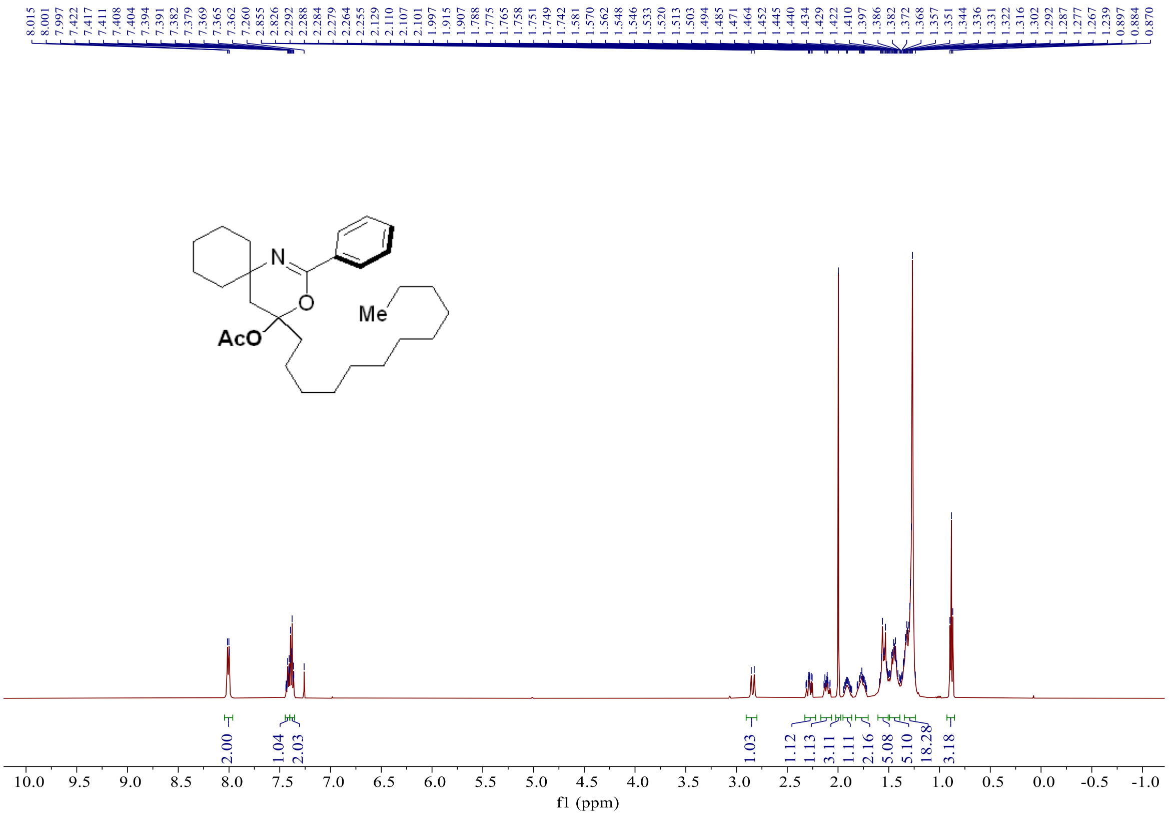

^{13}C NMR spectrum of **2r** (125 MHz, $CDCl_3$)

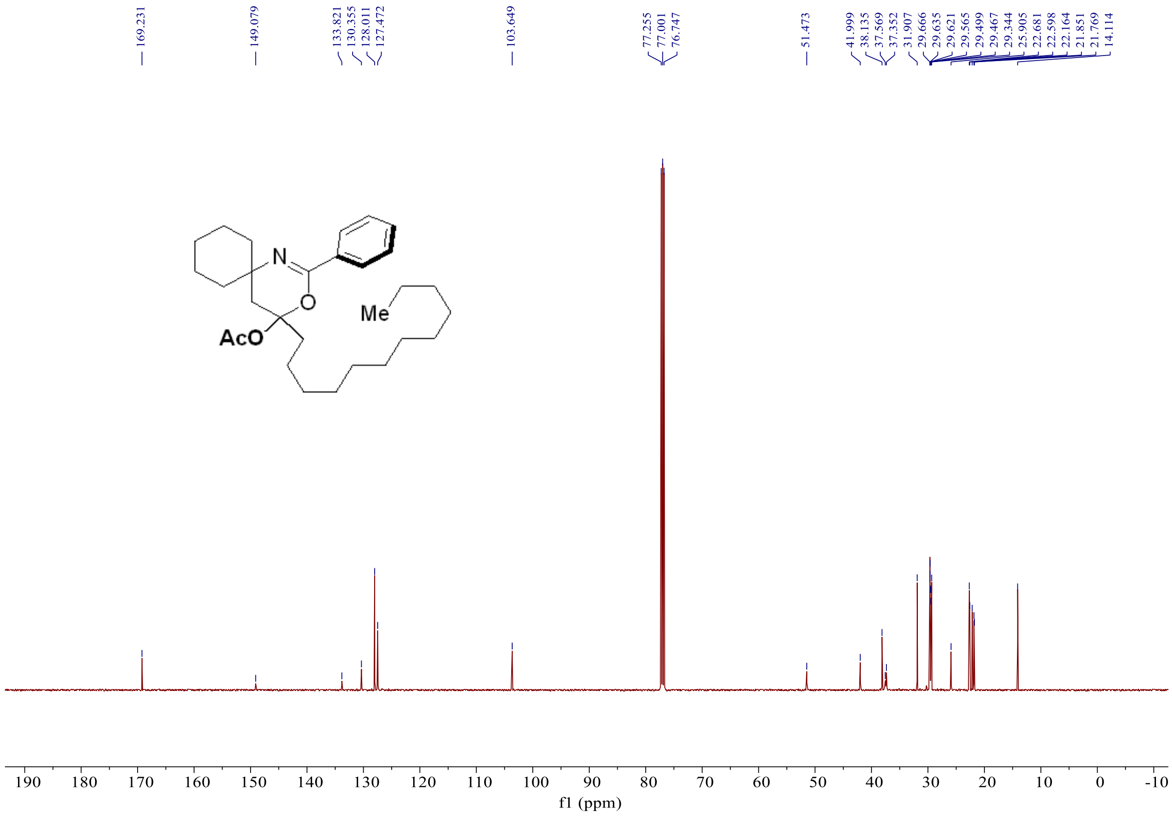

^{1}H NMR spectrum of **2s** (600 MHz, $CDCl_3$)

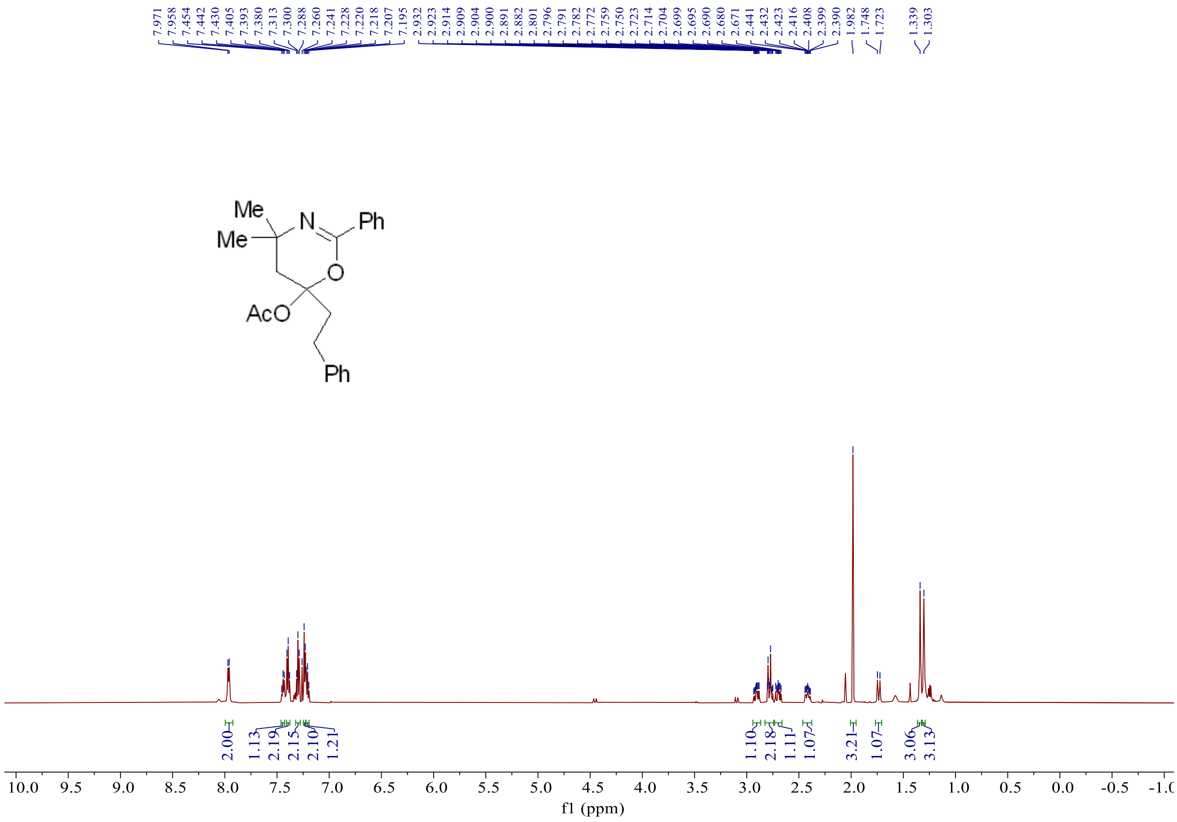

^{13}C NMR spectrum of **2s** (151 MHz, $CDCl_3$)

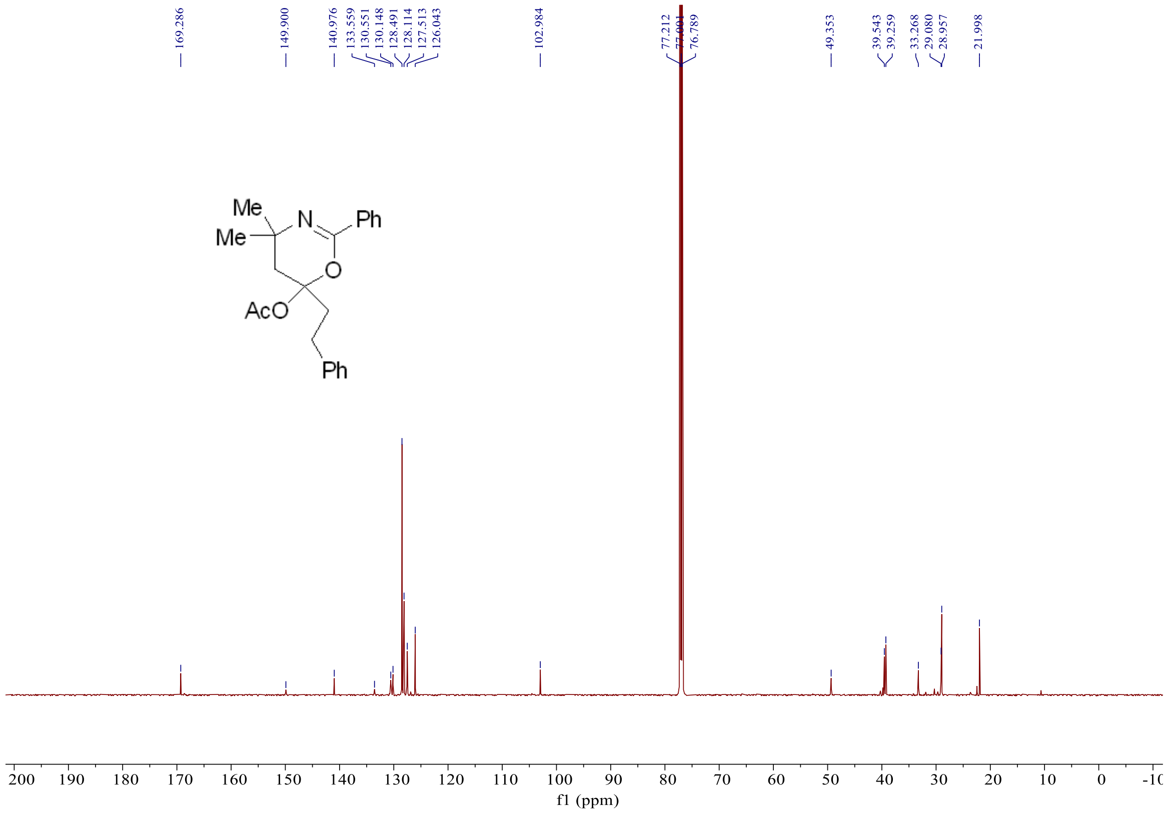

^{1}H NMR spectrum of **2t** (600 MHz, $CDCl_3$)

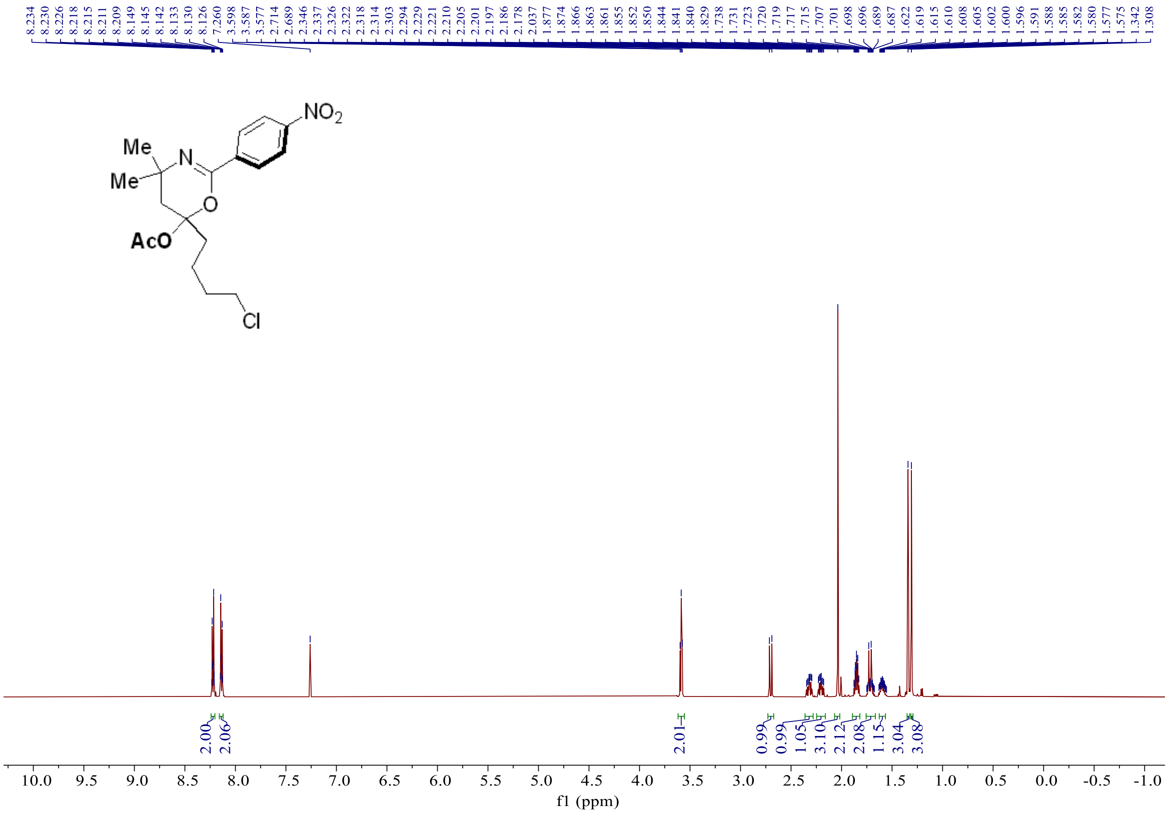

^{13}C NMR spectrum of **2t** (151 MHz, $CDCl_3$)

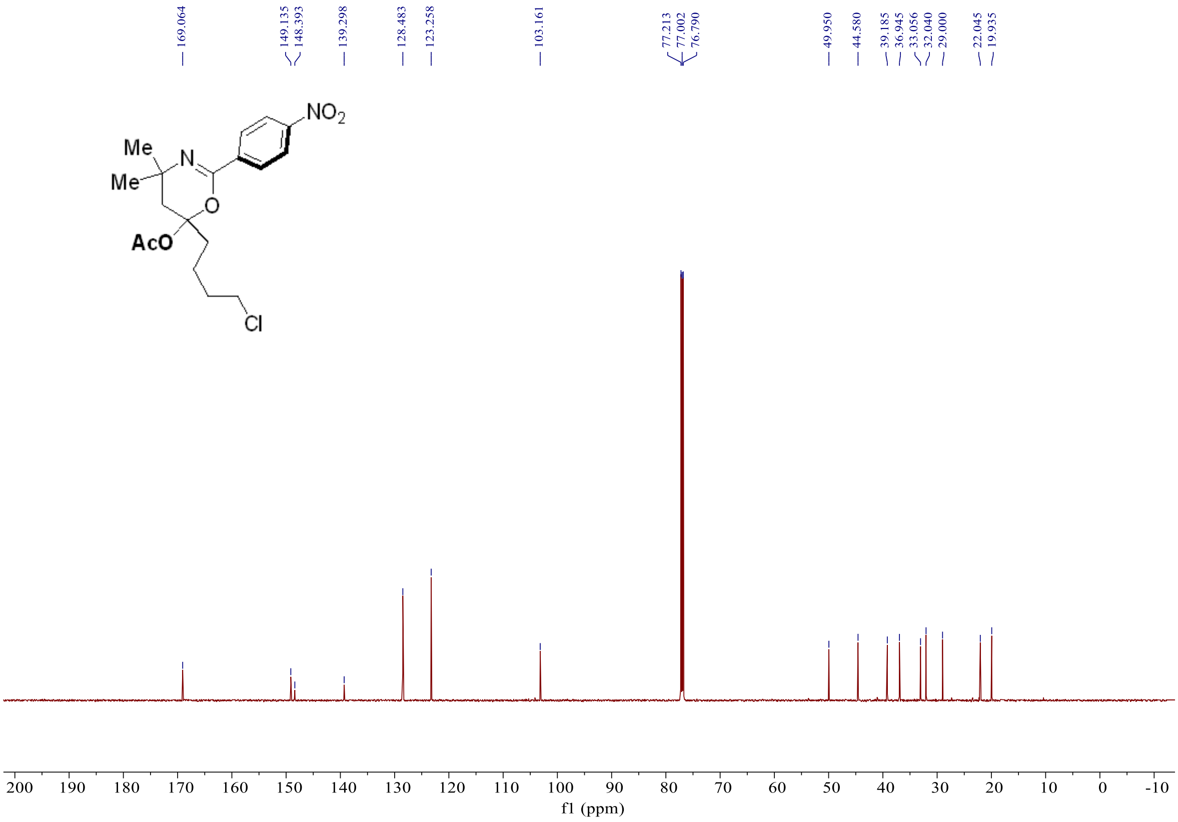

^{1}H NMR spectrum of **2u** (600 MHz, $CDCl_3$)

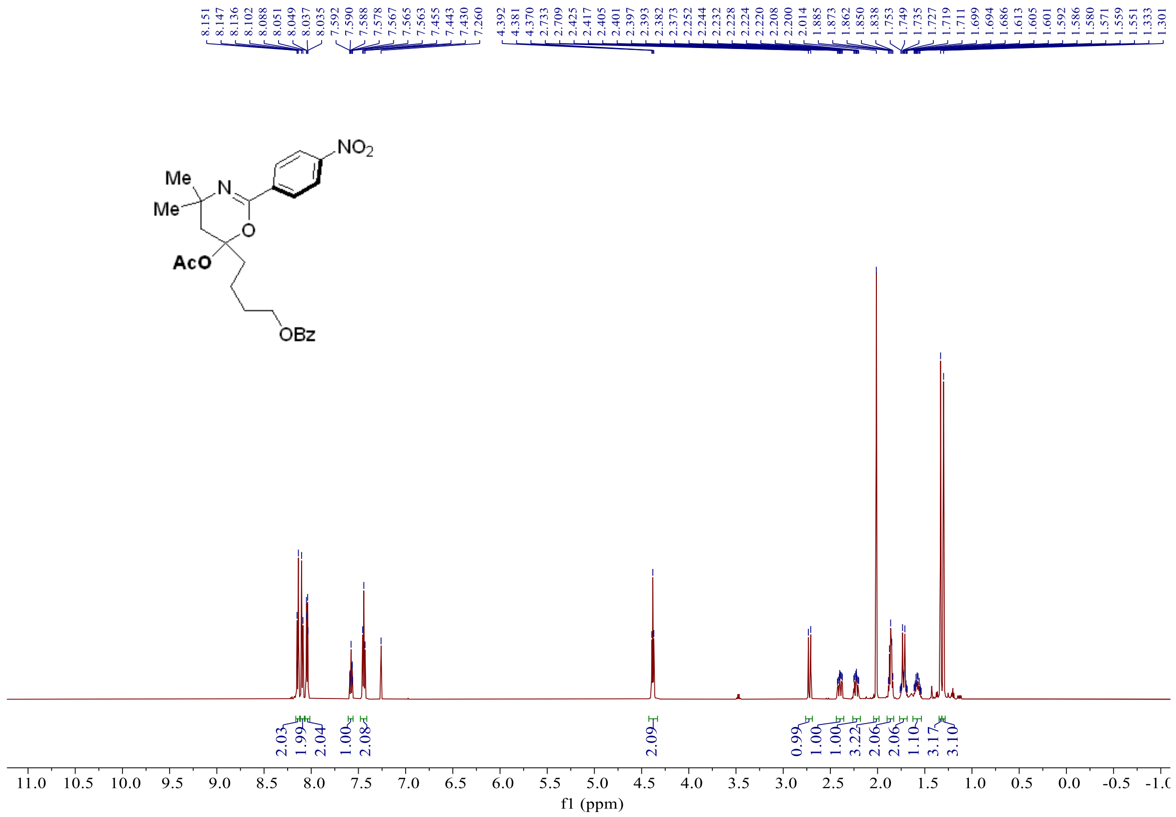

^{13}C NMR spectrum of **2u** (151 MHz, $CDCl_3$)

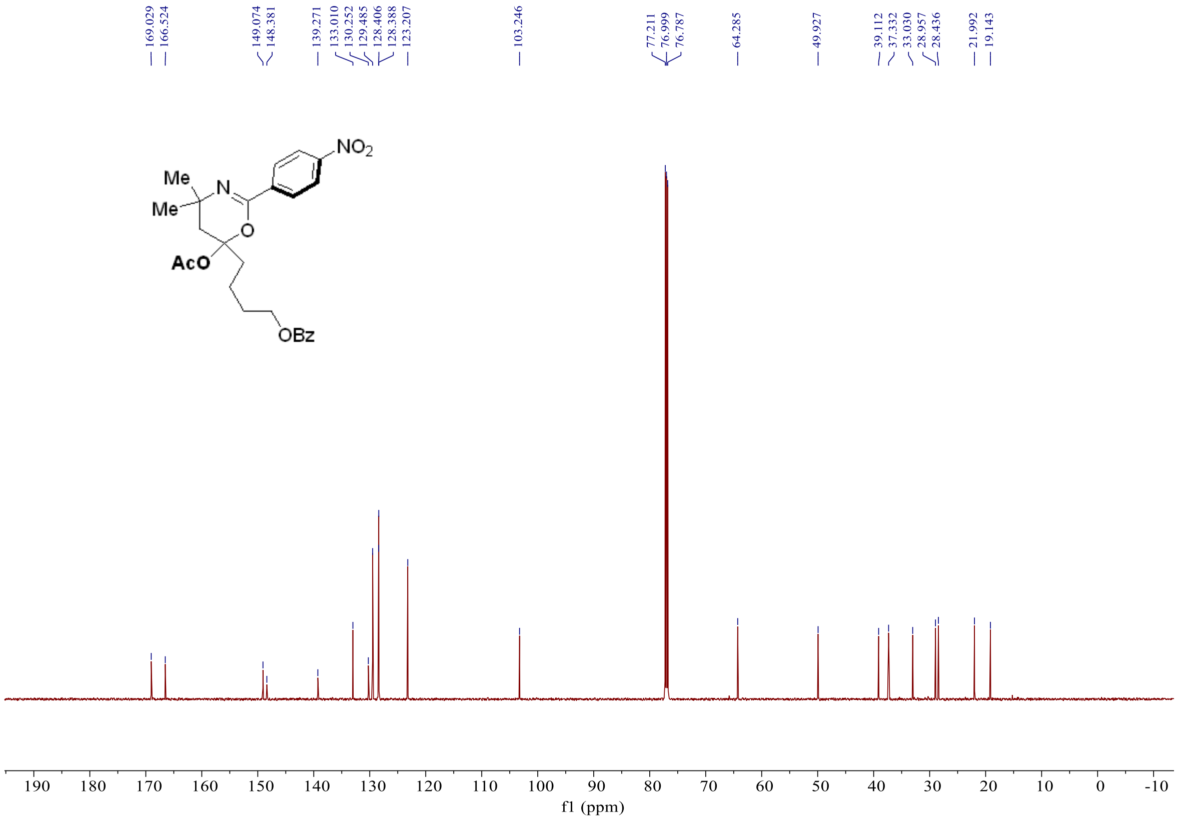

^{1}H NMR spectrum of **2v** (600 MHz, $CDCl_3$)

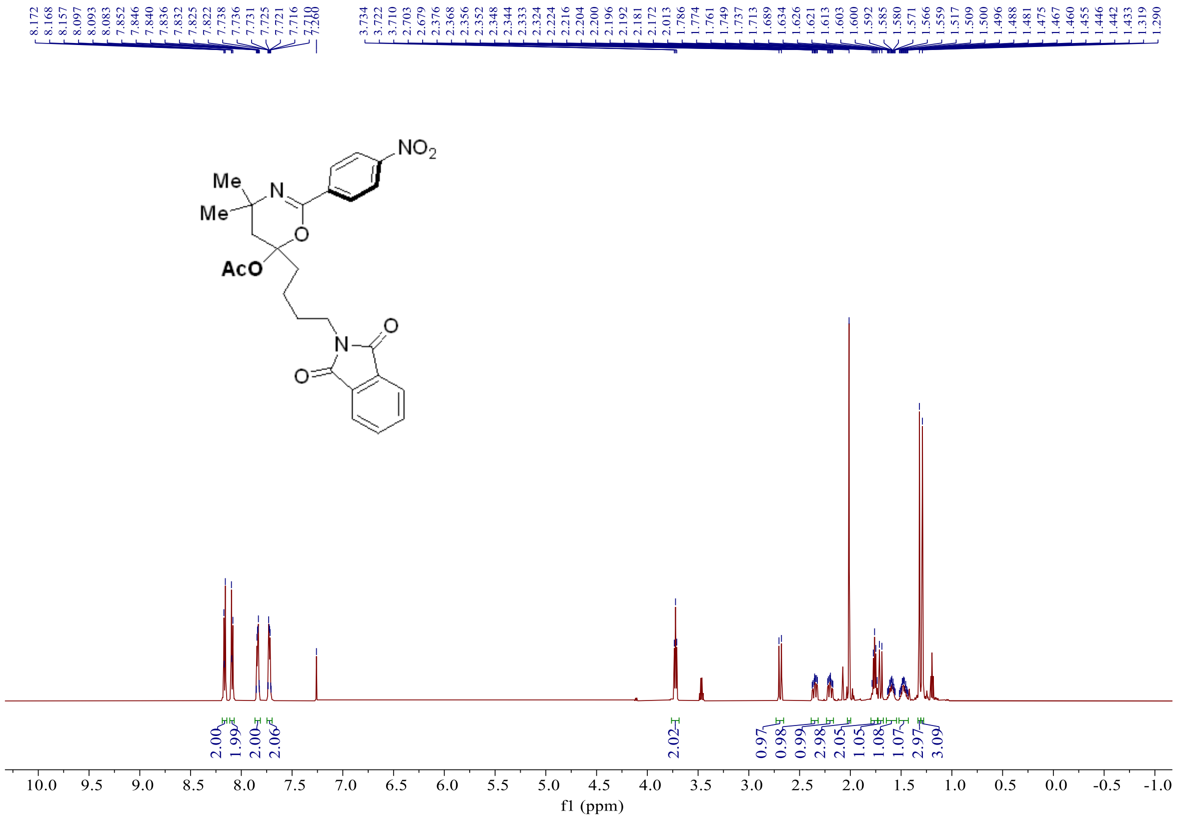

^{13}C NMR spectrum of **2v** (151 MHz, $CDCl_3$)

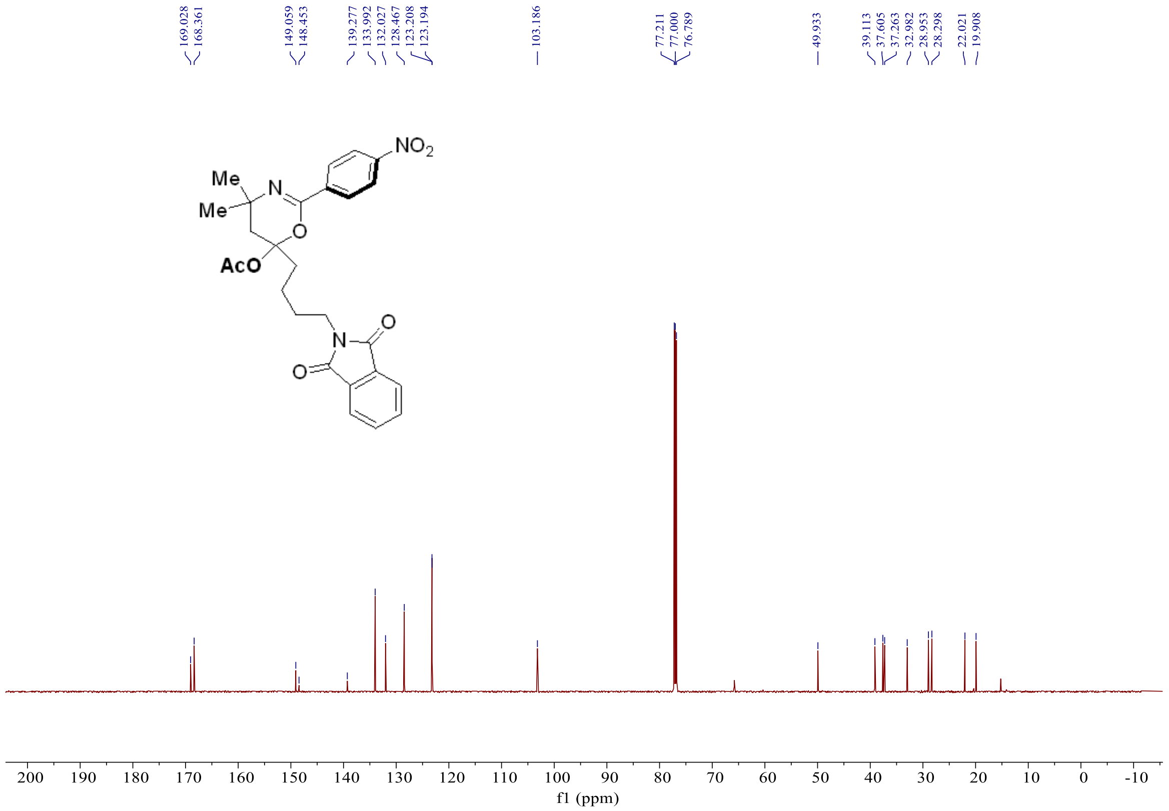

^{1}H NMR spectrum of **2w** (500 MHz, $CDCl_3$)

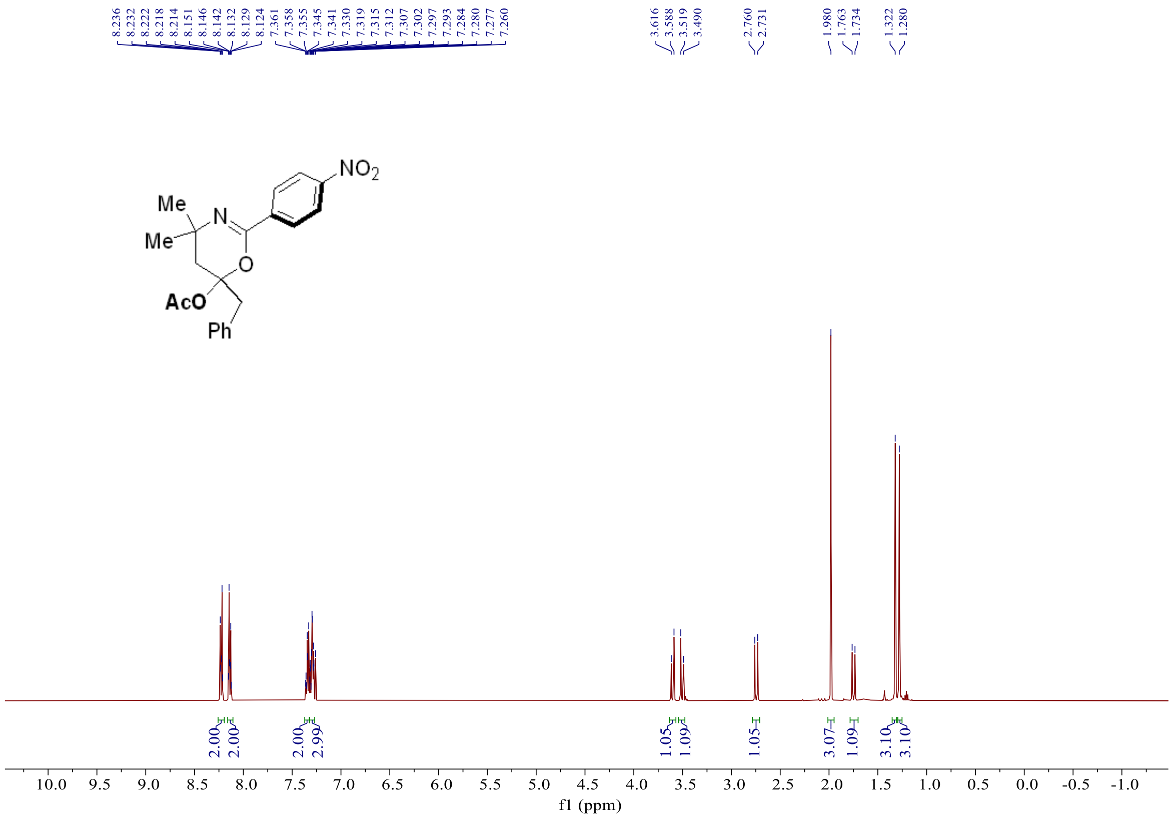

^{13}C NMR spectrum of **2w** (126 MHz, $CDCl_3$)

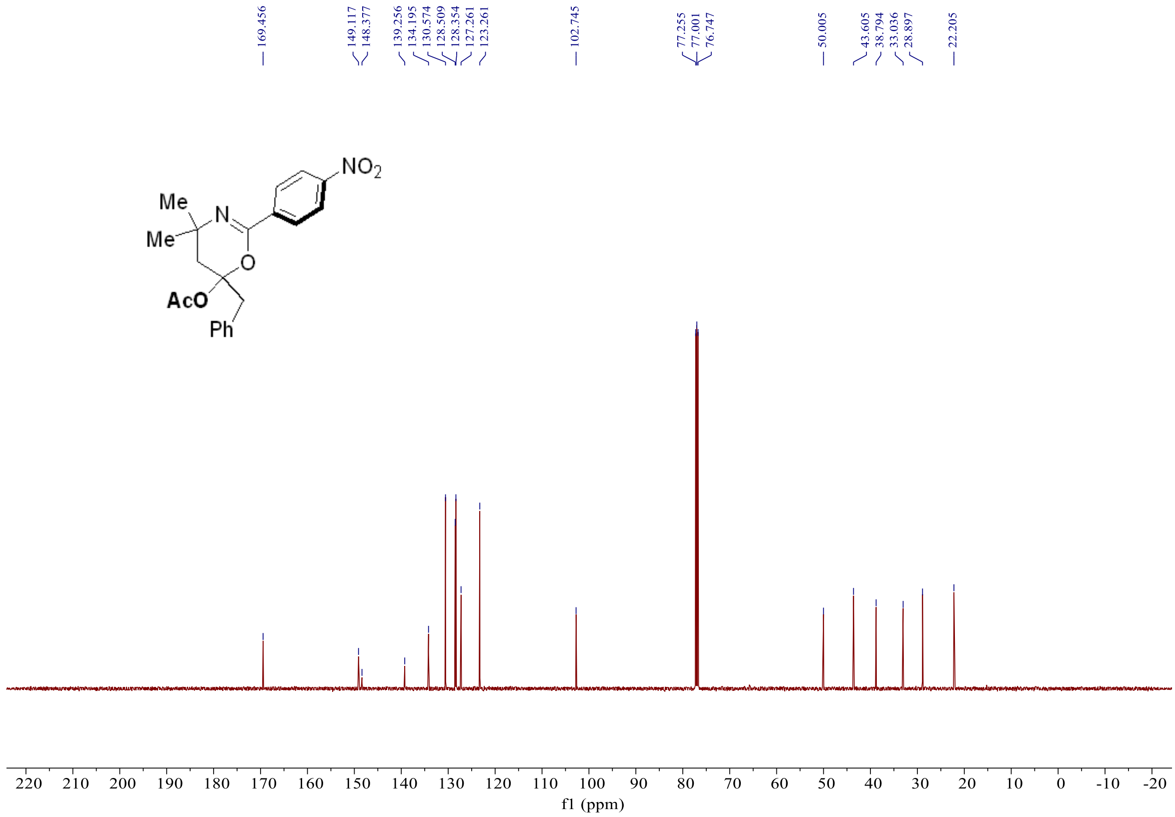

^{1}H NMR spectrum of **2x** (500 MHz, $CDCl_3$)

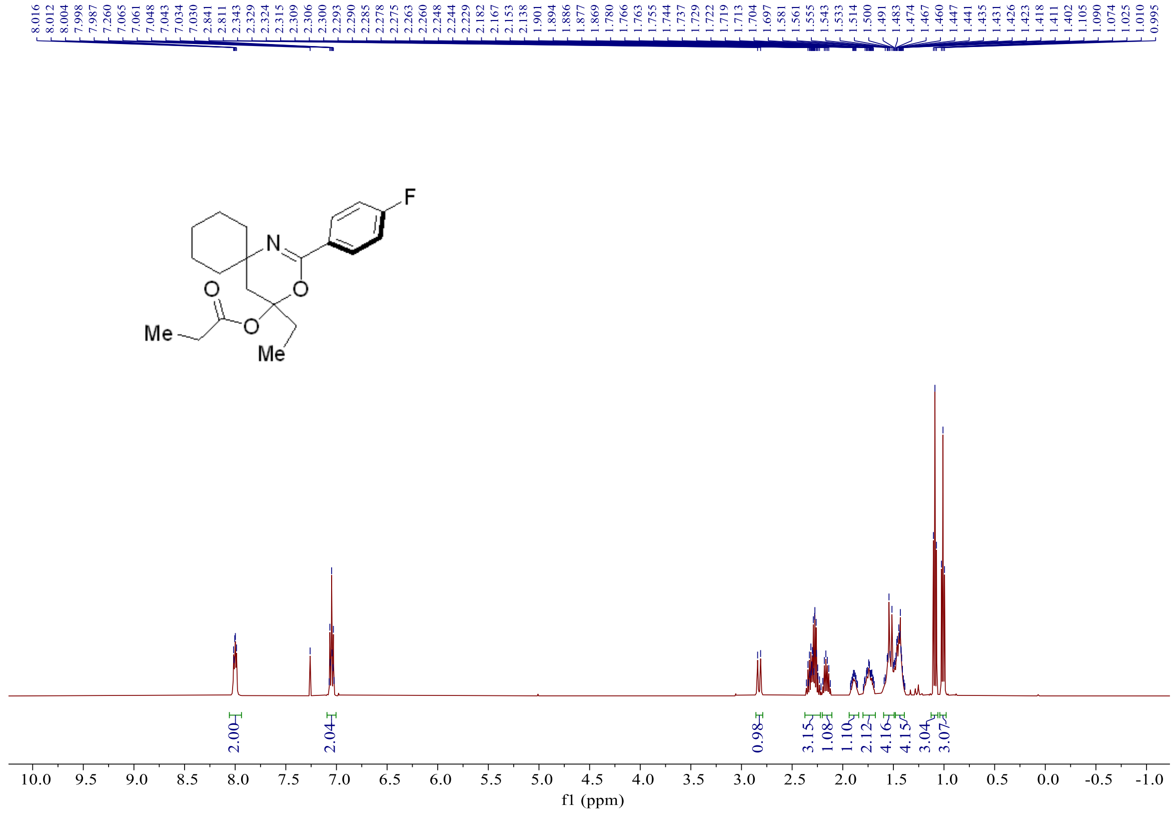

^{13}C NMR spectrum of **2x** (126 MHz, $CDCl_3$)

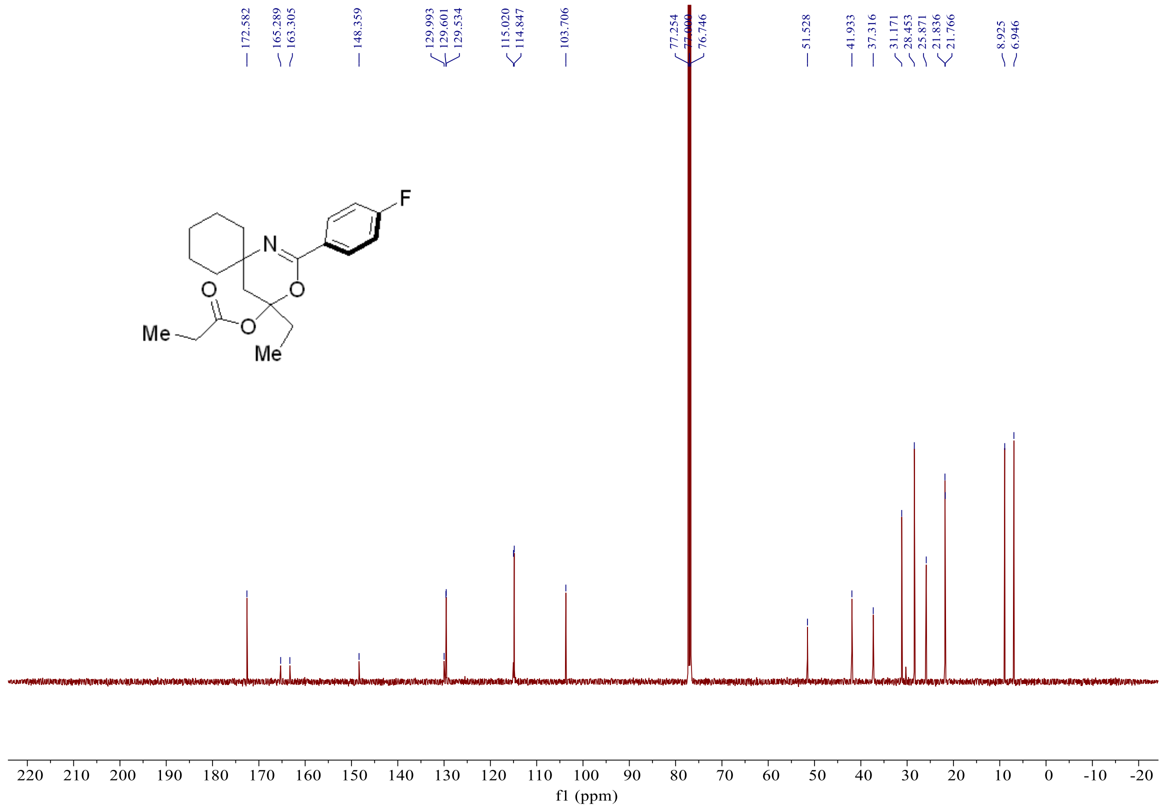

^{19}F NMR spectrum of **2x** (377 MHz, $CDCl_3$)

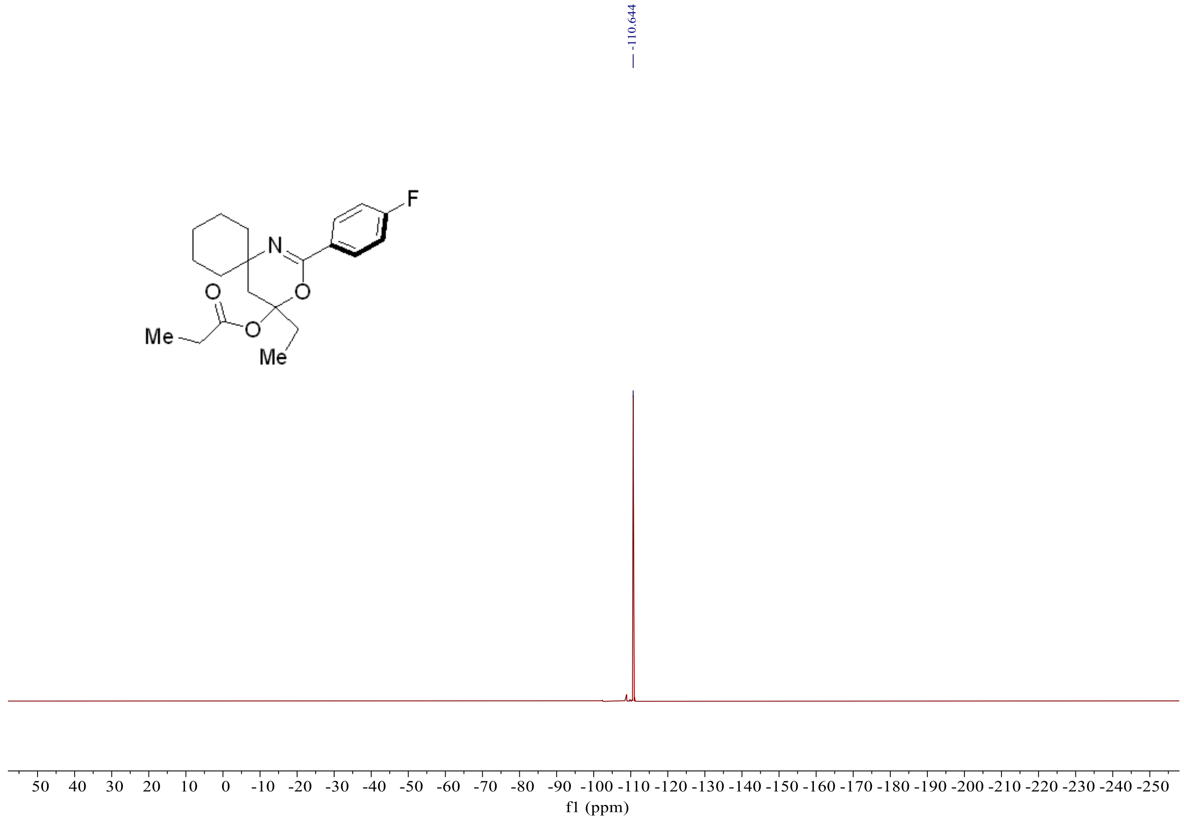

^{1}H NMR spectrum of **2y** (600 MHz, $CDCl_3$)

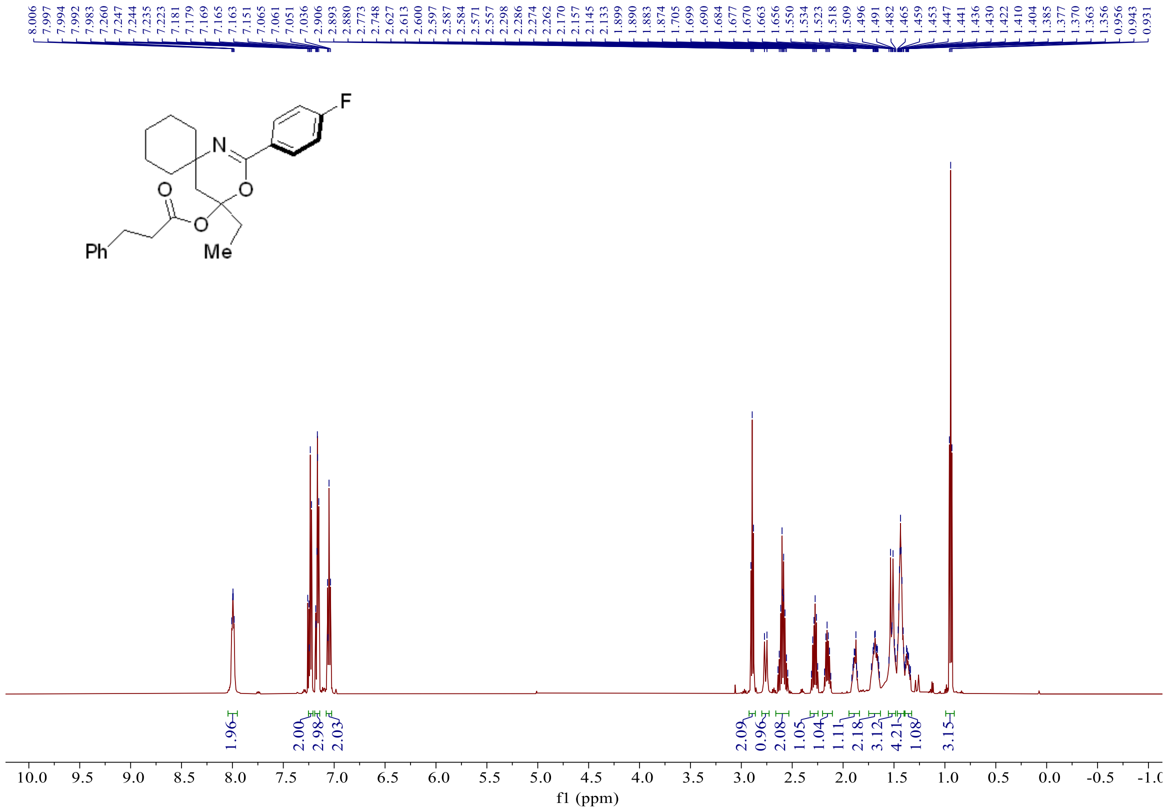

^{13}C NMR spectrum of **2y** (151 MHz, $CDCl_3$)

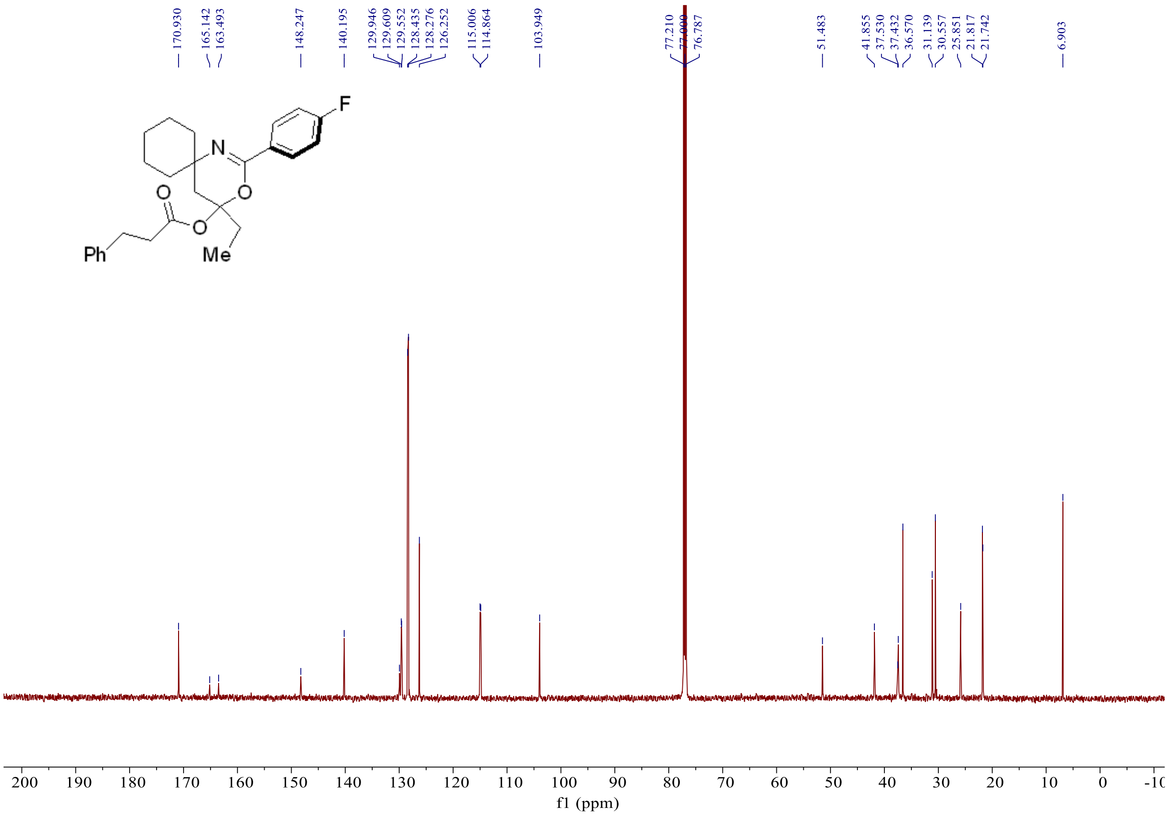

^{19}F NMR spectrum of **2y** (377 MHz, $CDCl_3$)

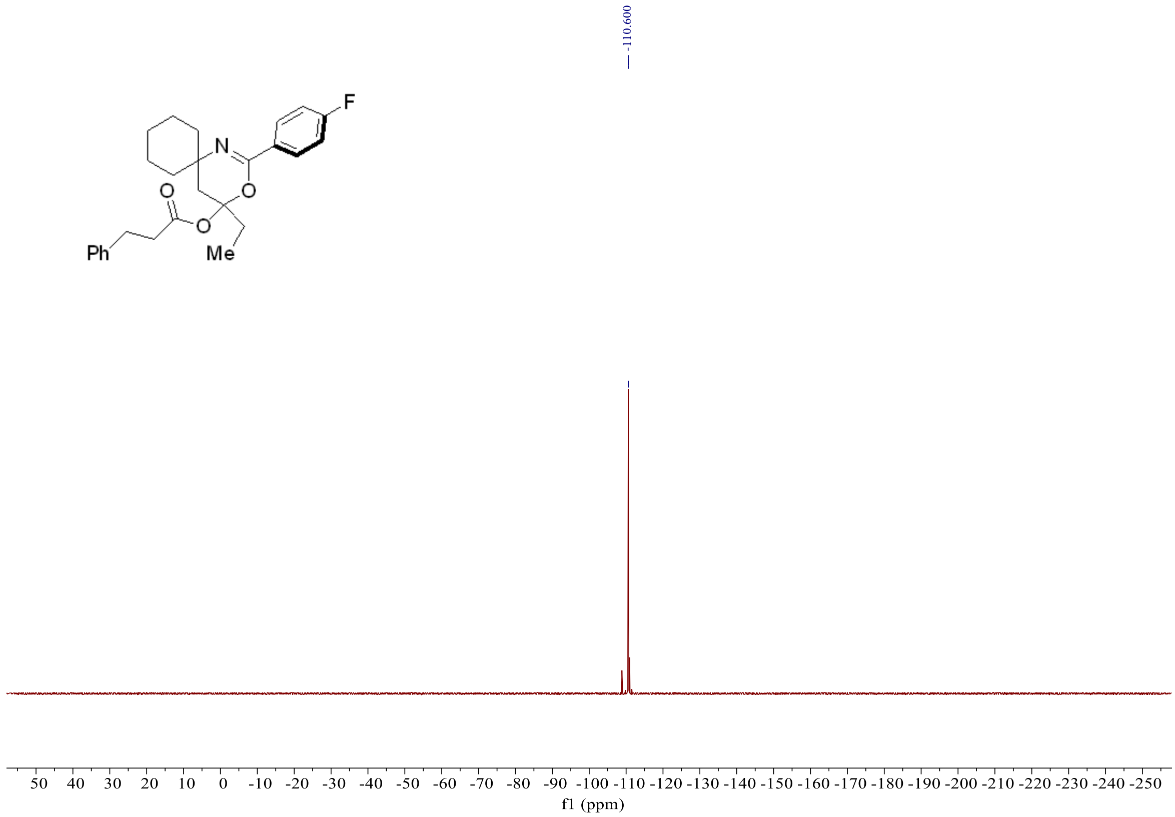

^{1}H NMR spectrum of **2z** (500 MHz, $CDCl_3$)

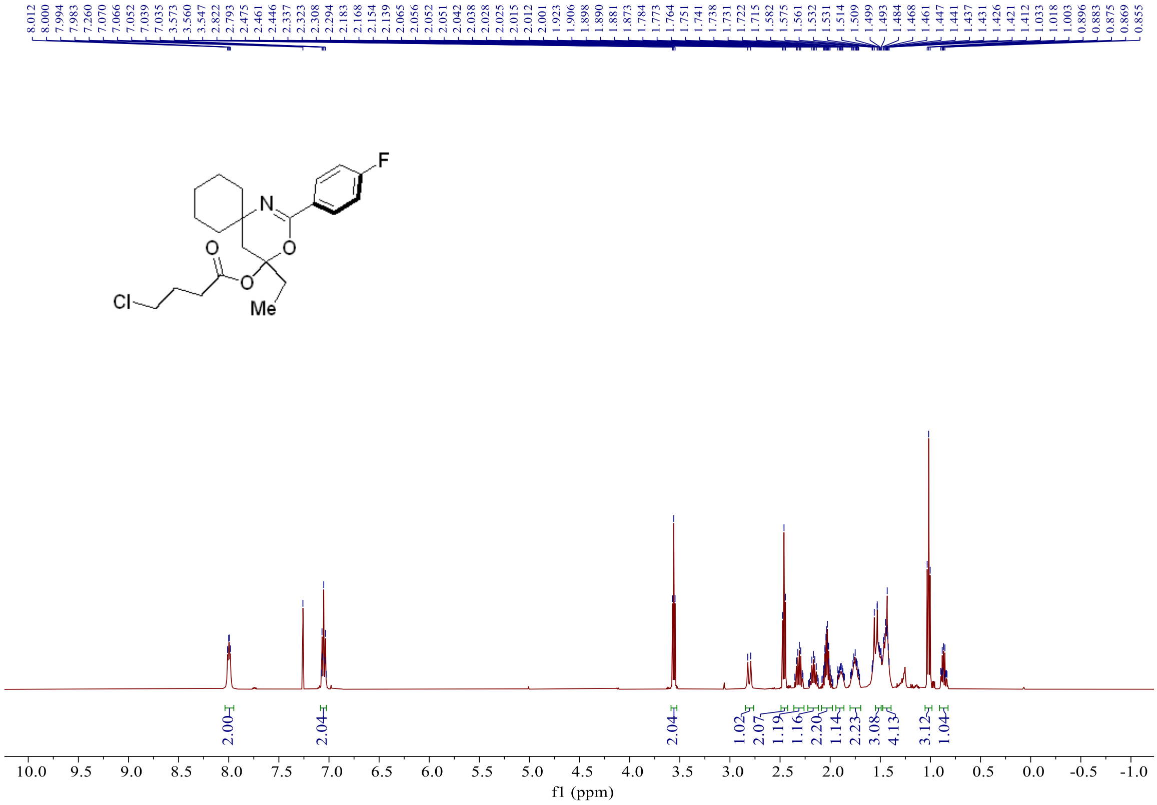

^{13}C NMR spectrum of **2z** (126 MHz, $CDCl_3$)

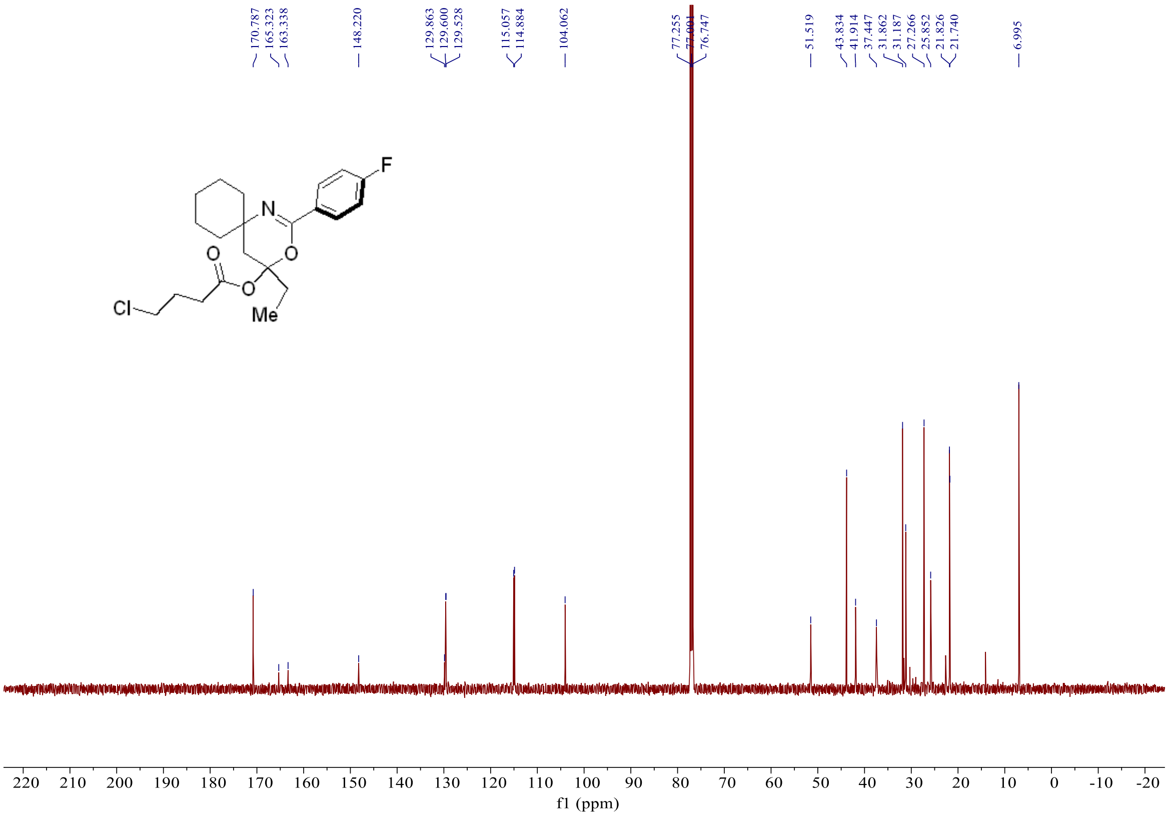

^{19}F NMR spectrum of **2z** (377 MHz, $CDCl_3$)

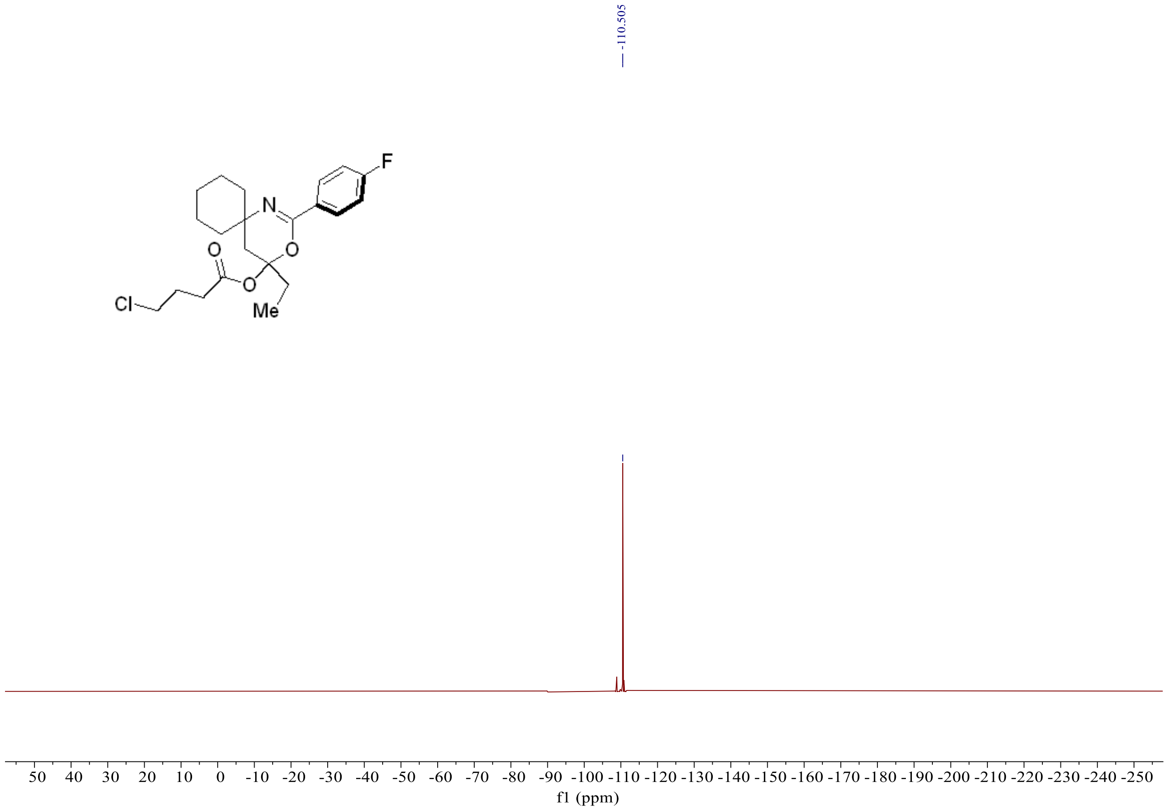

^{1}H NMR spectrum of **2aa** (600 MHz, $CDCl_3$)

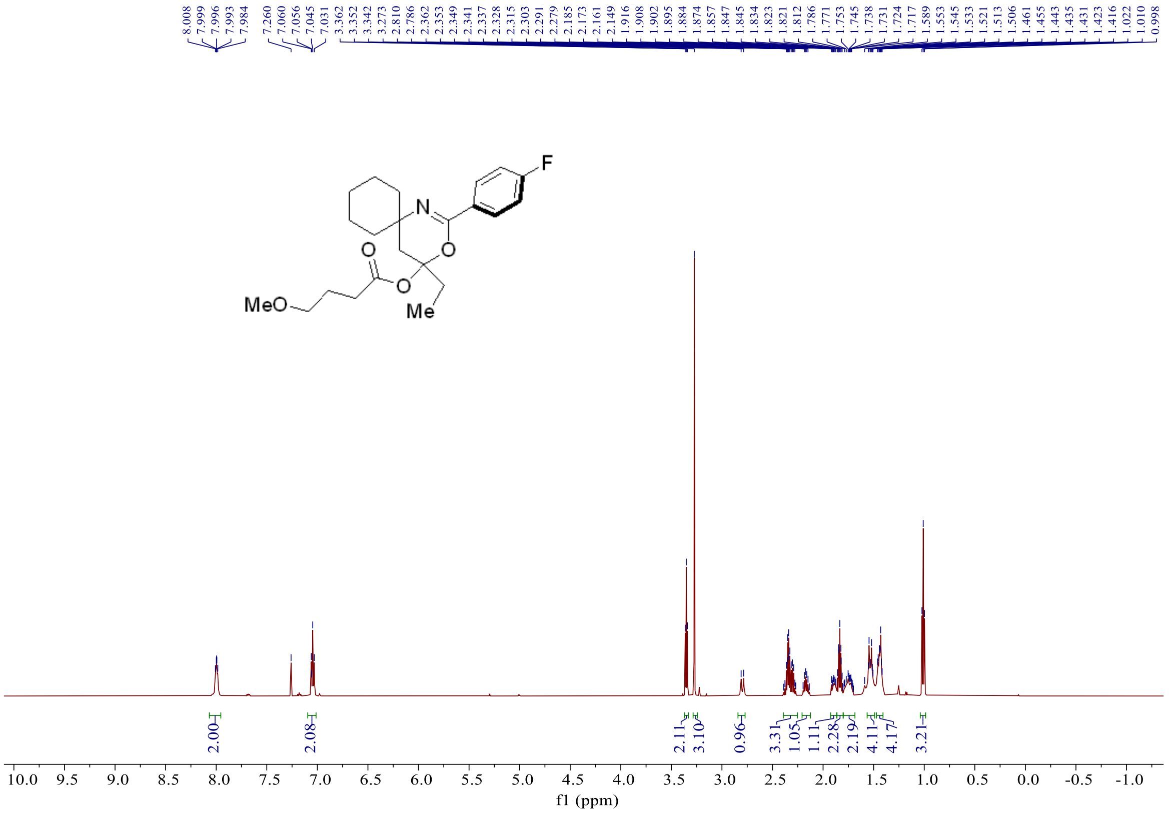

^{13}C NMR spectrum of **2aa** (151 MHz, $CDCl_3$)

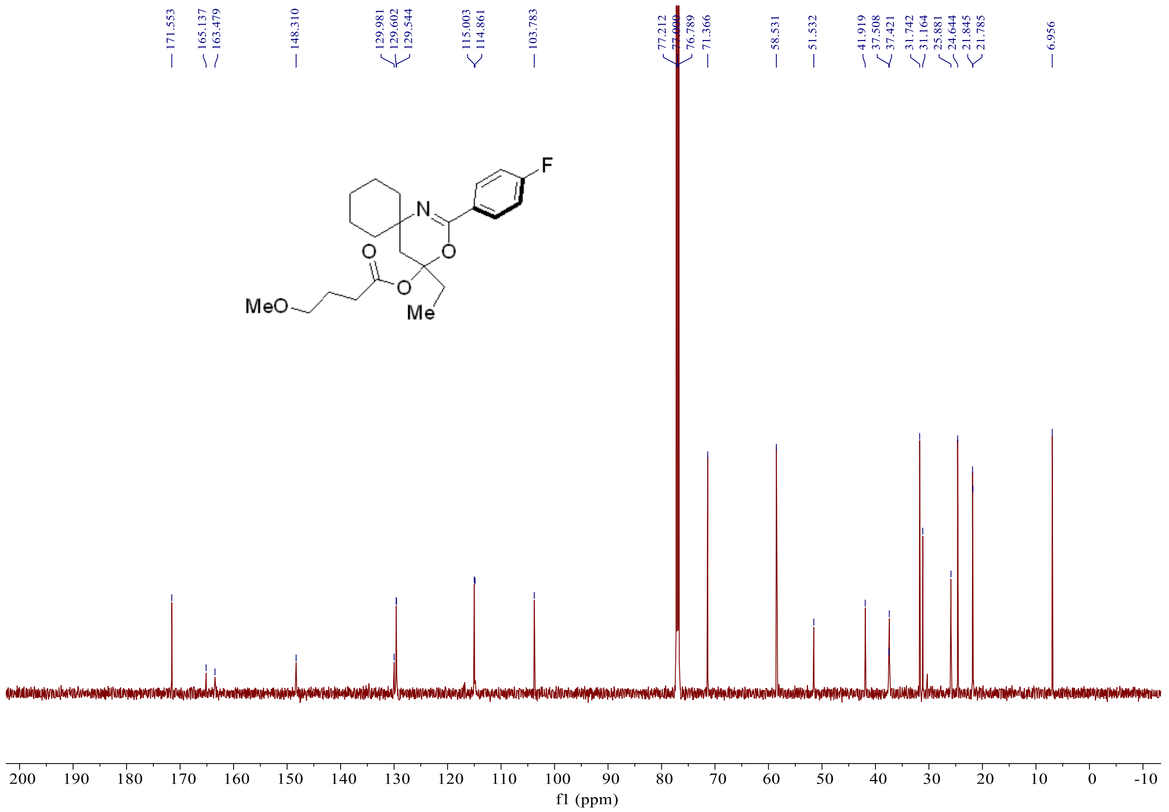

^{19}F NMR spectrum of **2aa** (377 MHz, $CDCl_3$)

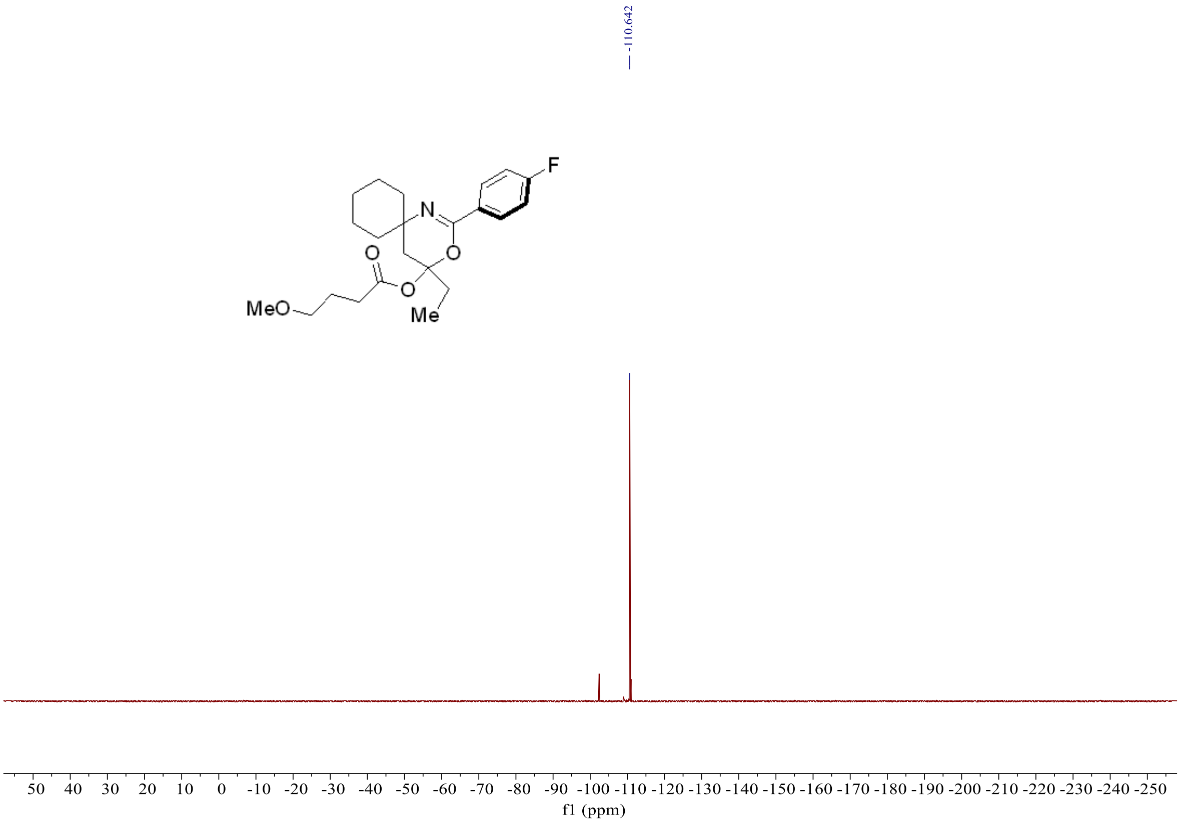

^{1}H NMR spectrum of **2ab** (600 MHz, $CDCl_3$)

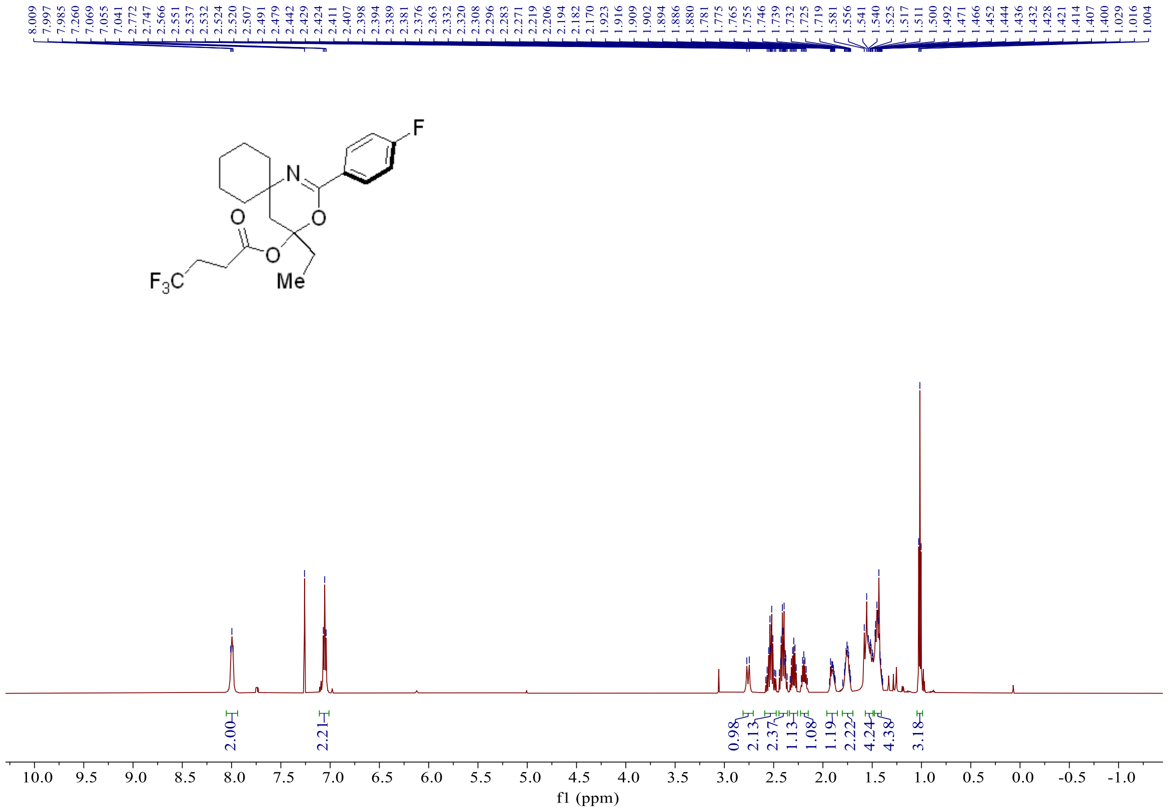

^{13}C NMR spectrum of **2ab** (151 MHz, $CDCl_3$)

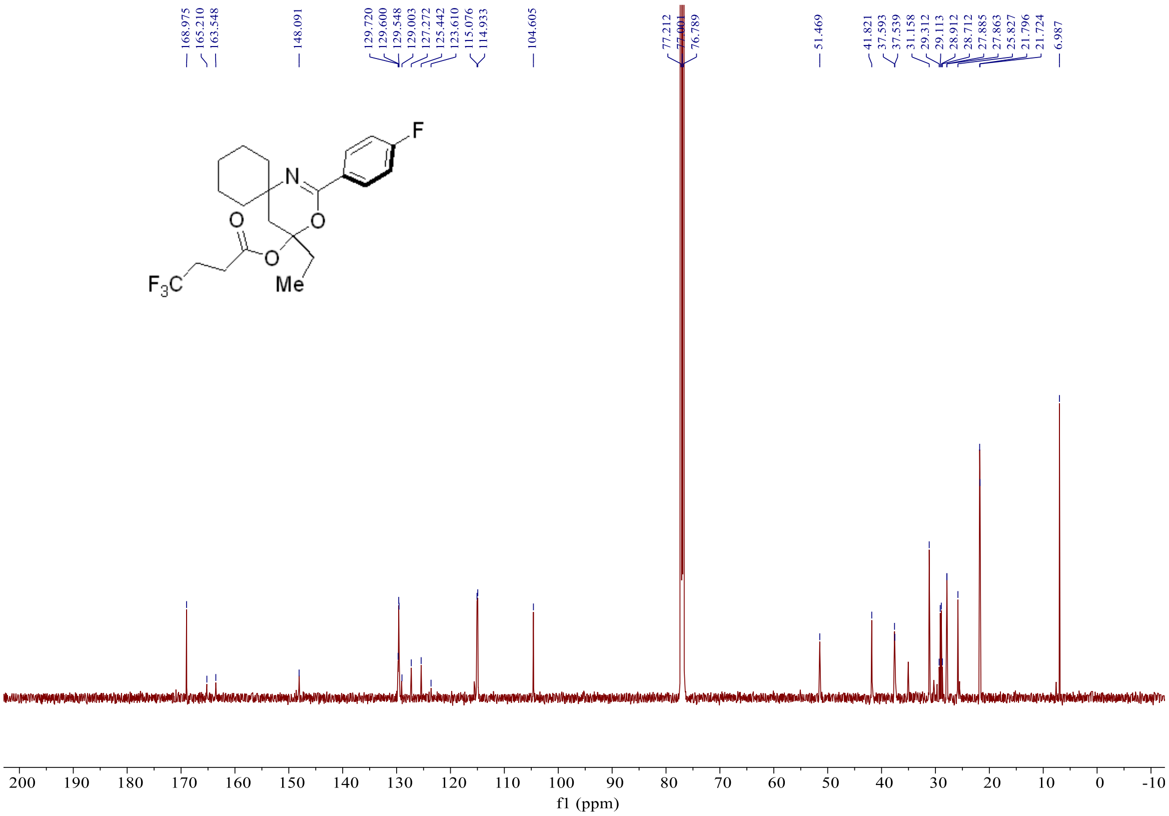

^{19}F NMR spectrum of **2ab** (377 MHz, $CDCl_3$)

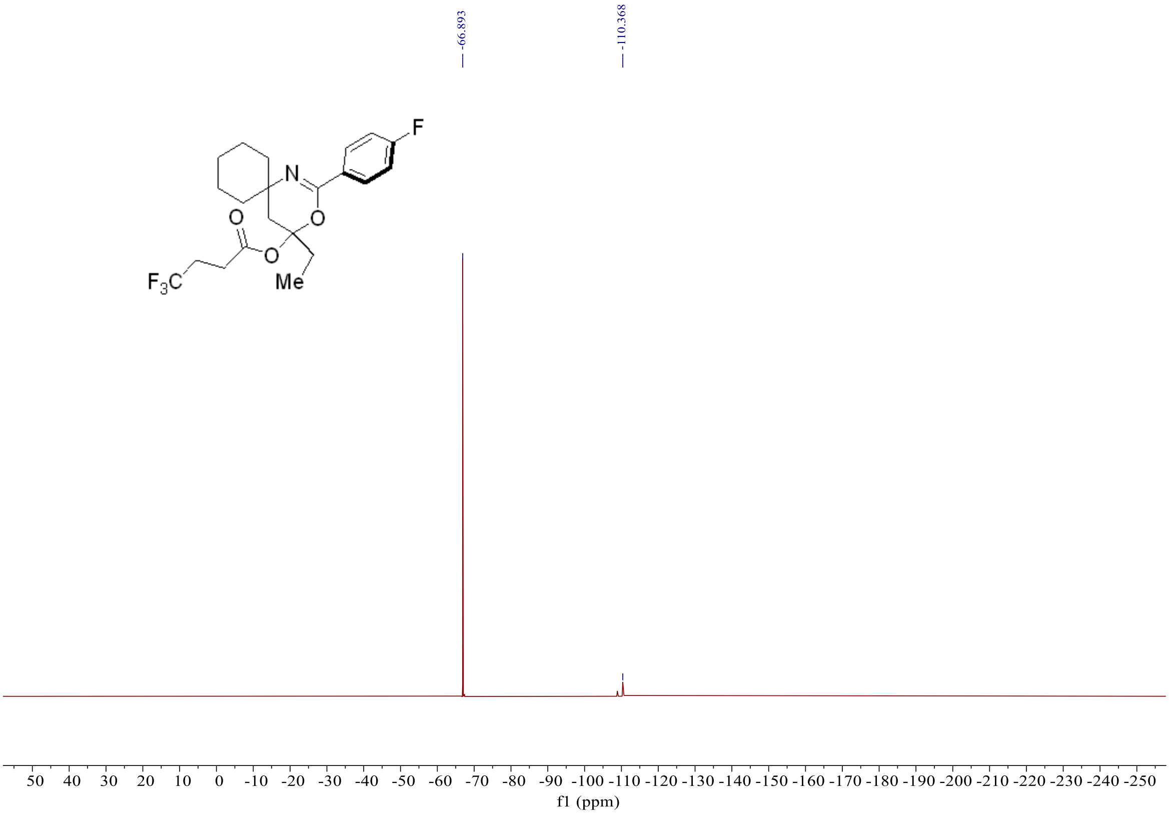

^{1}H NMR spectrum of **2ac** (600 MHz, $CDCl_3$)

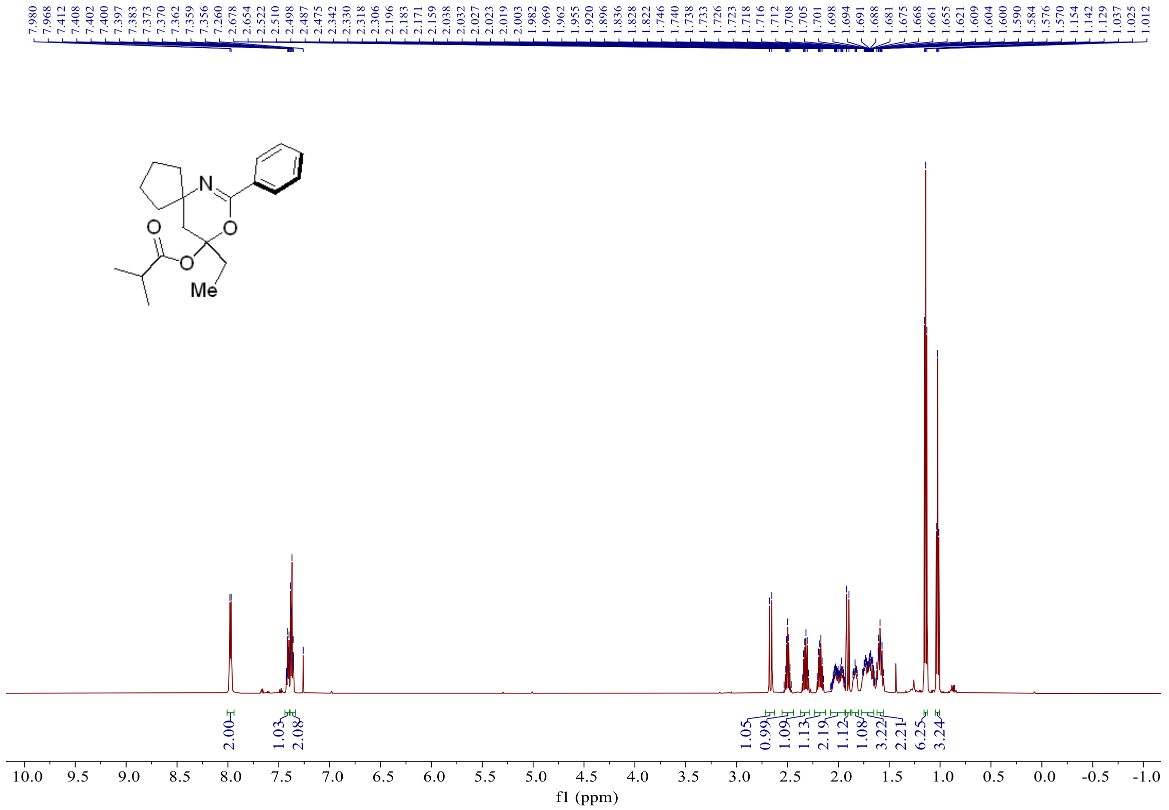

^{13}C NMR spectrum of **2ac** (151 MHz, $CDCl_3$)

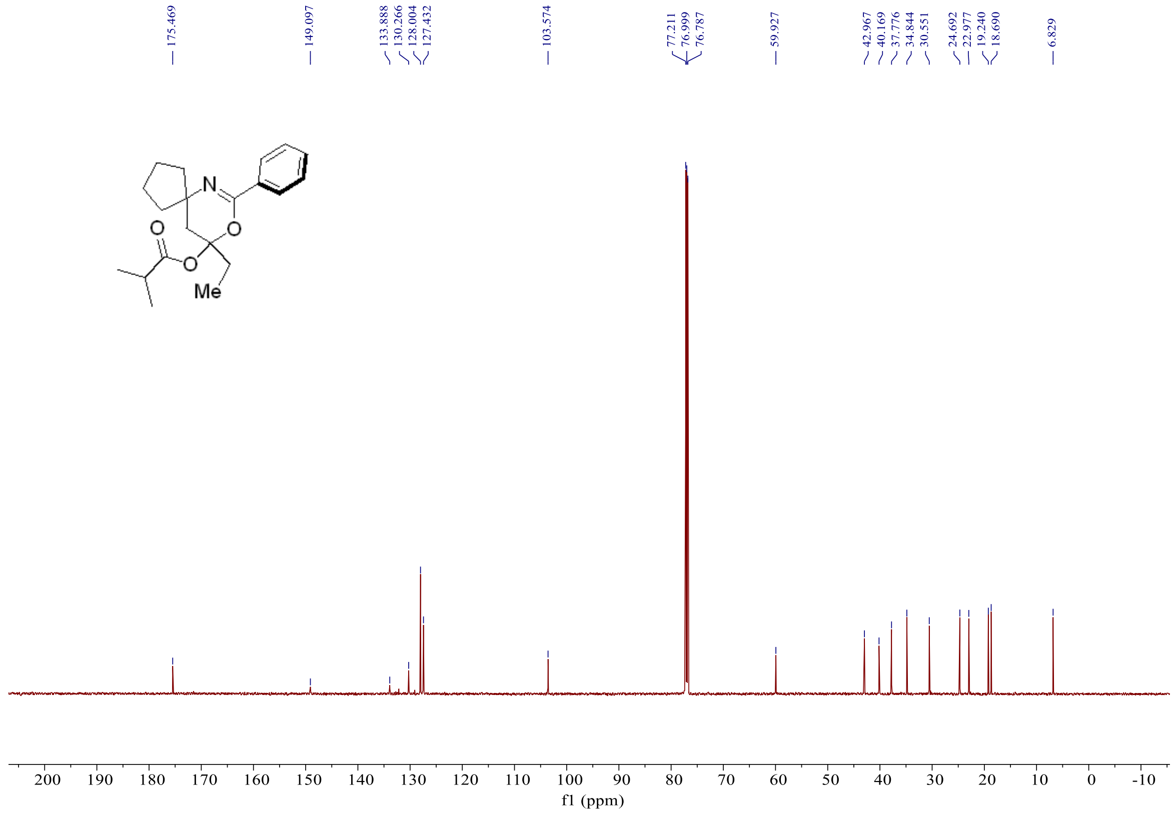

^{1}H NMR spectrum of **2ad** (600 MHz, $CDCl_3$)

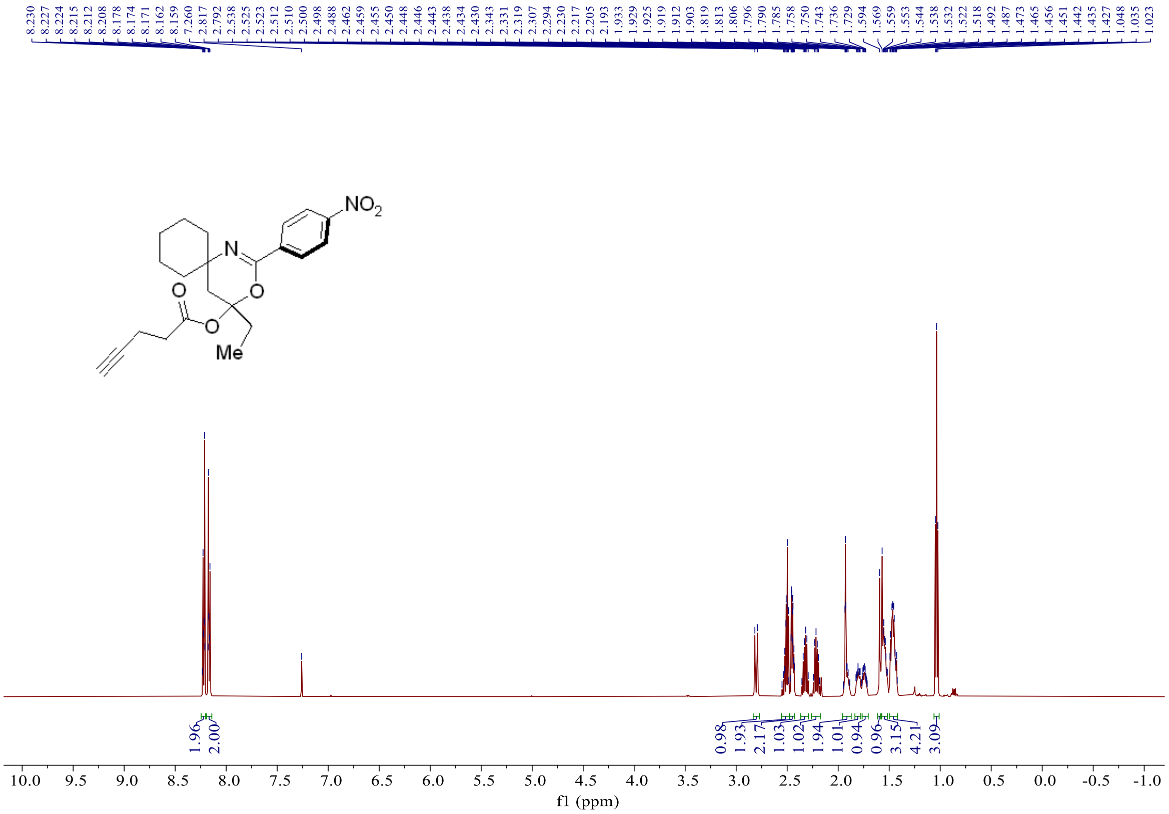

^{13}C NMR spectrum of **2ad** (151 MHz, $CDCl_3$)

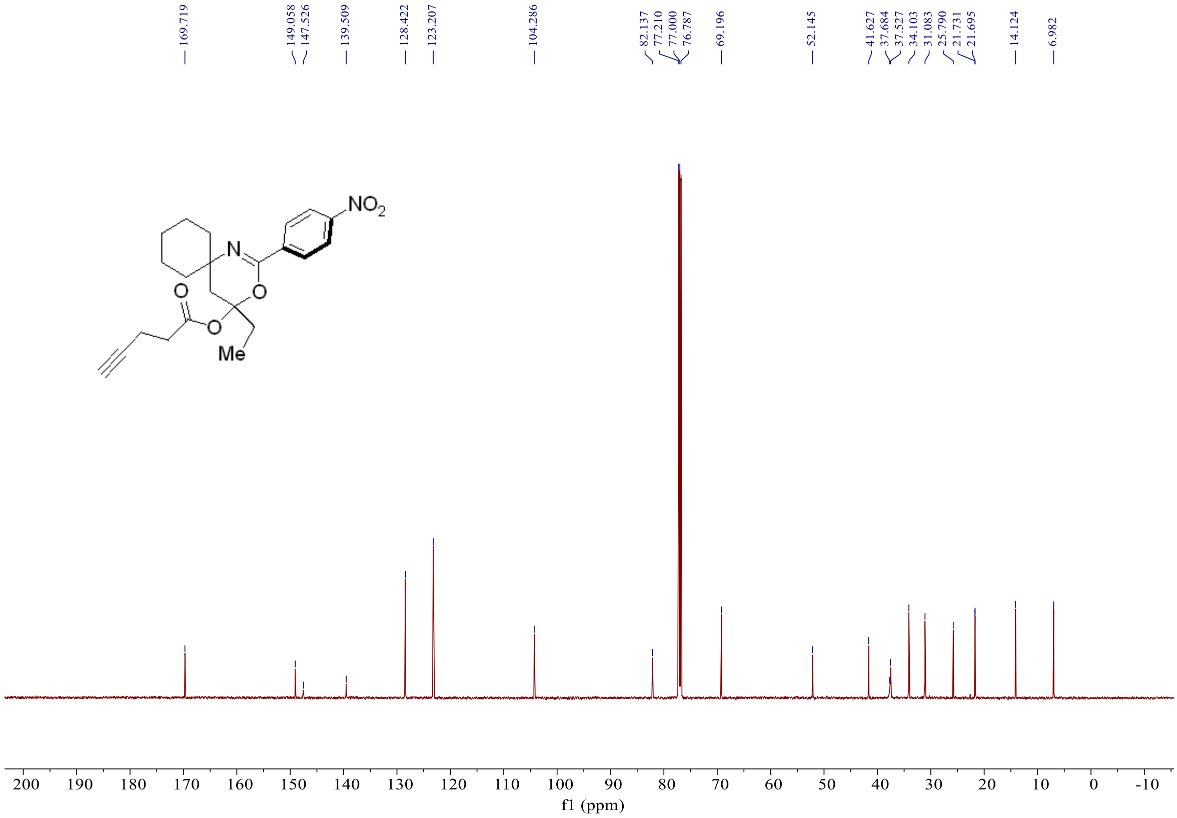

^{1}H NMR spectrum of **2ae** (500 MHz, $CDCl_3$)

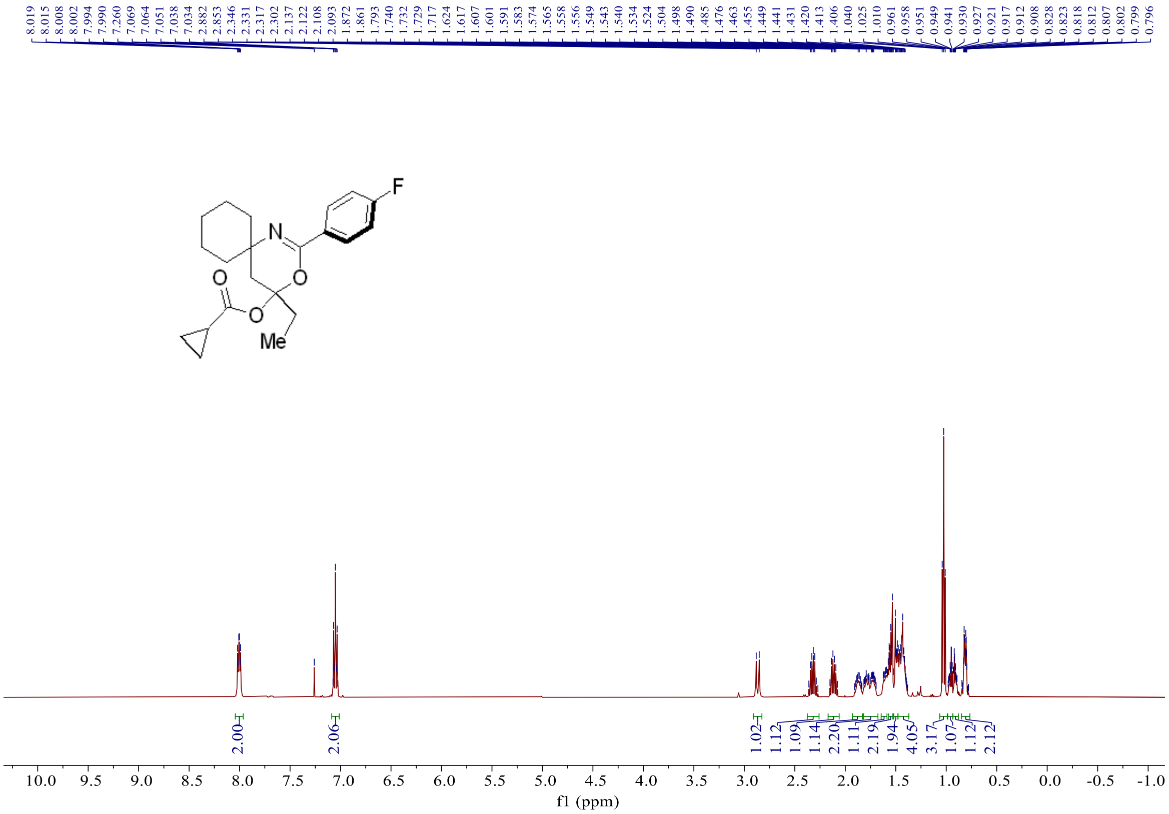

^{13}C NMR spectrum of **2ae** (126 MHz, $CDCl_3$)

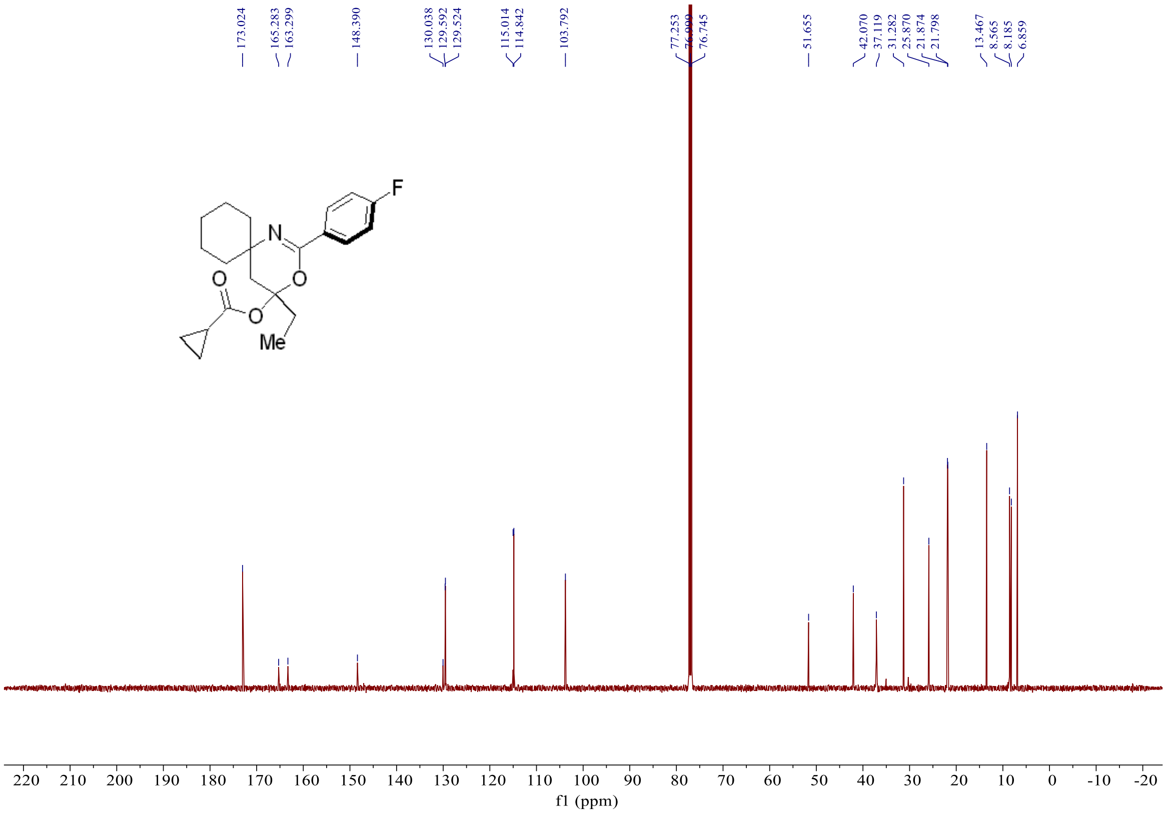

^{19}F NMR spectrum of **2ae** (377 MHz, $CDCl_3$)

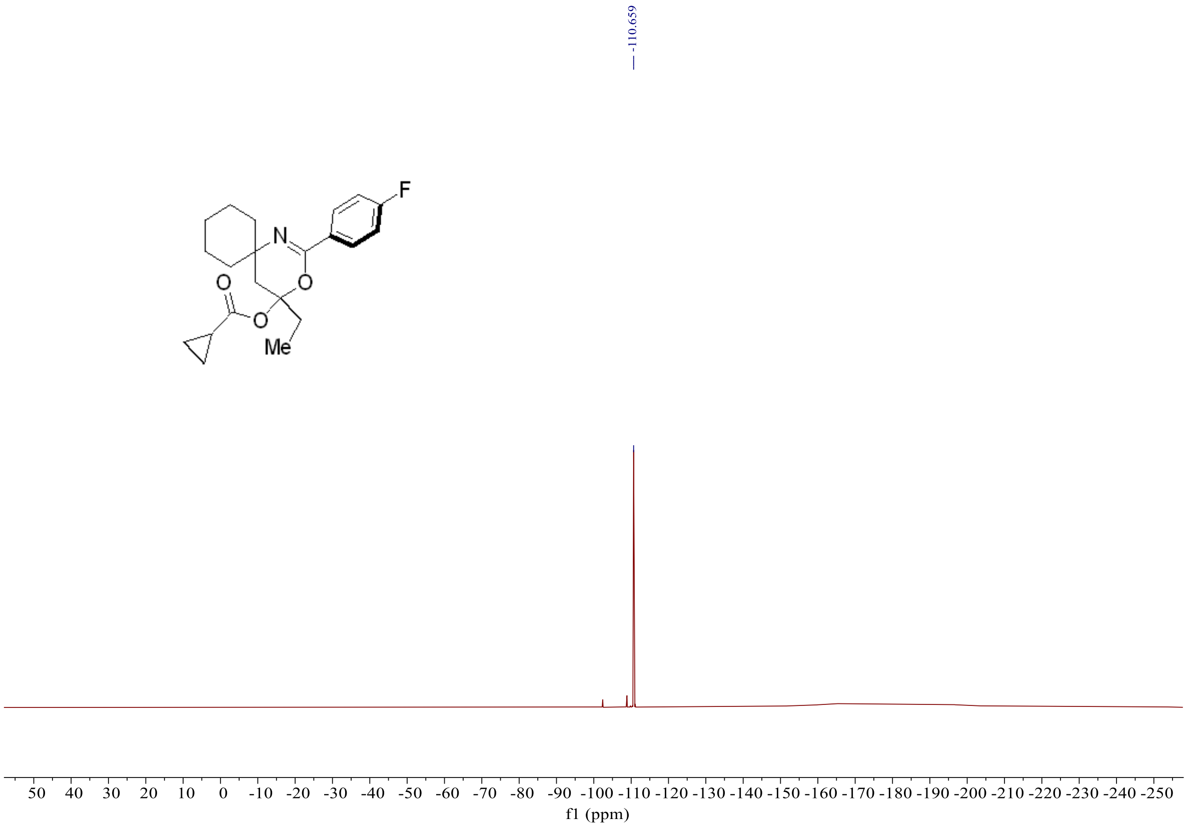

^{1}H NMR spectrum of **2af** (600 MHz, $CDCl_3$)

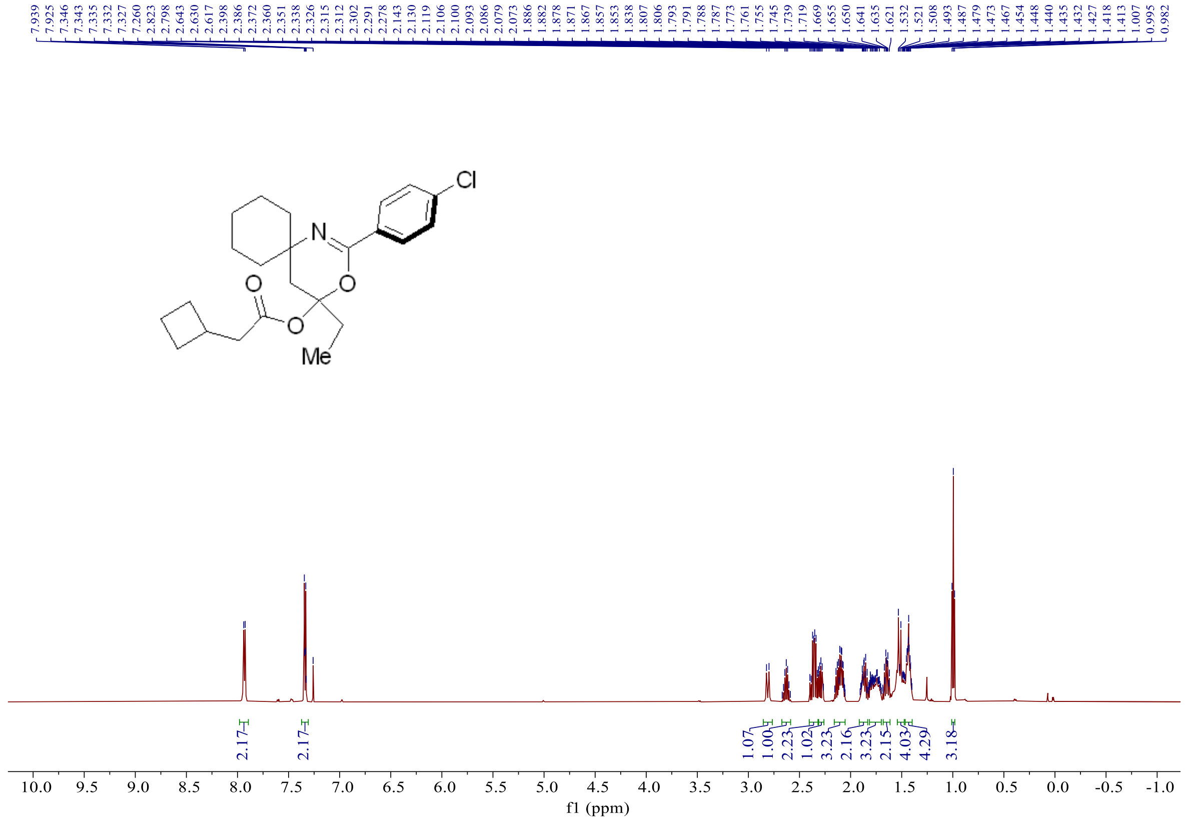

^{13}C NMR spectrum of **2af** (151 MHz, $CDCl_3$)

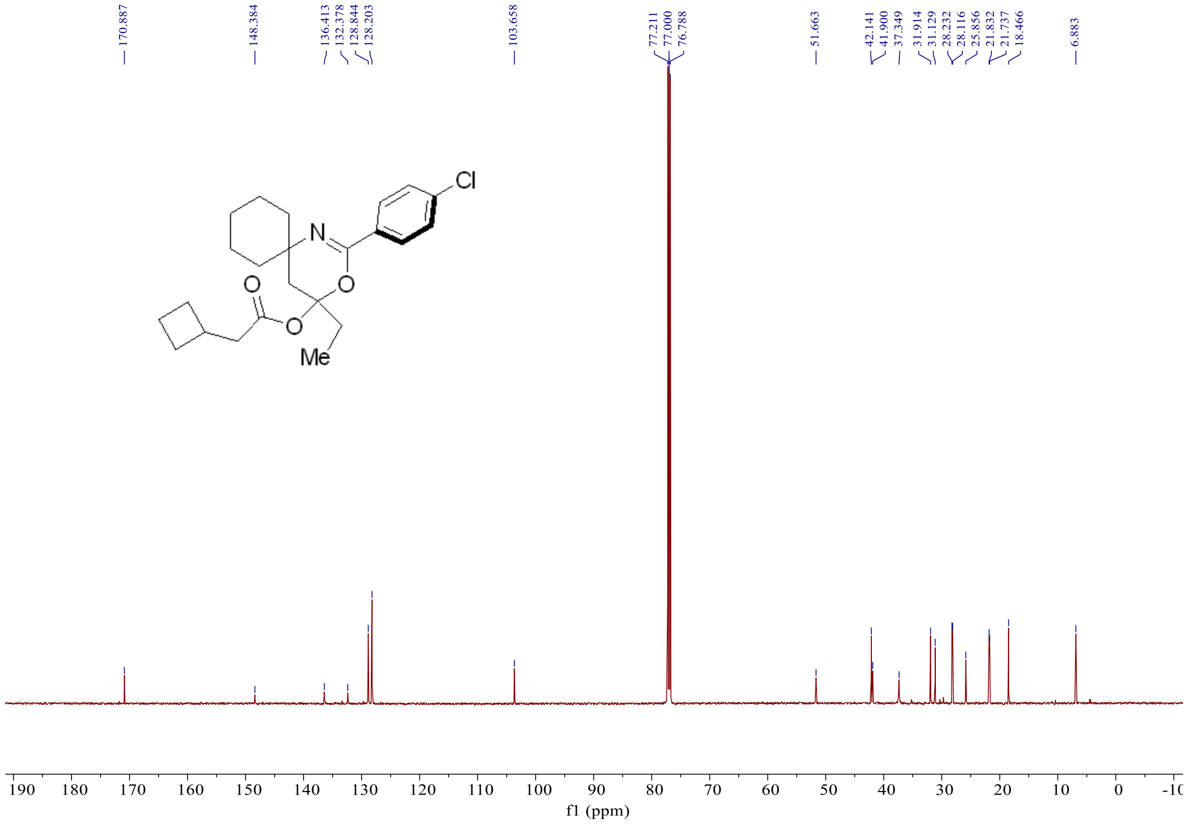

^{1}H NMR spectrum of **2ag** (600 MHz, $CDCl_3$)

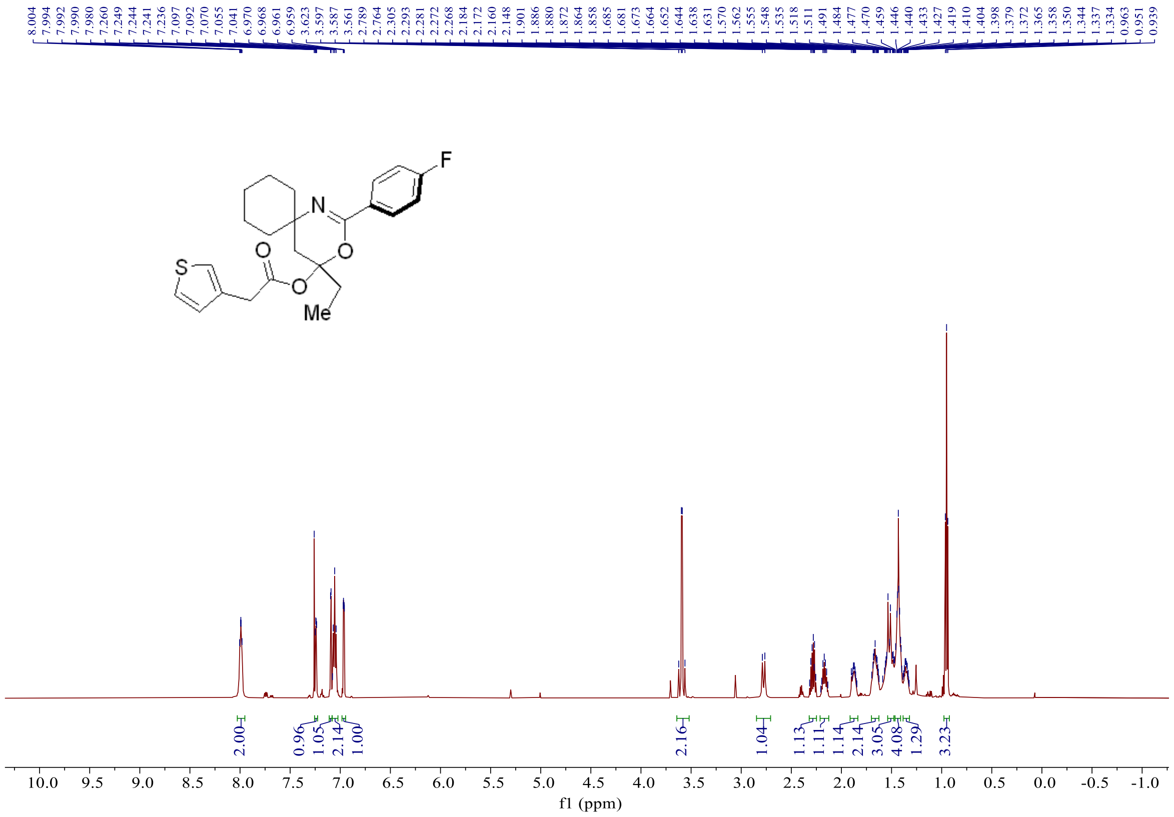

^{13}C NMR spectrum of **2ag** (151 MHz, $CDCl_3$)

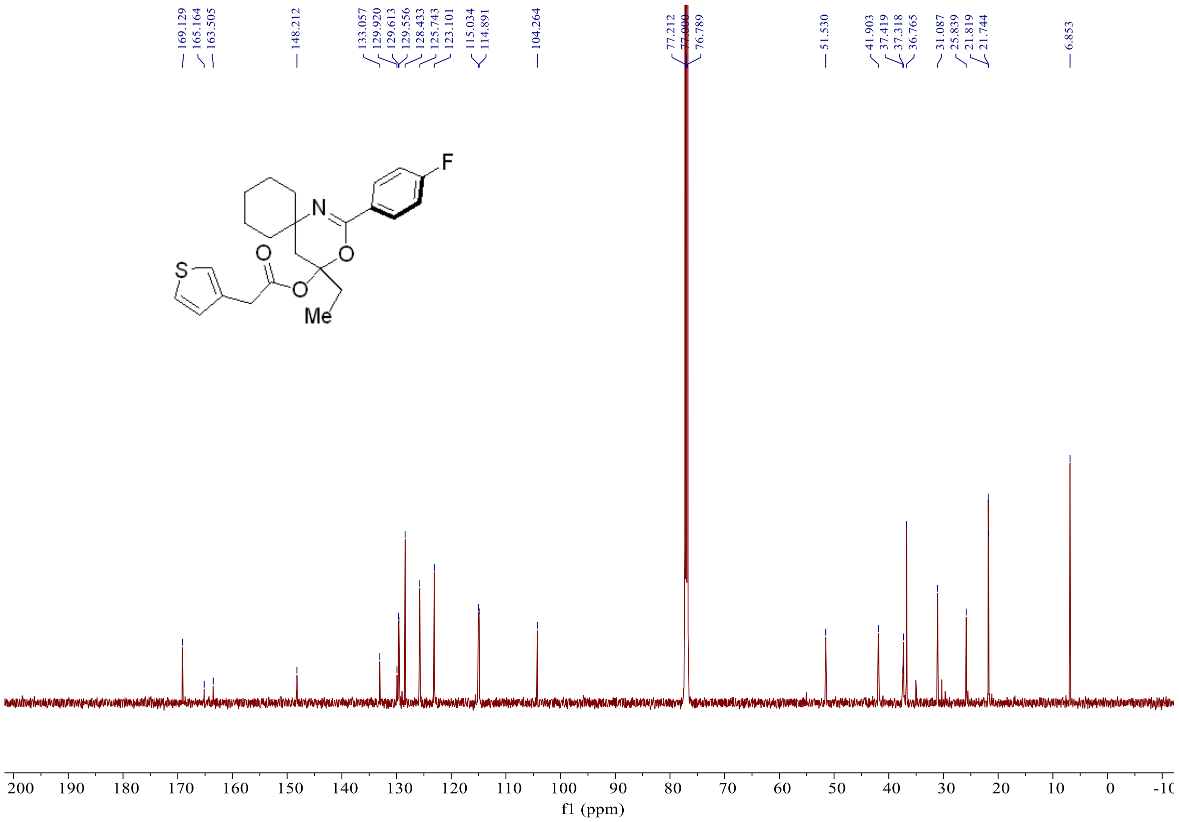

^{19}F NMR spectrum of **2ag** (377 MHz, $CDCl_3$)

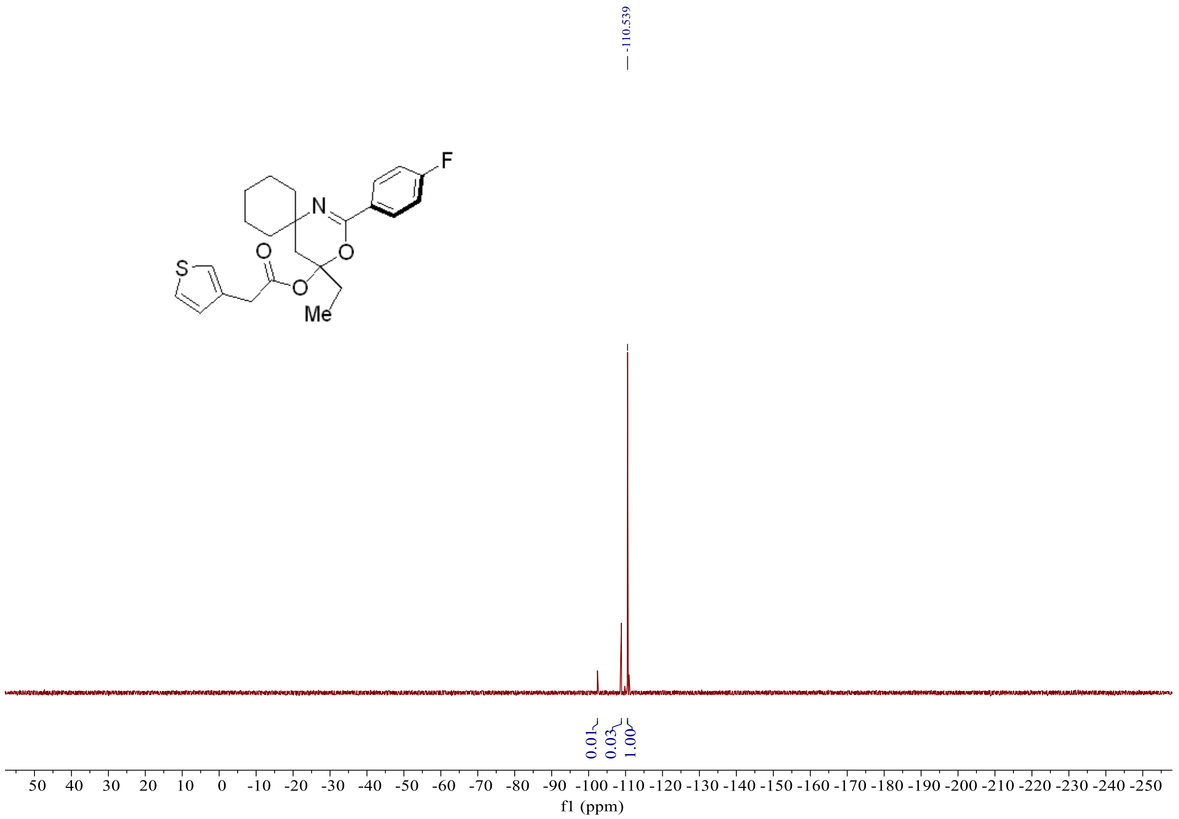

^{1}H NMR spectrum of **2ah** (500 MHz, $CDCl_3$)

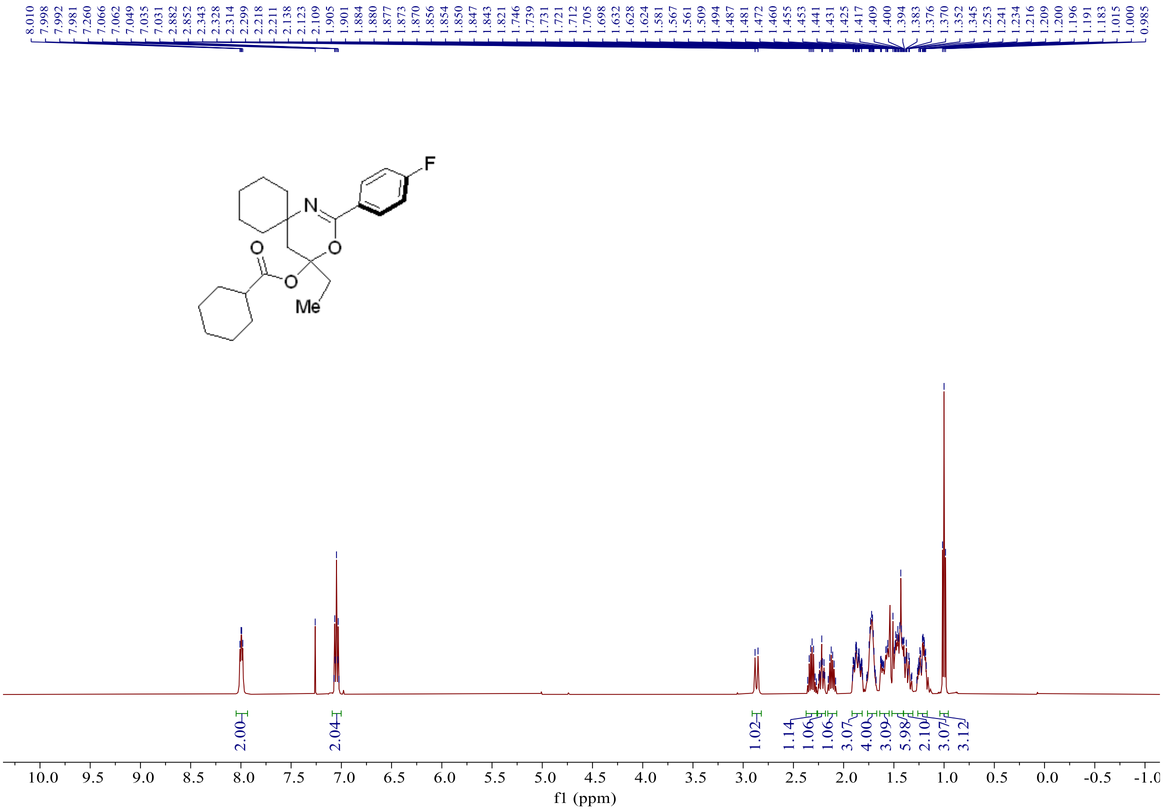

^{13}C NMR spectrum of **2ah** (126 MHz, $CDCl_3$)

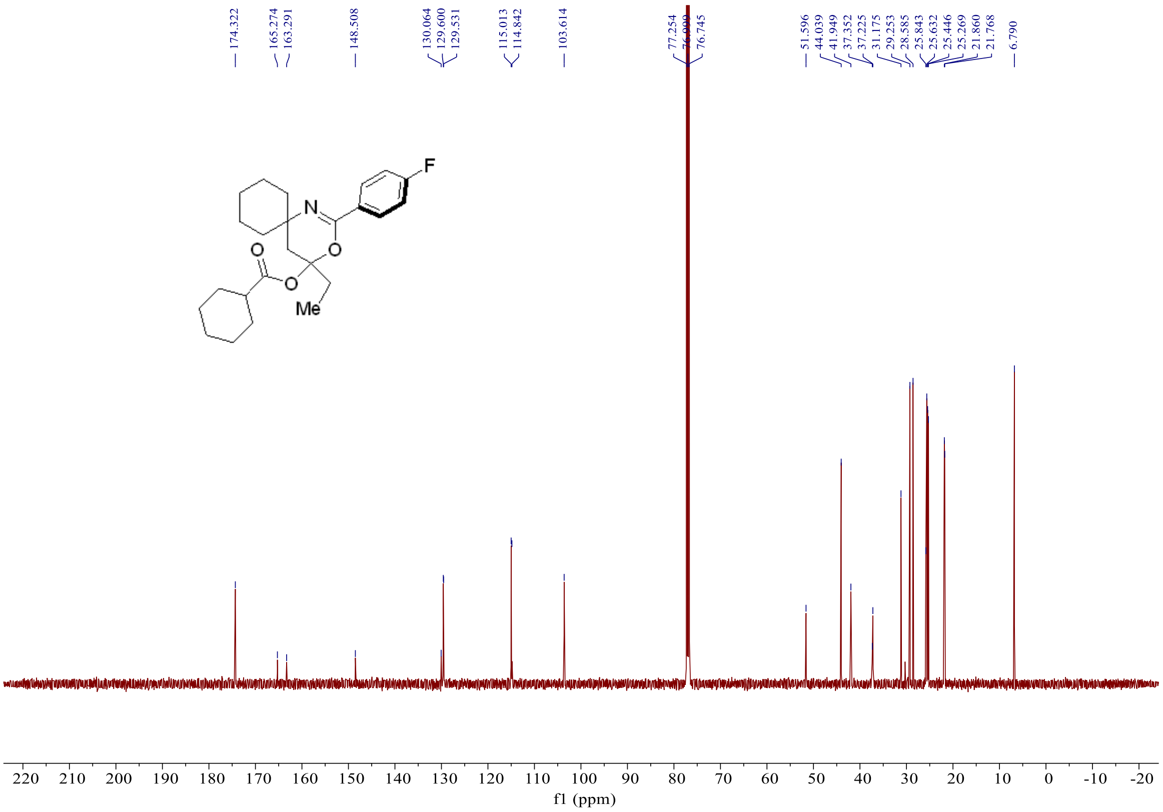

^{19}F NMR spectrum of **2ah** (377 MHz, $CDCl_3$)

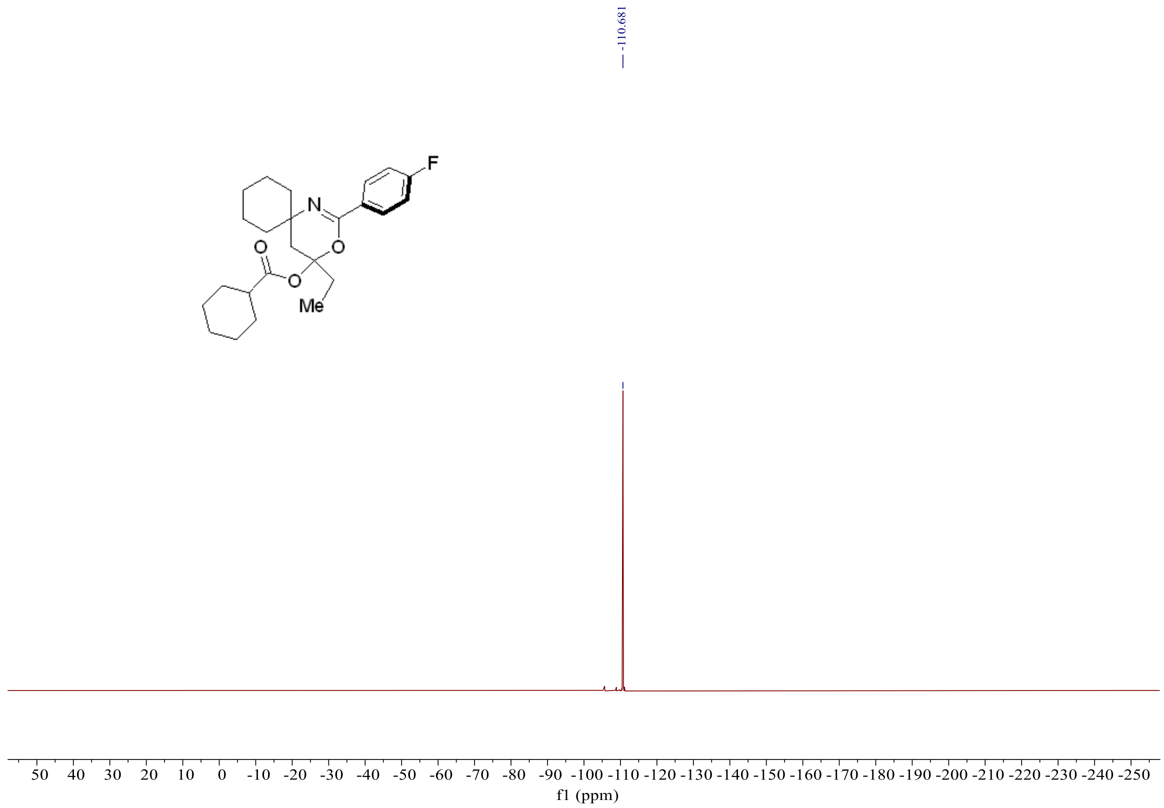

^{1}H NMR spectrum of **2ai** (500 MHz, $CDCl_3$)

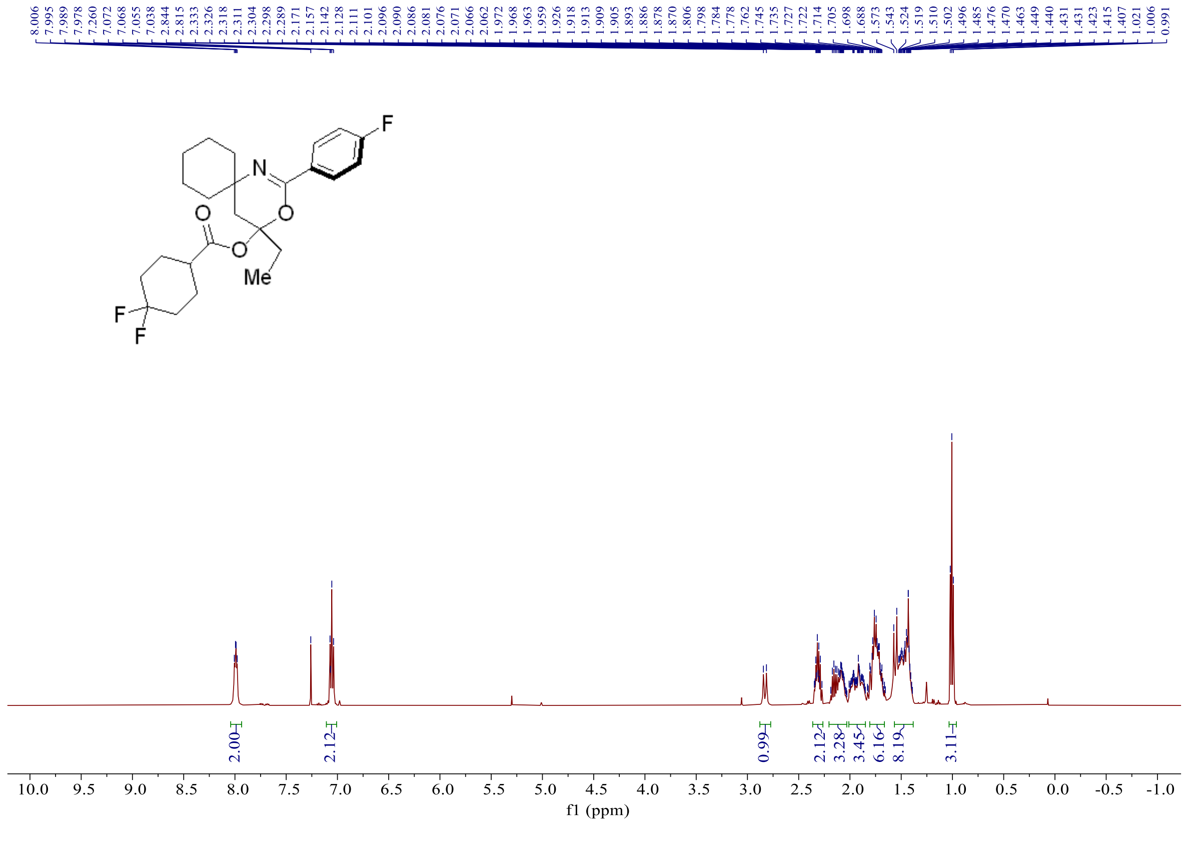

^{13}C NMR spectrum of **2ai** (126 MHz, $CDCl_3$)

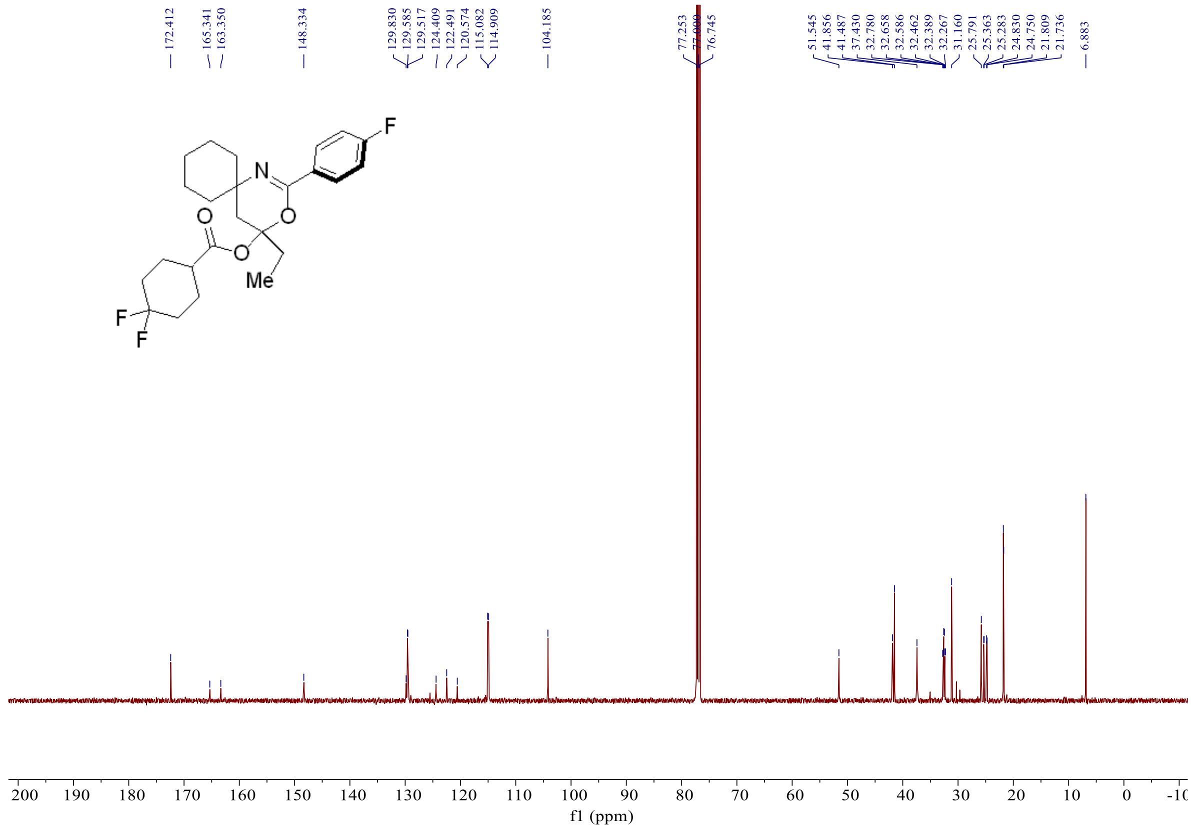

^{19}F NMR spectrum of **2ai** (377 MHz, $CDCl_3$)

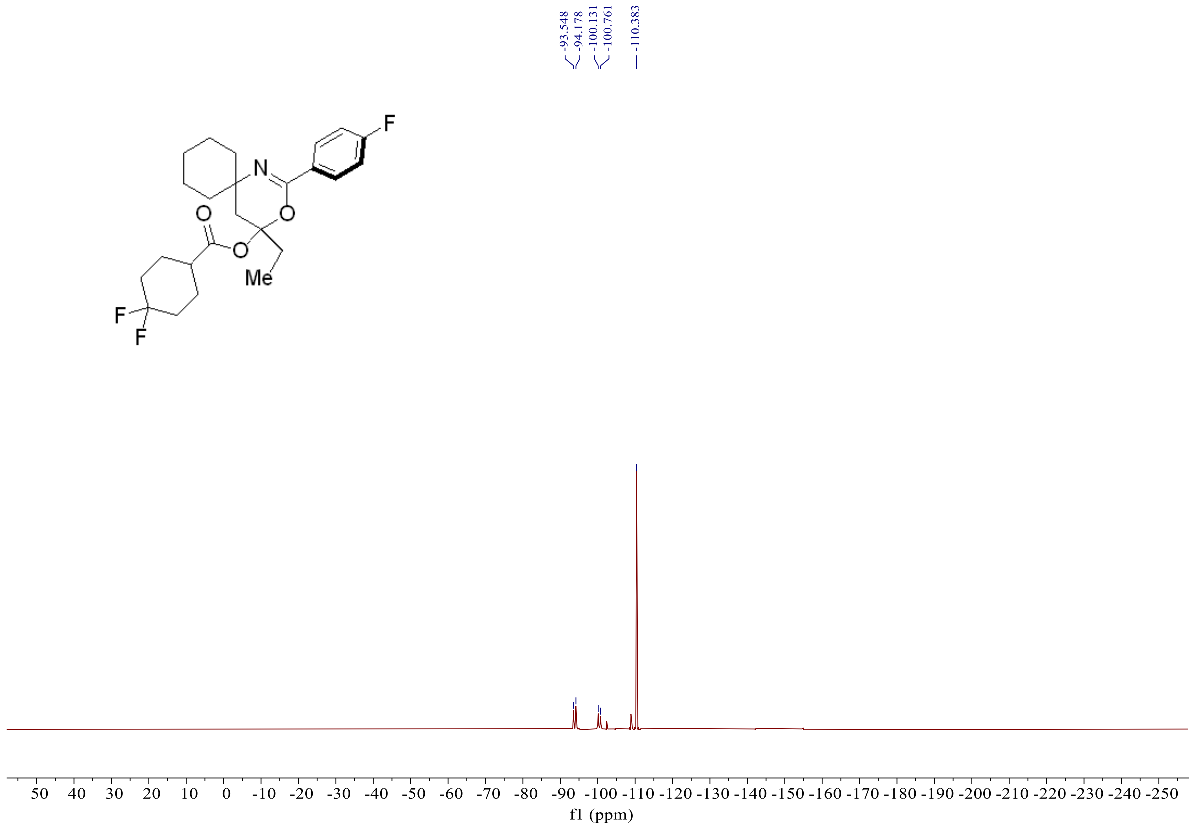

^{1}H NMR spectrum of **2aj** (500 MHz, $CDCl_3$)

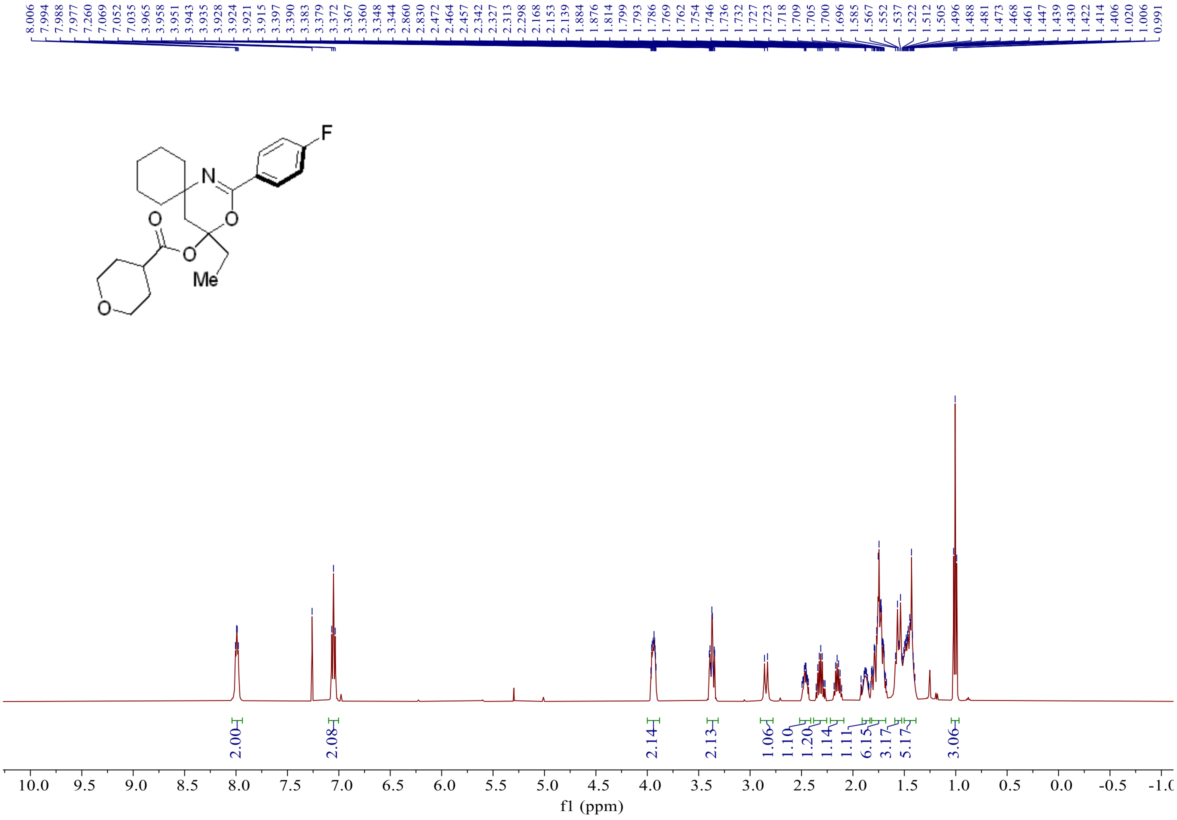

^{13}C NMR spectrum of **2aj** (126 MHz, $CDCl_3$)

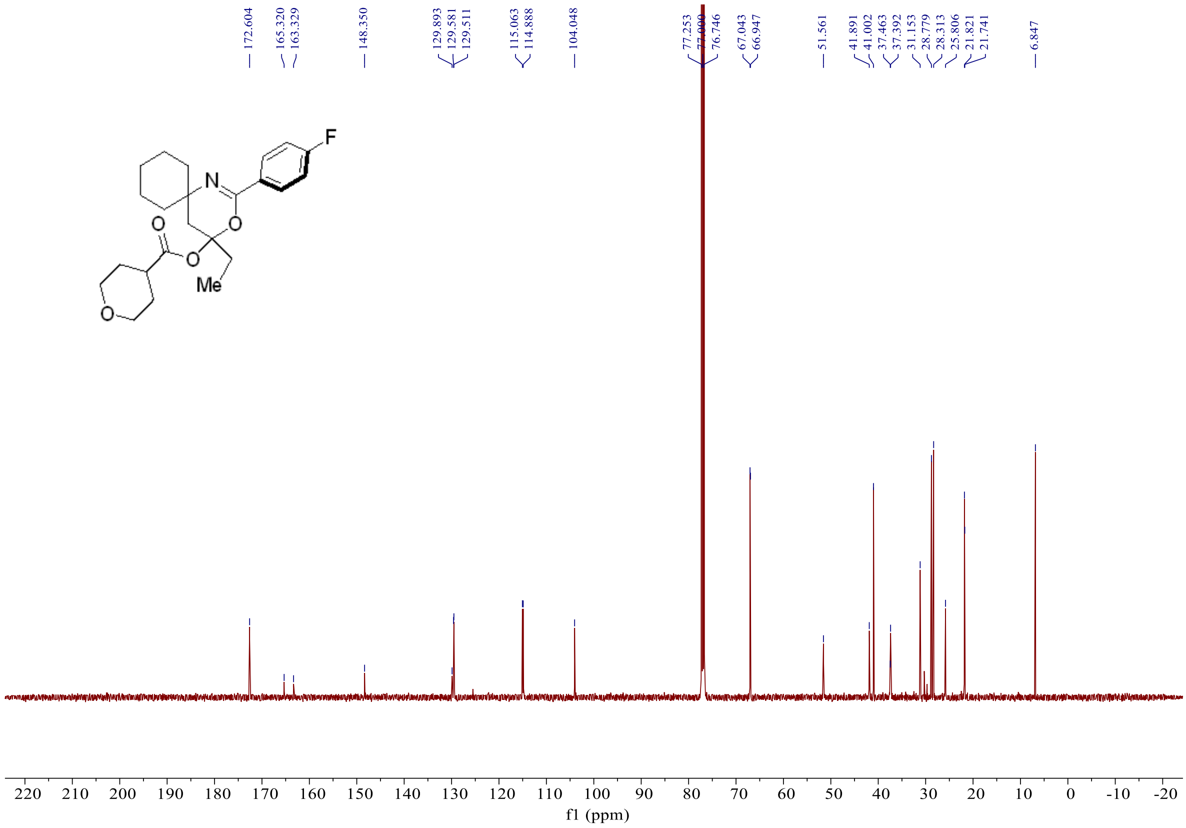

^{19}F NMR spectrum of **2aj** (377 MHz, $CDCl_3$)

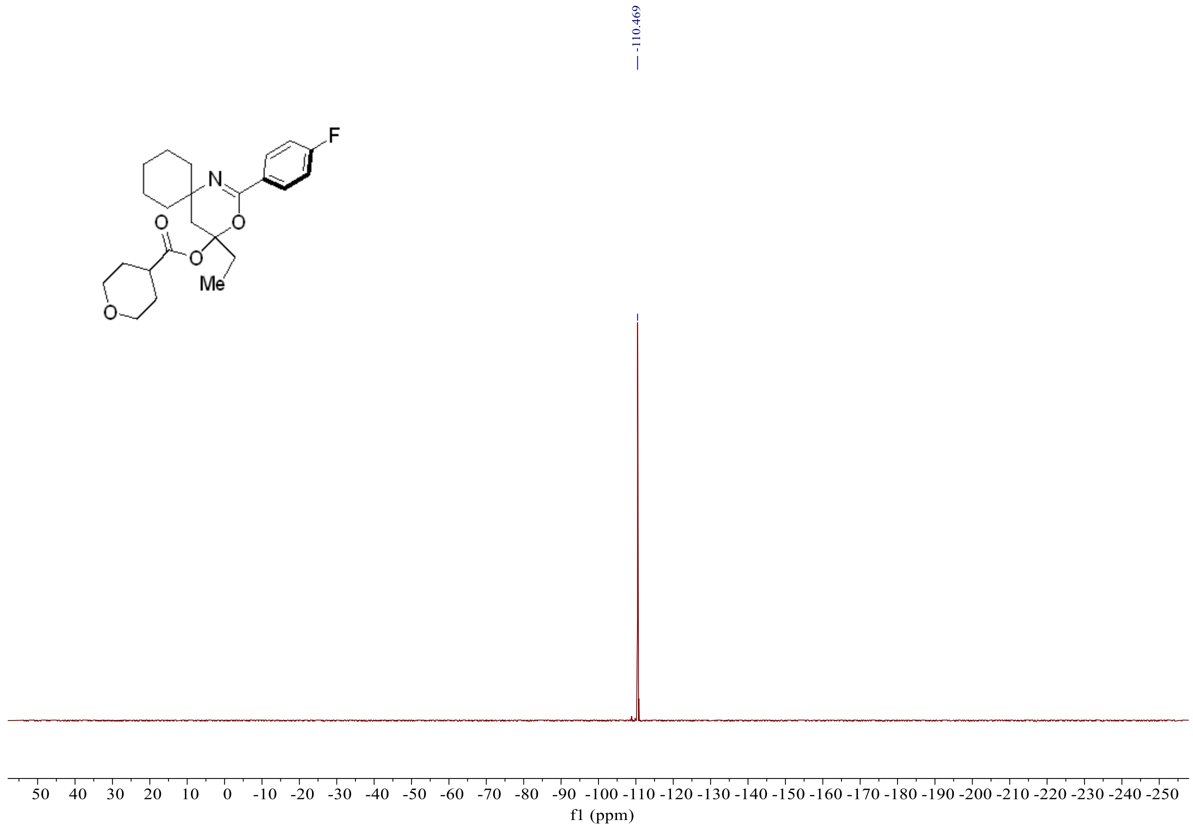

^{1}H NMR spectrum of **2ak** (400 MHz, $CDCl_3$)

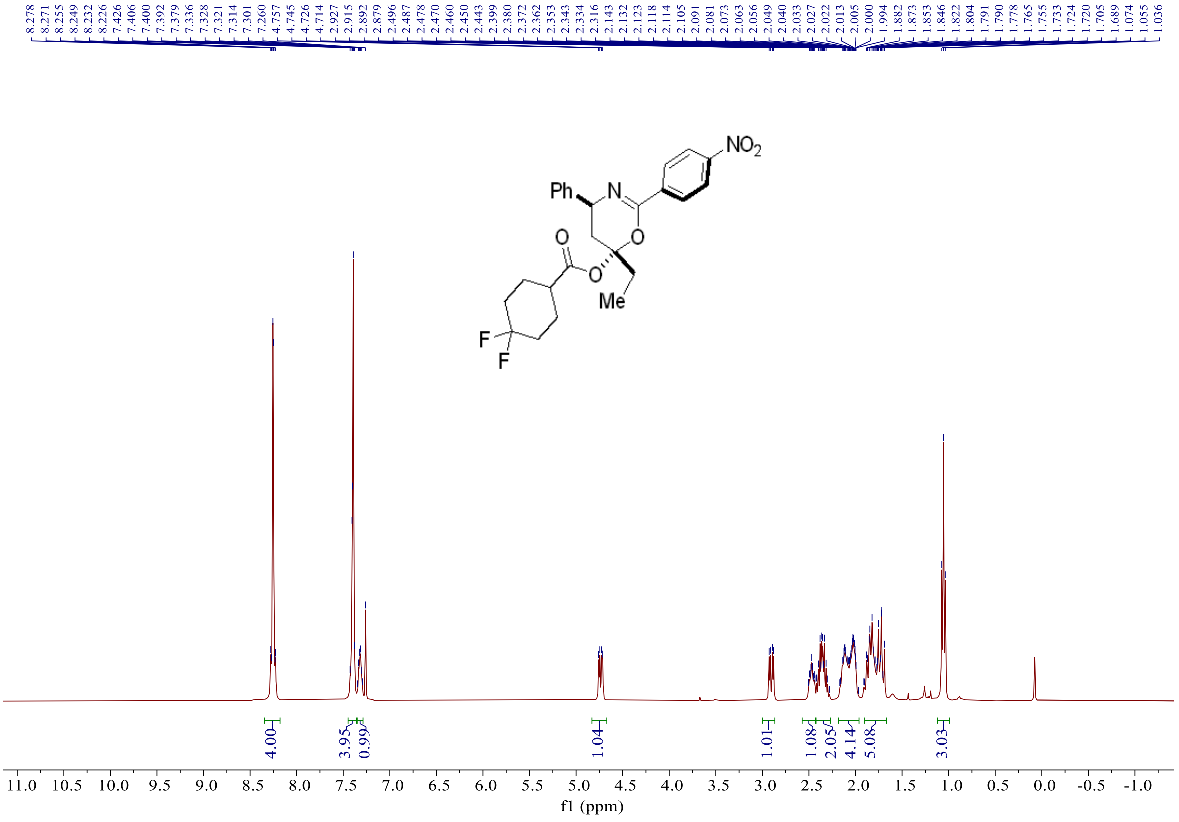

^{13}C NMR spectrum of **2ak** (151 MHz, $CDCl_3$)

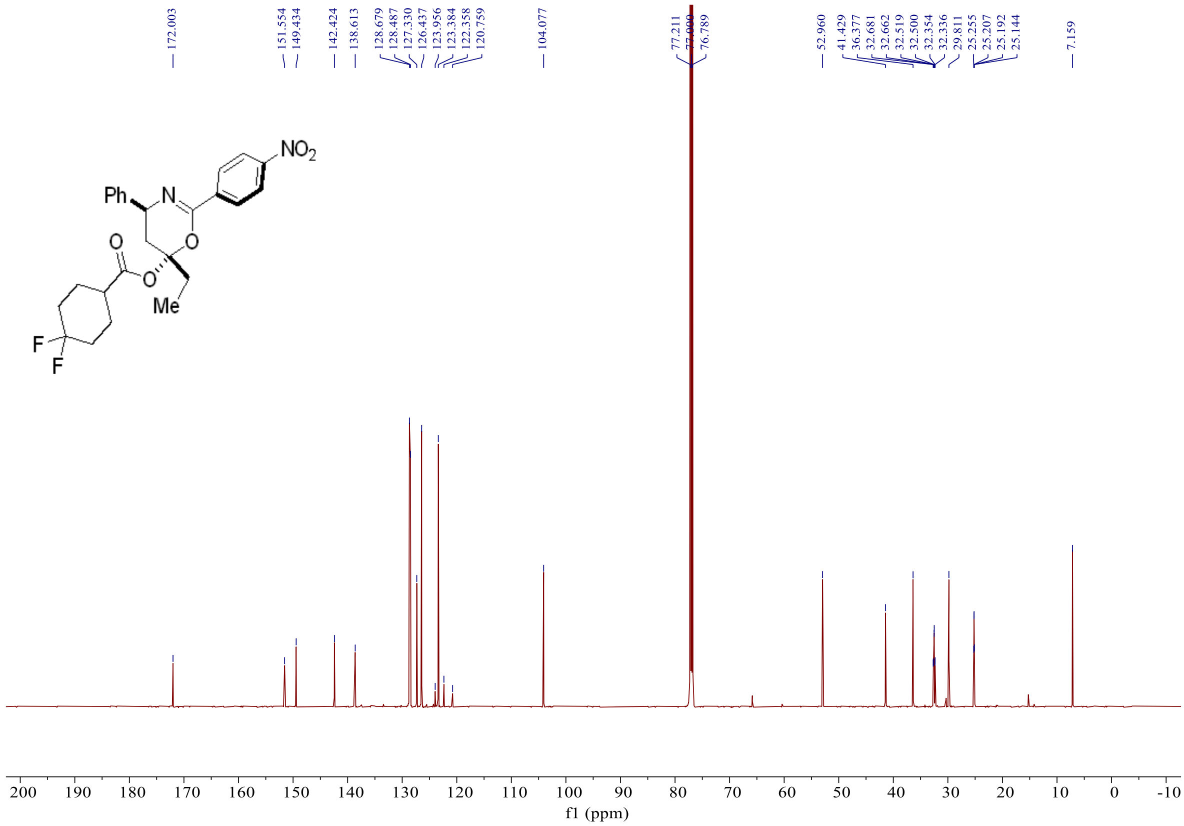

^{19}F NMR spectrum of **2ak** (377 MHz, $CDCl_3$)

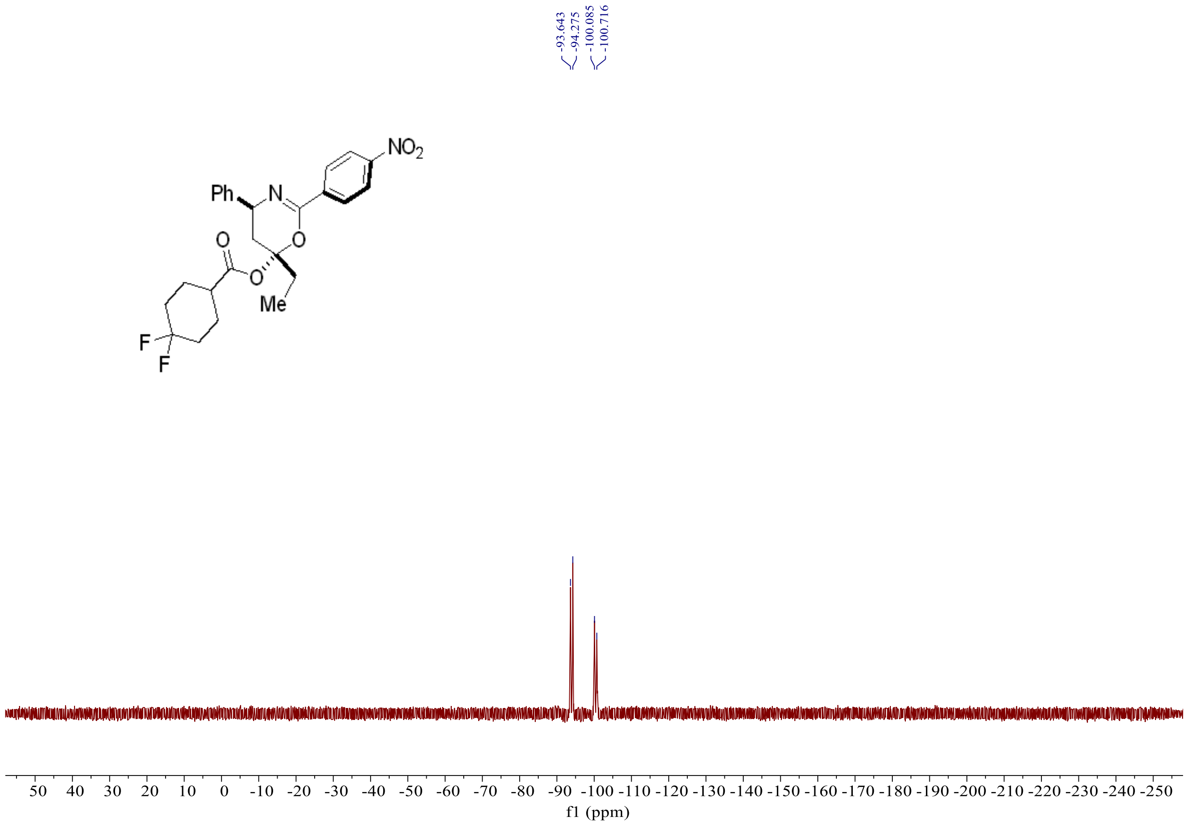

^{1}H NMR spectrum of [^{13}C]-**2j** (600 MHz, $CDCl_3$)

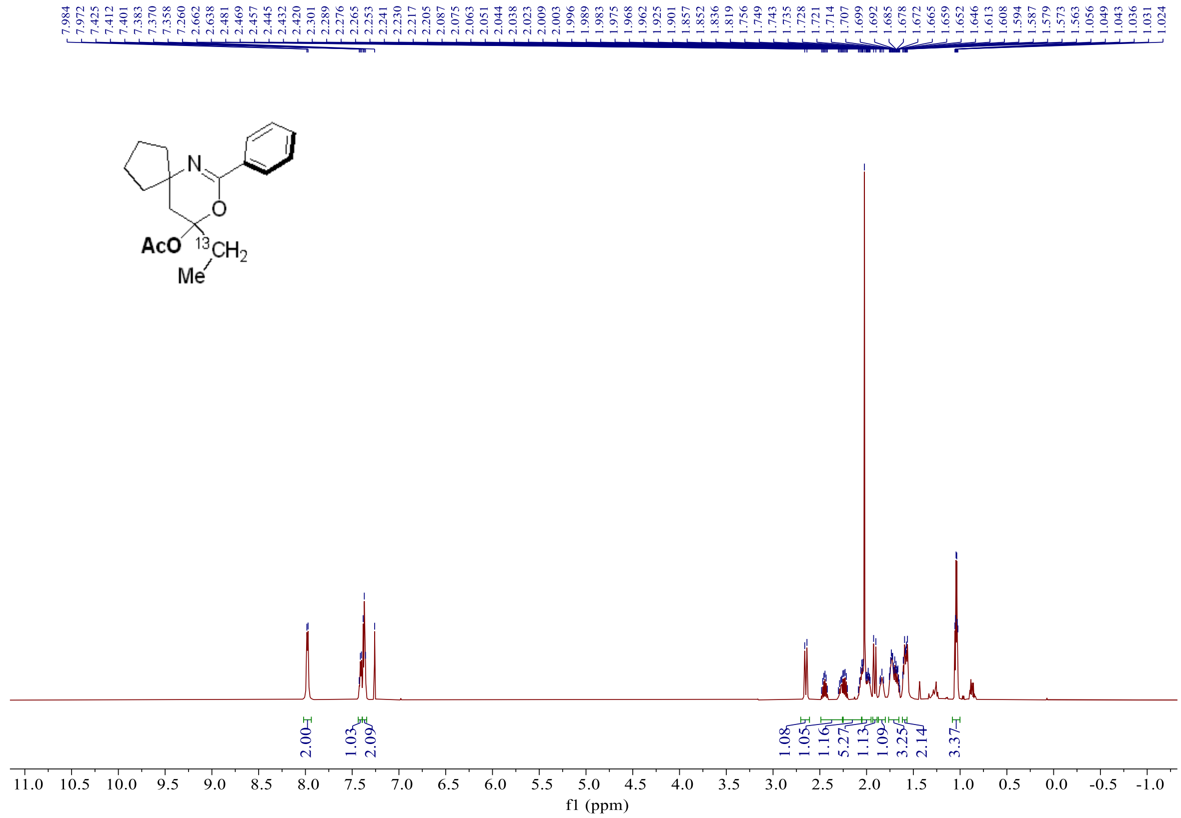

^{13}C NMR spectrum of [^{13}C]-**2j** (151 MHz, $CDCl_3$)

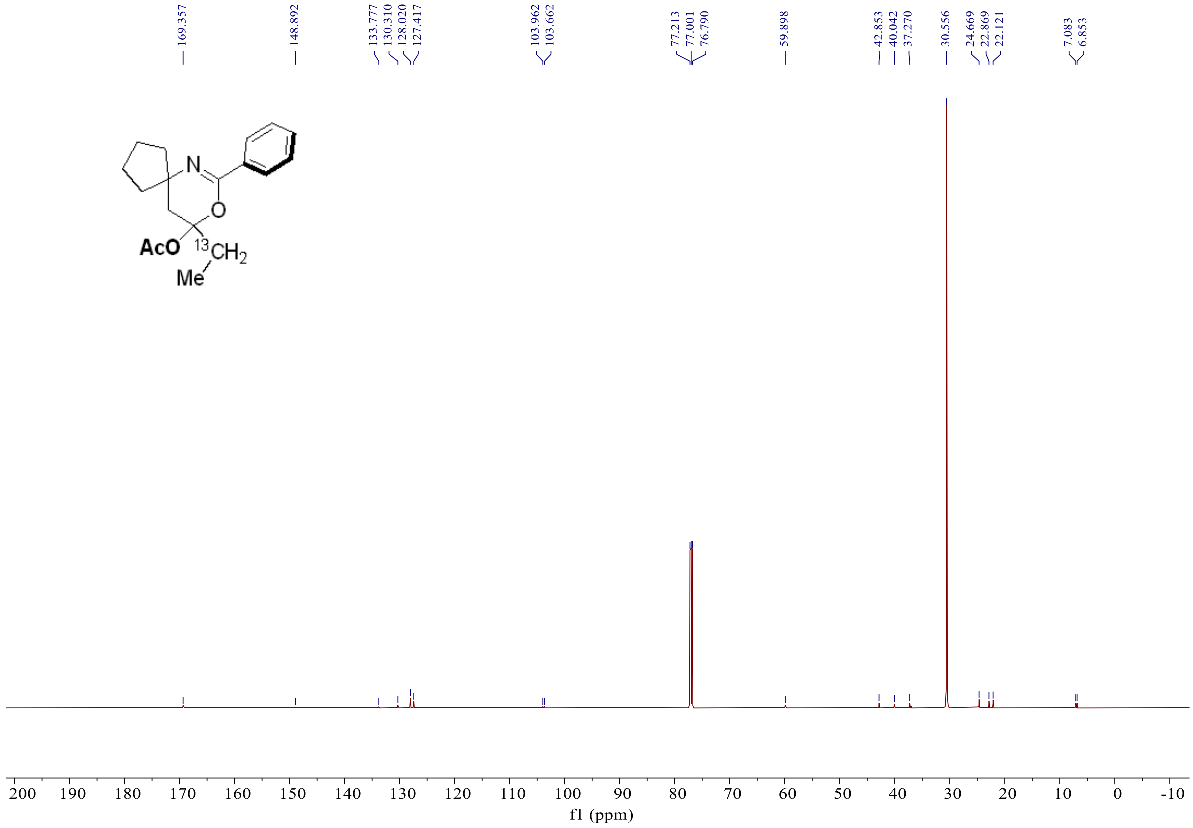

^{1}H NMR spectrum of **3** (500 MHz, $CDCl_3$)

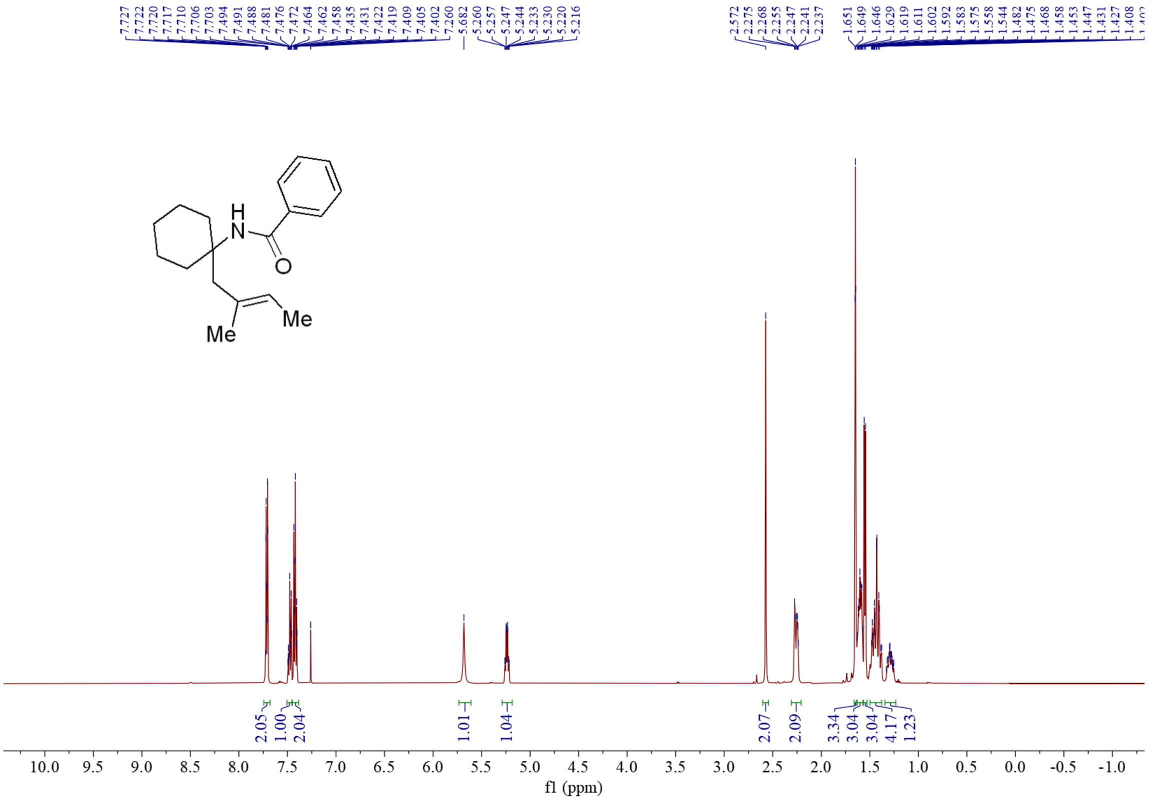

^{13}C NMR spectrum of **3** (126 MHz, $CDCl_3$)

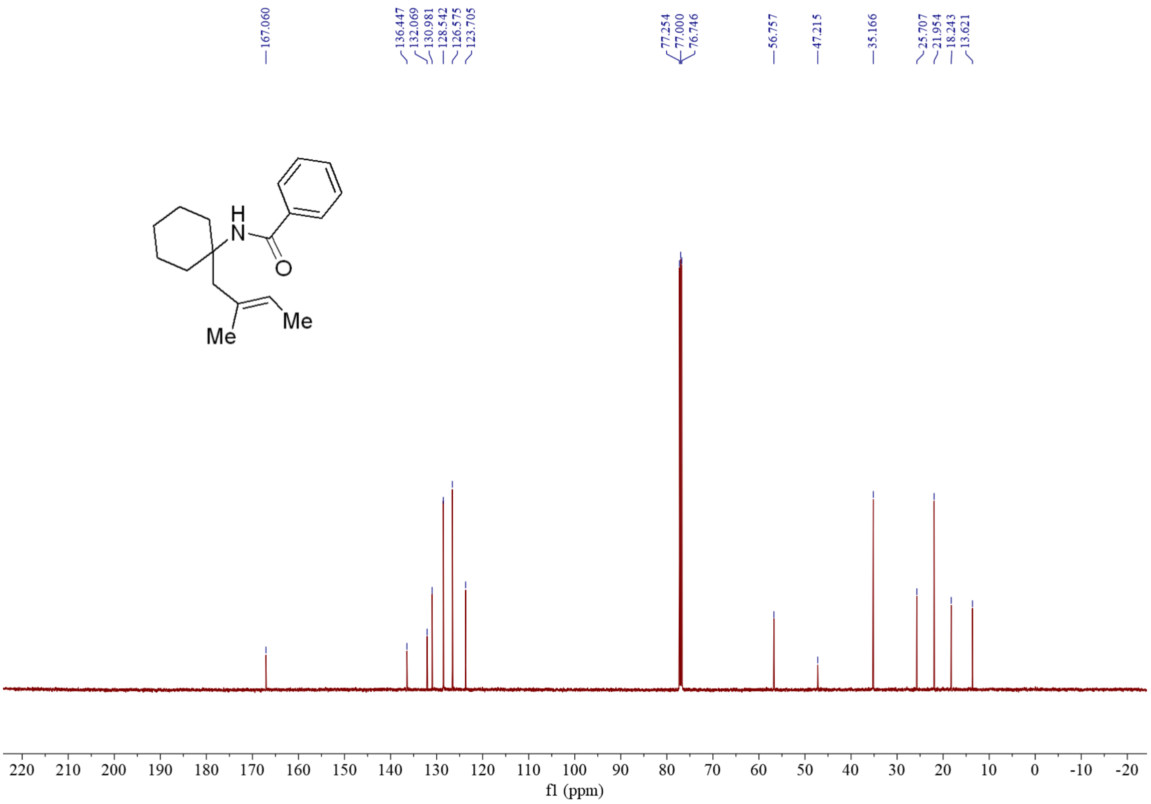

^{1}H NMR spectrum of **4** (500 MHz, $CDCl_3$)

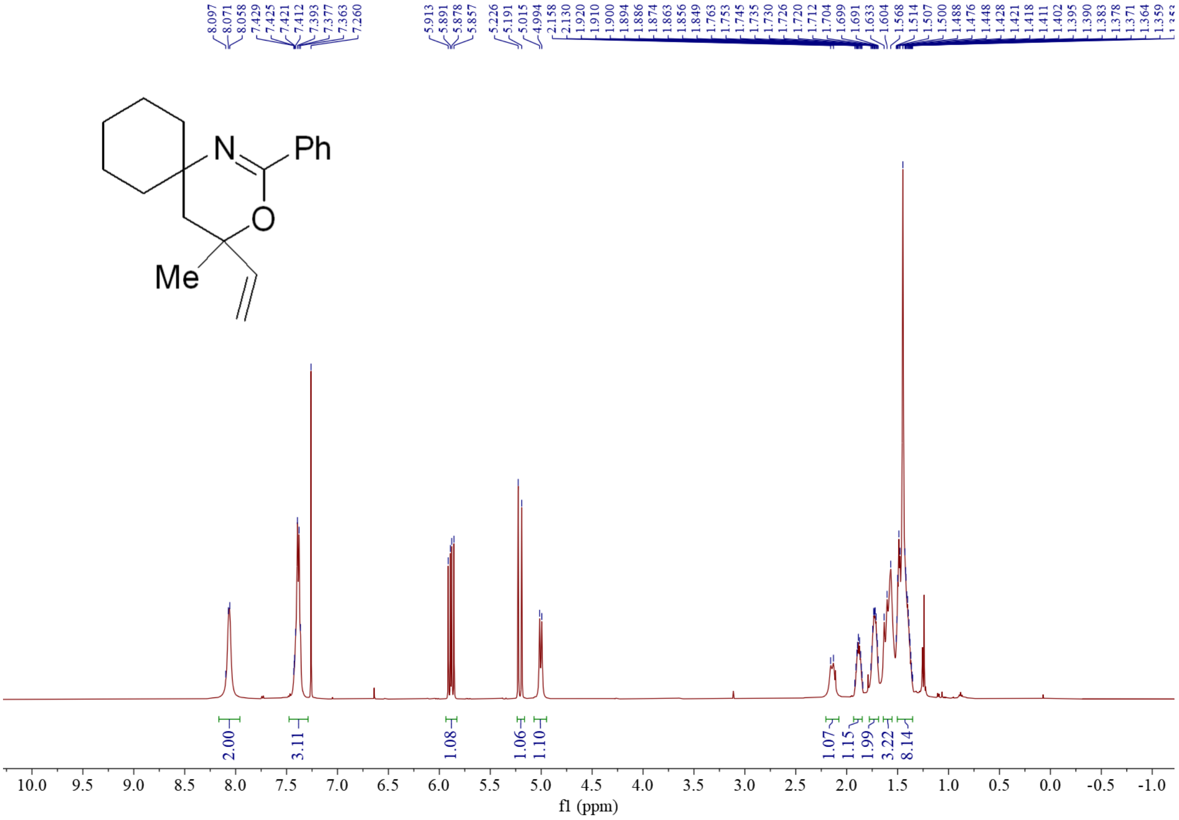

^{13}C NMR spectrum of **4** (126 MHz, $CDCl_3$)

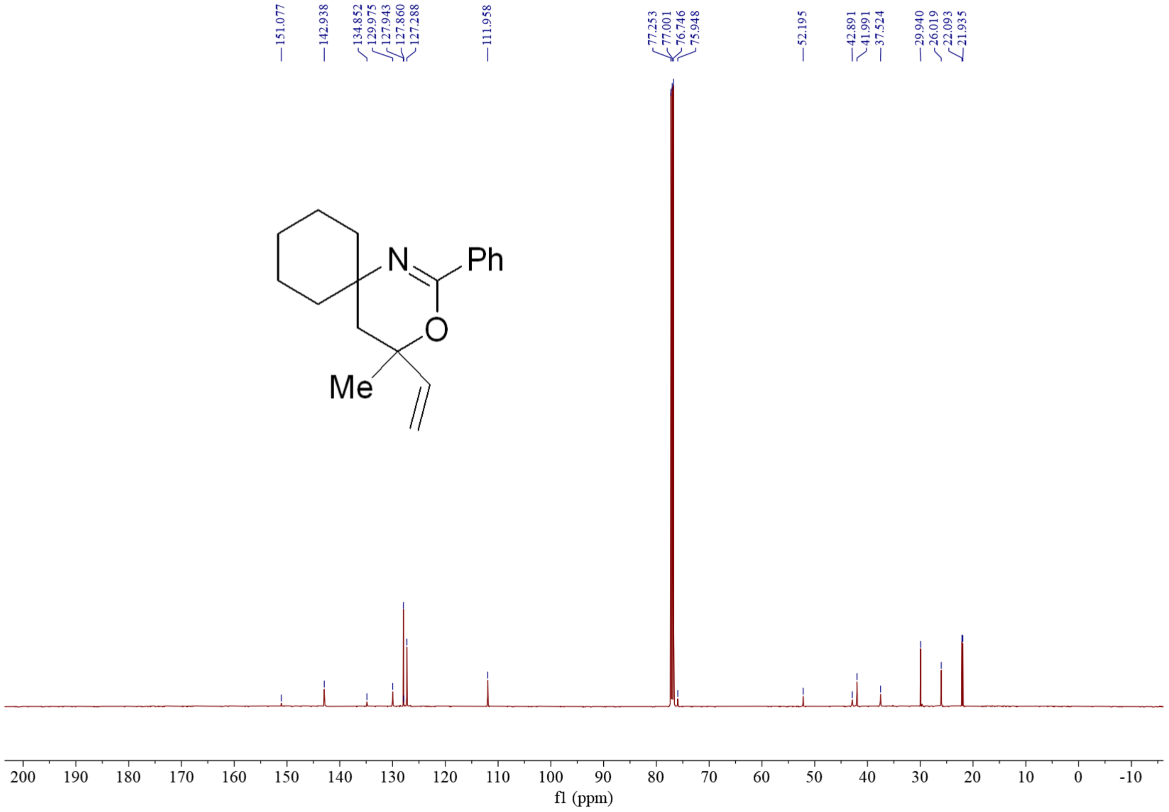

^{1}H NMR spectrum of **5** (400 MHz, Acetonitrile-d_3)

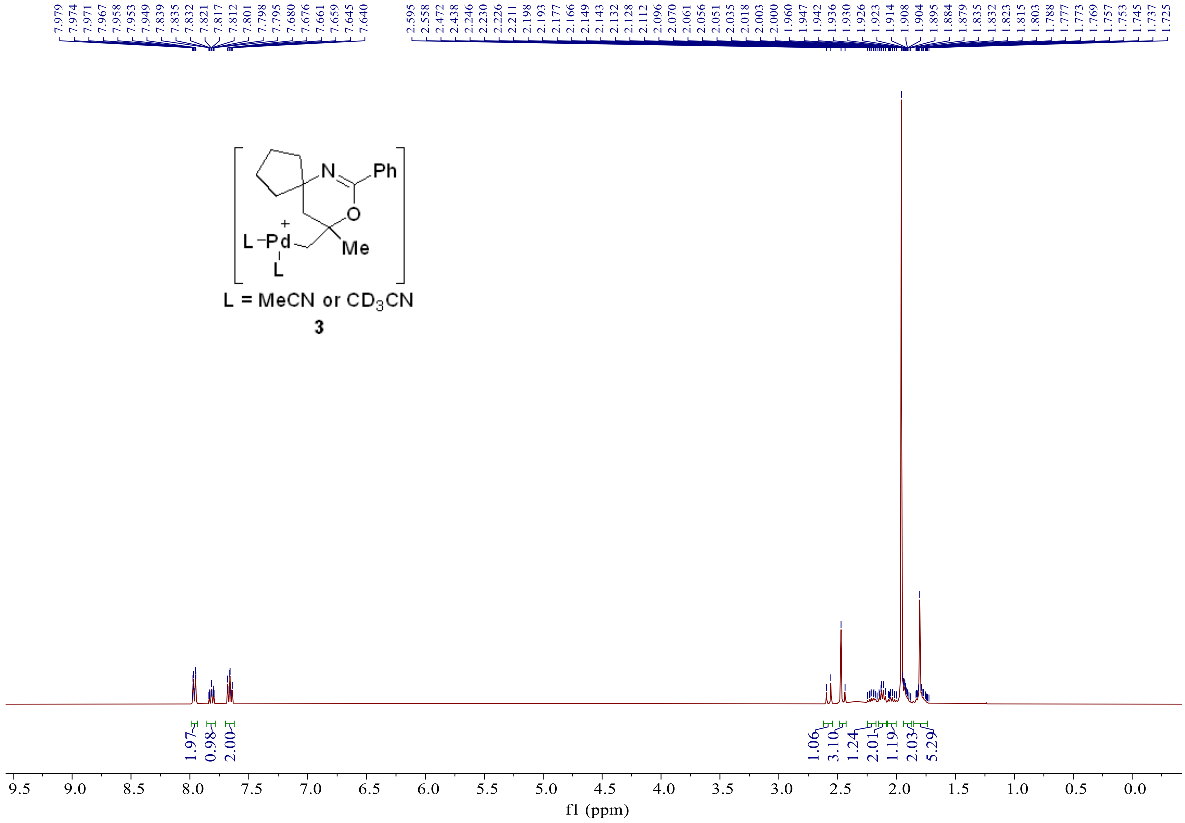

^{13}C NMR spectrum of **5** (151 MHz, Acetonitrile-d_3)

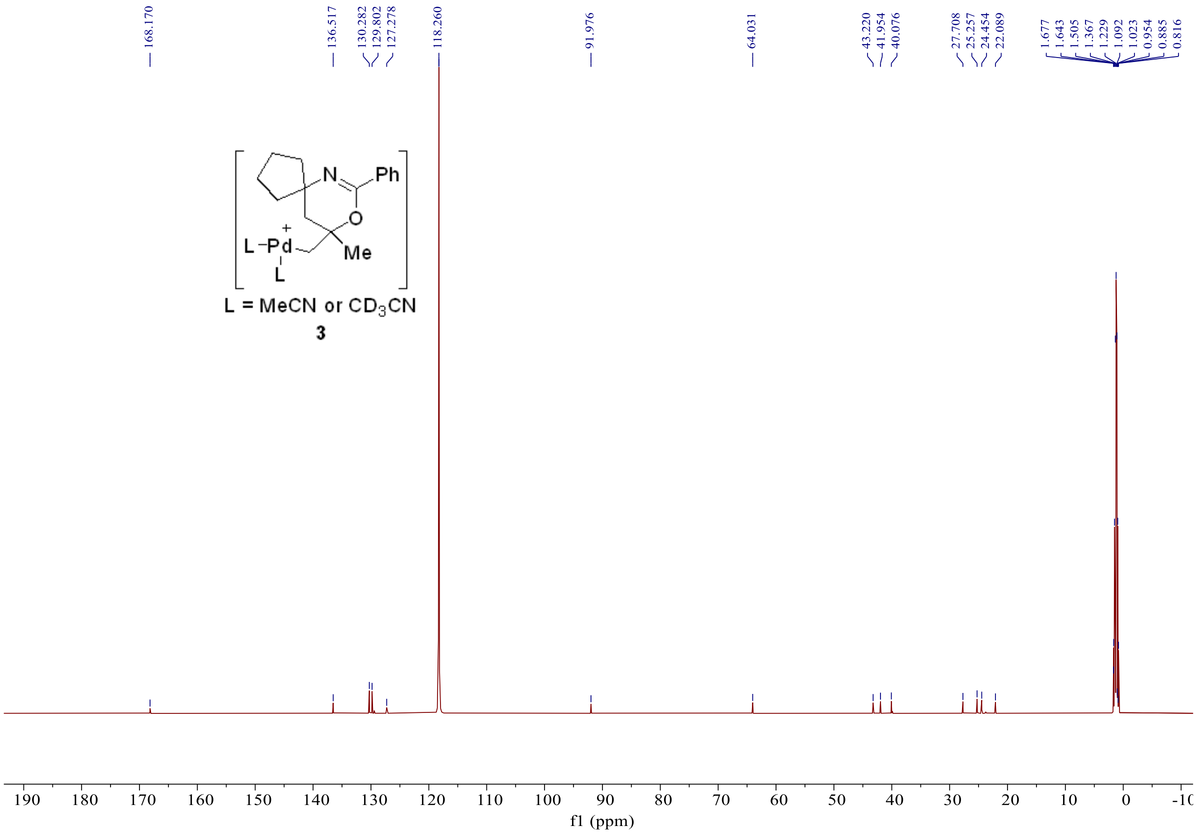

^{1}H NMR spectrum of **7** (600 MHz, $CDCl_3$)

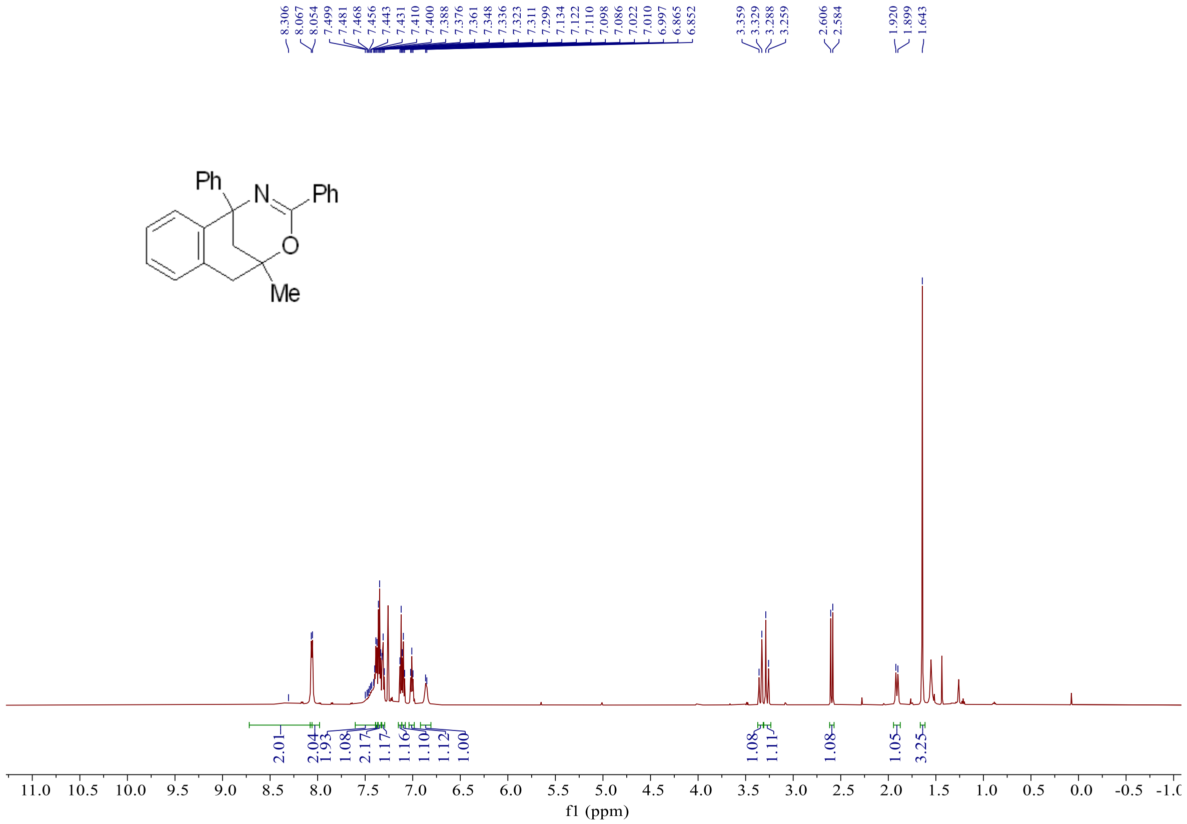

^{13}C NMR spectrum of **7** (151 MHz, $CDCl_3$)

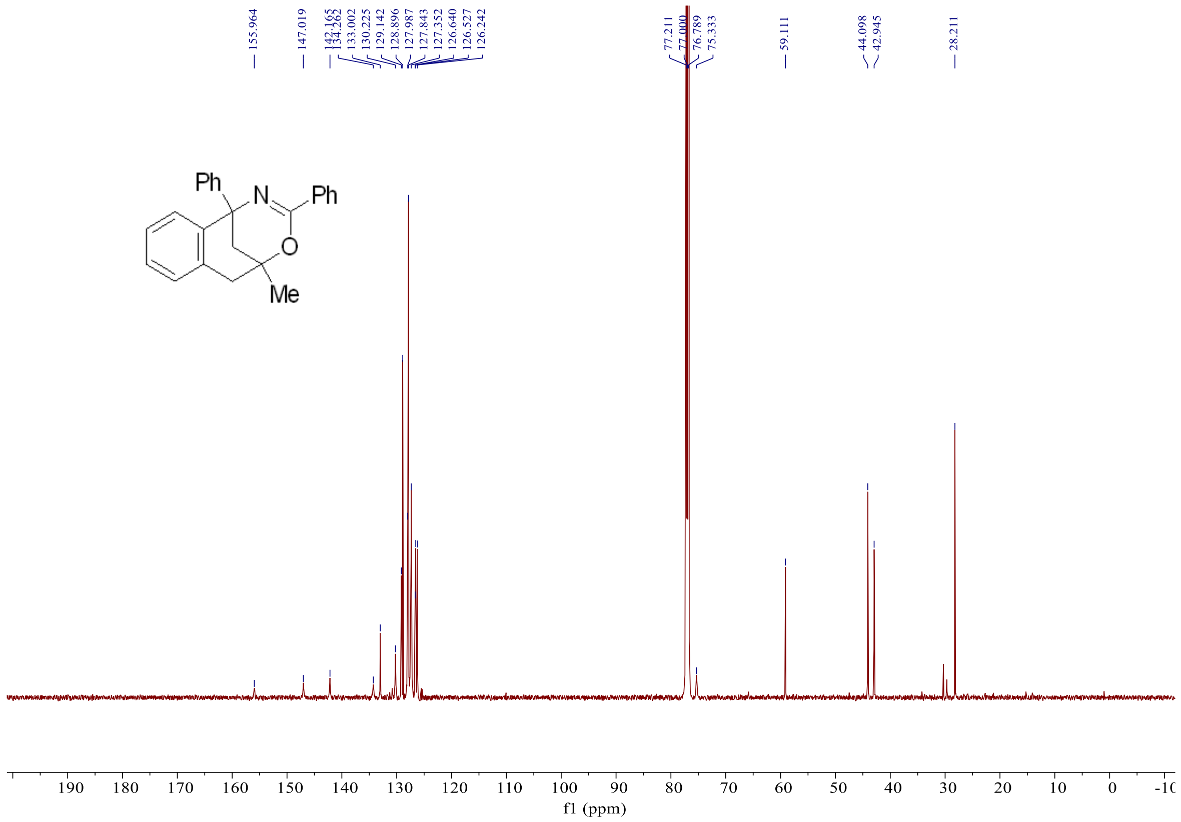

Supplement: Supplementary file 1 — Supporting Information [file ANIE-64-e202518735-s001.pdf]
